# Combination of Pseudo-LC-NMR and HRMS/MS-Based Molecular Networking for the Rapid Identification of Antimicrobial Metabolites From *Fusarium petroliphilum*

**DOI:** 10.3389/fmolb.2021.725691

**Published:** 2021-10-22

**Authors:** Abdulelah Alfattani, Laurence Marcourt, Valérie Hofstetter, Emerson Ferreira Queiroz, Sara Leoni, Pierre-Marie Allard, Katia Gindro, Didier Stien, Karl Perron, Jean-Luc Wolfender

**Affiliations:** ^1^ School of Pharmaceutical Sciences, University of Geneva, Geneva, Switzerland; ^2^ Institute of Pharmaceutical Sciences of Western Switzerland, ISPSO, University of Geneva, Geneva, Switzerland; ^3^ Institute for Plant Production Sciences IPS, Agroscope, Nyon, Switzerland; ^4^ Microbiology Unit, Department of Botany and Plant Biology, University of Geneva, Geneva, Switzerland; ^5^ Laboratoire de Biodiversité et Biotechnologie Microbienne, USR3579, CNRS, Sorbonne Université, Banyuls-sur-mer, France

**Keywords:** *Posidonia oceanica*, fungal endophyte, *Fusarium petroliphilum*, UHPLC-HRMS/MS molecular networking, high-resolution semi-preparative HPLC, pseudo-LC-NMR, antimicrobial natural products, anti-quorum sensing assays

## Abstract

An endophytic fungal strain isolated from a seagrass endemic to the Mediterranean Sea (*Posidonia oceanica*) was studied in order to identify its antimicrobial constituents and further characterize the composition of its metabolome. It was identified as *Fusarium petroliphilum* by in-depth phylogenetic analyses. The ethyl acetate extract of that strain exhibited antimicrobial activities and an ability to inhibit quorum sensing of *Staphylococcus aureus*. To perform this study with a few tens of mg of extract, an innovative one-step generic strategy was devised. On one side, the extract was analyzed by UHPLC-HRMS/MS molecular networking for dereplication. On the other side, semi-preparative HPLC using a similar gradient profile was used for a single-step high-resolution fractionation. All fractions were systematically profiled by ^1^H-NMR. The data were assembled into a 2D contour map, which we call “pseudo-LC-NMR,” and combined with those of UHPLC-HRMS/MS. This further highlighted the connection within structurally related compounds, facilitated data interpretation, and provided an unbiased quantitative profiling of the main extract constituents. This innovative strategy led to an unambiguous characterization of all major specialized metabolites of that extract and to the localization of its bioactive compounds. Altogether, this approach identified 22 compounds, 13 of them being new natural products and six being inhibitors of the quorum sensing mechanism of *S. aureus* and *Pseudomonas aeruginosa*. Minor analogues were also identified by annotation propagation through the corresponding HRMS/MS molecular network, which enabled a consistent annotation of 27 additional metabolites. This approach was designed to be generic and applicable to natural extracts of the same polarity range.

## Introduction

The rapid and efficient identification of novel natural products (NPs) in complex biological systems is a priority for the search for new lead compounds (Atanasov et al., 2021). In this context, the development of approaches that allow both rapid and unambiguous identification of natural compounds and estimation of their biological activity is key to NP research. This is particularly true in the context of the search for anti-infective compounds from microorganism cultures that are often obtained on a small scale in screening programs dedicated to finding new molecules to combat the challenging resistance problems in the field (Paytubi et al., 2017).

Following physical isolation, a classical approach for the structural determination of a metabolite naturally consists of combining high-resolution mass spectrometry (HRMS) and nuclear magnetic resonance (NMR) data to obtain a definitive identification (Breton and Reynolds, 2013; Halabalaki et al., 2014). To speed up this process, HPLC coupled with NMR has proven to be an interesting alternative to working directly in mixtures but also has some limitations. On-line and at-line LC-^1^H-NMR hyphenations have permitted us to partly characterize metabolites in crude extracts (Bohni et al., 2014). However, they are limited by their low sensitivity and resolution, in addition to practical issues such as solvent compatibility and solvent suppression (Exarchou et al., 2005; Wolfender et al., 2005). One way to solve these problems and to improve both spectral quality and sensitivity was the introduction of solid phase extraction (SPE) in the LC-NMR process, which resulted in the development of the LC-SPE-NMR (Lambert et al., 2007; Gebretsadik et al., 2021). Several recent works have demonstrated the benefits of this approach to working on natural extracts (Silva et al., 2018; Chu et al., 2019; Li et al., 2019). A limitation, however, may be that this approach often requires repeated collection of chromatographic peaks in order to yield sufficient amounts of metabolites from given LC peaks of interest.

One of the main axes of research on NPs is the discovery of new antibiotics. In this context, the integration of new approaches to rapidly identifying antibacterials is of great interest. Indeed, the emergence of bacterial strains resistant to classical antibiotics represents a major health problem (Hernando-Amado et al., 2019). New chemical entities with original activity profiles are particularly needed in drug discovery to fight such multi-resistant pathogenic bacterial strains (Brown and Wright, 2016). Among these multi-resistant bacteria, special attention must be paid to methicillin-resistant *Staphylococcus aureus* (MRSA) and *Pseudomonas aeruginosa* (PA). The gram-positive bacterium *S. aureus* causes superficial and potentially fatal infections, such as sepsis and pneumonia (Holden et al., 2013; Foster et al., 2014). The gram-negative bacterium *P. aeruginosa* is an opportunistic pathogen considered to be life-threatening to immunocompromised patients and to cystic fibrosis patients. In addition, it is a major cause of sepsis upon burn injuries (Church et al., 2006). Unfortunately, currently available antibiotics are often ineffective against multi-resistant bacterial strains due to the loss of their efficacy against what are now called “superbugs” (Cordell, 2000; Foster et al., 2014). In this respect, the discovery of molecules which are capable of blocking quorum sensing (QS) could offer a promising alternative to current antibiotics. Indeed, QS or cell-to-cell communication in bacteria is a regulatory process, governed by chemical signaling, that ensures sufficient cell density before inducing the expression of certain genes at the same time throughout the bacterial population. In the case of pathogens, these genes often code for virulence factors. The disruption of this system can therefore limit the virulence of pathogenic bacteria (Bassler and Losick, 2006; Ng and Bassler, 2009; Saeki et al., 2020).

In this context, NPs and their derivatives represent a historical source of unique chemical scaffolds with potential anti-infective properties. They represent 55% of FDA-approved antibiotics introduced in the period of 1981–2019 (Newman and Cragg, 2020). Today, intense research is still ongoing (Schneider, 2021), which led, for example, to the discovery of plazomicin, a recent FDA-approved antibiotic which targets multi-drug–resistant Enterobacteriaceae (Saravolatz and Stein, 2019). This underlines the importance of NPs as a valuable source of chemical entities for new treatments of bacterial diseases. At present, the majority of naturally originated antibiotics have been isolated from soil microorganisms. Thus, investigation of specialized metabolites from marine microbial strains in this regard is an expanding field (Blunt et al., 2018; Sun et al., 2019).

Microbial communities which often exist in competitive environments with other strains are evolving specialized metabolite pathways to produce a wide range of chemical entities which could be an interesting source of novel NPs with antibiotic activity (Peric-Concha and Long, 2003). In this relation, metabolomics study of endophyte communities is a first step to orienting further drug discovery approaches on such sources. Endophytes are organisms, often fungi and bacteria, that live inside plant tissues (Nisa et al., 2015). They establish different relationships with plants that vary from symbiotic to bordering on pathogenic. Endophytes have shown promising potential as a source of bioactive NPs by evolving the diversity of specialized metabolites (Gouda et al., 2016).

Among the possible sources of NPs from endophytes, the marine-derived fungus *F. petroliphilum* isolated from *Posidonia oceanica* (Posidoniaceae) was chosen in this work. *P. oceanica* is an underwater seagrass endemic to the Mediterranean Sea; it forms dense meadows from the surface down to a depth of 40 m. This marine vascular plant is known for its longevity and being, potentially, the host for a diverse microbial community including endophytes and epiphytes (Cuomo et al., 1985; Panno et al., 2013). However, only a few studies on *Posidonia* reported true fungal endophytes (Torta et al., 2015; Vohnik et al., 2015). *P. oceanica* plays an important role at the ecological and sedimentary levels (Vassallo et al., 2013); however, the species is endangered due to intense human activities. In this study, we hypothesize that the interactions between different microorganisms in such a closed environment and the long-living characteristic of *P. oceanica* would result in NP biosynthesis, which could be of bioactive interest. Furthermore, a better understanding of its endophytic community and its biosynthetic potential is of interest to better preserving this marine plant. To this end, we investigated the metabolome of *F. petroliphilum* in depth for its composition novelty and antimicrobial activity.

In the present work and from a methodological aspect, we developed an alternative approach to LC-SPE-NMR to obtain consecutive NMR spectra of all fractions from a single high-resolution semi-preparative HPLC injection which is hereafter defined as pseudo-LC-NMR. This approach enables NMR analyses obtained at the semi-preparative level to be linked to UHPLC-HRMS metabolite profiling on the analytical scale with high spectral quality data on both NMR and MS dimensions. The combination of these HRMS/MS and NMR data, fraction by fraction, often allows an unambiguous identification of the metabolites present and an estimation of their amounts in parallel with biological activity tests. On the other hand, working on a semi-preparative scale enables a collection of numerous metabolites in the low mg range which is compatible with different types of biological assays. This approach was applied to a strain of *F. petroliphilum* and allowed us to identify metabolites responsible for antibacterial activity. It also provided a good overview of the metabolome composition of this marine endophyte.

## Results and Discussion

In order to identify a fungal strain that produces bioactive compounds and to rapidly identify the metabolites responsible for this activity, the following procedure was applied in this study. *1*) A bioactivity screen was performed on a set of *Posidonia* fungal endophytes. *2*) The most active strain was selected for the study. *3*) An aliquot from the extract was subjected to UHPLC-HRMS and automated data-dependent acquisition MS/MS for metabolite profiling, followed by dereplication of known compounds through molecular network (MN) analysis. *4*) Since the annotated compounds were not reported to act as QS inhibitors, their unambiguous characterization had to be performed. *5*) In order to localize and identify the bioactive compounds, the fungal extract was thoroughly analyzed by pseudo-LC-NMR. The pseudo-LC-NMR process was developed for the purpose of this study but was intended to be generic in order to rapidly provide complementary NMR information to the LC-MS metabolite profiling of crude extracts available in limited amounts.

**FIGURE 1:**
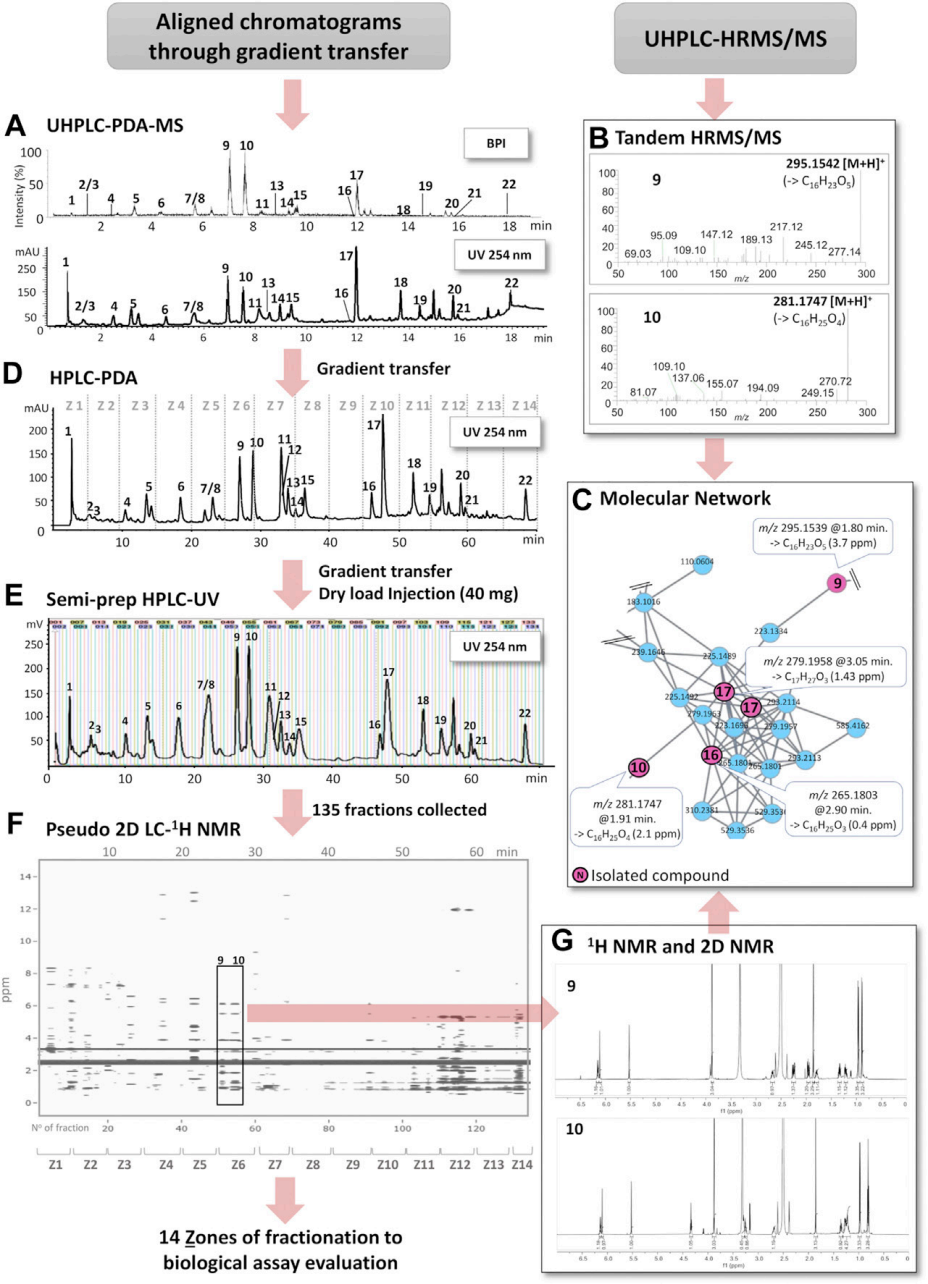
Schematic overview of the workflow of this study. **(A)** Optimized UHPLC-DAD-MS profile of the ethyl acetate crude extract (base peak intensity (BPI), PI mode) and UV trace (254 nm). **(B)** HRMS/MS spectra of the LC peaks from the UHPLC-HRMS/MS extract profile corresponding to the isolated compounds **9** and **10**. **(C)** Selected part of an MN cluster highlighting the features of **9**, **10**, **16**, and **17** (series of structurally related pyrone derivatives). Some of the other features were dereplicated based on MN annotation propagation (see Table 2). **(D)** HPLC-DAD chromatogram after gradient transfer from **(A)**. Z1 to Z14 indicate chromatographic zones combined in semi-preparative HPLC for the bioassay monitoring, shown on **(D)** for a clearer display. **(E)** Semi-preparative HPLC-UV chromatogram after gradient transfer from **(D)** and dryload sample introduction; vertical lines represent collected fractions. **(F)** Pseudo-LC-NMR obtained by a combination of the ^1^H-NMR spectra of all fractions from **(E)** into a single matrix (ppm vs. R_t_ or fraction N^º^). **(G)** ^1^H-NMR spectra of fractions F51 and F55 highlighted in the 2D plot **(F)**.

The key steps for pseudo-LC-NMR were as follows: *1*) an optimized geometrical transfer was applied from the scale of UHPLC to analytical HPLC and then to semi-preparative HPLC (Figures 1A,D,E). *2*) The crude extract was injected by LC on the semi-preparative scale with automated fraction collection every 30 s. *3*) All fractions were submitted to ^1^H-NMR analyses, followed by LC-HRMS/MS. *4*) ^1^H-NMR spectra were stacked and plotted in a 2D map sequenced according to the retention time generating the pseudo-LC-NMR plot (Figure 1F). *5*) The concomitant processing of the pseudo-LC-NMR with the LC-MS profile allowed us to increase the level of annotation and to conduct specific 2D-NMR experiments when needed.

In order to localize the bioactivity, the fractionation strategy had to be adapted to the sensitivity of the assays. In this specific study, the measurement of antibacterial and anti-QS activity required larger quantities than those obtained from the 30 s/fraction on the semi-preparative scale described above. Thus, a pooling of fractions was designed to highlight the area of chromatographic activity (Figures 1D,E). This finally permitted the conducting of a specific targeted purification of bioactive compounds on an enriched extract with the same experimental setting.

### Identification of the Fungal Strain

**FIGURE 2:**
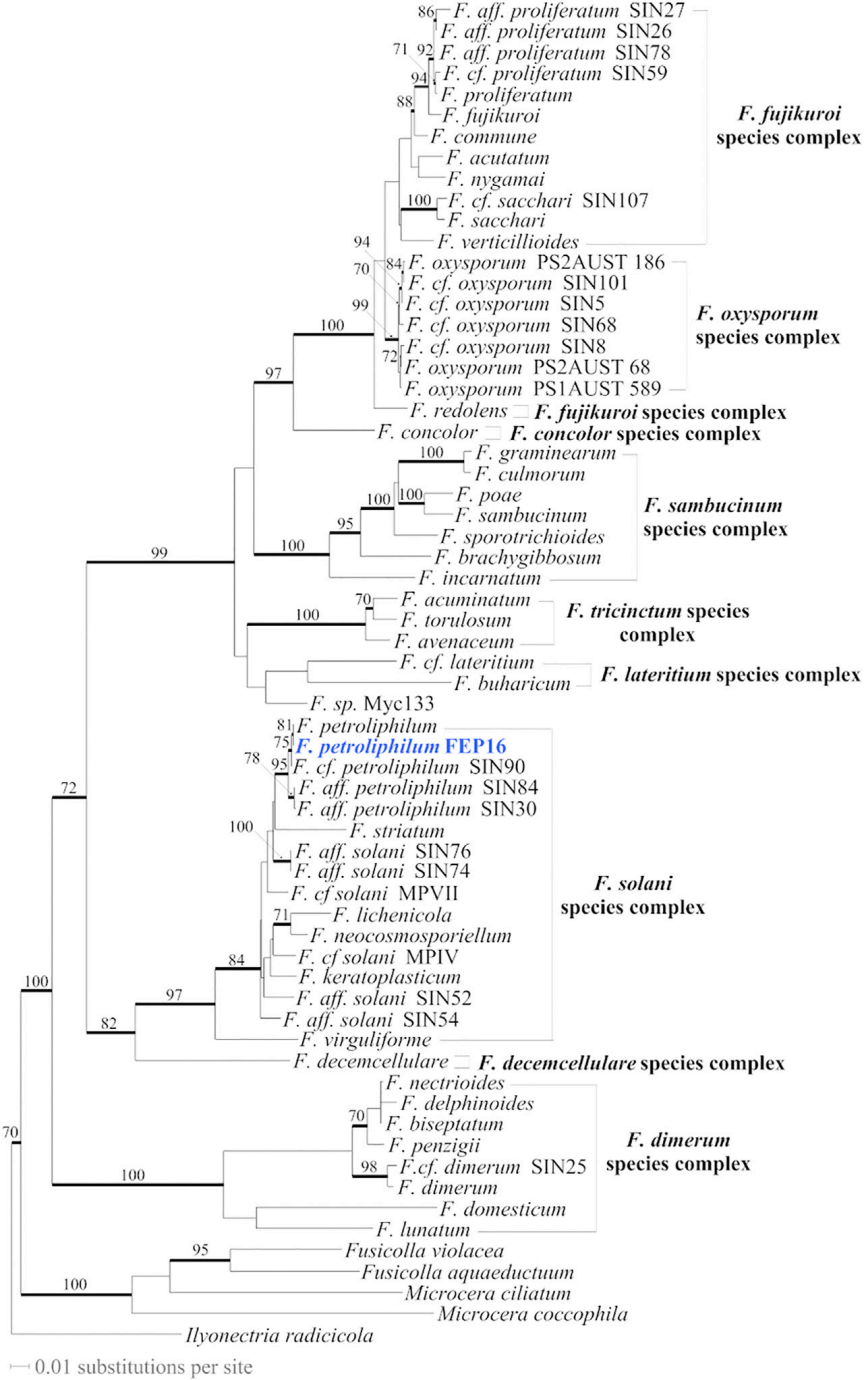
Most likely tree (-ln = 20922.71682) inferred from the *Fusarium* 5 locus-64 taxa dataset (alignment length after exclusion of ambiguously aligned regions = 2442 characters (char); calmodulin = 142 char, beta-tubulin = 262 char, ITS = 364 char, *RPB2* = 1500 char, TEF-1 = 174 char). The fungal collection newly sequenced for that study is in blue. Branches that received significant bootstrap (BS) values (≥70%) are in bold with BS values reported along the branches.

Although ITS is presently the best barcode sequence for fungi (Schoch et al., 2012), it did not allow the assignment of the selected fungal strain, FEP 16, at the species rank. The BLAST top score results in GenBank (GB) indicated that the FEP 16 ITS sequence was 100% similar with a 100% coverage to the sequences of several species: *F. petroliphilum* (Q.T. Chen and X.H. Fu) (D. Geiser, O’Donnell, Short and Zhang) (2013) (GB accession number: LC512834), *F. macroceras* (Wollenweber and Reinking 1925) (MH854821), and *F. solani* (Mart. 1842) (MH855493). These species, based on ITS BLAST results, belong to the *F. solani* species complex in which many species are still not formally described (Short et al., 2013; Chehri et al., 2015; O'Donnell, 2019). Trying to further determine to which species FEP 16 belongs, we sequenced four more loci for that strain (see the experimental section) and combined these data with data sampled in the study by Bohni et al. (2016). Unfortunately for *F. macroceras*, only ITS and part of the nuclear large subunit (28S) were available in GB. After a similarity search of the 28S (MH866321) of *F. macroceras* in GB, that sequence appeared to be 100% similar to several sequences for *F. solani* (i.e., AY097317) with 100% sequence coverage but also to four sequences for *F. petroliphilum* (i.e., MH874378) but with 90% sequence coverage. Consequently, these two taxa are likely to be identical but without sequences for other more variable loci (*RPB2*, beta-tubulin, and calmodulin) for *F. macroceras*, and without type sequences for these species, it is not possible to clarify the situation. Combined analyses for the *Fusarium* 5 locus-64 taxa (Figure 2) (Supplementary Table S1) allowed us to identify FEP 16 as *F. petroliphilum*.

### Evaluation of the Antibacterial Activity of the Crude Extracts

The minimum inhibitory concentration (MIC) of the extracts of all strains was tested against a methicillin-resistant strain of *Staphylococcus aureus* (ATCC 33591-MRSA) and a strain of *Pseudomonas aeruginosa* (ATCC 27853) (Supplementary Table S2). The crude extract of the fungus *F. petroliphilum* (FEP 16) presented an antibacterial activity against MRSA with an MIC at 32 μg/ml but no activity against the Gram-negative *P. aeruginosa* strain.

In order to further verify if activity could be revealed at the level of quorum sensing (QS) inhibition for *P. aeruginosa*, we performed a reporter assay based on percentage of fluorescence to evaluate the potential of the crude extract on QS (Hentzer et al., 2002). Interestingly, the crude extract displayed about 80% reduction of the fluorescence level of 2 reporter genes (*pqsA* and *lasB*) normally induced by QS in *P. aeruginosa* (Table 1). The *pqsA* gene encodes an enzyme involved in the synthesis of PQS, which is a signaling molecule of QS, while the *lasB* gene encodes an important elastase enzyme (Jimenez et al., 2012). These primary bioassay results encouraged us to go for further investigations.

**TABLE 1:** Minimum inhibitory concentration (MIC) and quorum sensing inhibition (QS) assays of crude extract compared to several antibiotics.

	MIC (µg/ml)		QS (% of fluorescence)	
	MRSA	PA	PA (*pqsA*)	PA (*lasB*)
Crude extract (small scale)	32	>128	21	19
Crude extract (large scale)	32	>128	20	25
Azithromycin	NA	10	32	39
Gentamicin	1	NA		
Tetracycline	16			
Erythromycin	16			
Chloramphenicol	32			

Values show the mean of triplicates. Test results were compared to DMSO fixed at 100%. MRSA, methicillin-resistant Staphylococcus aureus; PA, Pseudomonas aeruginosa; NA, not applicable.

### Metabolite Dereplication by UHPLC-HRMS/MS

In order to have a primary overview of the crude extract chemical content, it was analyzed by UHPLC-HRMS/MS in positive (PI) and negative (NI) ionization modes; MS/MS spectra of all detected features were recorded by data-dependent analysis (DDA). Feature-based molecular networks (Nothias et al., 2020) for both modes were built to arrange the extracted ions (precursor ions) into clusters based on MS/MS similarity. This process was done by filtering ions below an intensity threshold at 10^6^, which yielded 1900 features in the PI mode and 2270 in the NI mode for building the corresponding MNs (Supplementary Figures S1, S2). The precursor masses and their associated MS/MS spectra were matched against experimental data from Global Natural Products Social Molecular Networking (GNPS) (Wang et al., 2016) and predicted spectra obtained using the *in silico* MS/MS fragmentation database (ISDB-DNP) (Allard et al., 2016).

For each node of the MN, possible structure candidates were listed according to MS/MS similarity (initial rank) up to a maximum of the top 50 compounds reported to occur in fungi. To increase the level of confidence in annotation, a reweighting step based on taxonomy was performed. This step takes into consideration the matching between the biological source reported in the DNP at the level of species > genus > family, resulting in a maximum of the top 5 candidates (final rank) for each annotated node (Rutz et al., 2019). As a result, in the PI mode, 823 of the 1900 detected features were annotated, 214 of them were found in the family Nectriaceae, 148 of them were present in the genus *Fusarium*, and 28 features were reported in the closely related species *F. solani*. In the NI mode, 705 features were annotated out of 2720, 200 of them belonged to Nectriaceae, 135 of them were reported in the same genus, and 19 of them have been found in *F. solani*. The annotated metabolites corresponding to the most intense MS peaks detected in both PI (threshold > 5 × 10^7^) and NI (threshold > 10^7^) are presented in Table 2. Their annotation was further refined by taxonomical ranking and by structural consistency of corresponding clusters. Compounds not previously found in the Nectriaceae family at least were not considered in this LC-MS dereplication process. The full MNs with the complete annotation are deposited in Figshare (https://doi.org/10.6084/m9.figshare.14706198.v1). The same MN will be used later on in this study to apply annotation propagation through the location of isolated molecules in the MN.

**TABLE 2:** Annotated compounds by UHPLC-HRMS/MS in both PI and NI modes in the crude extract of *F. petroliphilum*.

N° F.	N° C.	R_t_	C. index	Isolated as	Annotated as	T. score	Chemical class	MF	I. mode	*m/z*	Error (ppm)
F4	1	1.03	Sgl	Adenosine	Adenosine	3	Purine nucleoside	C_10_H_13_N_5_O_4_	[M+H]^+^	268.1039	−1.49
F10	3	2.31	19	6-(1-hydroxyethyl)-3-methyl-2H-pyran-2-one	-	3	Pyrone	C_8_H_10_O_3_	[M+H]^+^	155.0703	−3.22
F10	2a	2.21	155	6-(2,3-dihydroxybutan-2-yl)-3-methyl-2H-pyran-2-one	Fusanolide B	3	Pyrone	C_10_H_14_O_4_	[M+H]^+^	199.0967	−2.51
F10	2b	2.27	155	Isomer of 2a	Fusanolide B	3	Pyrone	C_10_H_14_O_4_	[M+H]^+^	199.0872	−1.51
-	-	2.45	3	-	Arthropsolide A	2	Polyketide	C_13_H_14_O_5_	[M−H]^−^	249.0766	−1.2
-	-	2.47	145	-	Fusaquinone A	2	Naphthofuran	C_16_H_18_O_6_	[M+H]^+^	307.1178	−1.17
-	-	2.56	145	-	Fusarnaphthoquinone B	2	Naphthofuran	C_15_H_16_O_5_	[M+H]^+^	277.1066	−3.61
-	-	2.59	9	-	Diaporthin	1	Benzopyone	C_13_H_14_O_5_	[M+H]^+^	251.0911	−3.65
F20	4	2.65	329	Gibepyrone D	Gibepyrone D	2	Pyrone	C_10_H_10_O_4_	[M−H]^−^	193.0498	−1.55
F26	5	2.71	37	Aloesol	Aloesol	2	Benzopyran	C_13_H_14_O_4_	[M+H]^+^	235.0963	−2.97
			Sgl						[M−H]^−^	233.0821	3.42
-	-	2.74	Sgl	-	Cladobotrin V	2	Pyranone	C_10_H_12_O_4_	[M−H]^−^	195.0655	−0.85
-	-	2.95	222	-	Javanicin O-De-Me	2	Naphthoquinone	C_14_H_12_O_6_	[M−H]^−^	275.0564	3.08
-	-	3.01	Sgl	-	Gibepyrone A	3	Pyranone	C_10_H_12_O_2_	[M−H]^−^	163.0753	−0.22
-	-	3.2	19	-	Fusarpyrone A	3	Pyran	C_10_H_12_O_3_	[M+H]^+^	181.0858	0.8
-	-	3.23	222	-	5,8-dihydroxy-6-hydroxymethyl-7-(2-hydroxypropyl)-2-methoxy-1,4-naphthoquinone	3	Naphthoquinone	C_15_H_15_O_7_	[M−H]^−^	307.0825	2.44
-	-	3.24	9	-	Fusarubin 5-deoxy	3	Benzoquinone	C_15_H_14_O_6_	[M+H]^+^	291.086	−2.8
-	-	3.37	207	-	Bostrycoidin 6-deoxy	3	Quinone	C_15_H_11_NO_4_	[M+H]^+^	270.0759	−2.76
F35	6	6.63	207	5-hydroxy-4-(hydroxymethyl)-8-methoxy-2-methyl-1H-benzo[g]indole-6,9-dione	-	3	Quinone	C_15_H_13_NO_5_	[M+H]^+^	288.0868	−1.38
-	-	3.37	5	-	Sescandelin 1'-ketone	1	Isocoumarin	C_11_H_8_O_5_	[M−H]^−^	219.0295	1.6
-	-	3.85	306	-	5-acetyl-1,2,4,6-tetrahydroxyanthraquinone2-me ether	2	Quinone	C_17_H_12_O_7_	[M+H]^+^	329.0636	2.6
F43	7	3.86	9	Fusarubin	Fusarubin	3	Benzoquinone	C_15_H_14_O_7_	[M+H]^+^	307.0823	1.62
F44	8	3.86	-	3-O-methylfusarubin	3-O-methylfusarubin	3	Quinone	C_16_H_16_O_7_	[M−H]^−^	319.0825	2.19
-	-	3.86	45	-	Anhydrofusarubin	3	Quinone	C_15_H_12_O_6_	[M+H]^+^	289.0715	1.03
-	-	3.87	54	-	3-acetonyl-2,5,8-trihydroxy-6-methoxy naphthoquinone	3	Naphthoquinone	C_14_H_12_O_7_	[M−H]^−^	291.0511	1.04
F51	9	3.96	25	(6E)-7-(4-methoxy-6-oxo-6H-pyran-2-yl)-3,5-dimethyloct-6-enoic acid	7S-hydroxy-O-demethyllasiodiplodin	3	Pyran	C_16_H_22_O_5_	[M+H]^+^	295.1544	−3.72
-	x	3.97	54	-	Rhodolamprometrin	2	Anthracene	C_16_H_10_O_7_	[M−H]^−^	313.0352	1.13
F55	10	4.4	29	6-((E)-6-ethyl-7-hydroxy-4-methylhept-2-en-2-yl)-4-methoxy-2H-pyran-2-one	-	3	Pyrone	C_16_H_24_O_4_	[M+H]^+^	281.1745	−2.13
-	-	4.54	45	-	Solaniol or karuquinone C	3	Naphthalene	C_15_H_16_O_6_	[M+H]^+^	293.1017	−2.73
		4.55	54						[M−H]^−^	291.0885	4.8
-	-	4.61	45	-	Dihydroanhydrojavanicin	3	Naphthofuran	C_15_H_14_O_5_	[M+H]^+^	275.0912	−1.89
F60	11	5.63	19	Bostrycoidin	Bostrycoidin	3	Quinone	C_15_H_11_NO_5_	[M+H]^+^	286.0695	−0.34
F62	12	5.34	Sgl	5β,6β-23,26-diepoxy-3β,7α,9α-trihydroxy-(20Z,23S,24S,25R)ergosta-8(14),20-dien-15-one	-	3	Steroid	C_28_H_40_O_6_	[M+Na]^+^	495.2706	−3.23
F64	13	5.44	86	2-(2,3,5,6,7,7a-hexahydro-1-((*E*)-6-hydroxy-5,6-dimethylhept-3-en-2-yl)-7a-methyl-5-oxo-1H-inden-4-yl)acetic acid	-	3	Cyclohexenone	C_21_H_32_O_4_	[M+H]^+^	349.2378	−0.28
		5.44	Sgl						[M−H]^−^	347.2228	1.72
F66	14	5.68	86	2-(2,3,5,6,7,7a-hexahydro-1-((*E*)-7-hydroxy-5,6-dimethylhept-3-en-2-yl)-7a-methyl-5-oxo-1H-inden-4-yl)acetic acid	-	3	Cyclohexenone	C_21_H_32_O_4_	[M+H]^+^	349.2386	2
			Sgl						[M−H]^−^	347.2232	2.87
F69	15	3.38	7	5-hydroxy-8-methoxy-2,4-dimethyl-1H-benzo[g]indole-6,9-dione	-	3	Quinone	C_15_H_13_NO_4_	[M+H]^+^	272.0923	0.1
-	-	7.05	173	-	kauranoic acid ent-16β-hydroxy-19	2	Fatty acid	C_20_H_32_O_3_	[M+H]^+^	321.2442	2.1
-	-	7.81	19	-	Fusarone	2	Cyclopentanone	C_14_H_22_O_3_	[M+H]^+^	239.1661	0.5
F91	16	8.89	29	4-methoxy-6-((*E*)-4,6-dimethyloct-2-en-2-yl)-2H-pyran-2-one	-	3	Pyrone	C_16_H_24_O_3_	[M+H]^+^	265.1805	0.37
F93	17	9.6	Sgl	4-methoxy-3-methyl-6-((*E*)-4,6-dimethyloct-2-en-2-yl)-2H-pyran-2-one	-	3	Pyrone	C_17_H_26_O_3_	[M+H]^+^	279.1956	−1.43
F103	18	11.34	Sgl	2-(2,3,5,6,7,7a-hexahydro-7a-methyl-1-((*E*)-5,6-dimethylhept-3-en-2-yl)-5-oxo-1H-inden-4-yl)acetic acid	-	3	Cyclohexenone	C_21_H_32_O_3_	[M+H]^+^	333.2426	0.3
									[M−H]^−^	331.228	2.11
107	19	14.5	-	3-O-β-D-glucopyranoside-stigmast-8-en-3-ol	-	3	Steroid	C_35_H_60_O_6_	[M+Na]^+^	599.428	−1.16
F116	20	14.6	-	24R/24S cerevisterol	-	3	Steroid	C_28_H_46_O_3_	[M+Na]^+^	453.3397	11.6
F117	21	14.27	-	6-dehydrocerevisterol	-	3	Steroid	C_28_H_44_O_3_	[M+H]^+^	429.3387	4.42
F122	x	16.48	-	-	3-acetoxy-2,3-dihydropiptoporic acid	1	Fatty acid	C_23_H_28_O_5_	[M-H]^−^	383.1898	1.8
F132	22	13.62	-	Ergosterol	Ergosterol	3	Steroid	C_28_H_44_O	[M−H]^−^	395.3325	2.78

N° F., Number of semi-preparative fractions; N° C., Isolated compounds **1**–**22**; R_t_, retention time in UHPLC-HRMS/MS analysis of the extract; C. index, component index (cluster number in MN); Sgl, singleton node; T. score, taxonomically informed score; I.mode, ionization mode; 1, family (Nectriaceae); 2, genus (*Fusarium*); 3, species (*F. solani*); MF, molecular formula.

Most of the annotated metabolites belong to pyran and pyrone derivatives, furan lactones, naphthoquinones, isocoumarins, terpenes, and sterols. Such data are consistent with previously reported studies on the chemical content of *Fusarium* species (Wei and Wu, 2020), in particular, the presence of some frequently reported compounds in *Fusarium*, such as gibepyrone D (Wang et al., 2011), aloesol (Kashiwada et al., 1984), fusarubin (Tatum and Baker, 1983), anhydrofusarubin (Shao et al., 2010), and bostrycoidin (Arsenault, 1965), which are highlighted in Table 2. As shown in Table 2, more than 30% of the most intense MS peaks could not be annotated through this process, which could be either unknown compounds or compounds never reported in the Nectriaceae family.

Based on these dereplication results and the bioactivity data measured on the extract, this prompted us to establish an efficient approach to obtaining complementary NMR data in line with metabolite profiling. This workflow is designed to run with amounts in the range of 30–60 mg of extract to preserve column resolution while maximizing sample loading. Such amounts of crude extracts are usually generated with solid culture of fungi in 10–20 petri dishes scale.

### Culture Scale-Up and Semi-Preparative HPLC Fractionation

In order to obtain enough material, the culture of *F. petroliphilum* was scaled up to 100 petri dishes under the conditions described in Materials and Methods. This yielded 300 mg of ethyl acetate crude extract which exhibited bioactivity results comparable to those obtained during screening (Table 1).

To effectively link the expected fractionation of this extract with the metabolite profiling data (see above), a chromatographic gradient transfer method was used to find the correct separation parameters, ideally for a single separation at the semi-preparative level. In practice, an intermediate step at the analytical HPLC level (Figure 1D) was necessary on a column having the exact same phase chemistry as the one used at the semi-preparative level (*see* experimental section). The optimum HPLC conditions were determined with UV monitoring at 254 nm. This latter linear gradient method was then geometrically transferred to the semi-preparative scale (Guillarme et al., 2008).

In order to obtain an efficient high-resolution separation of this complex mixture and to avoid any loss of chromatographic resolution, the crude extract (40 mg) was introduced into a dry loading cell according to our previously published protocol (Queiroz et al., 2019). Using this approach, it was possible to obtain equivalent separations on analytical and semi-preparative scales (Figures 1A,D,E). To match the high chromatographic resolution that was obtained, 135 fractions were automatically collected on the basis of one fraction per 30 s. All fractions were immediately dried under vacuum and weighted. This ensured a full removal of solvent for high-quality NMR profiling and estimation of the amount for the bioactivity assay to be performed.

### Combination of NMR Spectra for an Overview as a Pseudo-LC-NMR Plot

With the idea to obtain a comprehensive NMR profile of all fractions and be able to align all spectra over the extended polarity range of the various metabolites that were separated, each fraction was diluted in 600 µl of DMSO-*d*_6_ and submitted to ^1^H-NMR analysis. DMSO-*d*_6_ was selected since it is known to have good solubility properties and for its compatibility with bioassays. The ^1^H-NMR spectra of all fractions were obtained by automated acquisition (29 h of total acquisition). In order to visualize all ^1^H-NMR signals from the 135 fractions (F1-F135), a 2D plot was generated. For this, all individual spectra were binned and combined into a single matrix (ppm vs. R_t_ or fraction N^º^). This plot simulates the actual output of a classic on-flow LC-NMR analysis (Queiroz et al., 2002). The chromatographic dimension of the plot was expressed either as fraction number or retention time since all spectra were stacked according to their elution order, which allows a straightforward correlation with the corresponding analytical HPLC-DAD trace (Figure 1F). An interactive version of the plot can be explored here: (https://oolonek.github.io/pseudo_lcnmr_plotter/2dNMR.html).

**FIGURE 3:**
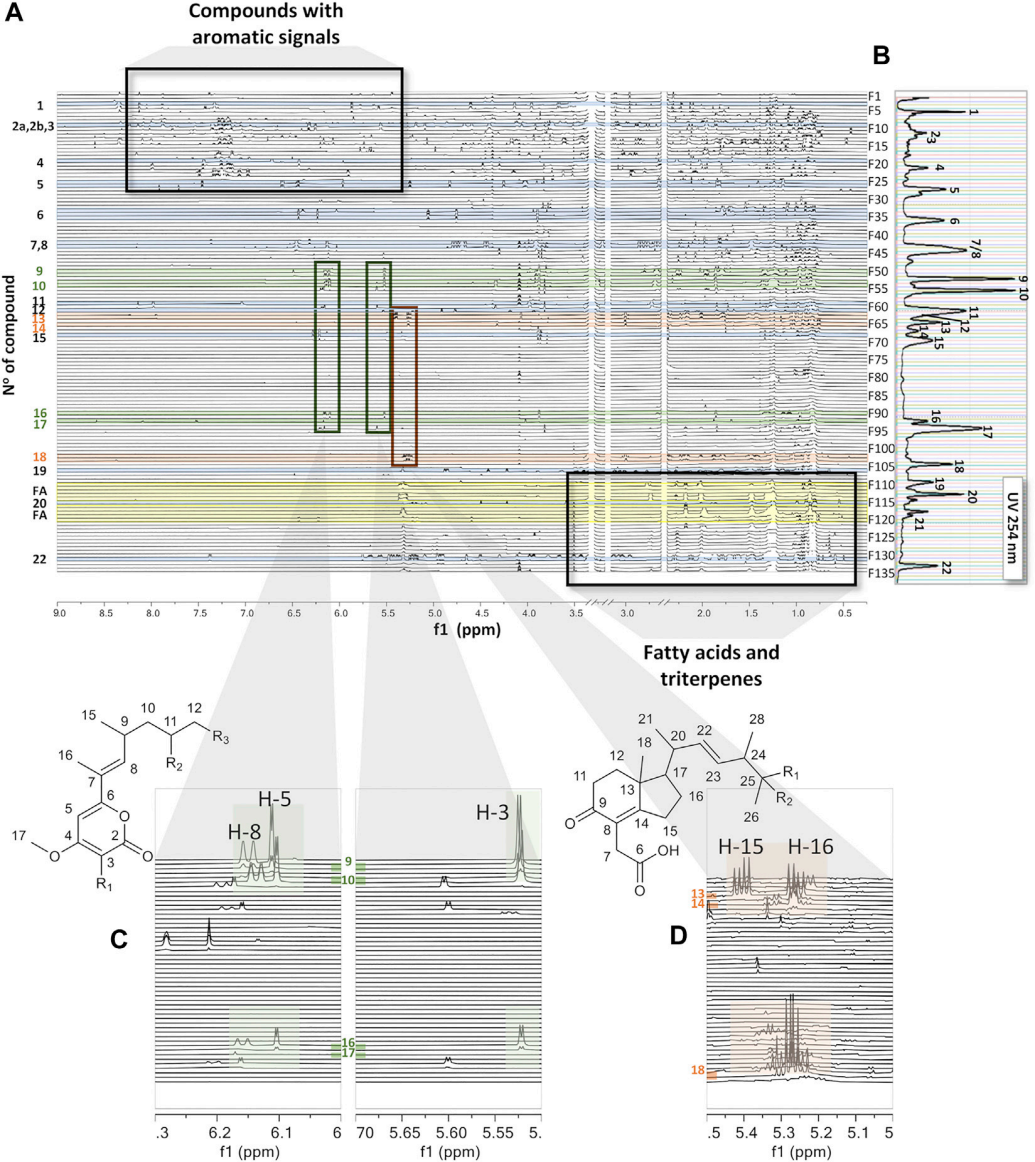
**(A)** Stacked view of the ^1^H-NMR spectra of all fractions ordered by retention time from the top to the bottom, all recorded in DMSO-*d*_*6*_ as the bridging solvent. The green shadow highlights common signals in fractions F51–56 in the regions of δ_H_ 5.5 to 5.7 and δ_H_ 6.0 to 6.3. The orange shadow highlights common signals in fractions F64–F65 and F103–F104 in the region of δ_H_ 5.2 to 5.3. The yellow shadow highlights the presence of fatty acids. **(B)** UV trace of the semi-preparative HPLC chromatogram for LC peak localization (Figure 1E). **(C)** Inset showing characteristic signals of the series across a set of fractions, F51–F56 and F90–F93. **(D)** Inset showing characteristic signals of the series across a set of fractions, F64–F65 and F103–F104.

The generated 2D plot from collected and dried micro-fractions has several advantages over classical LC-NMR. In this case, all ^1^H-NMR signals are perfectly aligned, since all spectra are recorded with the same solvent across the whole separation (DMSO-*d*_6_). By comparing our approach to on-flow LC-NMR, no solvent suppression is necessary. In addition, compared to at-line LC-NMR detection methods, such as LC-SPE-NMR (Chang et al., 2020), the spectra obtained are recorded from a single LC separation, and multiple injections for sample enrichment are not necessary. We define this workflow as a pseudo-LC-NMR analysis, which can be viewed either as a 2D plot (Figure 1F) or as a stacked view (Figure 3A) for a comprehensive analysis of the evolution of the ^1^H signals across the chromatographic dimension. Since the NMR response is directly proportional to the amounts of compounds, the overall observation of the 2D plot provides, in a first instance, an unbiased view of the molar ratios between constituents. However, this is of course also related to the number of magnetically diverse protons for each constituent.

In order to determine molar ratios between fractions, a histogram was created based on ^1^H-NMR peak integration, which represents total proton intensities of each recorded spectrum (Supplementary Figure S3A). This histogram was rather similar to the one obtained using weighed dry fractions (Supplementary Figure S3B) which shows mainly major apolar constituents eluting at the end of the chromatogram between fractions F110 and F132. Analysis of the related ^1^H-NMR spectra revealed signal patterns, which mainly correspond to fatty acids, that is, a methyl group at δ_H_ 0.5 and an intense signal from methylene protons around δ_H_ 1.25. A specific histogram based on the methylene chain signal found typically in fatty acids was built across all fractions; it allows us to highlight their presence mainly in fractions F110, 111, 114, 115, 117, 118, 119, 122, and 123 (Supplementary Figure S3C). This trace matches well with the ELSD detection as shown in Supplementary Figure S3D and demonstrates that the crude extract was dominated by apolar constituents. Most of these fatty acids were found to be unsaturated fatty acids and exhibited a characteristic signal of their ethylenic protons around δ_H_ 5.3, whereas fractions F117, 122, and 123 contained saturated fatty acids.

In addition to fatty acids, steroids and triterpenes were also identified in this chromatographic region. For example, ergosterol (**22**), a main constituent of the fungal membrane, could be identified in F132, yielding an intense signal in the histogram (Supplementary Figure S3A). Its ^1^H-NMR spectrum was in good agreement with reported data (Kwon et al., 2002; Yang et al., 2003) and confirmed the dereplication results (Table 2). Moreover, cerevisterol (**20**) could be confirmed in F116 by comparing its ^1^H-NMR spectrum with those in the literature (Kawagishi et al., 1988). Based on the extraction procedure, the presence of these main common apolar fungal constituents is not surprising. The analysis of the pseudo-LC-NMR 2D plot facilitated their identification and estimations of the ratio in an unbiased manner.

Further inspection of this pseudo-LC-NMR plot revealed the presence of specialized metabolites with aromatic signals (between δ_H_ 6.5 and 8.0), which were easily identified at the beginning of the chromatogram (F1-F27). In this part of the semi-preparative HPLC chromatogram, the LC-UV profile exhibited main peaks that were not fully separated (Figure 1E). The inspection of the 2D plot and the related ^1^H-NMR profile of each fraction was in line with this observation and exhibited spectra with overlapping signals. In this polar region, several compounds were dereplicated by HRMS/MS (Table 2) and confirmed by NMR upon comparison with reported data in the literature: adenosine (**1**) (Ciuffreda et al., 2007) was identified in fraction F4, gibepyrone D (**4**) (Wang et al., 2011) in fraction F20, and aloesol (**5**) (Kashiwada et al., 1984) in fraction F26. In this region of the chromatogram, arthropsolide A (Ayer et al., 1992) (Table 2) was dereplicated by HRMS/MS, but in this case, it could not be confirmed by ^1^H-NMR, and this could indicate that this common *Fusarium* metabolite is most likely present but in quantities below the NMR detection limit.

**FIGURE 4:**
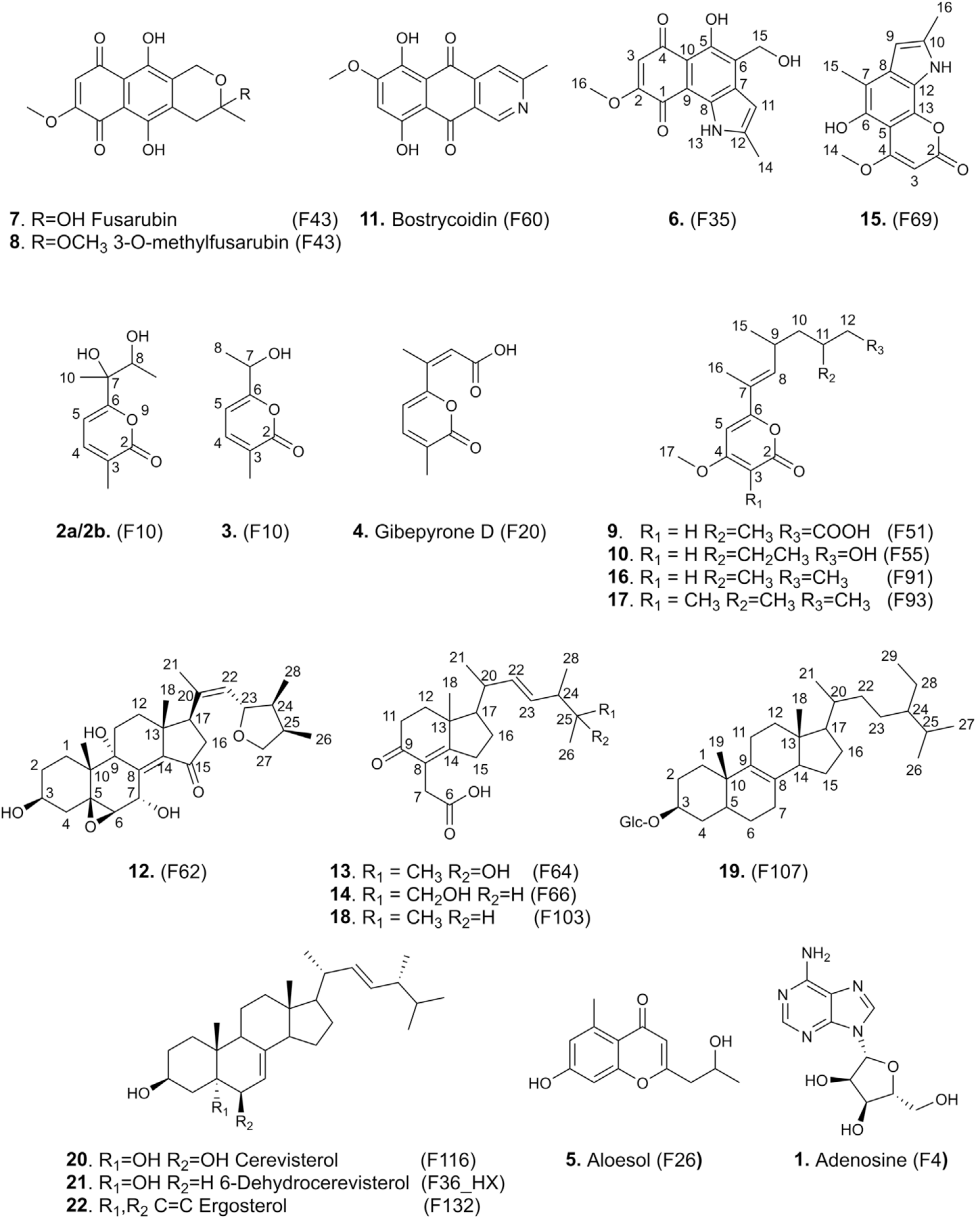
Structures of the isolated compounds **1**–**22**.

In the intermediate region of the chromatogram (fractions F28–F109), three known compounds which are common to *Fusarium* were dereplicated by HRMS/MS and confirmed by ^1^H-NMR as bostrycoidin (**11**) (Arsenault, 1965) in F60 and a mixture of fusarubin (**7**) and 3-O-methylfusarubin (**8**) (Tatum and Baker, 1983) in F43 and F44. Gibepyrone A (Westphal 2018) was dereplicated by HRMS/MS and confirmed by some of its characteristic ^1^H-NMR signals in fraction F42; this compound was, however, present in very low concentrations. In addition, minor metabolites such as dihydroanhydrojavanicin (Tatum et al., 1989) and solaniol (Niu et al., 2019) were dereplicated by HRMS/MS but could not be verified by NMR (Table 2). In this medium-polarity region, several fractions (F51–F56 and F91–F93) seem to have ^1^H signals in common, particularly in the regions between δ_H_ 5.5 and 5.7 and between δ_H_ 6.0 and 6.3 (Figure 3A, highlighted in green), indicating that these fractions may contain structures with close skeletons (**9**, **10**, **16**, and **17**). Another class of molecules (cyclohexanones) could be highlighted in fractions F64–F65 and F103–F104, which were characterized by their signals between δ_H_ 5.2 and 5.3 (orange). The analysis of NMR spectra of these fractions, for which no confident HR-MS/MS annotations were obtained, revealed three new compounds (**13**, **14**, and **18**), as shown in Figure 4.

Since all NMR spectra were recorded in DMSO-*d*_6_, the low field region (δ_H_ 11.0–14.0) of the pseudo-LC-NMR 2D plot highlighted characteristic deshielded mobile protons. Among them, the acidic protons of fatty acids were clearly seen (δ_H_ 12.0) in F113–F119, and quinonic–phenolic groups of fusarubin (**7**) and 3-O-methylfusarubin (**8**) were observed (Figure 1F). Several similar types of signals were linked to the unknown metabolites in F35 and F69.

These selected examples show a good complementarity of NMR and HRMS/MS data for the dereplication of main constituents and help highlight new *Fusarium* metabolites. For full *de novo* structure identification, 2D-NMR spectra were recorded for the compounds of interest and those exhibiting a sufficient S/N ratio in the ^1^H-NMR spectra.

### Structure Elucidation of New Compounds

**FIGURE 5:**
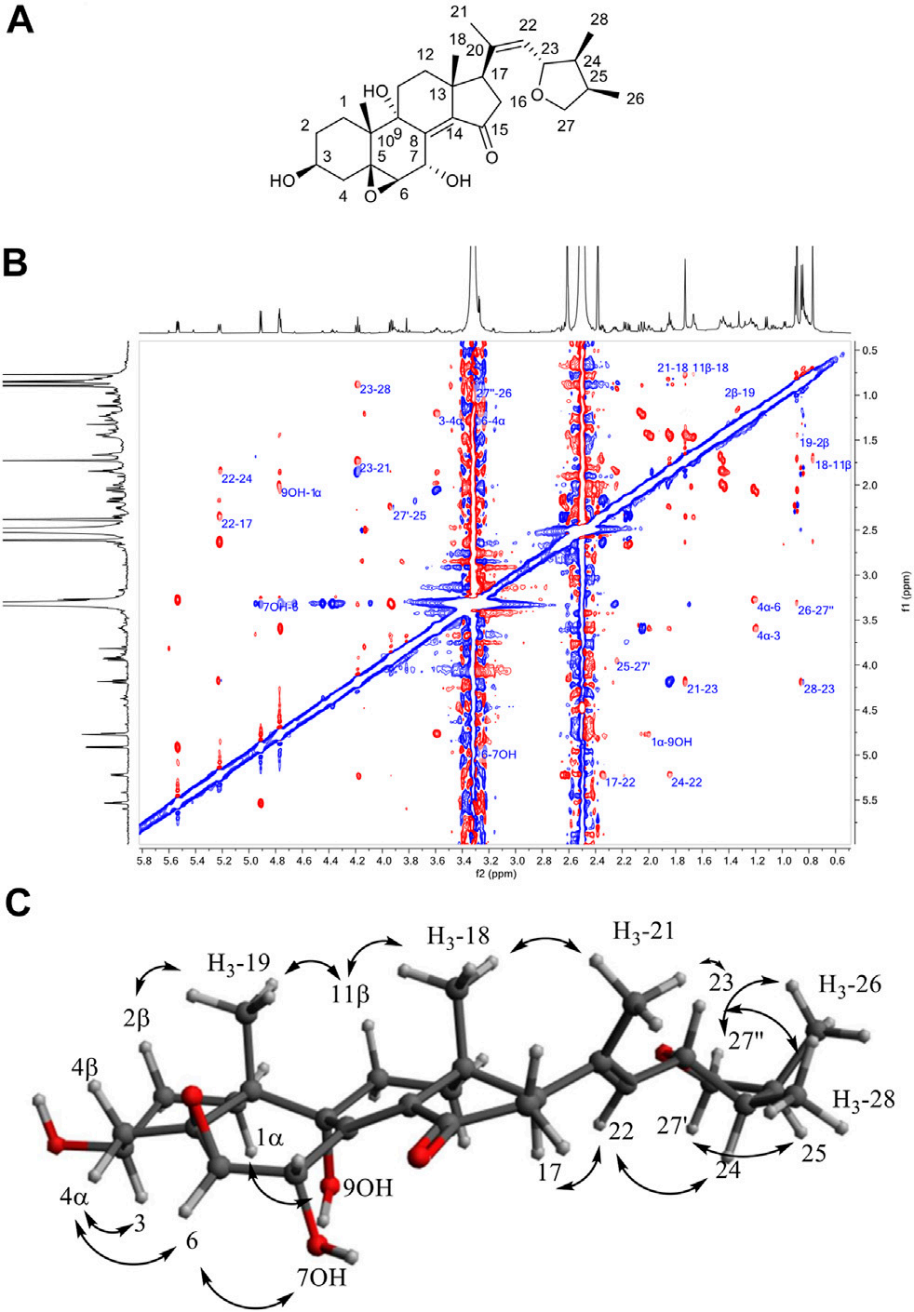
**(A)** Structure of **12**. **(B)** ROESY NMR spectrum of **12** in DMSO-*d*_6_. **(C)** Observed ROESY correlations for compound **12**.

Careful interpretation of the HRMS/MS and ^1^H-NMR data resulted in the identification of 22 compounds in one step (**1**–**22**). Among them, 13 are original NPs described here for the first time (**2a**/**2b**, **3**, **6**, **9**, **10**, **12**, **13**, **14**, **15**, **16**, **17**, **18**, and **19**) and presented in Figure 5. The known dereplicated compounds described above were also confirmed, when necessary, by additional 2D-NMR data and were all previously reported in *Fusarium* species: adenosine (**1**) (Hou et al., 2015), gibepyrone D (**4**) (Wang et al., 2011), aloesol (**5**) (Kashiwada et al., 1984), fusarubin (**7**) and 3-O-methylfusarubin (**8**) (Tatum and Baker, 1983), bostrycoidin (**11**) (Yamamoto et al., 2002), cerevisterol (**20**) (Wang et al., 2011), 6-dehydrocerevisterol (**21**) (Qiao et al., 2017), and ergosterol (**22**) (Thammawong et al., 2011).

Compound **6** (F35) was isolated as a pale rose amorphous solid. The HRMS spectrum showed a molecular ion at *m/z* 288.0846 [M + H]^+^ (calculated for C_15_H_14_NO_5_, 288.0866). No valid annotations based on MS/MS could be obtained for this compound among all described Nectriaceae metabolites. The ^1^H and edited-HSQC NMR spectra showed three exchangeable protons at δ_H_ 5.06 (1H, t, *J* = 5.6 Hz, OH-15), 11.39 (1H, s, NH-13), and 12.88 (1H, s, OH-5), two aromatic protons at δ_H_ 6.23 (1H, s, H-3) and 6.44 (1H, dd, *J* = 2.1, 1.1 Hz, H-11), an oxymethylene at δ_H_ 4.75 (2H, d, *J* = 5.5 Hz, H_2_-15), a methoxy group at δ_H_ 3.89 (3H, s, H_3_-16), and a methyl at δ_H_ 2.44 (3H, d, *J* = 1.0 Hz, H_3_-14). A methoxy naphthoquinone moiety such as that found in fusarubin (**7**) was identified, thanks to the HMBC correlations from H-3 to the carbonyl C-4 (δ_C_ 191.0) and C-1 (δ_C_ 179.5) to the quaternary carbons C-2 (δ_C_ 160.5) and C-10 (δ_C_ 107.4) and from the methoxy group to C-2 (Table 3). The methoxy signals were clearly shared in the pseudo-LC-NMR plot between compounds **6** (F35) and **15** (F69) as well as the two furarubins, **7** and **8** (F43-F44). This was also clearly visible for the common H-3 aromatic signal of these molecules (Figure 4). The HMBC correlations from the deshielded hydroxyl OH-5 to C-10, C-5 (δ_C_ 152.1), and C-6 (δ_C_ 126.8) and from the oxymethylene H_2_-15 to C-5, C-6, and C-7 allowed us to position these two groups. In addition, a 2-methyl-pyrrole group was placed in C7–C8 according to the HMBC correlations from the aromatic proton H-11 to C-6, C-7, and C-8 (δ_C_ 130.5), from the methyl protons H_3_-14 to C-11 (δ_C_ 99.8) and C-12 (δ_C_ 156.7), and from NH-13 to C-7, C-8, C-11, and C-12. The ROESY correlations from H-3 to the methoxy H_3_-16 and from H-11 to H_2_-15 and H_3_-14 confirmed the structure. Compound **6** was thus identified as 5-hydroxy-4-(hydroxymethyl)-8-methoxy-2-methyl-1*H*-benzo[g]indole-6,9-dione.

**TABLE 3:** NMR chemical shifts of compound **6** and **15** in DMSO-*d*_6_ at 600 MHz.

N°	Compound 6				Compound 15	
	δ_H_ (multiplicity, *J*, nH)	δ_C_	HMBC	ROESY	δ_H_	δ_C_
1	-	179.5		-	-	179.2
2	-	160.5		-	-	160.5
3	6.23 (s, 1H)	109.0	C-2, C-1, C-10, C-4	H-16	6.21 (s, 1H)	108.8
4	-	191.0		-	-	-
5	-	152.1		-	-	152.2
6	-	126.8		-	-	123.9
7	-	137.5		-	-	137.6
8	-	130.5		-	-	129.8
9	-	-		-		
10	-	107.4		-	-	107.3
11	6.44 (dd, 2.1, 1.1 Hz, 1H)	99.8	C-8, C-7, C-12	H-15, OH-5	6.28 (s, 1H)	98.9
12	-	146.7		-	-	146.2
NH-13	11.39 (s, 1H)	-	C-8, C-7, C-12, C-11	H-14	11.39 (s, 1H)	-
14	2.44 (d, 1.0 Hz, 3H)	13.8	C-12, C-11	H-13	2.44 (s, 3H)	13.7
15	4.75 (d, 5.5 Hz, 2H)	55.1	C-7, C-6, C-5	H-11, OH-15	2.36 (s, 3H)	12.0
16	3.89 (s, 3H)	56.6	C-2	H-3	3.89 (s, 3H)	56.5
OH-5	12.88 (s, 1H)	-	C-10, C-6, C-5	-	12.84 (s, 1H)	-
OH-15	5.06 (d, 5.5 Hz, 3H)	-	C-6, C-15	H-11, H-15		

Compound **15** (F69) showed a molecular ion at *m/z* 272.0923 [M+H]^+^ and a molecular formula of C_15_H_13_NO_4_, which actually correspond to one oxygen less than the one of **6**. The NMR data of **15** also showed close similarities to those of **6** (Table 3), except that the oxymethylene H_2_-15 was replaced by a methyl, H_3_-15, at δ_H_ 2.36. This compound was thus identified as 5-hydroxy-8-methoxy-2,4-dimethyl-1*H*-benzo[g]indole-6,9-dione (Figure 4).

The HRMS spectrum of **9** (F51) displayed a molecular ion at *m/z* 295.1542 [M + H]^+^, corresponding to the molecular formula C_16_H_22_O_5_. A 4-methoxy-6-substituted-α-pyrone was identified based on the HMBC correlations from the aromatic proton H-5 to C-3, C-6, and C-7, from H-3 to C-5, C-4, and C-2, and from the methoxy signal H_3_-17 to C-4 (Table 4). An alkyl side chain was attached in C-7; the HMBC correlations from H_3_-16 to C-6, C-7, and C-8, from H_3_-15 to C-8, C-9, and C-10, from H_3_-14 to C-10, C-11, and C-12, and from H_2_-12 to C13 allowed us to unambiguously assign this 3,5,7-trimethylhept-6-enoic acid side chain. The *E* configuration of the double bond of the side chain was deduced from the ROE correlation between H_3_-16 and H-9. As discussed previously on the pseudo-LC-NMR plot, some of the ^1^H-NMR signals of **9** were common to other metabolites (**10**, **16**, and **17**) found in F55, F91, and F93, suggesting a common pyrone moiety (Figure 4). Compound **9** was identified as (6*E*)-7-(4-methoxy-6-oxo-6H-pyran-2-yl)-3,5-dimethyloct-6-enoic acid.

**TABLE 4:** NMR chemical shifts of compound **9**, **10**, **16,** and **17** in DMSO-*d*_6_ at 600 MHz.

N°	Compound 9				Compound 10		Compound 16		Compound 17	
	δ_H_ (multiplicity, *J*, nH)	δ_C_	HMBC	ROESY	δ_H_	δ_C_	δ_H_	δ_C_	δ_H_	δ_C_
2	-	180.2		-	-	180.2			-	179.7
3	5.52 (d, 1.8 Hz, 1H)	88.7	C-5, C-4, C-2	H-17	5.52 (d, 1.8 Hz, 1H)	88.6	5.52 (d, 1.8 Hz, 1H)	88.8	-	98.9
4	-	167.2		-	-	167.2	-	167.4	-	162.0
5	6.11 (d, 1.8 Hz, 1H)	108.7	C-3, C-6, C-7	H-16	6.10 (d, 1.8 Hz, 1H)	108.6	6.10 (d, 1.8 Hz, 1H)	108.9	6.17 (s, 1H)	108.3
6	-	159.6		-	-	159.6	-	159.7	-	158.3
7	-	124.3		-	-	124.1	-	124.2	-	124.4
8	6.15 (dd, 9.8, 1.4 Hz, 1H)	140.0	C-6, C-16, C-9, C-10, C-15	H-10'', H-15	6.14 (dd, 9.9, 1.5 Hz, 1H)	140.3	6.16 (dq, 9.8, 1.2 Hz, 1H)	140.5	6.19 (d, 9.4 Hz, 1H)	140.1
9	2.67 (m, 1H)	29.9	C-7, C-8, C-10, C-15	H-16, H-12', H-14	2.69 (m, 1H)	29.9	2.66 (m, 1H)	30.1	2.66 (m, 1H)	30.1
10	1.34 (dt, 3.4, 6.8 Hz, 1H)	43.3	C-8, C-9, C-15, C-11, C-12, C-14	-	1.35 (m, 1H)	37.8	1.32 (m, 1H)	43.5	-	43.5
	1.21 (dt, 13.4, 7.4 Hz, 1H)		C-8, C-9, C-15, C-11, C-12, C-14	H-8	1.22 (m, 1H)		1.14 (m, 1H)			
11	1.79 (dqd, 8.5, 6.8, 5.1 Hz, 1H)	27.6	C-10, C-12, C-14	-	1.23 (m, 1H)	39.1	1.32 (m, 1H)	31.5	1.34 (m, 1H)	31.4
12	2.25 (m, 1H)	40.9	C-10, C-11, C-13, C-14	H-9	1.27 (m, 2H)	22.6	1.32 (m, 1H)	28.6	1.34 (m, 1H)	28.7
	1.96 (dd, 15.0, 8.5 Hz, 1H)		C-10, C-11, C-13, C-14	-			1.08 (m, 1H)		1.24 (m, 1H)	
13	11.99 (s, 1H)	173.7		-	0.81 (d, 7.3 Hz, 3H)	10.5	0.82 (t, 7.4 Hz, 3H)	11.0	0.83 (d, 7.4 Hz, 3H)	11.1
14	0.89 (d, 6.6 Hz, 3H)	19.8	C-10, C-11, C-12	H-9	3.30 (dt, 10.2, 5.0 Hz, 1H)	62.9	0.84 (d, 6.3 Hz, 3H)	19.3	0.85 (d, 6.1 Hz, 3H)	19.3
					3.25 (dt, 10.2, 5.0 Hz, 1H)					
15	0.96 (d, 6.6 Hz, 3H)	19.9	C-8, C-9, C-10	H-8	0.98 (d, 6.6 Hz, 3H)	20.4	0.96 (d, 6.5 Hz, 3H)	20.1	0.98 (d, 6.7 Hz, 3H)	20.1
16	1.86c	12.3	C-6, C-7, C-8	H-5, H-9	1.86 (d, 1.3 Hz, 3H)	12.1	1.86 (d, 1.2 Hz, 3H)	12.3	1.88 (d, 1.3 Hz, 3H)	12.3
17	3.88 (s, 3H)	56.4	C-4	H-3	3.88 (s, 3H)	56.3	3.88 (s, 3H)	56.5	4.02 (s, 3H)	56.0
OH-14					4.34 (t, 5.2 Hz, 1H)				1.69 (s, 3H)	6.5
18										

The NMR data of **10** (F55) indeed exhibited close similarities to those of **9** (Table 4); they share the same 4-methoxy-α-pyrone and the difference lies in the side chain. One of the methyl doublet signals present in **9** was replaced in **10** by a methyl triplet at δ_H_ 0.81 (3H, t, *J* = 7.3 Hz, H_3_-13). An additional oxymethylene was observed at δ_H_ 3.25 (1H, dt, *J* = 10.2, 5.0 Hz, H-14'') and 3.30 (1H, dt, *J* = 10.2, 5.0 Hz, H-14') in addition to a hydroxyl at δ_H_ 4.34 (1H, t, *J* = 5.2 Hz, OH-14), whereas the carbonyl of the acid group was no longer observed. The 2D-NMR experiments were in good agreement with the structure presented in Figure 4, and the ion at *m/z* 281.1744 [M + H]^+^ confirmed the structure as 6-((*E*)-6-ethyl-7-hydroxy-4-methylhept-2-en-2-yl)-4-methoxy-2H-pyran-2-one.

Compound **16**, which has a molecular formula of C_16_H_24_O_3_ and ionized at *m/z* 265.1816 [M + H]^+^ in F91, shares the same skeleton as **9** and **10**. The carboxyl group presented at the end of the side chain of **9** was replaced in this case by a methyl group at δ_H_ 0.82 (3H, t, *J* = 7.4 Hz, H_3_-13) (Table 4). Compound **16** was thus identified as 4-methoxy-6-((*E*)-4,6-dimethyloct-2-en-2-yl)-2H-pyran-2-one.

Compound **17** (C_17_H_26_O_3_) which was ionized at *m/z* 279.1956 [M + H]^+^ in F93 also belongs to the same family. The side chain was the same as that of **16**, but an additional methyl group was observed in C-3 at δ_H_ 1.69 (3H, s, H_3_-18). The HMBC correlations from the methyl H_3_-18 to the ester carbon C-2 (δ_C_ 179.7), the olefinic carbon C-3 (δ_C_ 98.9), and the oxygenated olefinic carbon C-4 (δ_C_ 162.0) confirmed this structure as 4-methoxy-3-methyl-6-((*E*)-4,6-dimethyloct-2-en-2-yl)-2H-pyran-2-one (Table 4). A zoom-in on the stacked plot also clearly highlights this similarity on the side chain (Figure 3D).

As discussed above, compounds **13**, **14**, and **18**, which shared many common ^1^H-NMR signals in the pseudo-LC-NMR plot, were assigned to the same structural type. Among them, **18** exhibited the most intense signals and was analyzed in depth first.

Compound **18** in F103 was isolated as a pale yellow amorphous solid. The HRMS spectrum showed a protonated ion at *m/z* 333.2441 corresponding to a molecular formula of C_21_H_32_O_3_. Its NMR data are summarized in Table 5. The HMBC correlations from methyl H_3_-18 to the methylene C-12, the quaternary carbon C-13, the *sp* ^2^ carbon C-14, and the methine C-17, from the methylene H_2_-7 to C-14, the *sp* ^2^ carbon C-8, the carbonyl C-9, and to the carboxyl C-6, from the methylene H_2_-11 to C-6, and from H_2_-15 to C-14 and C-17 in combination with COSY correlations from H_2_-11 to H_2_-12 and from H-17 to H_2_-16 allowed us to identify fused six- and five-membered rings. The side chain in C-17 was identified and positioned, thanks to the HMBC correlation from the methyl H_3_-21 to the methines C-17 and C-20 and the olefinic carbon C-22, from the methyl H_3_-28 to the olefinic carbon C-23 and the methines C-24 and C-25, and from the methyls H_3_-26 and H_3_-27 to the methines C-24 and C-25 (Table 5). This compound corresponds to a highly degraded ergostane-type steroid identified as 2-(2,3,5,6,7,7a-hexahydro-7a-methyl-1-((*E*)-5,6-dimethylhept-3-en-2-yl)-5-oxo-1H-inden-4-yl)acetic acid.

**TABLE 5:** NMR chemical shifts of compound **18**, **13,** and **14** in DMSO-*d*_6_ at 600 MHz.

N°	Compound 18				Compound 13		Compound 14	
	δ_H_ (multiplicity, *J*, nH)	δ_C_	HMBC	ROESY	δ_H_	δ_C_	δ_H_	δ_C_
6	-	171.7		-	-	171.7	-	171.8
7	3.02 (d, 16.5 Hz, 1H)	30.9	C-9, C-8, C-14, C-6	-	3.02 (d, 16.5 Hz, 1H)	30.8	3.02 (d, 16.4 Hz, 1H)	30.9
	2.98 (d, 16.5 Hz, 1H)				2.99 (d, 16.5 Hz, 1H)		2.98 (d, 16.4 Hz, 1H)	
8	-	125.0		-				
9	-	196.3		-				
11	2.56 (m, 1H)	32.7	C-9	H-18	2.22 (m, 1H)	32.7	2.53 (overlapped, 1H)	32.7
	2.22 (m, 1H)			-	2.19 (m, 1H)		2.22 (dd, 18.7, 4.8 Hz, 1H)	
12	2.15 (m, 1H)	35.8	-	-	2.16 (m, 1H)	35.8	2.17 (m, 1H)	35.9
	1.79 (m, 1H)				1.79 (m, 1H)		1.78 (m, 1H)	
13	-	44.6		-	-	44.7	-	44.6
14	-	175.2		-	-	175.2	-	175.2
15	2.46 (m, 1H)	27.3	C-14, C-17	-				
	2.34 (m, 1H)			H-7''				
16	1.78 (m, 1H)	27.2	-	-	1.78 (m, 1H)	27.0	1.78 (m, 1H)	27.3
	1.51 (m, 1H)			H-18	1.51 (m, 1H)		1.51 (p, 11.4 Hz, 1H)	
17	1.42 (m, 1H)	55.4	-	H-21	1.42 (m, 1H)	55.5	1.43 (m, 1H)	55.5
18	1.06 (s, 3H)	16.3	C-14, C-13, C-12, C-17	H-11', H-16'', H-20	1.06 (s, 3H)	16.2	1.06 (s, 3H)	16.3
20	2.18 (m, 1H)	38.1	C-17, C-22, C-23	H-18	2.17 (m, 1H)	38.1	2.17 (m, 1H)	38.2
21	1.03 (d, 6.6 Hz, 3H)	20.9	C-17, C-20, C-22	H-17	1.03 (d, 7.1 Hz, 3H)	20.8	1.03 (d, 6.7 Hz, 3H)	21.0
22	5.25 (dd, 15.2, 7.9 Hz, 1H)	134.7	C-24	-	5.26 (dd, 15.3, 8.7 Hz, 1H)	135.0	5.27 (m, 1H)	135.0
23	5.29 (dd, 15.2, 7.0 Hz, 1H)	132.0	C-20, C-28	-	5.41 (dd, 15.3, 7.0 Hz, 1H)	131.1	5.27 (m, 1H)	130.9
24	1.88 (m, 1H)	42.0	C-22, C-23, C-25, C-28	-	2.01 (p, 7.0 Hz, 1H)	47.1	2.17 (m, 1H)	37.0
25	1.48 (m, 1H)	32.4	C-24	-	-	70.5	1.43 (m, 1H)	40.6
26	0.83 (d, 6.9 Hz, 3H)	19.7	C-24, C-25, C-27	-	1.03 (s, 3H)	28.2	0.75 (d, 6.9 Hz, 3H)	13.1
27	0.81 (d, 6.9 Hz, 3H)	19.4	C-24, C-25, C-26	-	0.99 (s, 3H)	26.0	3.16 (d, 6.4 Hz, 1H)	64.4
							3.17 (d, 6.4 Hz, 1H)	
28	0.91 (d, 6.7 Hz, 3H)	17.3	C-23, C-24, C-25	-	0.92 (d, 7.0 Hz, 3H)	14.9	0.94 (d, 7.0 Hz, 3H)	18.3

The NMR signals of the isomeric **13** and **14**, both possessing an MF of C_21_H_32_O_4_ and protonated ions at *m/z* 349.2349, showed great similarities to those of **18** in F103 (C_21_H_32_O_3_), except for the terminal part of the side chain. Compound **13** contains an additional hydroxy group in C-25 as evidenced by the HMBC correlations from the methyls H_3_-26 and H_3_-27 (δ_H_ 1.03 and 0.99, respectively) to the oxygenated quaternary carbon C-25 (δ_C_ 70.5) and the methine C-24 (δ_C_ 47.1). In compound **14**, the terminal methyl H_3_-27 was hydroxylated and replaced by an oxygenated methylene at δ_H_/δ_C_ 3.17/64.4.

Compound **12** in F62 was found to possess an MF of C_28_H_40_O_6_ with 8 degrees of unsaturation, as evidenced by HRMS at *m/z* 473.2877 [M+H]^+^ (calculated for C_28_H_41_O_6_). The ^1^H-NMR data of **12** showed typical signals of an oxygenated steroid: 2 methyl singlets at δ_H_ 0.77 (3H, s, H_3_-18) and 0.89 (3H, s, H_3_-19), three methyl doublets at δ_H_ 0.85 (3H, d, *J* = 7.0 Hz, H_3_-28), 0.90 (3H, d, *J* = 6.8 Hz, H_3_-26), and 1.73 (3H, d, *J* = 1.2 Hz, H_3_-21), four oxygenated methines at δ_H_ 3.27 (1H, d, *J* = 3.7 Hz, H-6), 3.59 (1H, tq, *J* = 10.6, 5.0 Hz, H-3), 4.18 (1H, t, *J* = 8.5 Hz, H-23), and 5.53 (1H, dd, *J* = 5.2, 3.7 Hz, H-7), an oxygenated methylene at δ_H_ 3.31 (1H, overlapped, H-27'') and 3.93 (1H, dd, *J* = 8.2, 6.4 Hz, H-27'), an ethylenic proton at δ_H_ 5.22 (1H, d, *J* = 8.4 Hz, H-22), and a series of methines and methylenes between δ_H_ 1.20 and 2.63. The HMBC also indicated the presence of two oxygenated quaternary carbons at δ_C_ 68.1 (C-5) and 74.8 (C-9), two quaternary *sp* ^2^ carbons at δ_C_ 134.1 (C-20) and 142.8 (C-14), and a carbonyl at δ_C_ 206.7 (C-15). HMBC correlations from H_3_-19 to C-1 (δ_C_ 25.6), C-5, C-9, and C-10 (δ_C_ 37.0), from H_3_-18 to C-12 (δ_C_ 32.1), C-13 (δ_C_ 43.1), C-14, and C-17 (δ_C_ 51.5), and from the methylene H_2_-16 at δ_H_ 2.16 (1H, dd, *J* = 18.3, 7.3 Hz, H-16α) and 2.63 (1H, m, H-16β) to C-15 allowed us to identify the four member rings of sterol in compound **12**, which was identical to the 5β,6β-epoxy-3β,7α,9α-trihydroxy-(22E,24R)ergosta-8(14),22-dien-15-one previously isolated by Wang et al. (2012) from the culture of the Basidiomycete *Polyporus ellisii*. The chain attached to C-17 was found to be new; its linkage was established by the HMBC correlations from the methyl H_3_-21 to C-17 (δ_C_ 51.5), C-20, and C-22 (δ_C_ 128.1) and, thus, the presence of a double bond between C-20 and C-22. The HMBC correlations from H_3_-28 to C-23 (δ_C_ 79.7), C-24 (δ_C_ 42.9), and C-25 (δ_C_ 35.9), from H_3_-26 to C-24, C-25, and C-27 (δ_C_ 73.7), and from H_2_-27 to C-23 indicated the formation of a furan ring. The ROESY correlations from H_3_-18 to H_3_-21 and H-11β, from H_3_-19 to H-11β and H-2β, from H-4α to H-3 and H-6, from OH-7 to H-6, from H-1α to OH-9, from H-23 to H_3_-21 and H_3_-28, and from H-22 to H-17 and H-24 allowed us to determine the relative configuration of this new sterol (Figure 5). Compound (**12**) was characterized as 5β,6β-23,26-diepoxy-3β,7α,9α-trihydroxy-(20Z,23S,24S,25R) ergosta-8(14),20-dien-15-one.

Compound **19** in F107 showed a sodium adduct ion at *m/z* 599.4380 [M+Na]^+^ which correlated with C_35_H_60_O_6_. The NMR spectra of **19** indicated the presence of stigmast-8-en-3-ol with typical signals like two methyl singlets at δ_H_ 0.65 (3H, s, H_3_-18) and 0.96 (3H, s, H_3_-19), three methyl doublets at δ_H_ 0.79 (3H, d, *J* = 6.9 Hz, H_3_-27), 0.82 (3H, d, *J* = 6.9 Hz, H_3_-26), and 0.90 (3H, d, *J* = 6.5 Hz, H_3_-21), one methyl triplet at δ_H_ 0.82 (3H, t, *J* = 7.3 Hz, H_3_-29), one oximethine at δ_H_ 3.46 (1H, tt, *J* = 11.2, 4.3 Hz, H-3), and one olefinic carbon detected on the HMBC spectrum from the correlations of methyl H-18 with C-9 (δ_C_ 140.4). Additional signals corresponding to a glucose unit were detected at δ_H_ 2.89 (1H, td, *J* = 8.4, 4.8 Hz, H-2'), 3.01 (1H, td, *J* = 9.2, 5.0 Hz, H-4'), 3.06 (1H, m, H-5'), 3.12 (1H, td, *J* = 8.9, 4.8 Hz, H-3'), 3.40 (1H, m, H-6'b), 3.64 (1H, dd, *J* = 11.1, 6.2 Hz, H-6'a), and 4.22 (1H, d, *J* = 7.8 Hz, H-1'). Due to the very small amount of isolated compound and the presence of an overlapping fatty acid in the fraction, it was not possible to obtain a complete assignment of the molecule. However, the ROESY correlation between the H-1' proton of glucose and the H-3 proton of the stigmasterol skeleton allowed the positioning of glucose in C-3 and the identification of **19** as 3-*O*-β-D-glucopyranoside-stigmast-8-en-3-ol.

In addition to the compounds described in the polar part of the chromatogram (F1-27), F10 exhibited a ^1^H-NMR spectrum of possibly two to three metabolites. The HRMS data confirmed the presence of two ions at *m/z* 199.0965 and 155.0703 [M + H]^+^ which are typical for C_10_H_15_O_4_ and C_8_H_11_O_3_, respectively.

A detailed 2D-NMR analysis of the fraction revealed the presence of three compounds which share a 3-methyl-pyran-2-one moiety similar to the one of **4**, as indicated by the aromatic H-4 and H-5 at δ_H_/δ_C_ 7.35–7.36/140.4–140.5 and 6.26–6.33/100.4–101.4, respectively, and the methyl at δ_H_/δ_C_ 1.95–1.96/16.0–16.2. Compounds **2a** and **2b** (C_10_H_15_O_4_) were diastereoisomers with a 2,3-dihydroxybutan-2-yl side chain characterized by a methyl doublet (*J* = 6.3 Hz, H_3_-9) at δ_H_ 0.97 and 1.03, an oxygenated methine (m, H-8) at δ_H_ 3.73 and 3.74, and a methyl singlet (H_3_-10) at δ_H_ 1.37 and 1.24 for **2a** and **2b**, respectively. The hydroxylation in C-7 and the linkage of the 2,3-dihydroxybutyl chain in C-6 were confirmed by the HMBC correlations from the methyl H_3_-10 to C-6 (δ_C_ 167.0 and 167.2 for **2a** and **2b**, respectively), C-7 (δ_C_ 74.5 and 75.0 for **2a** and **2b**, respectively), and C-8 (δ_C_ 70.2 and 70.4 for **2a** and **2b**, respectively), and from the aromatic protons H-4 and H-5 to C-6. On the other hand, the 3-methyl-pyran-2-one of **3** (C_8_H_11_O_3_) was substituted by a hydroxyethyl group in C-6 as indicated by the methyl doublet at δ_H_ 1.30 (3H, d, *J* = 6.6 Hz, H_3_-8), the methine at δ_H_ 4.41 (1H, q, *J* = 6.6 Hz, H-7), and the HMBC correlation from the methyl to C-6 (δ_C_ 165.6) and C-7 (δ_C_ 64.9).

Overall, the combination of the LC-HRMS/MS data and the pseudo-LC-NMR plot together with in-depth 2D-NMR analysis of selected peaks provided a good overview of all the main constituents of fractions F1–F135 in a single semi-preparative HPLC separation.

### Determination of Bioactive Zones

Our workflow permitted us to identify all main compounds from the crude extract in one step. However, as many of the fractions were in very small quantities (in the sub-mg range), we had to find a strategy to collect enough amounts of compounds for the bioassays. This was indeed a limitation since the bioassays could only be conducted with at least 500 µg of pure compound.

**FIGURE 6:**
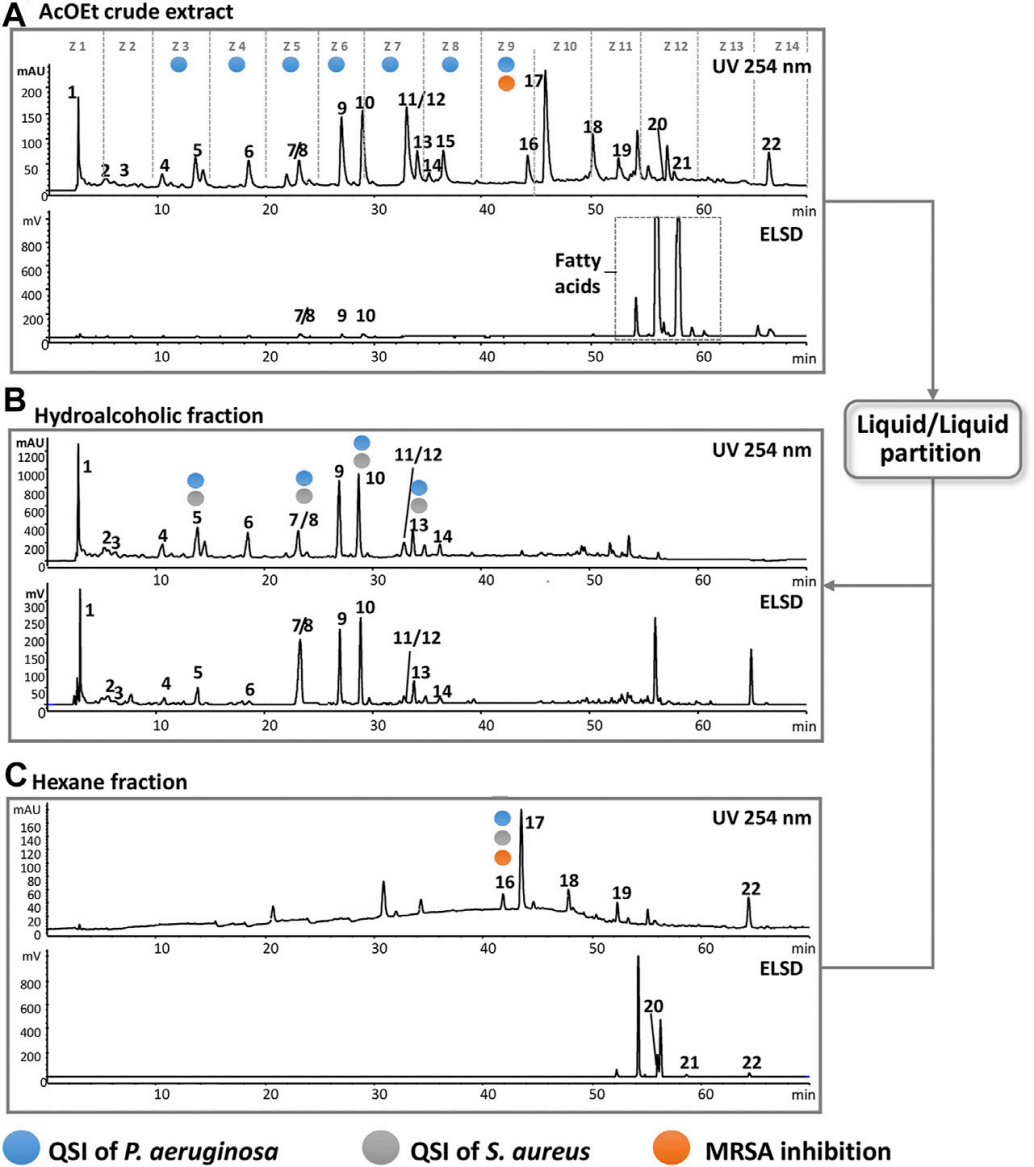
HPLC-DAD-ELSD analysis highlighting the effect of the extract enrichment and the location of various bioactivities monitoring. **(A)** Chromatogram of the crude extract; dashed lines represent the 14 chromatographic zones. Highlighted zones represent the part of the chromatogram for possible bioactivity. The square in the ELSD highlights the presence of fatty acids. **(B)** Chromatogram of the hydroalcoholic part. **(C)** Chromatogram of the hexane. Compounds that possess bioactivity are annotated by colored circles, and the process demonstrates that they can be precisely localized after enrichment.

To ensure the accumulation of enough material for the bioassays, the 135 collected fractions were pooled into 14 chromatographic zones (Z1–Z14), where each zone represents a 5-min window of elution time (Figure 6A). Submitting of the pooled fractions to bioactivity tests gives an approximate location of the active compounds and facilitates targeting them in an additional chromatographic separation. Since the extract was active against a methicillin-resistant *Staphylococcus aureus* (MRSA), the 14 zones were first subjected to MIC tests against this strain. As a result, only zone 9 (Z9) showed significant inhibitory effect at 32 μg/ml, which suggested the presence of an antibacterial compound in fractions F81–F91. In parallel, the 14 zones were evaluated for anti-QS of PA in the same way as for the crude extract and on the same reporter genes (*pqsA* and *lasB*).

For this purpose, we considered zones with bioactive candidates, those that have shown values of 70% or less in the fluorescence level for at least one of the reporters. The value of 70% was chosen since each zone is still a mixture of several constituents and to avoid missing pure bioactive candidates. As a result, seven zones from Z3–Z9, which represent the mid-polarity region, presented activity as QS inhibitors. Z5 was the most active one and significantly reduced the fluorescence level of the gene *pqsA* to 31% and that of *lasB* to 43% (Table 6). In order to determine the specific molecules responsible for these activities, an additional LC-peak targeted chromatographic separation at the semi-preparative level was performed. Thus, an enrichment step was designed to remove the very lipophilic compounds which clearly were not responsible for bioactivity, as shown when the chromatographic zones of the crude extracts were tested.

**TABLE 6:** Minimum inhibitory concentration (MIC) and quorum sensing inhibition (QS) assays of zones of fractions and their corresponding compounds compared to several positive controls.

Zone	% PA (*pqsA*)	% PA (*lasB*)	Compound	% PA (*pqsA*)	% PA (*lasB*)	% SA (*lacZ*)
Control	32 ± 0.2	39 ± 2.9	NA	32 ± 0.2	39 ± 2.9	27 ± 1.6
Z_3	68 ± 0.0	85 ± 0.2	(**4**)	60 ± 4.5	76 ± 8.0	79 ± 0.7
			(**5**)	40 ± 8.5	51 ± 7.7	**11** ± 5.0
Z_4	48 ± 2.6	65 ± 1.2	(**6**)	59 ± 2.7	69 ± 0.9	37 ± 0.0
Z_5	31 ± 1.6	43 ± 0.4	(**7/8**)	**12** ± 0.6	**15** ± 1.0	**3** ± 0.5^a^
Z_6	45 ± 0.7	60 ± 0.9	(**9**)	58 ± 3.6	68 ± 2.7	90 ± 0.0
			(**10**)	36 ± 2.2	41 ± 0.4	**17** ± 4.2
Z_7	60 ± 4.3	75 ± 0.4	(**11**)	66 ± 10.1	71 ± 0.5	121 ± 22.0
			(**12**)	58 ± 3.6	68 ± 2.7	79 ± 0.7
			(**13**)	34 ± 2.6	**39** ± 0.4	**13** ± 0.2
Z_8	64 ± 0.3	82 ± 1.8	(1**4**)	72 ± 6.5	73 ± 0.2	31 ± 1.2
			(**15**)	57 ± 2.9	63 ± 0.1	36 ± 3.3
Z_9 (MIC MRSA at 32 μg/ml)	63 ± 0.6	82 ± 0.0	(**16**) (MIC MRSA at 32 μg/ml)	37 ± 2.1	39 ± 6.9	**15** ± 11.0

Values show the mean of triplicates ± SD. Values in **bold** are lower than the corresponding control. Positive controls of QS assays are azithromycin at 2 μg/ml for PA and S. caprae AIP 1 µM for SA. Results were compared to DMSO fixed at 100%. MRSA, methicillin-resistant Staphylococcus aureus; PA, Pseudomonas aeruginosa; NA, not applicable.

a Fluorescence was biased due to the natural coloration of the compound.

### Enrichment of the Crude Extract for Targeted Purification of Bioactive Compounds

In order to increase the concentration of the bioactive compounds in Z3–Z9, the ethyl acetate crude extract was submitted to liquid–liquid separation using water/methanol in a ratio of 7:3 and hexane. This yielded approximately 70 mg of the hydroalcoholic fraction and 90 mg of the hexane fraction from 160 mg of crude extract. As seen in Figures 6B,C, all zones of interest (Z3–Z9) are retained in the hydroalcoholic part except Z9, and the ELSD traces highlight well the efficiency of the enrichment procedure.

Taking into account the enrichment factor (2.3 folds), a single semi-preparative HPLC fractionation was carried out on 30 mg of the hydroalcoholic fraction under the same conditions as for the crude extract (Supplementary Figure S4). This yielded a good baseline separation in most compounds that ease the peak targeted collection. Purity of collected fractions was checked by ^1^H-NMR and LC-ELSD-MS (data not shown) and enabled the bioactivity assessment of compounds **1**–**14**. Since Z9 was also a bioactive target, the apolar hexane fraction was purified similarly. Interestingly, by comparing the dry weights of collected fractions from enriched extract with the equivalent ones from crude extract, all compounds were collected in amounts higher than 500 µg, which was the threshold for the bioassay we selected. This also granted supplementary amounts to perform further bioassay experiments such as quantitative PCR.

As shown, in our strategy, the precise assignment of the bioactive LC peaks was dependent on the amounts of extract injected. While working with fungal extracts, the pseudo-LC-NMR analysis performed directly at the crude extract level identified all main metabolites and highlighted a high content of fatty acids. Such a profiling based on NMR detection was of interest to provide an unbiased view of the metabolome. However, our fractionation process did not allow a direct biological assessment of all the LC peaks collected, due to lack of assay sensitivity, and necessitated the pooling of fractions. The enrichment procedure followed by the targeted isolation using the same semi-preparative fractionation finally provided a sufficient amount for bioactivity assessments.

### Biological Assay of Pure Compounds

In order to assign compounds which are responsible for the antibacterial and anti-QS activity, purified compounds (**4**–**16**) that belong to Z3–Z9 were submitted to the same biological tests as described before.

**FIGURE 7:**
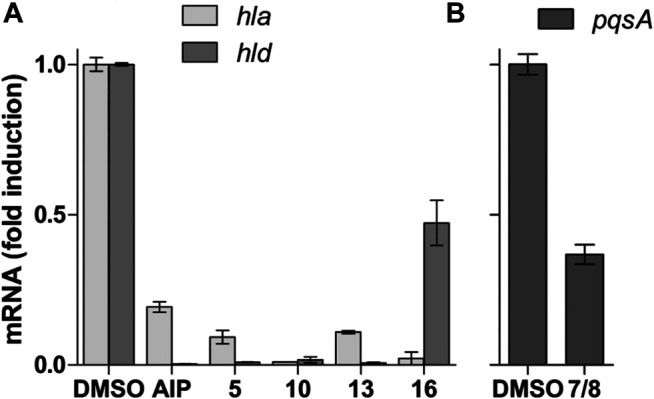
**(A)** Quantitative PCR test of *S. aureus* QS regulated genes *hla* and *hld*. AIP (auto-inducer from *S. caprae*) is used as the positive inhibitor control. **(B)** Quantitative PCR test of *P. aeruginosa* for QS regulated gene *pqsA*. Fold induction is represented relative to DMSO alone (fixed at 1).

For the QS test on *P. aeruginosa*, compounds **5**, **10**, **13,** and **16** presented moderate to weak activity profiles as they did not reach values under 30% in fluorescence reduction at 128 μg/ml (Table 6). However, a mixture of the known *Fusarium* quinones fusarubin (**7**) and 3-O-methylfusarubin (**8**), which previously located in the active Z5, presented an enhanced and significant QS inhibition (12 and 15%) in *pqs* and *las* systems, respectively, at 128 μg/ml. To further confirm these results, we performed quantitative RT-PCR analyses on the QS-regulated genes *pqsA, lasB*, and *rhlA* involved in rhamnolipid production (Van Gennip et al., 2009). The relative expression of the QS-regulated gene *pqsA* in the presence of **7** and **8** was two times less expressed than the reference (Figure 7B). However, relative expression of *lasB* and *rhlA* did not show significant effects (data not shown) unlike results obtained by *gfp* transcriptional fusions (Table 6). In addition, since the zone Z9 was the only one showing MRSA inhibition activity, its main compound is one of the new pyrones. **16** was evaluated against MRSA following the protocol of Wiegand et al. (2008). Compound **16** presented with an MIC at 32 μg/ml (Table 6).

On the whole, a series of the isolated compounds’ anti-QS assay for *S. aureus* on the reporter strain *rnaIII-lacZ* targeting the reporter gene *agr* was also performed following the method of (Nielsen et al., 2010). Compounds **5**, **10**, **13,** and **16** presented a QS *rnaIII-lacZ* inhibition at 32 μg/ml, showing fluorescence values less than 20% (Table 6). These results were confirmed by real-time quantitative reverse transcription (RT-qPCR) in the gene expression of *hla* and *hld*, both coding for QS regulated exotoxins (Figure 7A). Compared to the positive control, the antagonist auto-inducing peptide (AIP) from *Staphylococcus caprae*, all of these compounds show a better inhibition on *hla* gene expression, which codes for the alpha-hemolysin. The tendency is the same for *hld* gene expression, which codes for the delta-hemolysin, except for **16,** which shows a less efficient effect with an inhibition of only two folds (Figure 7A).

Overall, this comprehensive study of *F. petroliphilum* enables an unambiguous characterization of 22 compounds based on one single high-resolution semi-preparative HPLC separation of the crude extract which highlighted 13 compounds that were never reported to our knowledge. This allowed a better characterization of the composition of *F. petroliphilum*, which is a rarely chemically studied member of the *F. solani* species complex. Our genetic investigation of this endophyte also enabled its unambiguous identification and positioned this strain as a member of the *F. solani* species complex.

The pseudo-LC-NMR process correlated well with the dereplication results obtained from the molecular networks. For most of the unannotated metabolites, full *de novo* structure assignment unambiguously identified new fungal NPs. This also provided a valid set of standards which allowed efficient MN annotation propagation. In its current state, however, the proposed workflow still requires partial manual inspection/processing of both NMR and MS data. Future development of algorithms for connecting NMR information into the MN would facilitate the efficiency of a full metabolome composition assessment process. Our study mainly highlights the potential of such data integration and demonstrates that with well-optimized chromatographic conditions at the semi-preparative HPLC level and high-quality spectral data that can be efficiently generated in a restricted laboratory time frame.

On the bioactivity aspects, we were also able to show that the generic fractionation obtained by semi-preparative HPLC allowed a consistent concentration of activity from the broad chromatographic zones to the active ingredients. Several of the identified metabolites exhibited weak-to-moderate MIC values on a gram-positive MRSA and no growth inhibition on the gram-negative *P. aeruginosa*. However, an in-depth study of the QS activity of both strains through our selected assays revealed significant QS inhibition for some of the metabolites, especially for the known fusarubins for which anti-QS activities were never reported. Based on this approach, we plan to further study the different endophytes found in *Posidonia oceanica*, which we identify as an interesting model for the study of the endophytic community.

## Materials and Methods

### Plant Material, Fungal Endophyte Isolation and Identification

*Posidonia oceanica* shoots were collected from the shores of Banyuls-sur-Mer in France at a depth of 5–10 m. Fresh plant parts (leaves, roots, and rhizomes) were cleaned under stream water and then dipped into 70% ethanol for 3 min. Samples of all plant parts were cut into 1-cm^2^ pieces and placed in a culture plate containing potato dextrose agar (PDA). Fungal tips were transferred to a new PDA culture plate as soon as they appeared and were left to grow for 30 days.

Samples of the fungal cultures were sent to Bio2Mar, France (http://bio2mar.obs-banyuls.fr), who performed the amplification and sequencing of the internal transcribed spacers plus the 5.8S (ITS). After the removal of small and large subunit ITS flanking regions, a first identification of this fungal isolate was performed, searching for similarity of that ITS sequence to those deposited in GenBank (National Center for Biotechnology Information, U.S.) (Benson et al., 2018). Sequence similarity search in GenBank (BLAST; https://blast.ncbi.nlm.nih.gov/Blast.cgi) used the “blastn” (Megablast) option excluding “uncultured/environmental sample sequences.”

To identify the selected *Fusarium* strain FEP 16 more precisely, DNA was extracted from a sample of the fungal culture placed in 500 µl of cetyl-trimethyl-ammonium bromide buffer (CTAB 1x). DNA extraction was performed according to Hofstetter et al. (2002). Four more loci were amplified and sequenced: part of the transcription elongation factor 1-alpha (*TEF*1-α) using primers EF1-1F and EF1-1R (Morehouse et al., 2003), part of the RNA polymerase II second largest subunit [*RPB2*] using primers fRPB2-5F and fRPB2-7cR for regions 5–7 and fRPB2-7cF and fRPB2-11aR for regions 7–11 (Liu et al., 1999), part of β-tubulin using primers Bt2a and Bt2b (Glass and Donaldson, 1995), and part of calmodulin with primers CAL-228F and CAL-737R (Carbone and Kohn, 2019). Amplification of these loci used the reagents and conditions of a Taq PCR core kit (Qiagen Inc., Valencia, CA, United States). Sanger sequencing was performed using the amplification primers by Fasteris SA (Life Science Genesupport, Geneva, Switzerland). The obtained sequences were assembled in Sequencher v4.9 (Gene Codes Corp., United States). These sequences were combined with sequences sampled from the study by Bohni et al. (2016). The alignments of sequences were done in MacClade v4.08 (Maddison and Maddison, 1989). Ambiguously aligned regions (mostly spliceosomal introns in protein-coding genes and gap regions in ribosomal loci) were excluded from phylogenetic analyses.

Searches for the most likely tree included three independent runs conducted in PhyML v3.0 (Guindon and Gascuel, 2003), with evolutionary model = GTR and other parameters estimated during the search. Bootstrap values (BS) were inferred based on 500 replicates using the same settings as for the search of the most likely tree. Branch support was considered significant when BS values were ≥70% (Alfaro et al., 2003).

### Cultivation and Extraction of *Fusarium petroliphilum*

For the whole study, the same strain of *F. petroliphilum*, FEP 16, was used. The strain was cultivated in the laboratory using Sabouraud dextrose agar (CM0041; Oxoid). The agar was suspended in artificial sea water (Supplementary Table S3) and then poured over 8.5-cm petri dishes. The incubation was at room temperature for 18 days when the fungus fully dominated the plate (Kour et al., 2007). This setup was applied on both the small scale (10 plates) and the large scale (100 plates) to ensure equal outcomes. The fresh agar was cut into 1-cm squares and directly dipped into ethyl acetate and then agitated overnigh (Orbitron, Infors^®^, Bottmingen, Switzerland, room temperature, at 100 rpm), followed by 20 min of ultrasound sonication, and filtered through Whatman™ No. 1. This process was repeated three times with fresh solvent. The filtrates were gathered, and the solvent was evaporated to dryness using a rotary evaporator (Buchi^®^, Flawil, Switzerland) to yield 300 mg of crude extract.

### General Experimental Procedures

The NMR spectroscopic data were recorded at 298 K on a Bruker Avance III HD 600 MHz. An NMR spectrometer equipped with a QCI 5 mm Cryoprobe and a SampleJet automated sample changer (Bruker BioSpin, Rheinstetten, Germany) was used. Chemical shifts are measured in parts per million (δ) using the residual DMSO-d6 (δH 2.50; δC 39.5) as the internal standard for 1H and 13C, respectively, and coupling constants (J) are reported in Hz. Complete assignments were performed based on 2D-NMR experiments (correlation spectroscopy (COSY), rotational nuclear Overhauser effect spectroscopy (ROESY), heteronuclear single quantum correlation (HSQC), and heteronuclear multiple bond coherence (HMBC)). Optical rotations were measured using a Jasco P-2000 polarimeter (JASCO Corporation, Tokyo, Japan). UV absorbance was measured using a JASCO FT/IR-4100 spectrometer (JASCO Corporation) equipped with a PIKE MIRacle™ (JASCO Corporation).

### UHPLC-DAD-MS-ELSD and UHPLC-HRMS/MS Analyses

UHPLC-DAD-MS-ELSD analyses were conducted on an Acquity UHPLC system (Waters, Milford, MA, United States), equipped with DAD and MS single quadrupole (Acquity QDa) as detectors supplemented with an electrospray ionization source (ESI). DAD detection was set between 190 and 500 nm. The system was controlled using MassLynx^®^ v4.2 (Waters), and the ESI and MS acquisition conditions were set according to the work of (Righi et al., 2020). For the metabolite profiling of the ethyl acetate extract, 3 µl was injected on an Acquity BEH C18 column (100 × 2.1 mm i.d., 1.7 µm; Waters, Milford, MA, United States). The solvent system was H_2_O (A) and MeOH (B), both containing 0.1% formic acid (FA). The separation conditions were optimized by decreasing the slope of the LC linear gradient to ensure the best distribution of the metabolites across the chromatographic window. The gradient was set as follows: 34–100% of B in 16.57 min, followed by 3 min of washing at 100% B. The flow rate was 0.3 ml/min, and the separation was conducted at 40°C.

UHPLC-HRMS/MS analyses was performed on a Thermo Dionex Ultimate 3000 UHPLC system interfaced with a Q Exactive Plus MS (Thermo Scientific, Bremen, Germany) supplemented with a heated electrospray ionization source (HESI-II). Thermo Scientific Xcalibur 3.1 software was used for instrument control. The HESI-II parameters were set according to the work of (Rutz et al., 2019). For the profile of the ethyl acetate extract, 2 µl of the extract was injected using the same column as mentioned above. The mobile phase was H_2_O (A) and MeCN (B), both containing 0.1% FA. The gradient mode was as follows: 5–100% of B in 18 min, followed by 4 min of 100% B. The flow rate was 0.6 ml/min, and the separation was conducted at 25°C. Metabolite profiling of all fractions was conducted on the same system and the same ESI settings; 2 µl was injected through an Acquity BEH C18 column (50 × 2.1 mm i.d., 1.7 µm; Waters, Milford, MA, United States). The mobile phase was H_2_O (A) and MeCN (B), both containing 0.1% FA. The gradient mode was 5–100% of B in 4 min, followed by 2 min of washing and reconditioning.

### Generation of Molecular Networks

Raw spectral data of the extract were analyzed using MZmine 2.53 (Pluskal et al., 2010). The parameters were adjusted as follows: mass detection performed as MS level 1 (noise level at 10^6^) and MS level 2 (noise level at 0). The ADAP chromatogram builder was used with the threshold set to 4 × 10^5^. Chromatogram deconvolution (algorithm ADAP) was set within its default parameters, except for the R_t_ wavelet range, which was set as 0–0.1. The most intense isotopes were kept through the isotope peak grouper. Peak alignment was performed using the join aligner method where absolute R_t_ tolerance is at 0.03 min. Adduct search was performed for Na^+^, K^+^, NH_4_^+^, and ACN^+^ in the positive mode and [M-2H+Na]^−^ and [M-2H+K]^−^ in the negative mode (Thomas et al., 2014). Custom database search restricted to *Fusarium* was performed using the Dictionary of Natural Products v29.2. Molecular networks were built through GNPS (Wang et al., 2016) and visualized using Cytoscape v3.8 (Shannon et al., 2003).

### HPLC-DAD-ELSD Analysis

HPLC-DAD analyses were conducted on an HP 1260 system equipped with a diode-array detection unit (Agilent Technologies, Santa Clara, CA, United States) using an X-Bridge C18 column (250 × 4.6 mm i.d., 5 μm; Waters, Milford, MA, United States). The HPLC conditions were as follows: mobile phase H_2_O (A) and MeOH (B), both containing 0.1% FA. The flow rate was 1 ml/min, the injection volume was 10 μl, the separation temperature was 25°C, and the sample concentration was 10 mg/ml dissolved in MeOH. The gradient conditions were set following a gradient transfer from UHPLC to HPLC according to the work of (Guillarme et al., 2008). The separation conditions were optimized by decreasing the slope of the mobile phase gradient to ensure the best distribution of the metabolites across the chromatographic window. The gradient method was set as follows: 34–100% of B in 60 min, followed by 10 min of washing with 100% B. The detection was performed by DAD and ELSD. The DAD parameters were set as follows: UV wavelength at 210, 254, 280, and 366 nm, five spectra acquired per peak, and the threshold was 5 mAU. The ELSD parameters were set as follows: pressure 3.4 bar, 45°C, split to provide a 500 μl/min flow rate, gain 8.

### Generation of 2D Pseudo-LC-NMR Plot

For the generation of the pseudo-LC-NMR 2D plot (Figure 1F), aligned ^1^H-NMR spectra of all 135 fractions were divided into equally sized bins (0.01 ppm) in a range from −1 to 15 ppm and then exported as an Excel file (.csv) using MNova v14 (MasterLab, Santiago De Compostela, Spain). Sample names (number of fractions) were added manually to the Excel file. The file was loaded into an R script using Rstudio V1.2.5042, which was written to create an interactive 2D plot that combines all binned spectra in a matrix (sample vs. ppm). The following R packages were used: “plotly,” “stringr,” “reshape2,” “dplyr,” and “readr.” The R script is freely available here: (https://github.com/oolonek/pseudo_lcnmr_plotter/blob/main/src/plotter/NMR_data_plotter.Rmd). To improve the visualization, retention time (R_t_) of the semi-preparative separation was manually added to the created plot (Figure 1F).

### Liquid/Liquid Extraction and Extract Purification on the Semi-Preparative Scale

The crude extract was subjected to liquid/liquid partition; 160 mg was suspended in 50 ml 7:3 methanol/water, and 50 ml of hexane was added to the suspension and then gently mixed to avoid emulsion. The two phases were separated using a separating funnel, and this procedure was repeated four times. Both phases were completely dried using a rotary evaporator (Buchi, Flawil, Switzerland). The hydroalcoholic fraction yielded 70 mg, and the hexane fraction yielded 90 mg.

The original crude extract and enriched extracts were purified using semi-preparative HPLC-UV equipment (Shimadzu^®^ SPD-20A, Kyoto, Japan) through an X-Bridge C18 column (250 × 19 mm i.d., 5 μm; Waters, Milford, MA, United States). The gradient transfer from analytical to semi-preparative HPLC was calculated according to the work of (Guillarme et al., 2008). The flow rate was set at 17 ml/min, and the separation was conducted at room temperature. In order to avoid loss of resolution, samples were introduced into the column through a homemade dry load cell (Queiroz et al., 2019). Collection of fractions from the crude extract was done automatically every 30 s. For enriched extracts, all peaks have been collected manually by observing the UV response at 254 nm. After collection, each fraction was evaporated to dryness using a high-performance evaporation system (HT-4X Genevac^®^, Stone Ridge, NY, United States).

### Description of the New Compounds

The spectral data for all new NPs are summarized below; those recorded for previously isolated compounds can be found in the supplementary materials. The 1D- and 2D-NMR spectra of all described compounds below can be found in the supplementary materials (Supplementary Figures S5–S109). All NMR data produced in the article are available in the public archive Yareta (https://doi.org/10.26037/yareta:tmpbgvbqsvfrvltepotmjuunua).

**6-(2,3-dihydroxybutan-2-yl)-3-methyl-2H-pyran-2-one** (**2**): white amorphous solid; **2a**: ^1^H-NMR (DMSO-*d*_6_, 600 MHz) δ 0.97 (3H, d, *J* = 6.3 Hz, H_3_-9), 1.31 (3H, s, H_3_-10), 1.95 (3H, d, *J* = 1.2 Hz, H_3_-11), 3.73 (1H, m, H-8), 4.67 (1H, d, *J* = 6.1 Hz, OH-8), 5.19 (1H, s, OH-7), 6.32 (1H, d, *J* = 5.7 Hz, H-5), 7.35 (1H, m, H-4); ^13^C-NMR (DMSO-*d*_6_, 151 MHz) δ 16.0 (C-11), 17.4 (C-9), 21.8 (C-10), 70.2 (C-8), 74.5 (C-7), 101.4 (C-5), 121.3 (C-3), 140.4 (C-4), 162.7 (C-2), 167.0 (C-6); **2b**: ^1^H-NMR (DMSO-*d*_6_, 600 MHz) δ 1.03 (2H, d, *J* = 6.4 Hz, H_3_-9), 1.24 (3H, s, H_3_-10), 1.95 (3H, d, *J* = 1.2 Hz, H_3_-11), 3.74 (1H, m, H-8), 4.59 (1H, d, *J* = 5.8 Hz, OH-8), 5.09 (1H, s, OH-7), 6.33 (1H, d, *J* = 5.8 Hz, H-5), 7.35 (1H, m, H-4); ^13^C-NMR (DMSO-*d*_6_, 151 MHz) δ 16.0 (C-11), 17.1 (C-9), 22.6 (C-10), 70.4 (C-8), 75.0 (C-7), 101.4 (C-5), 120.9 (C-3), 140.4 (C-4), 162.7 (C-2), 167.2 (C-6). HRMS *m/z* 199.0962 [M + H]^+^ (calculated for C_10_H_15_O_4_, 199.0966).

**6-(1-hydroxyethyl)-3-methyl-2H-pyran-2-one** (**3**): white amorphous solid; ^1^H-NMR (DMSO-*d*_6_, 600 MHz) δ 1.30 (3H, d, *J* = 6.6 Hz, H_3_-8), 1.96 (3H, d, *J* = 1.2 Hz, H_3_-9), 4.41 (1H, q, *J* = 6.6 Hz, H-7), 5.56 (1H, d, *J* = 4.4 Hz, OH-7), 6.26 (1H, dd, *J* = 6.7, 0.8 Hz, H-5), 7.36 (1H, m, H-4); ^13^C-NMR (DMSO-*d*_6_, 151 MHz) δ 16.2 (C-9), 21.5 (C-8), 64.9 (C-7), 100.4 (C-5), 122.0 (C-3), 140.5 (C-4), 162.7 (C-2), 165.6 (C-6). HRMS *m/z* 155.0703 [M + H]^+^ (calculated for C_8_H_11_O_3_, 155.0708). **2a**, **2b** and **3** occurred as a mixture in a fraction 10 (0.6 mg)

**5-hydroxy-4-(hydroxymethyl)-8-methoxy-2-methyl-1H-benzo[*g*]indole-6,9-dione** (**6**): pale rose amorphous solid (0.2 mg); UV λ_max_ 213, 280 nm; ^1^H-NMR (DMSO-*d*_6_, 600 MHz) δ 2.44 (3H, d, *J* = 1.0 Hz, H_3_-14), 3.89 (3H, s, H_3_-16), 4.75 (2H, d, *J* = 5.5 Hz, H_2_-15), 5.06 (1H, t, *J* = 5.5 Hz, OH-15), 6.23 (1H, s, H-3), 6.44 (1H, dd, *J* = 2.1, 1.1 Hz, H-11), 11.39 (1H, s, NH-13), 12.88 (1H, s, OH-5); ^13^C-NMR (DMSO-*d*_6_, 151 MHz) δ 13.8 (C-14), 55.1 (C-15), 56.6 (C-16), 99.8 (C-11), 107.4 (C-10), 109.0 (C-3), 126.8 (C-6), 130.5 (C-8), 137.5 (C-7), 146.7 (C-12), 152.1 (C-5), 160.5 (C-2), 179.5 (C-1), 191.0 (C-4); HRMS *m/z* 288.0846 [M + H]^+^ (calculated for C_15_H_14_NO_5_, 288.0866).

**(6*E*)-7-(4-methoxy-6-oxo-6H-pyran-2-yl)-3,5-dimethyloct-6-enoic acid** (**9**): Red amorphous solid (0.4 mg); [α]^20^_D_ +22.3 (c 0.04, MeOH);UV (DAD) λ_max_ 218, 269; ^1^H-NMR (DMSO-*d*_6_, 600 MHz) δ 0.89 (3H, d, *J* = 6.6 Hz, H_3_-14), 0.96 (3H, d, *J* = 6.6 Hz, H_3_-15), 1.21 (1H, dt, *J* = 13.4, 7.4 Hz, H-10''), 1.34 (1H, dt, *J* = 13.4, 6.8 Hz, H-10'), 1.79 (1H, dqd, *J* = 8.5, 6.8, 5.1 Hz, H-11), 1.86 (3H, d, *J* = 1.4 Hz, H_3_-16), 1.96 (1H, dd, *J* = 15.0, 8.5 Hz, H-12''), 2.25 (1H, m, H-12'), 2.67 (1H, m, H-9), 3.88 (3H, s, H_3_-17), 5.52 (1H, d, *J* = 1.8 Hz, H-3), 6.11 (1H, d, *J* = 1.8 Hz, H-5), 6.15 (1H, dd, *J* = 9.8, 1.4 Hz, H-8), 11.99 (1H, s, COOH); ^13^C-NMR (DMSO-*d*_6_, 151 MHz) δ 12.3 (C-16), 19.8 (C-14), 19.9 (C-15), 27.6 (C-11), 29.9 (C-9), 40.9 (C-12), 43.3 (C-10), 56.4 (C-17), 88.7 (C-3), 108.7 (C-5), 124.3 (C-7), 140.0 (C-8), 159.6 (C-6), 167.2 (C-4), 173.7 (C-13), 180.2 (C-2). HRMS *m/z* 295.1542 [M + H]^+^ (calculated for C_16_H_23_O_5_, 295.1545).

**6-((*E*)-6-ethyl-7-hydroxy-4-methylhept-2-en-2-yl)-4-methoxy-2H-pyran-2-one** (**10**): Red amorphous solid (0.7 mg); [α]^20^_D_ +82.7 (c 0.03, MeOH); UV (DAD) λ_max_ 286, 422; ^1^H-NMR (DMSO-*d*_6_, 600 MHz) δ 0.81 (3H, t, *J* = 7.3 Hz, H_3_-13), 0.98 (3H, d, *J* = 6.6 Hz, H_3_-15), 1.22 (1H, m, H-10''), 1.23 (1H, m, H-11), 1.27 (2H, m, H_2_-12), 1.35 (1H, m, H-10'), 1.86 (3H, d, *J* = 1.3 Hz, H_3_-16), 2.69 (1H, m, H-9), 3.25 (1H, dt, *J* = 10.2, 5.0 Hz, H-14''), 3.30 (1H, dt, *J* = 10.2, 5.0 Hz, H-14'), 3.88 (3H, s, H_3_-17), 4.34 (1H, t, *J* = 5.2 Hz, OH-14), 5.52 (1H, d, *J* = 1.8 Hz, H-3), 6.10 (1H, d, *J* = 1.8 Hz, H-5), 6.14 (1H, dd, *J* = 9.9, 1.5 Hz, H-8); ^13^C-NMR (DMSO-*d*_6_, 151 MHz) δ 10.5 (C-13), 12.1 (C-16), 20.4 (C-15), 22.6 (C-12), 29.9 (C-9), 37.8 (C-10), 39.1 (C-11), 56.3 (C-17), 62.9 (C-14), 88.6 (C-3), 108.6 (C-5), 124.1 (C-7), 140.4 (C-8), 159.6 (C-6), 167.2 (C-4), 180.2 (C-2). HRMS *m/z* 281.1745 [M + H]^+^, calculated for C_16_H_25_O_4_, 281.1747.

**5β,6β-23,26-diepoxy-3β,7α,9α-trihydroxy-(20Z,23S,24S,25R)ergosta-8(14),20-dien-15-one** (**12**): pale yellow amorphous solid (0.4 mg); [α]^20^_D_ +18.9 (c 0.04, MeOH); UV λ_max_ 248, 229, 229; ^1^H-NMR (DMSO-*d*_6_, 600 MHz) δ 0.77 (3H, s, H_3_-18), 0.85 (3H, d, *J* = 7.0 Hz, H_3_-28), 0.89 (3H, s, H_3_-19), 0.90 (3H, d, *J* = 6.8 Hz, H_3_-26), 1.20 (1H, dt, *J* = 13.6, 3.3 Hz, H-4α), 1.45 (3H, m, H-1β, H-2β, H-11α), 1.66 (2H, m, H_2_-12), 1.69 (1H, m, H-11β), 1.73 (3H, d, *J* = 1.2 Hz, H_3_-21), 1.85 (2H, m, H-2α, H-24), 2.00 (1H, td, *J* = 14.4, 4.0 Hz, H-1α), 2.06 (1H, dd, *J* = 13.6, 11.5 Hz, H-4β), 2.16 (1H, dd, *J* = 18.3, 7.3 Hz, H-16α), 2.26 (1H, m, H-25), 2.35 (1H, m, H-17), 2.63 (1H, m, H-16β), 3.27 (1H, d, *J* = 3.7 Hz, H-6), 3.59 (1H, tq, *J* = 10.6, 5.0 Hz, H-3), 3.93 (1H, dd, *J* = 8.2, 6.4 Hz, H-27'), 4.18 (1H, t, *J* = 8.5 Hz, H-23), 4.77 (1H, d, *J* = 5.0 Hz, OH-3), 4.77 (1H, d, *J* = 2.3 Hz, OH-9), 4.91 (1H, d, *J* = 5.2 Hz, OH-7), 5.22 (1H, d, *J* = 8.4 Hz, H-22), 5.53 (1H, dd, *J* = 5.2, 3.7 Hz, H-7); ^13^C-NMR (DMSO-*d*_6_, 151 MHz) δ 11.2 (C-28), 13.5 (C-26), 17.6 (C-18), 18.8 (C-21), 19.8 (C-19), 25.6 (C-1), 26.7 (C-11), 30.2 (C-2), 32.1 (C-12), 35.9 (C-25), 37.0 (C-10), 39.5 (C-16), 39.9 (C-4), 42.9 (C-24), 43.1 (C-13), 51.5 (C-17), 59.7 (C-7), 60.4 (C-6), 66.5 (C-3), 68.1 (C-5), 73.7 (C-27), 74.8 (C-9), 79.7 (C-23), 128.1 (C-22), 134.1 (C-20), 142.8 (C-14), 206.7 (C-15). HRMS *m/z* 473.2877 [M + H]^+^ (calculated for C_28_H_41_O_6_, 473.2903).

**2-(2,3,5,6,7,7a-hexahydro-1-((*E*)-6-hydroxy-5,6-dimethylhept-3-en-2-yl)-7a-methyl-5-oxo-1H-inden-4-yl)acetic acid** (**13**): pale yellow amorphous solid (0.4 mg); [α]^20^_D_ +21.5 (c 0.04, MeOH); UV λ_max_ 247, 207; ^1^H-NMR (DMSO, 600 MHz) δ 0.92 (3H, d, *J* = 7.0 Hz, H_3_-28), 0.99 (3H, s, H_3_-27), 1.03 (3H, d, *J* = 7.1 Hz, H_3_-21), 1.03 (3H, s, H_3_-26), 1.06 (3H, s, H_3_-18), 1.42 (1H, m, H-17), 1.50 (1H, m, H-16''), 1.78 (1H, m, H-16'), 1.79 (1H, m, H-12''), 2.01 (1H, p, *J* = 7.0 Hz, H-24), 2.16 (1H, m, H-12'), 2.17 (1H, m, H-20), 2.19 (1H, m, H-11''), 2.22 (1H, m, H-11'), 2.99 (1H, d, *J* = 16.5 Hz, H-7''), 3.02 (1H, d, *J* = 16.5 Hz, H-7'), 5.26 (1H, dd, *J* = 15.3, 8.7 Hz, H-22), 5.41 (1H, dd, *J* = 15.3, 7.0 Hz, H-23); ^13^C-NMR (DMSO-*d*_6_, 151 MHz) δ 14.9 (C-28), 16.2 (C-18), 20.8 (C-21), 26.0 (C-27), 27.0 (C-16), 28.2 (C-26), 30.8 (C-7), 32.7 (C-11), 35.8 (C-12), 38.1 (C-20), 44.7 (C-13), 47.1 (C-24), 55.5 (C-17), 70.5 (C-25), 131.1 (C-23), 135.0 (C-22), 171.7 (C-6), 175.2 (C-14). HRMS *m/z* 349.2383 [M + H]^+^ (calculated for C_21_H_33_O_4_, 349.2378).

**2-(2,3,5,6,7,7a-hexahydro-1-((*E*)-7-hydroxy-5,6-dimethylhept-3-en-2-yl)-7a-methyl-5-oxo-1H-inden-4-yl)acetic acid** (**14**): pale yellow amorphous solid (0.3 mg); [α]^20^_D_ +19.8 (c 0.07, MeOH); UV λ_max_ 245, 212; ^1^H-NMR (DMSO-*d*_6_, 600 MHz) δ 0.75 (3H, d, *J* = 6.9 Hz, H_3_-26), 0.94 (3H, d, *J* = 7.0 Hz, H_3_-28), 1.03 (3H, d, *J* = 6.7 Hz, H_3_-21), 1.06 (3H, s, H_3_-18), 1.43 (2H, m, H-17, H-25), 1.51 (1H, p, *J* = 11.4 Hz, H-16''), 1.78 (2H, m, H-12'', H-16'), 2.17 (3H, m, H-12', H-20, H-24), 2.22 (1H, dd, *J* = 18.7, 4.8 Hz, H-11''), 2.53 (1H, overlapped, H-11'), 2.98 (1H, d, *J* = 16.4 Hz, H-7''), 3.02 (1H, d, *J* = 16.4 Hz, H-7'), 3.16 (1H, d, *J* = 6.4 Hz, H-27'), 3.17 (1H, d, *J* = 6.4 Hz, H-27''), 5.27 (2H, m, H-22, H-23); ^13^C-NMR (DMSO-*d*_6_, 151 MHz) δ 13.1 (C-26), 16.3 (C-18), 18.3 (C-28), 21.0 (C-21), 27.3 (C-16), 30.9 (C-7), 32.7 (C-11), 35.9 (C-12), 37.0 (C-24), 38.2 (C-20), 40.6 (C-25), 44.6 (C-13), 55.5 (C-17), 64.4 (C-27), 130.9 (C-23), 135.0 (C-22), 171.8 (C-6), 175.2 (C-14). HRMS *m/z* 349.2349 [M + H]^+^ (calculated for C_21_H_33_O_4_, 349.2378).

**5-hydroxy-8-methoxy-2,4-dimethyl-1*H*-benzo[g]indole-6,9-dione** (**15**): pale yellow amorphous (0.1 mg) solid; UV λ_max_ 280, 239; ^1^H NMR (DMSO-*d*_6_, 600 MHz) δ 2.36 (3H, s, H_3_-15), 2.44 (3H, s, H_3_-14), 3.89 (3H, s, H_3_-16), 6.21 (1H, s, H-3), 6.28 (1H, s, H-11), 11.39 (1H, s, NH-13), 12.84 (1H, s, OH-5); ^13^C-NMR (DMSO-*d*_6_, 151 MHz) δ 12.0 (C-15), 13.8 (C-14), 56.6 (C-16), 98.9 (C-11), 107.3 (C-10), 108.8 (C-3), 123.9 (C-6), 129.8 (C-8), 137.6 (C-7), 146.2 (C-12), 152.2 (C-5), 160.5 (C-2), 179.1 (C-1). HRMS *m/z* 258.0771 [M + H]^+^, calculated for C_14_H_12_NO_4_, 258.0766.

**4-methoxy-6-((*E*)-4,6-dimethyloct-2-en-2-yl)-2H-pyran-2-one** (**16**): light brown amorphous solid (0.1 mg); [α]^20^_D_ +17.5 (c 0.04, MeOH); UV λ_max_ 220,274; ^1^H-NMR (DMSO-*d*_6_, 600 MHz) δ 0.82 (3H, t, *J* = 7.4 Hz, H_3_-13), 0.84 (3H, d, *J* = 6.3 Hz, H_3_-14), 0.96 (3H, d, *J* = 6.5 Hz, H_3_-15), 1.08 (1H, m, H-12''), 1.14 (1H, m, H-10''), 1.32 (3H, m, H-10', H-11, H-12'), 1.86 (3H, d, *J* = 1.2 Hz, H_3_-16), 2.66 (1H, m, H-9), 3.88 (3H, s, H_3_-17), 5.52 (1H, d, *J* = 1.8 Hz, H-3), 6.10 (1H, d, *J* = 1.8 Hz, H-5), 6.16 (1H, dq, *J* = 9.8, 1.2 Hz, H-8); ^13^C-NMR (DMSO-*d*_6_, 151 MHz) δ 11.0 (C-13), 12.3 (C-16), 19.3 (C-14), 20.1 (C-15), 28.6 (C-12), 30.1 (C-9), 31.5 (C-11), 43.5 (C-10), 56.3 (C-17), 88.8 (C-3), 108.9 (C-5), 124.2 (C-7), 140.5 (C-8), 159.7 (C-6), 167.4 (C-4). HRMS *m/z* 265.1816 [M + H]^+^, calculated for C_16_H_25_O_3_, 265.1803.

**4-methoxy-3-methyl-6-((*E*)-4,6-dimethyloct-2-en-2-yl)-2H-pyran-2-one** (**17**): light brown amorphous solid (0.1 mg); [α]^20^_D_ +19.7 (c 0.04, MeOH); UV λ_max_ 218, 252; ^1^H-NMR (DMSO-*d*_6_, 600 MHz) δ 0.83 (3H, t, *J* = 7.4 Hz, H_3_-13), 0.85 (3H, d, *J* = 6.1 Hz, H_3_-14), 0.98 (3H, d, *J* = 6.7 Hz, H_3_-15), 1.24 (1H, m, H-12''), 1.34 (2H, m, H-11, H-12'), 1.69 (3H, s, H_3_-18), 1.88 (3H, d, *J* = 1.3 Hz, H_3_-16), 2.66 (1H, m, H-9), 4.02 (3H, s, H_3_-17), 6.17 (1H, s, H-5), 6.19 (1H, d, *J* = 9.4 Hz, H-8); ^13^C-NMR (DMSO-*d*_6_, 151 MHz) δ 6.5 (C-18), 11.1 (C-13), 12.3 (C-16), 19.3 (C-14), 20.1 (C-15), 28.7 (C-12), 30.1 (C-9), 31.4 (C-11), 43.5 (C-10), 56.0 (C-17), 98.9 (C-3), 108.3 (C-5), 124.4 (C-7), 140.1 (C-8), 158.3 (C-6), 162.0 (C-4), 179.7 (C-2). HRMS *m/z* 279.1967 [M + H]^+^ (calculated for C_17_H_27_O_3_, 279.1955).

**2-(2,3,5,6,7,7a-hexahydro-7a-methyl-1-((*E*)-5,6-dimethylhept-3-en-2-yl)-5-oxo-1H-inden-4-yl)acetic acid** (**18**): pale yellow amorphous solid (0.6 mg); [α]^20^_D_ +14.9 (c 0.05, MeOH); UV λ_max_ 246, 239; ^1^H-NMR (DMSO-*d*_6_, 600 MHz) δ 0.81 (3H, d, *J* = 6.9 Hz, H_3_-27), 0.83 (3H, d, *J* = 6.9 Hz, H_3_-26), 0.91 (3H, d, *J* = 6.7 Hz, H_3_-28), 1.03 (3H, d, *J* = 6.6 Hz, H_3_-21), 1.06 (3H, s, H_3_-18), 1.42 (1H, m, H-17), 1.48 (1H, m, H-25), 1.51 (1H, m, H-16''), 1.78 (1H, m, H-16'), 1.79 (1H, m, H-12''), 1.88 (1H, m, H-24), 2.15 (1H, m, H-12'), 2.18 (1H, m, H-20), 2.22 (1H, m, H-11''), 2.34 (1H, m, H-15''), 2.46 (1H, m, H-15'), 2.56 (1H, m, H-11'), 2.98 (1H, d, *J* = 16.5 Hz, H-7''), 3.02 (1H, d, *J* = 16.5 Hz, H-7'), 5.25 (1H, dd, *J* = 15.2, 7.9 Hz, H-22), 5.29 (1H, dd, *J* = 15.2, 7.0 Hz, H-23); ^13^C-NMR (DMSO-*d*_6_, 151 MHz) δ 16.3 (C-18), 17.3 (C-28), 19.4 (C-27), 19.7 (C-26), 20.9 (C-21), 27.2 (C-16), 27.3 (C-15), 30.9 (C-7), 32.4 (C-25), 32.7 (C-11), 35.8 (C-12), 38.1 (C-20), 42.0 (C-24), 44.6 (C-13), 55.4 (C-17), 125.0 (C-8), 132.0 (C-23), 134.7 (C-22), 171.7 (C-6), 175.2 (C-14), 196.3 (C-9). HRMS *m/z* 333.2426 [M + H]^+^ (calculated for C_21_H_33_O_3_, 333.2429).

**3-*O*-*β*-D-glucopyranoside-Stigmast-8-en-3-ol** (**19**): pale yellow amorphous solid (0.1 mg); [α]^20^_D_ +6.2 (c 0.08, MeOH); UV λ_max_ 234, 207; ^1^H-NMR (DMSO-*d*_6_, 600 MHz) δ 0.65 (3H, s, H_3_-18), 0.79 (3H, d, *J* = 6.9 Hz, H_3_-27), 0.82 (3H, d, *J* = 6.9 Hz, H_3_-26), 0.82 (3H, t, *J* = 7.3 Hz, H_3_-29), 0.88 (1H, m, H-5), 0.90 (3H, d, *J* = 6.5 Hz, H_3_-21), 0.91 (1H, m, H-24), 0.96 (3H, s, H_3_-19), 0.97 (2H, m, H-1b, H-14), 1.01 (1H, m, H-22b), 1.09 (1H, m, H-17), 1.20 (1H, m, H-28b), 1.25 (1H, m, H-28a), 1.30 (1H, m, H-22a), 1.33 (1H, m, H-20), 1.47 (1H, m, H-2b), 1.63 (1H, m, H-25), 1.79 (1H, m, H-1a), 1.80 (3H, s), 2.12 (1H, t, *J* = 12.5 Hz, 4''), 1.81 (1H, m, H-2a), 2.12 (1H, m, H-4b), 2.37 (1H, m, H-4a), 2.89 (1H, td, *J* = 8.4, 4.8 Hz, H-2'), 3.01 (1H, td, *J* = 9.2, 5.0 Hz, H-4'), 3.06 (1H, m, H-5'), 3.12 (1H, td, *J* = 8.9, 4.8 Hz, H-3'), 3.40 (1H, m, H-6'b), 3.46 (1H, tt, *J* = 11.2, 4.3 Hz, H-3), 3.64 (1H, dd, *J* = 11.1, 6.2 Hz, H-6'a), 4.22 (1H, d, *J* = 7.8 Hz, H-1'), 4.42 (1H, t, *J* = 5.8 Hz, OH-6'), 4.85 (1H, d, *J* = 5.0 Hz, OH-4'), 4.86 (1H, d, *J* = 4.8 Hz, OH-2'), 4.88 (1H, d, *J* = 4.8 Hz, OH-3'); ^13^C-NMR (DMSO-*d*_6_, 151 MHz) δ 11.6 (C-18), 11.8 (C-29), 18.6 (C-21), 19.0 (C-27), 19.1 (C-19), 19.6 (C-26), 22.5 (C-28), 28.7 (C-25), 29.3 (C-2), 33.3 (C-22), 35.4 (C-20), 36.3 (C-10), 36.7 (C-1), 38.3 (C-4), 39.1 (C-12), 41.8 (C-13), 45.1 (C-24), 49.6 (C-5), 55.4 (C-17), 56.1 (C-14), 61.0 (C-6'), 70.1 (C-4'), 73.4 (C-2'), 76.7 (C-5'), 76.8 (C-3, C-3'), 100.7 (C-1'), 140.4 (C-9). HRMS *m/z* 599.4280 [M + Na]^+^, calculated for C_35_H_59_O_6_Na, 599.4287.

### Minimum Inhibitory Concentration Test

Methicillin-resistant *Staphylococcus aureus* (MRSA, ATCC 33591) and *Pseudomonas aeruginosa* (ATCC 27853) strains were used for the antibacterial assay. The minimum inhibitory concentration (MIC) of the extract and the isolated compounds were determined in triplicate according to Wiegand et al. (2008) in Mueller–Hinton medium (MH). After the incubation of the inoculated 96-well plates at 37°C for 24 h, iodonitrotetrazolium chloride (INT, Sigma-Aldrich) was added to each well at a final concentration of 0.2 mg/ml and incubated for 20 min (Eloff, 1998). The highest dilution of a compound in which no growth appears corresponds to its MIC. Gentamicin for *P. aeruginosa* and chloramphenicol for *S. aureus* were used as controls.

### Anti-Quorum Sensing Assay for *Pseudomonas aeruginosa*

The assay was performed according to the protocol proposed by Hentzer et al. (2002) on a black 96-well plate with a clear bottom. The reporter strain PPAO1 *pqsA-gfp* was constructed using the following primers, GCT​CTA​GAT​CGA​GCA​AGG​GTT​GTA​ACG​GTT​TTT​G and GCT​GCT​GCA​TGC​GAC​AGA​ACG​TTC​CCT​CTT​CAG​CGA, to amplify the *pqsA* gene promoter and cloned using usual molecular methods into XbaI-SphI sites of the *lasB-gfp* plasmid (Hentzer et al., 2002), in place of the *lasB* promoter. Reporter strains containing the *lasB-gfp* or the *pqsA-gfp* plasmid were grown at a starting OD600 of 0.05 in PTSB (5% peptone, 0.25% trypticase soy broth) supplied with gentamicin 50 μg/ml and each sample at 128 μg/ml. Azithromycin (2 μg/ml) was used as the positive control. Plates were incubated at 37°C and 160 rpm. After 15 h, OD600 and fluorescence at 480/520 nm were measured using a microplate reader (SynergyHT BioTek). Results are represented in percentage of fluorescence compared to the solvent control (DMSO) fixed at 100%.

### Anti-Quorum Sensing Assay for *Staphylococcus aureus*

The assay was performed according to the protocol proposed by Nielsen et al. (2010). In a 96-well plate, the reporter strain *rnaIII-lacZ* (Nielsen et al., 2010) was grown at a starting OD600 of 0.05 in MH, supplied with erythromycin 5 μg/ml and each sample at 128 μg/ml. *Staphylococcus caprae* auto-inducing peptide (AIP) (Paharik et al., 2017) at a concentration of 1 µM was used as the positive control. After 6 h at 37°C and 160 rpm, incubation was stopped and the OD600 value was read. Then, 10 µl of freshly made 4-methylumbelliferyl-β-D-galactopyranoside (MUG) (10 mg/ml) was added to each well and left to incubate for 1 h at room temperature. The reaction was stopped by the addition of 100 µl of Na_2_CO_3_ (0.4M), and fluorescence was read using a microplate reader (SynergyHT BioTek) at 360/460 nm. Results are represented in percentage of fluorescence compared to the solvent control (DMSO) fixed at 100%.

### Quantitative qRT-PCR

Cultures were grown in triplicate for 4 h in the presence of the compound of interest at 128 μg/ml. Then, 1 ml of bacteria was treated with RNA protect Bacteria Reagent (Qiagen) before centrifugation and storage at −20°C. Pellets were resuspended in 100 μl TE (pH 8) and 2.5 µl of lysostaphin (10 mg/ml) for *S. aureus* PR01 and incubated for 10 min at 37°C, or 100 μl TE (pH 8) and lysozyme (1 mg/ml) and incubated for 5 min at RT for *P. aeruginosa* PAO1. RNA was extracted using an RNeasy kit (Qiagen) according to the manufacturer’s protocol. RNA was eluted in 40 µl of RNAse-free water, quantified using a Qubit 2.0 fluorometer (Invitrogen), and DNase treated with RQ1 RNase-free DNase (Promega) according to the manufacturer’s instructions. Then, 500 ng of RNA were reverse-transcribed into cDNA using random primers (Promega) and Improm-II reverse transcriptase (Promega) according to the manufacturer. qPCR was performed using SYBR select master mix (Thermo Fisher). Primers for the amplification of target genes are listed in Supplementary Table S4. *HU* and *oprF* genes were used for normalization for *S. aureus* and *P. aeruginosa*, respectively.

## Data Availability

The datasets presented in this study can be found in online repositories. The names of the repository/repositories and accession number(s) can be found below: https://yareta.unige.ch/#/home https://doi.org/10.26037/yareta:tmpbgvbqsvfrvltepotmjuunua.
