# Supplementary material for: Combination of Pseudo-LC-NMR and HRMS/MS-Based Molecular Networking for the Rapid Identification of Antimicrobial Metabolites From Fusarium petroliphilum
Source: Front Mol Biosci. 2021 Oct 22;8:725691. doi: 10.3389/fmolb.2021.725691 (PMC8569130; doi:10.3389/fmolb.2021.725691)
Supplement: Supplementary file 1 [file DataSheet2.PDF]

## Description of the known compounds isolated in this study.

**Adenosine (1)**: white amorphous solid (0.3 mg); $^1$H-NMR (DMSO-$d_6$, 600 MHz) δ 3.55 (1H, dd, *J*=12.1, 3.5 Hz, H-5'b), 3.67 (2H, dd, *J*=12.1, 3.5 Hz, H-5'a), 3.96 (2H, q, *J*=3.4 Hz, H-4'), 4.14 (1H, dd, *J*=4.8, 3.1 Hz, H-3'), 4.60 (1H, t, *J*=5.6 Hz, H-2'), 5.87 (1H, d, *J*=6.3 Hz, H-1'), 7.33 (1H, s, OH-5), 8.13 (1H, s, H-7), 8.34 (1H, s, H-2); $^{13}$C-NMR (DMSO-$d_6$, 151 MHz) δ 61.6 (C-5'), 70.6 (C-3'), 73.4 (C-2'), 85.8 (C-4'), 87.8 (C-1'), 119.3 (C-4), 139.8 (C-2), 156.1 (C-5). HRMS/MS *m/z* 268.1033 [M + H]$^+$ (calculated for $C_{10}H_{14}N_5O_4$, 268.1045).

**Gibepyrone D** (**4**): pale yellow amorphous solid (0.5 mg); $^1$H-NMR (DMSO-$d_6$, 600 MHz) δ 2.03 (3H, d, *J*=1.3 Hz, $H_3$-10), 2.27 (3H, s, $H_3$-11), 6.43 (1H, s, H-8), 6.72 (1H, d, *J*=6.9 Hz, H-5), 7.44 (1H, dd, *J*=6.9, 1.3 Hz, H-4); $^{13}$C-NMR (DMSO-$d_6$, 151 MHz) δ 12.9 (C-11), 16.2 (C-10), 105.6 (C-5), 125.8 (C-3), 139.8 (C-4), 156.6 (C-6), 161.4 (C-2). HRMS/MS *m/z* 195.1016 [M + H]$^+$ (calculated for $C_{10}H_{11}O_4$, 195.0657).

**Aloesol** (**5**): milk white crystals (0.3 mg); $^1$H-NMR (DMSO-$d_6$, 600 MHz) δ 1.14 (3H, d, *J*=6.2 Hz, $H_3$-14), 2.54 (1H, dd, *J*=14.3, 7.7 Hz, H-12b), 2.60 (1H, dd, *J*=14.3, 5.2 Hz, H-12a), 2.65 (3H, s, $H_3$-11), 4.02 (1H, dq, *J*=7.6, 5.5 Hz, H-13), 4.83 (1H, d, *J*=5.1 Hz, OH-13), 5.96 (1H, s, H-3), 6.60 (1H, d, *J*=2.4 Hz, H-6), 6.62 (1H, d, *J*=2.4 Hz, H-8); $^{13}$C-NMR (DMSO-$d_6$, 151 MHz) δ 22.3 (C-11), 23.3 (C-14), 42.7 (C-12), 63.9 (C-13), 100.4 (C-8), 111.4 (C-3), 114.3 (C-10), 116.3 (C-6), 141.3 (C-5), 159.1 (C-7), 160.8 (C-9), 164.6 (C-2). HRMS/MS *m/z* 235.0963 [M + H]$^+$ (calculated for $C_{13}H_{15}O_4$, 235.0965).

**Fusarubin** (**7**): Reddish rose crystals; $^1$H-NMR (DMSO-$d_6$, 600 MHz) δ 1.48 (3H, s, $H_3$-11), 2.59 (1H, m, H-4''), 2.78 (1H, dd, *J*=18.0, 1.9 Hz, H-4'), 3.91 (3H, s, $H_3$-12), 4.67 (1H, dt, *J*=17.5, 2.4 Hz, H-1''), 4.72 (1H, d, *J*=17.5 Hz, H-1'), 6.13 (1H, d, *J*=1.6 Hz, OH-3), 6.46 (1H, s, H-8), 12.49 (1H, s, OH-5), 13.00 (1H, s, OH-10); $^{13}$C-NMR (DMSO-$d_6$, 151 MHz) δ 28.4 (C-11), 32.8 (C-4), 57.0 (C-12), 57.3 (C-1), 92.9 (C-3), 107.1 (C-9a), 109.7 (C-8), 133.3 (C-4a), 136.7 (C-10a), 156.3 (C-10), 159.6 (C-5), 160.7 (C-7), 177.8 (C-6), 184.7 (C-9). HRMS/MS *m/z* 307.0823 [M + H]$^+$ (calculated for $C_{15}H_{15}O_7$, 307.0812).

**3-*O*-methylfusarubin** (**8**): Reddish rose crystals; $^1$H-NMR (DMSO-$d_6$, 600 MHz) δ 1.47 (3H, s, $H_3$-11), 2.67 (1H, dt, *J*=18.0, 1.9 Hz, H-4''), 2.85 (1H, dd, *J*=18.0, 2.0 Hz, H-4'), 3.20 (3H, s, $H_3$-13), 3.91 (3H, s, $H_3$-12), 4.44 (1H, dt, *J*=17.7, 2.5 Hz, H-1''), 4.78 (1H, d, *J*=17.7 Hz, H-1'), 6.45 (1H, s, H-8), 12.49 (1H, s, OH-5), 12.99 (1H, s, OH-10); $^{13}$C-NMR (DMSO-$d_6$, 151 MHz) δ 22.4 (C-11), 32.5 (C-4), 48.3 (C-13), 57.0 (C-12), 57.9 (C-1), 96.3 (C-3), 109.7 (C-8), 132.1 (C-4a), 135.7 (C-10a), 155.4 (C-10), 158.7 (C-5), 160.7 (C-7), 178.4 (C-6), 185.1 (C-9). HRMS/MS *m/z* 319.0976 [M - H]$^-$ (calculated for $C_{16}H_{15}O_7$, 319.0817).

**Bostrycoidin** (**11**): Dark red amorphous solid (0.5 mg); $^1$H-NMR (DMSO-$d_6$, 600 MHz) δ 2.73 (3H, s, $H_3$-15), 3.98 (3H, s, $H_3$-16), 7.04 (1H, s, H-7), 7.98 (1H, s, H-4), 9.33 (1H, s, H-1), 12.87 (1H, s, OH-5/8), 13.54 (1H, s, OH-5/8); $^{13}$C-NMR (DMSO-$d_6$, 151 MHz) δ 24.5 (C-15), 56.7 (C-16), 107.8 (C-7), 117.5 (C-4), 124.0 (C-13), 138.4 (C-14), 147.9 (C-1), 157.6 (C-6), 164.8 (C-3). HRMS/MS *m/z* 286.0715 [M + H]$^+$ (calculated for $C_{15}H_{12}NO_5$, 286.0716).

**Cerevisterol** (**20**)**:** White amorphous solid (0.9 mg) $^1$H-NMR (DMSO-$d_6$, 600 MHz) δ 0.55 (3H, s, $H_3$-18), 0.80 (3H, d, *J*=7.3 Hz, $H_3$-27), 0.81 (3H, d, *J*=7.3 Hz, $H_3$-26), 0.88 (3H, d, *J*=6.8 Hz, $H_3$-28), 0.90 (3H, s, $H_3$-19), 0.99 (3H, d, *J*=6.5 Hz, $H_3$-21), 3.37 (1H, t, *J*=5.5, 4.8 Hz, H-6), 3.59 (1H, s, OH-5), 3.76 (1H, tq, *J*= 10.4, 5.0 Hz, H-3), 4.22 (1H, d, *J*=5.6 Hz, OH-3), 4.49 (1H, d, *J*=5.5 Hz, OH-6), 5.08 (1H, dt, *J*=4.8, 2.2 Hz, H-7), 5.17 (1H, dd, *J*=15.3, 8.3 Hz, H-22), 5.25 (1H, dd, *J*=15.3, 7.4 Hz, H-23); $^{13}$C-NMR (DMSO-$d_6$, 151 MHz) δ 12.1 (C-18), 17.2 (C-28), 17.7 (C-19), 19.5 (C-26), 19.8 (C-27), 20.9 (C-21), 21.3 (C-16), 22.5 (C-15), 27.6 (C-11), 31.1 (C-2), 32.4 (C-1, C-25), 36.6 (C-10), 38.9 (C-12), 39.9 (C-20), 40.1 (C-4), 42.0 (C-24), 42.2 (C-9), 42.9 (C-13), 54.1 (C-14), 55.3 (C-17), 65.9 (C-3), 72.1 (C-6), 74.4 (C-5), 119.5 (C-7),

131.4 (C-23), 138.4 (C-22), 139.6 (C-8). HRMS/MS *m/z* 453.3397 $[M + Na]^+$, calculated for $C_{28}H_{46}O_3Na$ 453.3345.

**6-Dehydrocerevisterol** (**21**): White amorphous solid (0.6 mg); $^1$H-NMR (DMSO-$d_6$, 600 MHz) δ 0.55 (3H, s, $H_3$-18), 0.78 (3H, s, $H_3$-19), 0.81 (3H, d, *J*=7.0 Hz, $H_3$-27), 0.80 (3H, d, *J*=7.0 Hz, $H_3$-26), 0.89 (3H, d, *J*=6.8 Hz, $H_3$-28), 1.00 (3H, d, *J*=6.6 Hz, $H_3$-21), 3.71 (1H, tq, *J*= 7.0, 5.5 Hz, H-3), 4.40 (1H, d, *J*=5.5 Hz, OH-3), 5.12 (1H, s, OH-5), 5.18 (1H, dd, *J*=15.3, 8.4 Hz, H-22), 5.25 (1H, dd, *J*=15.3, 7.4 Hz, H-23), 5.39 (1H, t, *J*=2.4 Hz, H-7); $^{13}$C-NMR (DMSO-$d_6$, 151 MHz) δ 12.4 (C-18), 15.8 (C-19), 17.3 (C-28), 19.5 (C-27), 19.7 (C-26), 20.9 (C-21), 21.2 (C-11), 22.0 (C-15), 27.6 (C-16), 30.0 (C-1), 30.4 (C-2), 32.5 (C-25), 35.9 (C-4), 38.1 (C-12), 39.8 (C-20), 40.1 (C-10), 42.0 (C-24), 43.2 (C-9), 44.0 (C-13), 54.8 (C-14), 55.2 (C-17), 65.5 (C-3), 76.0 (C-5), 119.3 (C-7), 131.6 (C-23), 135.1 (C-22), 163.3 (C-8), 198.6 (C-6). HRMS/MS *m/z* 429.3387 $[M + H]^+$, calculated for $C_{28}H_{45}O_3$, 429.3368.

**Ergosterol** (**22**): White amorphous solid (0.4 mg); $^1$H-NMR (DMSO-$d_6$, 600 MHz) δ 0.59 (3H, s, $H_3$-18), 0.80 (3H, d, *J*=6.9 Hz, $H_3$-27), 0.81 (3H, d, *J*=7.0 Hz, $H_3$-26), 0.86 (3H, s, $H_3$-19), 0.89 (3H, d, *J*=6.8 Hz, $H_3$-28), 1.01 (3H, d, *J*=6.6 Hz, $H_3$-21), 3.36 (1H, overlapped, H-3), 4.40 (1H, d, *J*=4.4 Hz, OH-3), 5.18 (1H, dd, *J*=15.3, 8.1 Hz, H-22), 5.24 (1H, dd, *J*=15.3, 7.2 Hz, H-23), 5.32 (1H, dt, *J*=5.0. 2.1 Hz, H-7), 5.47 (1H, dd *J*=5.5. 2.1 Hz, H-7); $^{13}$C-NMR (DMSO-$d_6$, 151 MHz) δ 11.8 (C-18), 16.0 (C-19), 17.3 (C-28), 19.5 (C-26), 19.8 (C-27), 20.6 (C-11), 21.0 (C-21), 22.6 (C-15), 27.9 (C-16), 31.8 (C-2), 32.5 (C-25), 36.6 (C-10), 37.9 (C-1), 38.4 (C-12), 39.9 (C-20), 40.7 (C-4), 42.0 (C-24), 42.3 (C-13), 45.7 (C-9), 53.9 (C-14), 55.0 (C-17), 68.6 (C-3), 116.2 (C-7), 118.7 (C-6), 131.4 (C-23), 135.4 (C-22), 140.2 (C-8), 140.6 (C-5). HRMS/MS *m/z* 395.3325 $[M - H]^-$, calculated for $C_{28}H_{45}O$, 395.3313.

## 1D and 2D NMR spectra of all compounds isolated in this study.

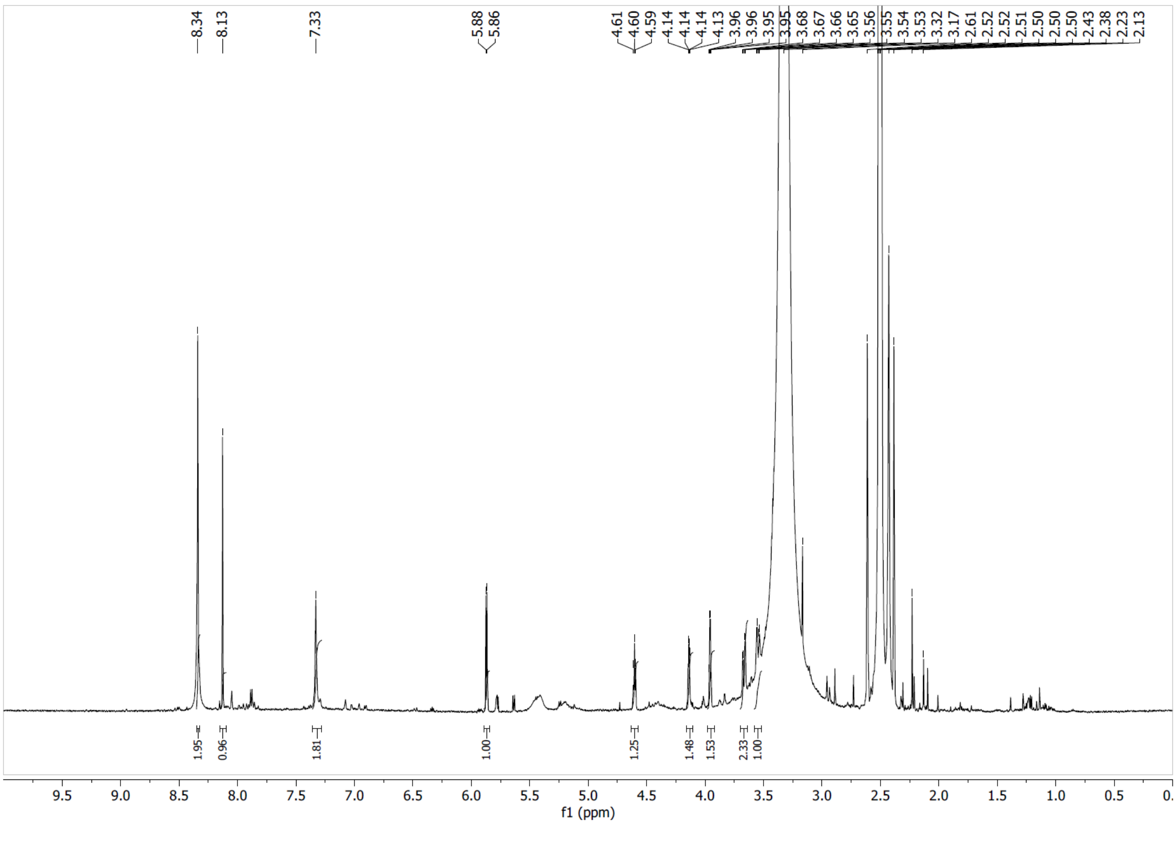


Figure S5: $^1$H NMR spectrum of compound 1 in DMSO-$d_6$ at 600 MHz

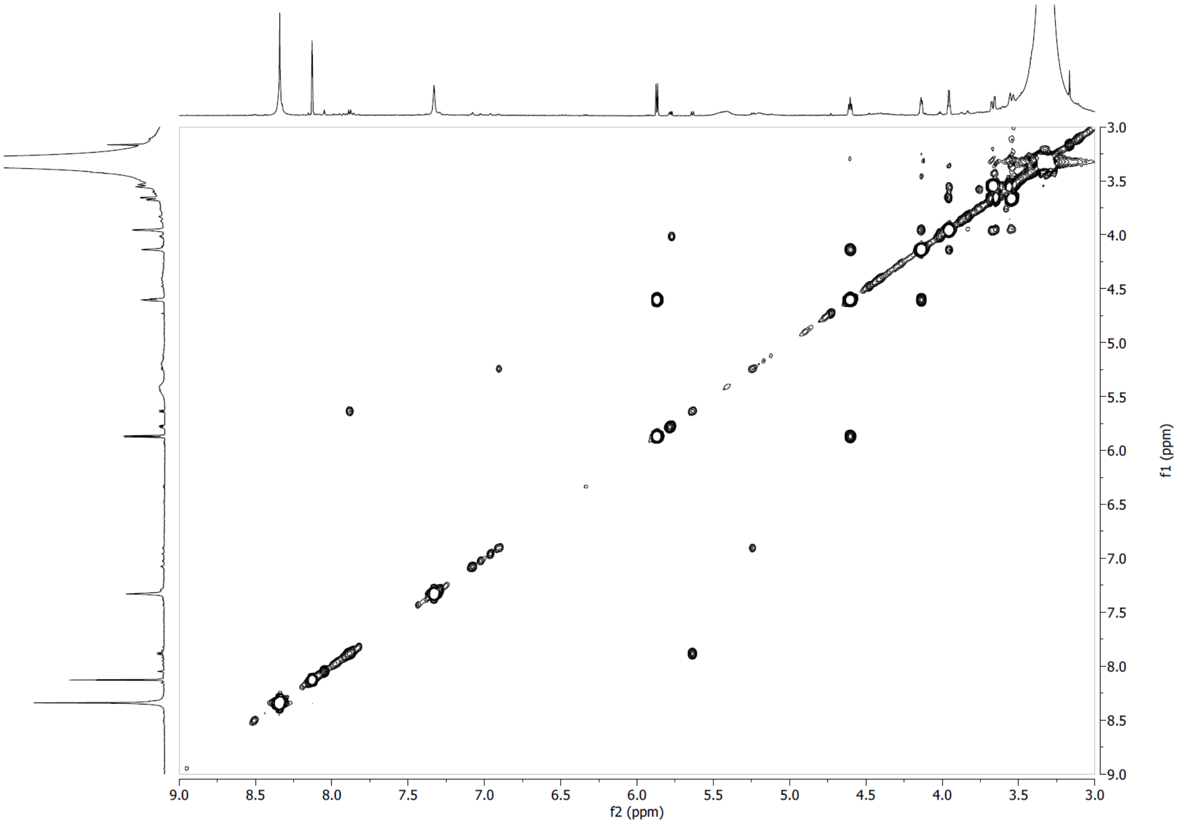


Figure S6: COSY NMR spectrum of compound **1** in DMSO-$d_6$

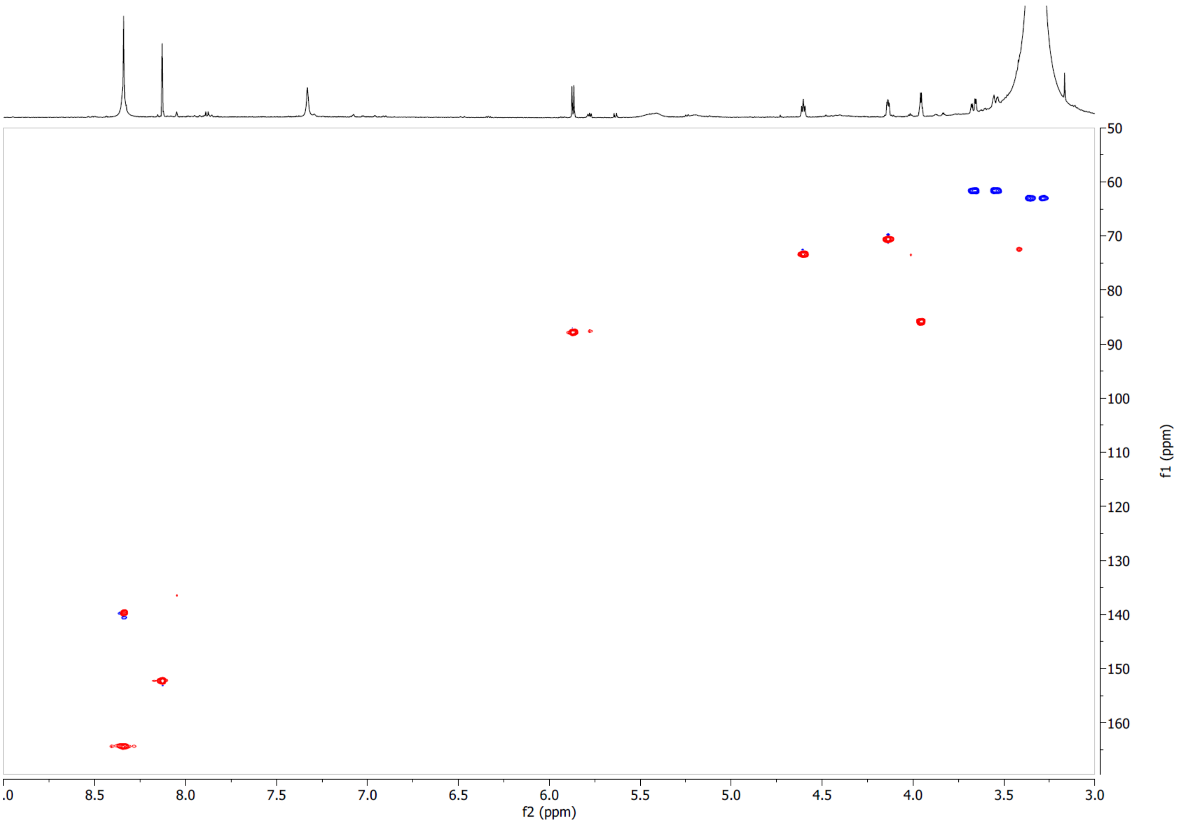


Figure S7: Edited-HSQC NMR spectrum of compound **1** in DMSO-$d_6$

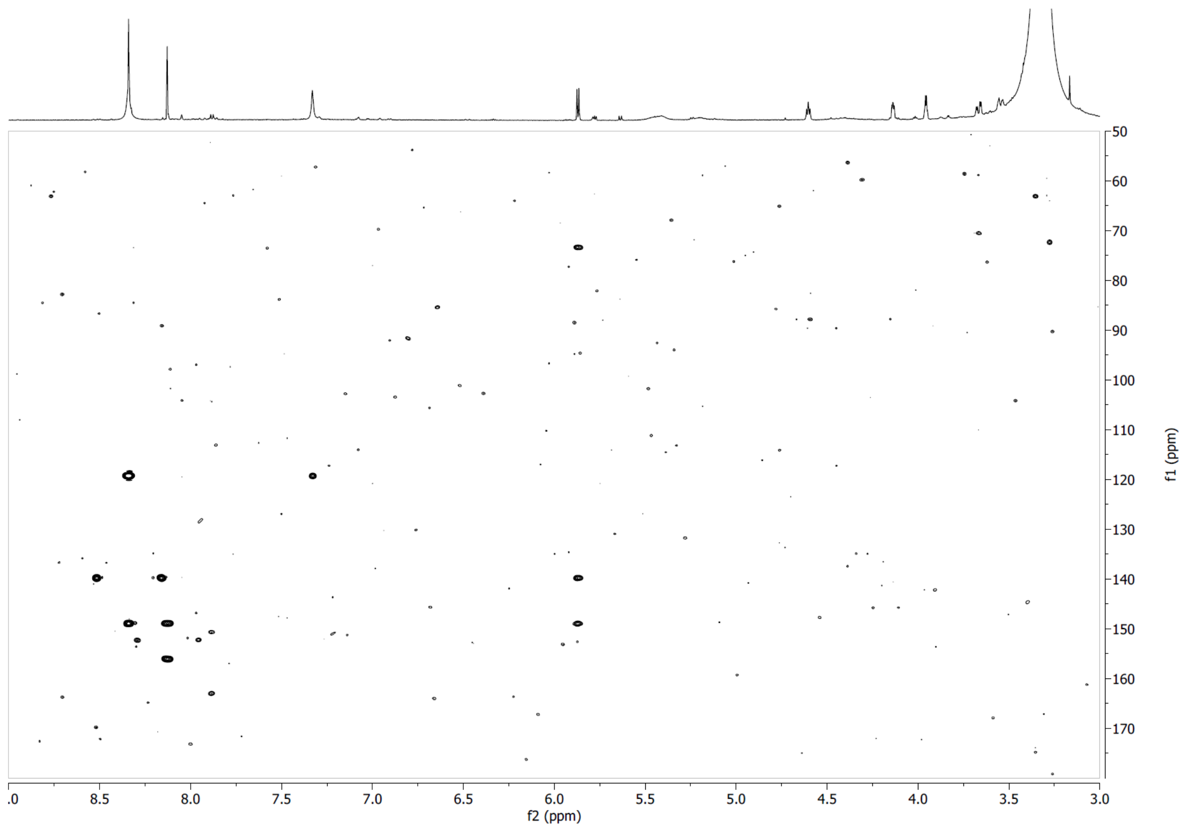


Figure S8: HMBC NMR spectrum of compound **1** in DMSO-$d_6$

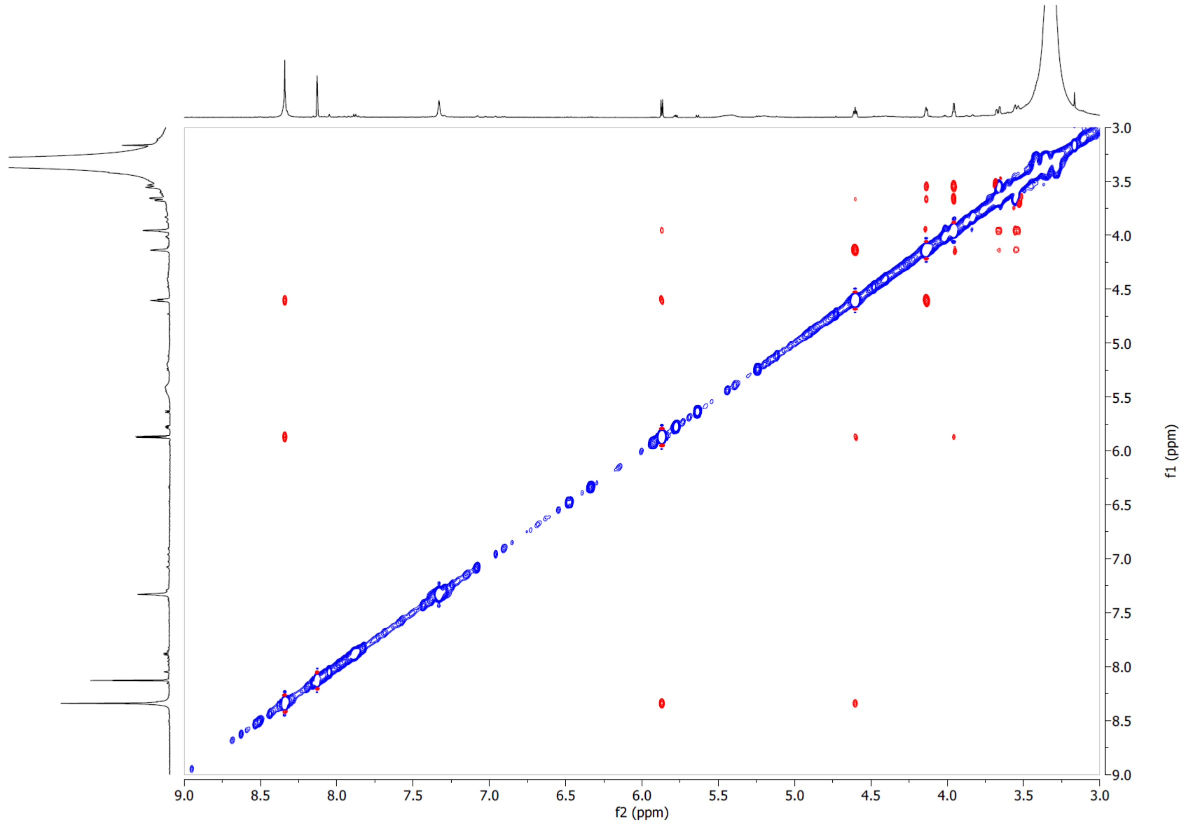


Figure S9: ROESY NMR spectrum of compound **1** in DMSO-$d_6$

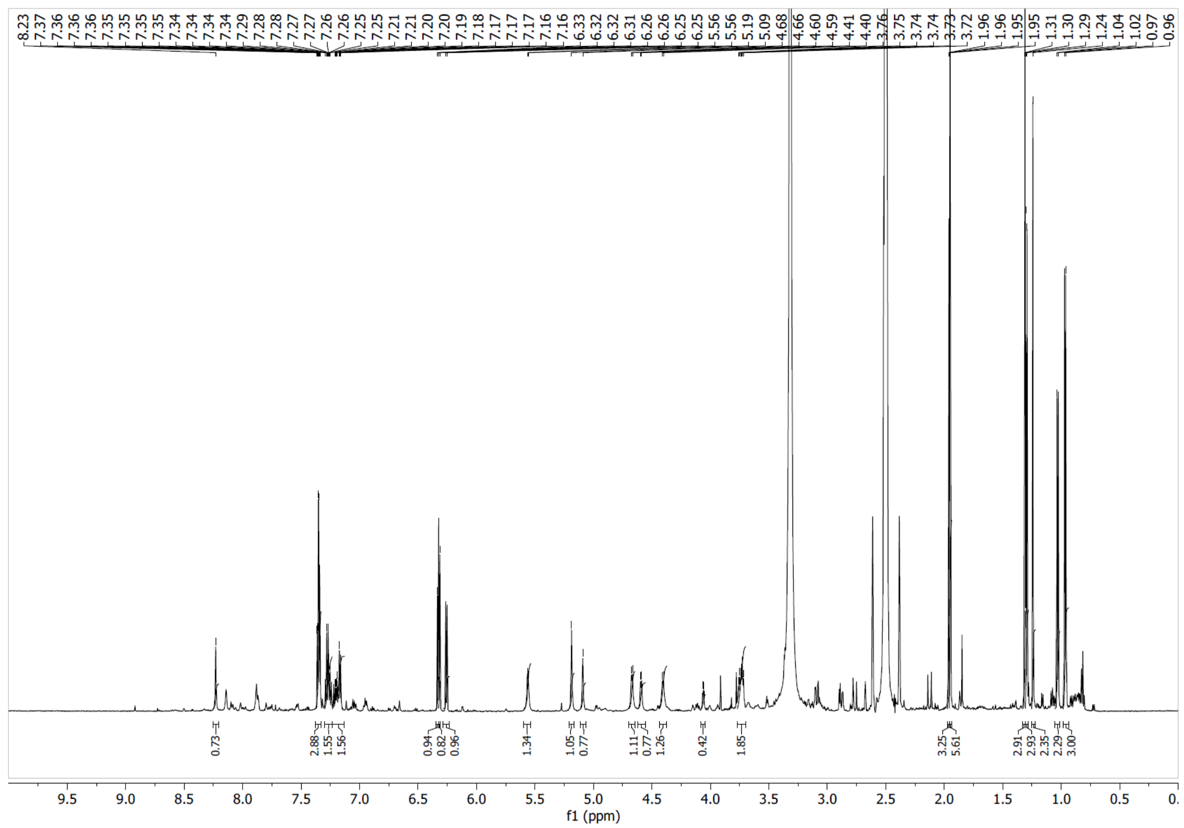


Figure S10: $^{1}$H NMR spectrum of compound **2a**, **2b** and **3** in DMSO-$d_6$ at 600 MHz

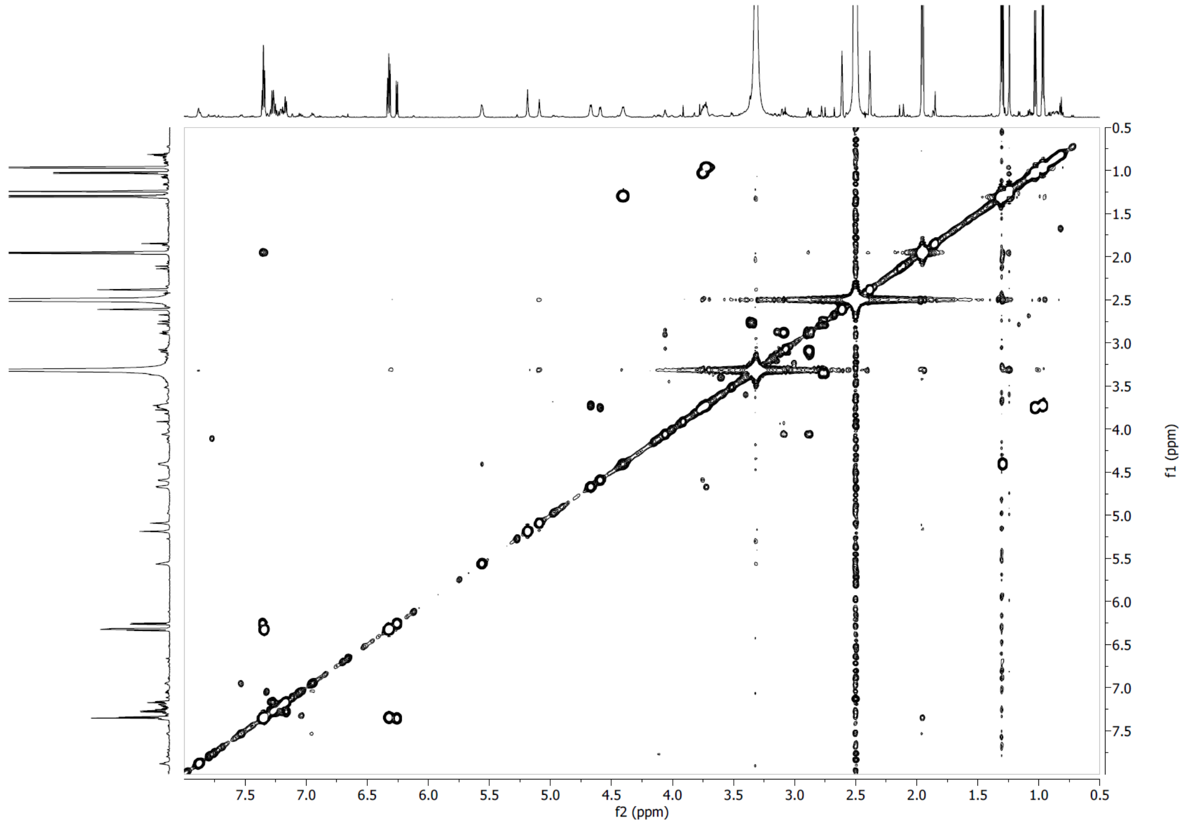

Figure S11: COSY NMR spectrum of compound **2a**, **2b** and **3** in DMSO-$d_6$

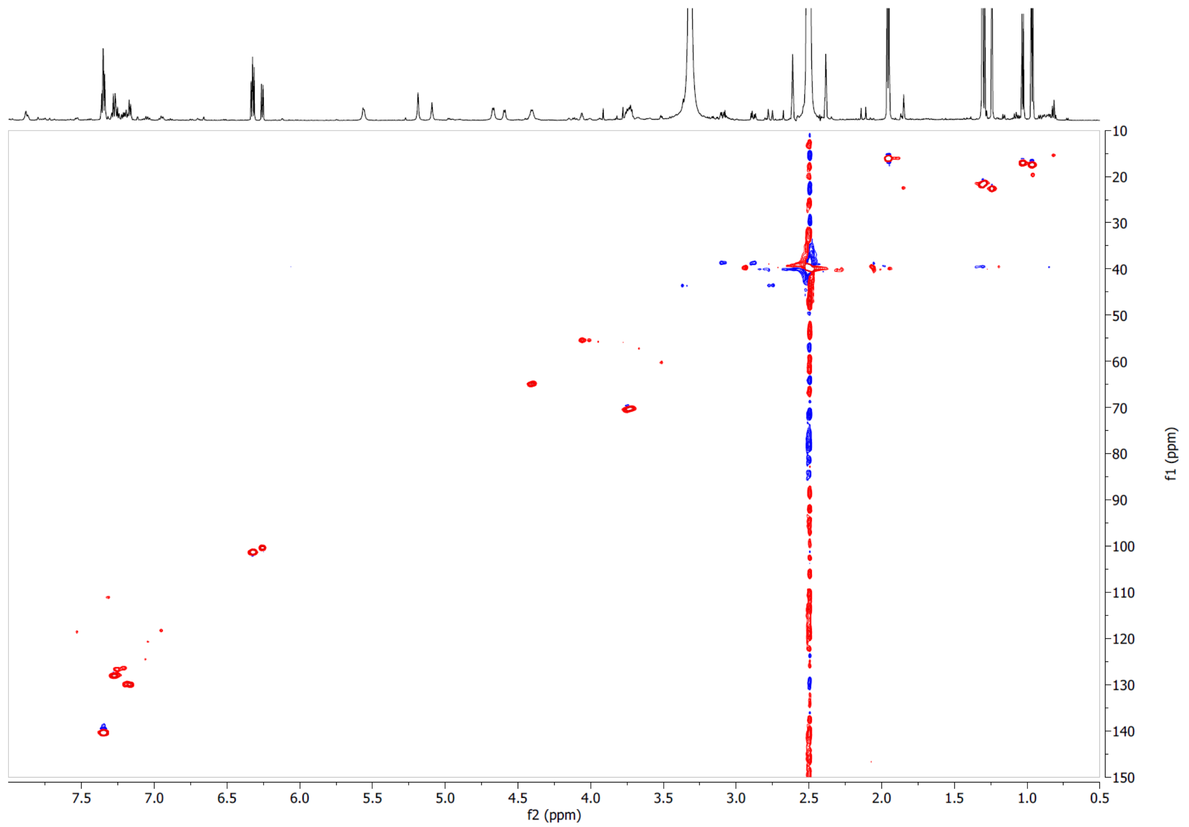


Figure S12: Edited-HSQC NMR spectrum of compound **2a**, **2b** and **3** in DMSO-$d_6$

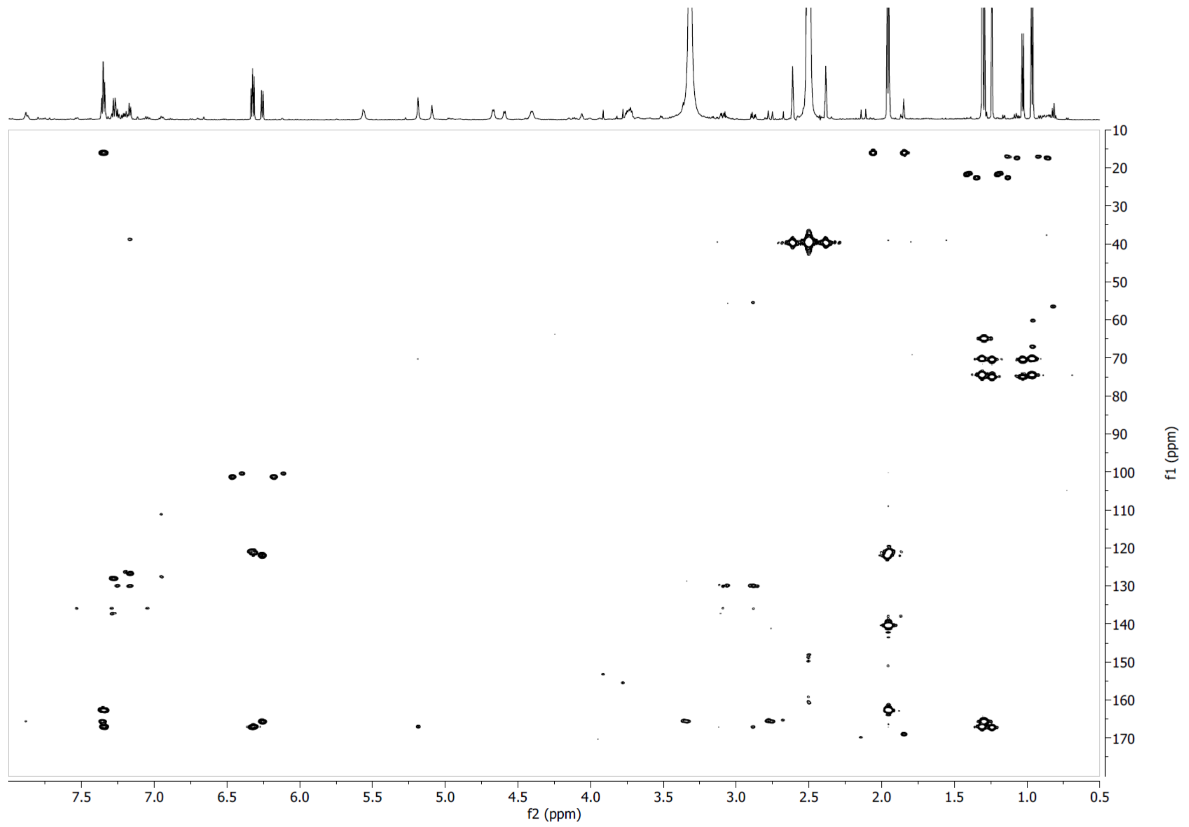

Figure S13: HMBC NMR spectrum of compound **2a**, **2b** and **3** in DMSO-$d_6$

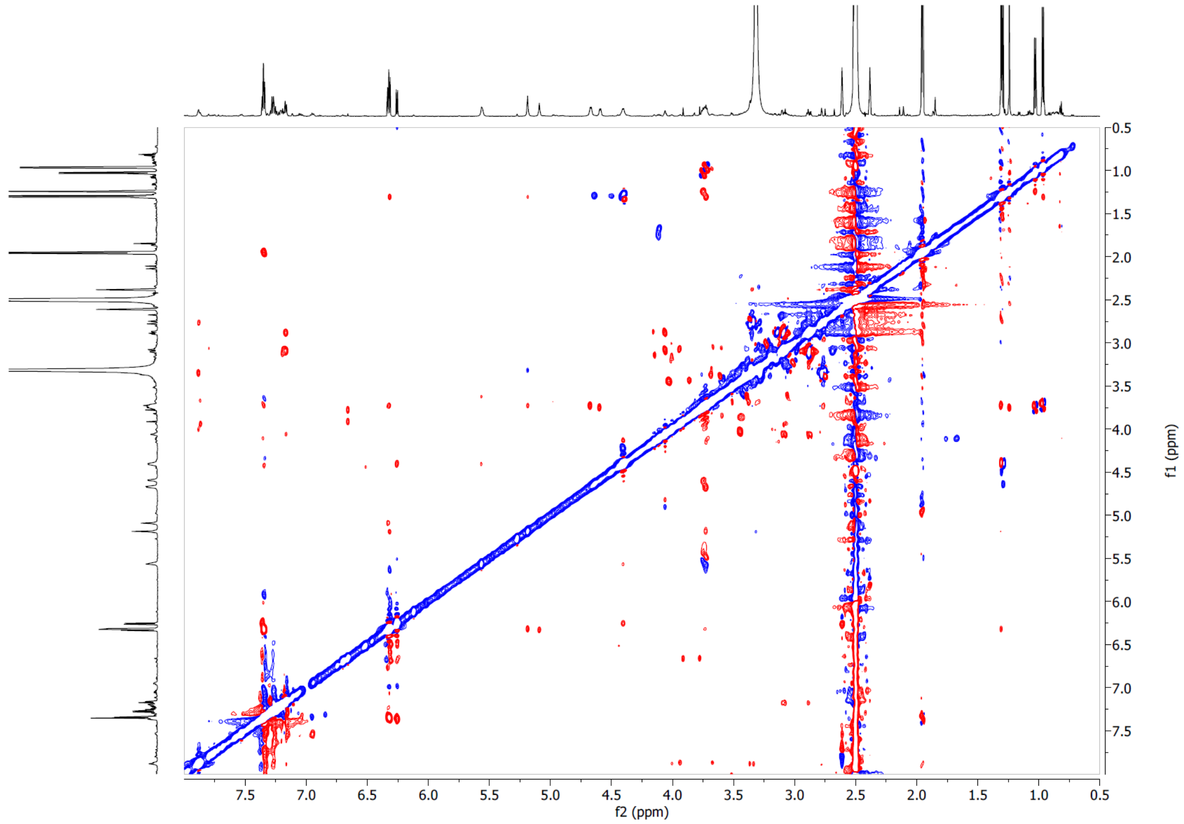


Figure S14: ROESY NMR spectrum of compound **2a**, **2b** and **3** in DMSO-$d_6$

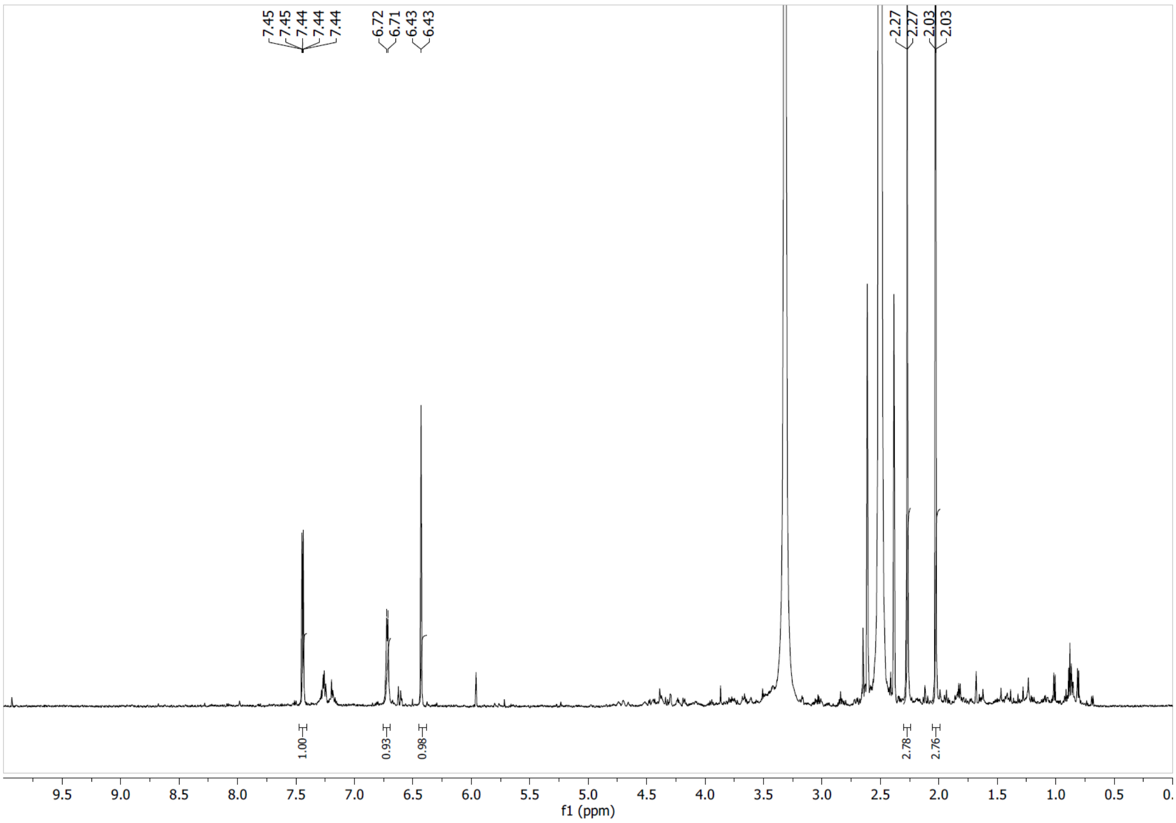

Figure S15: $^{1}$H NMR spectrum of compound **4** in DMSO-$d_6$ at 600 MHz

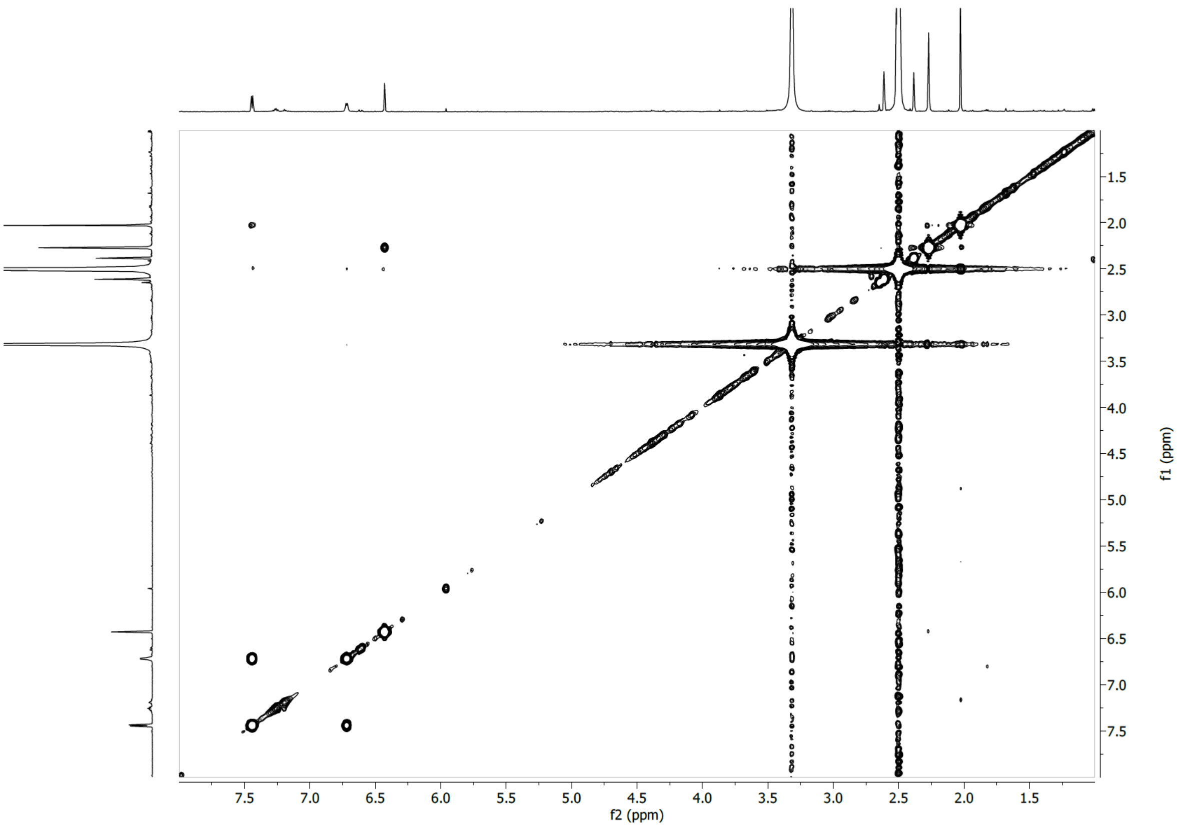


Figure S16: COSY NMR spectrum of compound **4** in DMSO-$d_6$

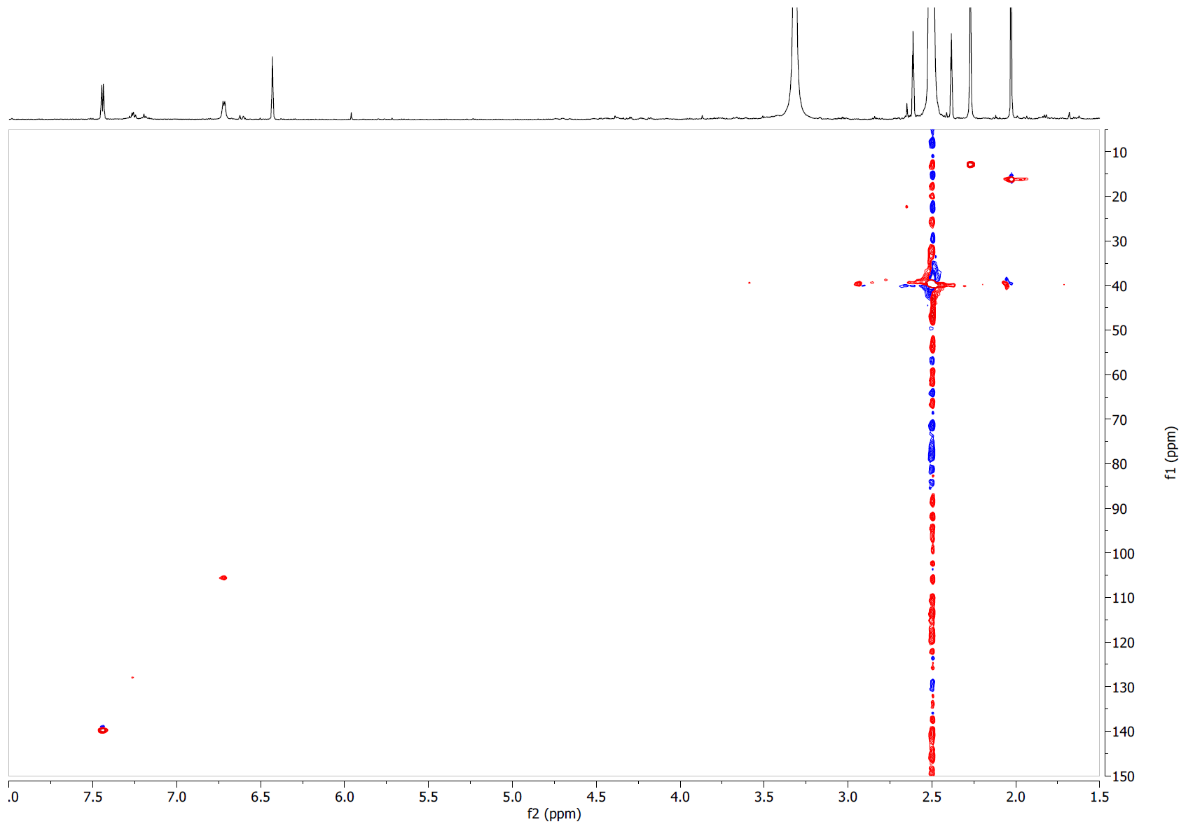

Figure S17: Edited-HSQC NMR spectrum of compound **4** in DMSO-$d_6$

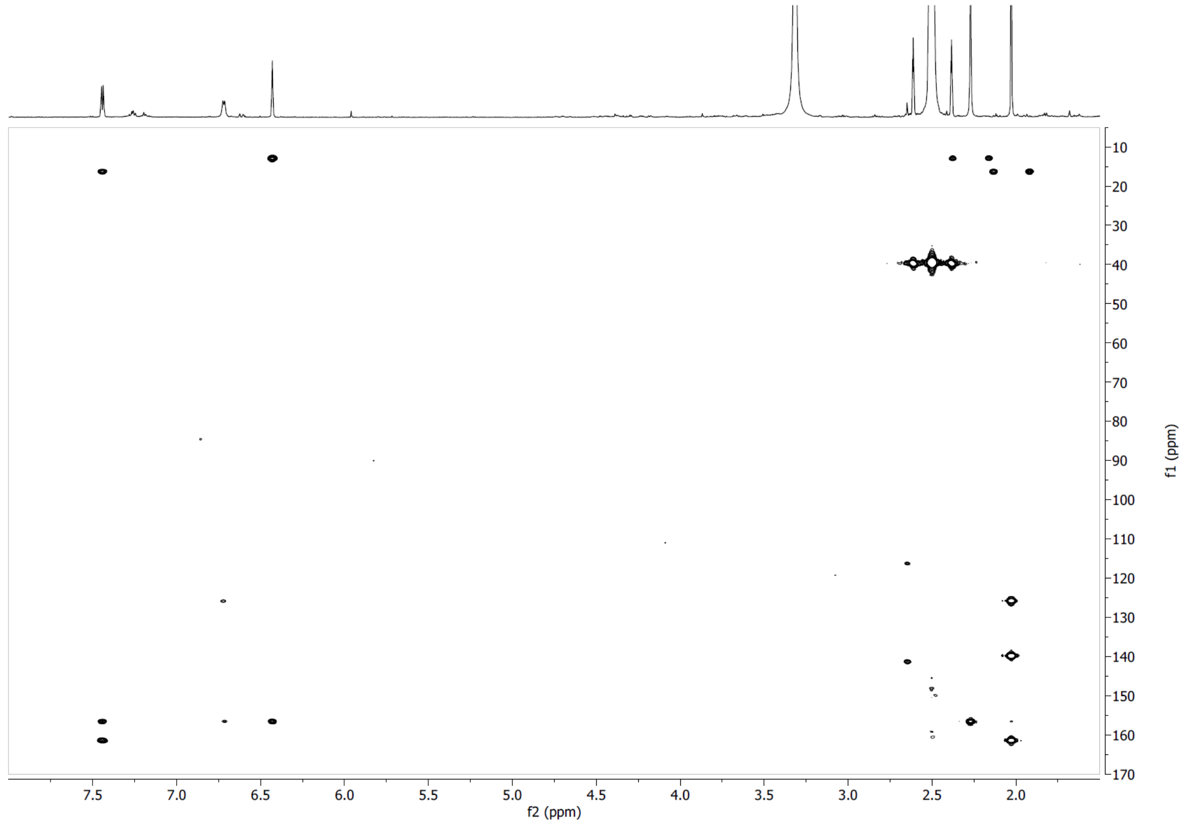


Figure S18: HMBC NMR spectrum of compound **4** in DMSO-$d_6$

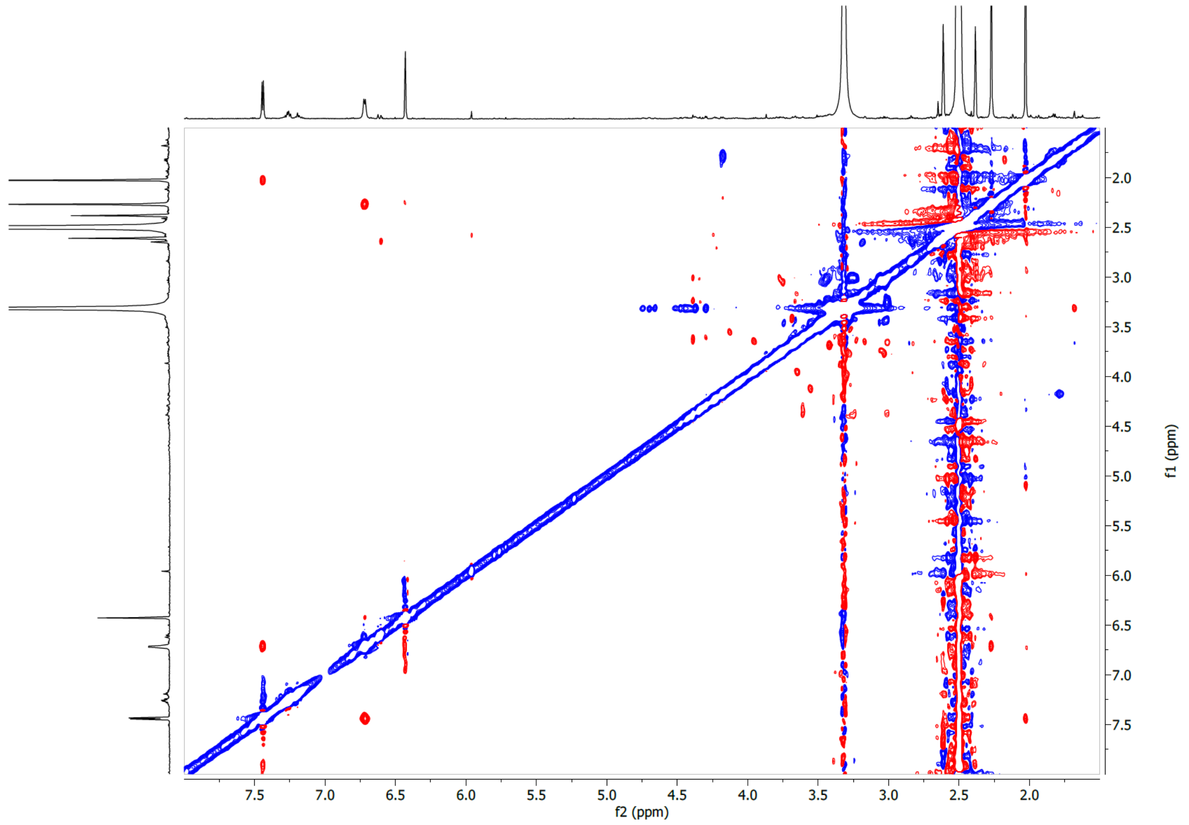

Figure S19: ROESY NMR spectrum of compound **4** in DMSO-$d_6$

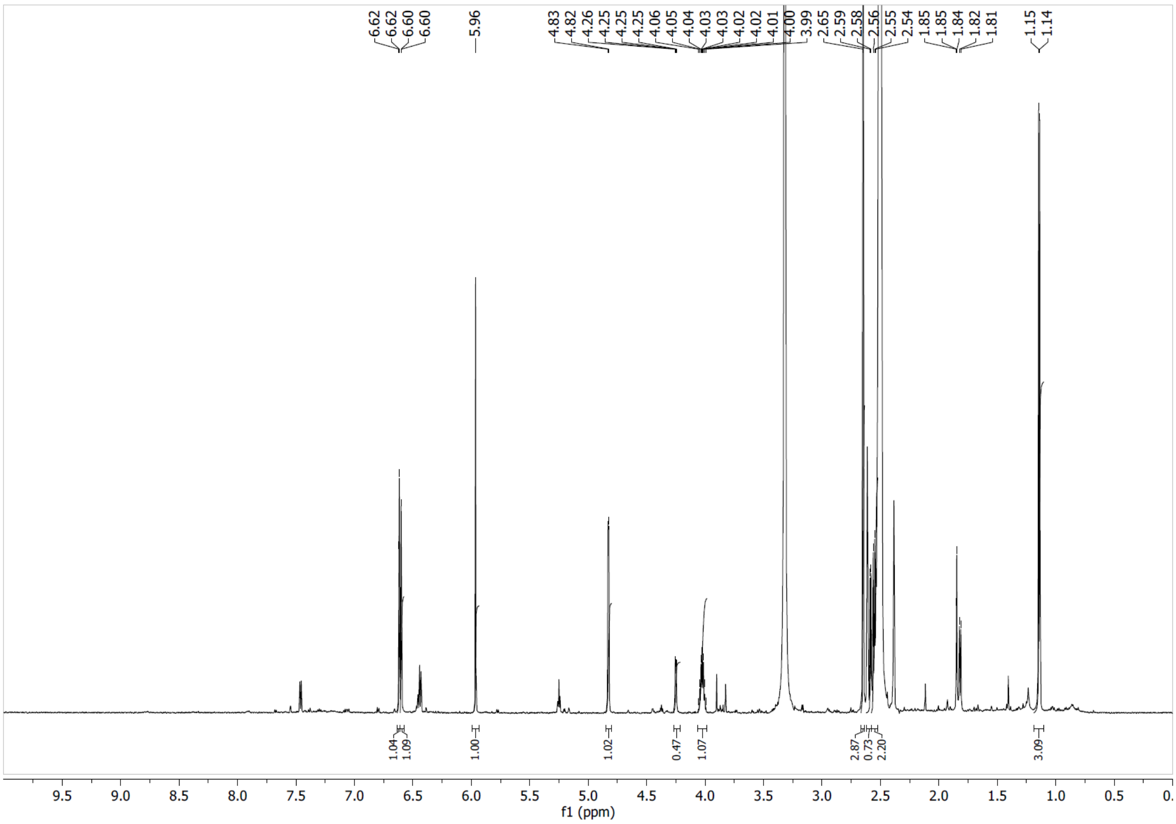


Figure S20: $^{1}$H NMR spectrum of compound **5** in DMSO-$d_6$ at 600 MHz

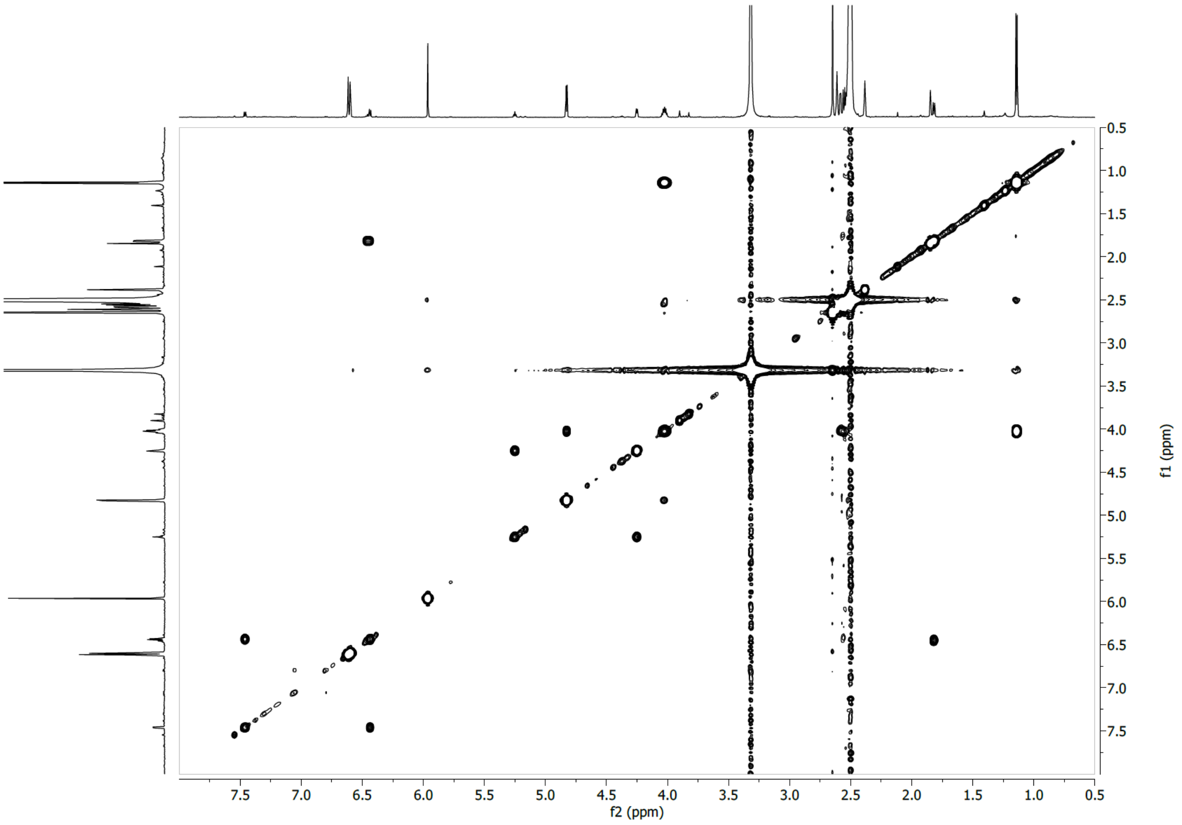

Figure S21: COSY NMR spectrum of compound **5** in DMSO-$d_6$

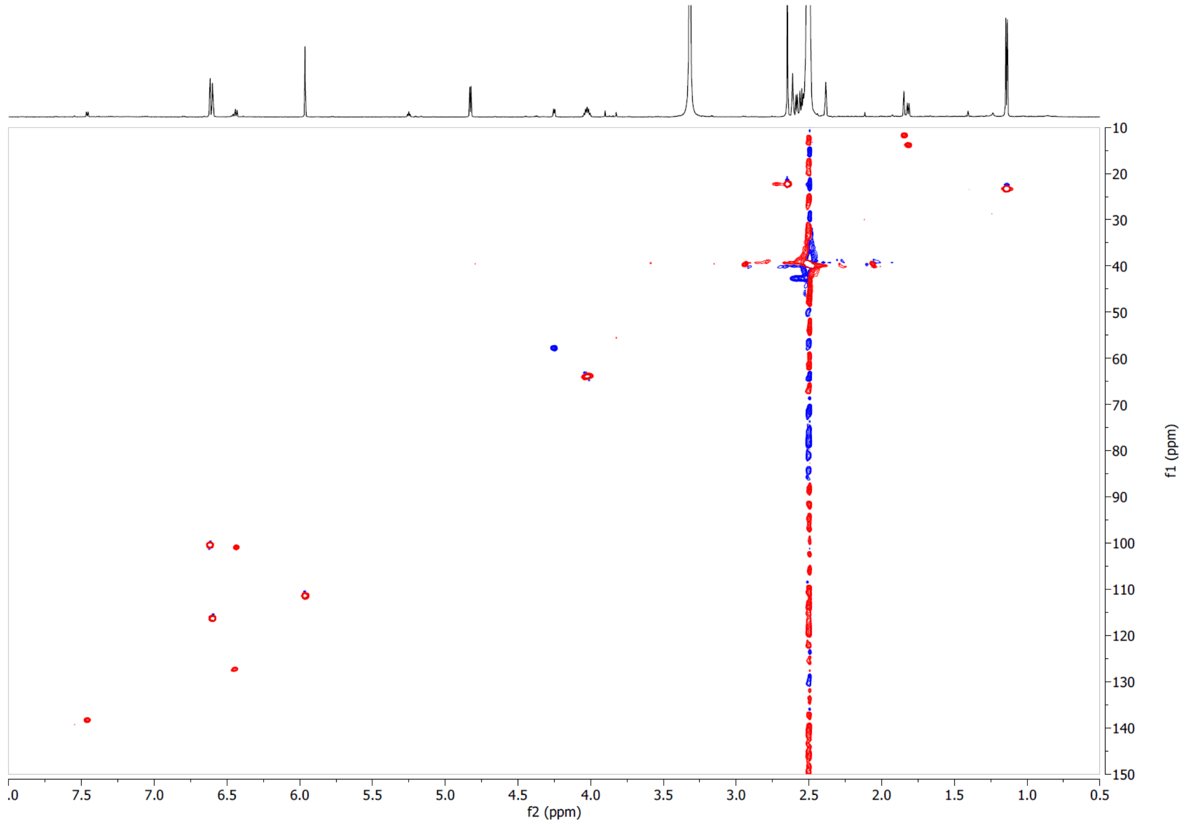


Figure S22: Edited-HSQC NMR spectrum of compound **5** in DMSO-$d_6$

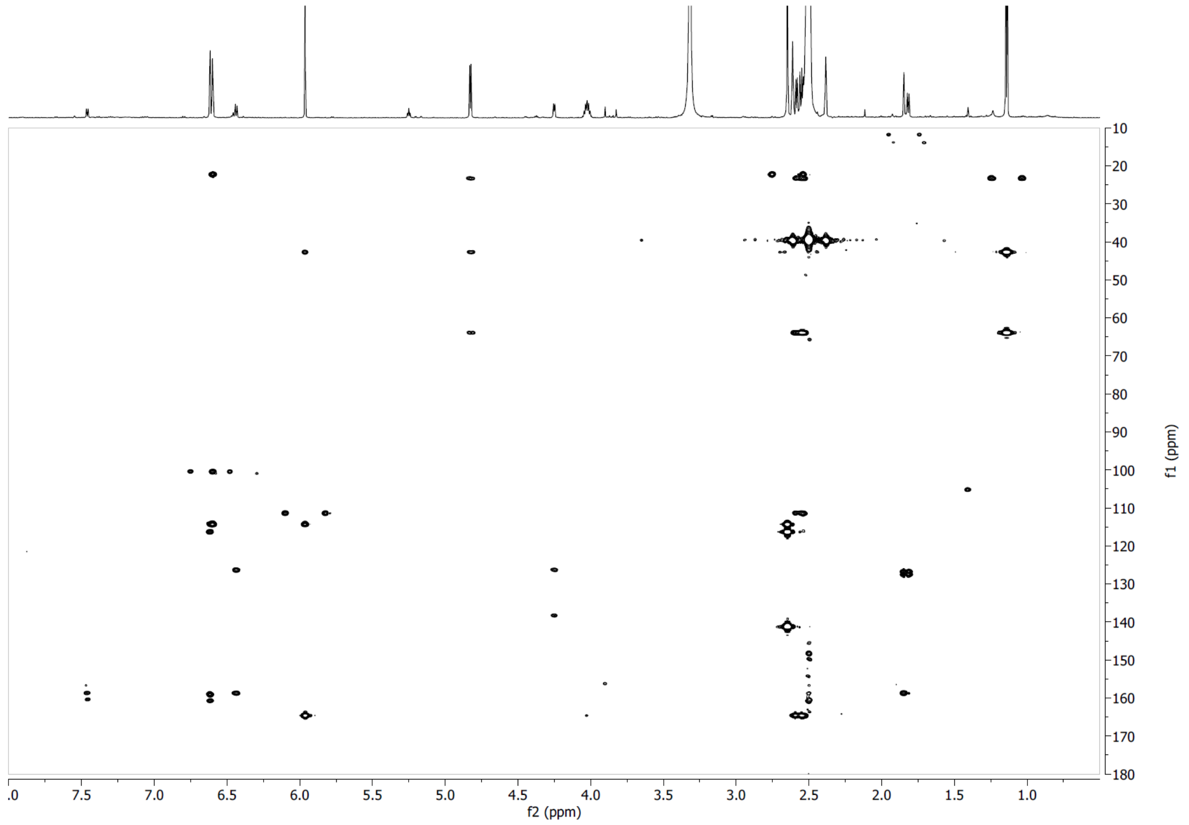

Figure S23: HMBC NMR spectrum of compound **5** in DMSO-$d_6$

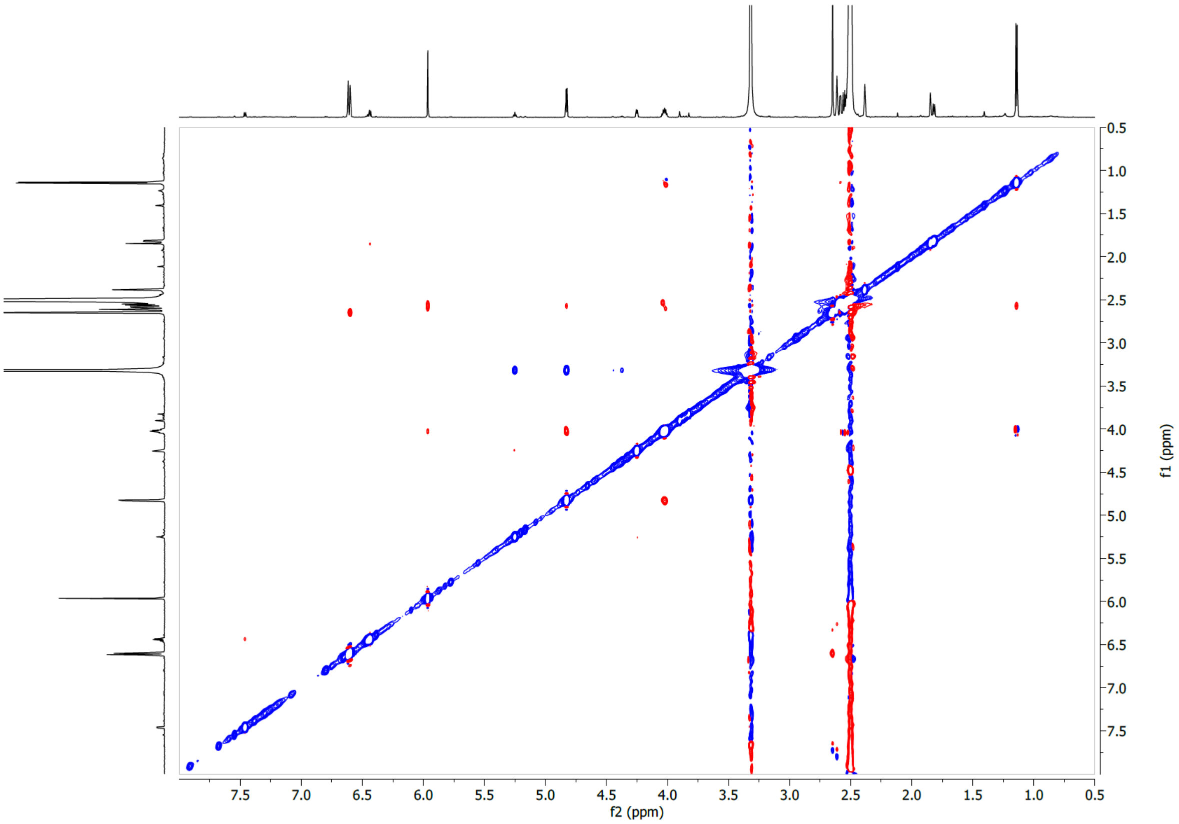


Figure S24: ROESY NMR spectrum of compound **5** in DMSO-$d_6$

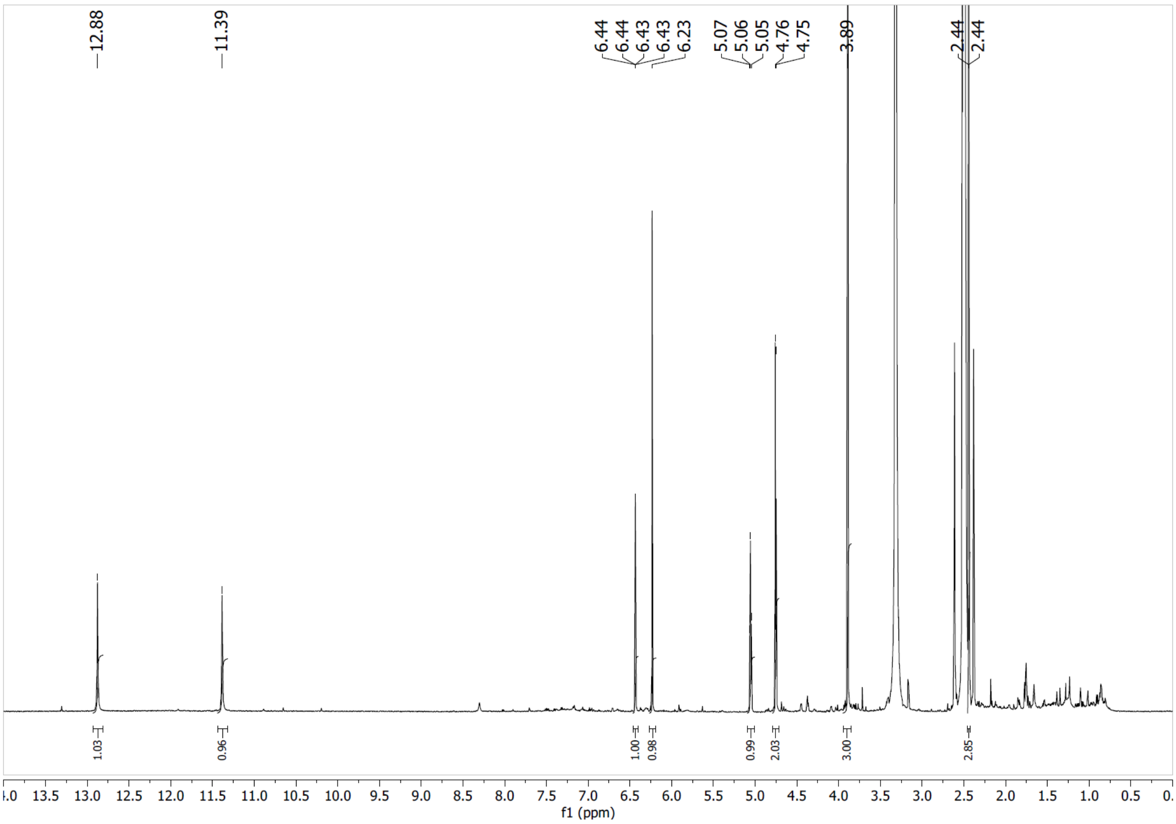

Figure S25: $^{1}$H NMR spectrum of compound **6** in DMSO-$d_6$

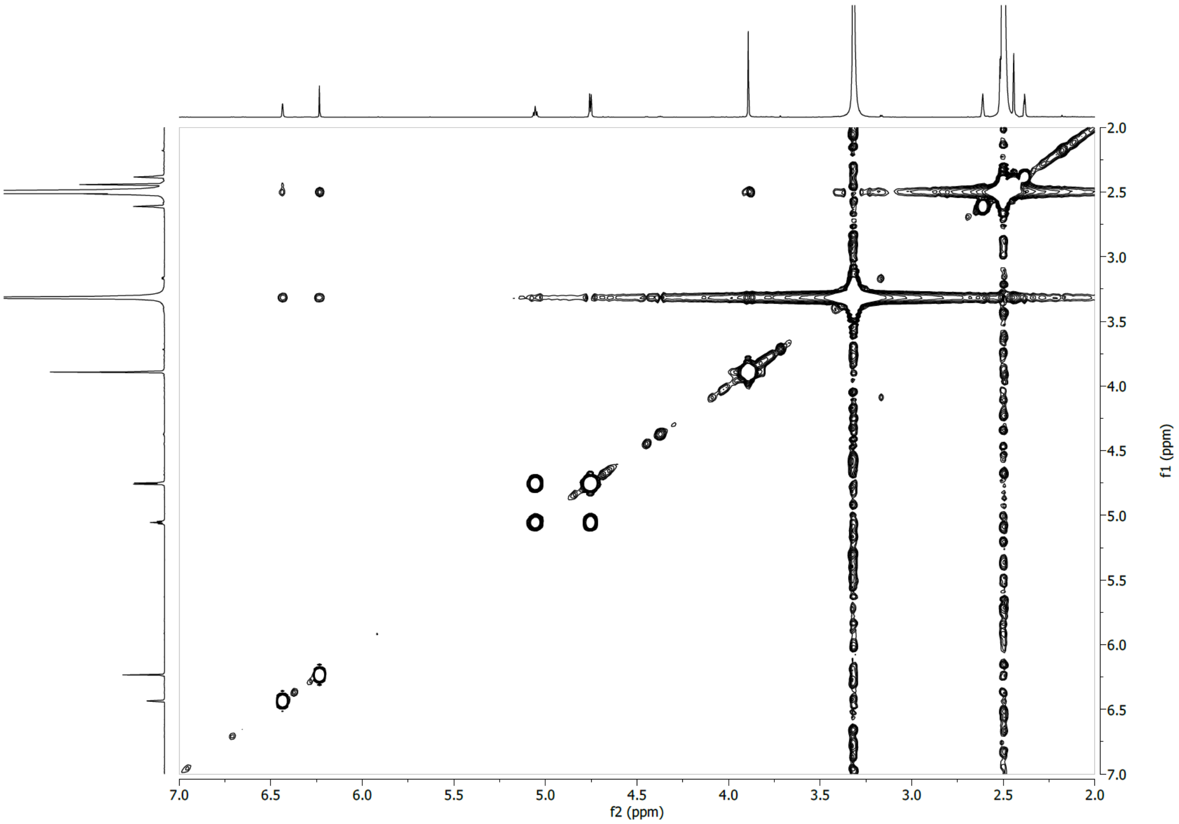


Figure S26: COSY NMR spectrum of compound **6** in DMSO-$d_6$

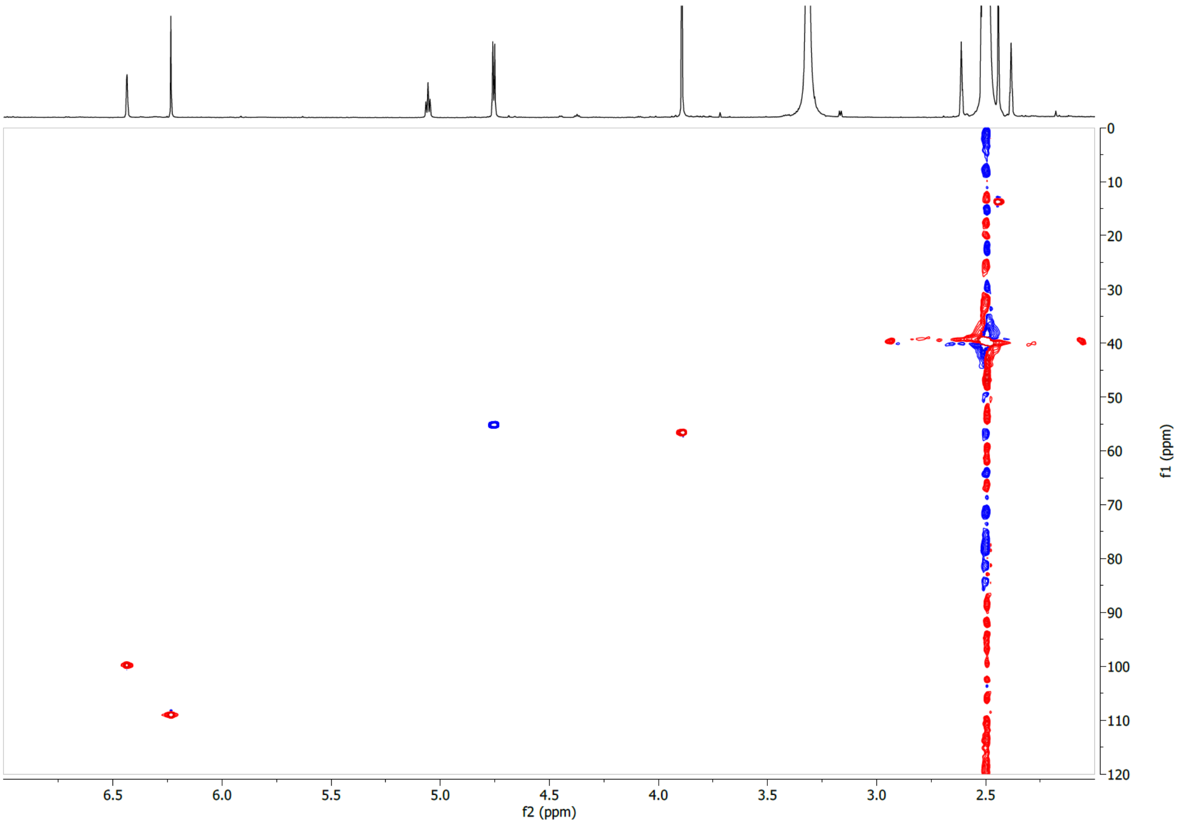

Figure S27: Edited-HSQC NMR spectrum of compound **6** in DMSO-$d_6$

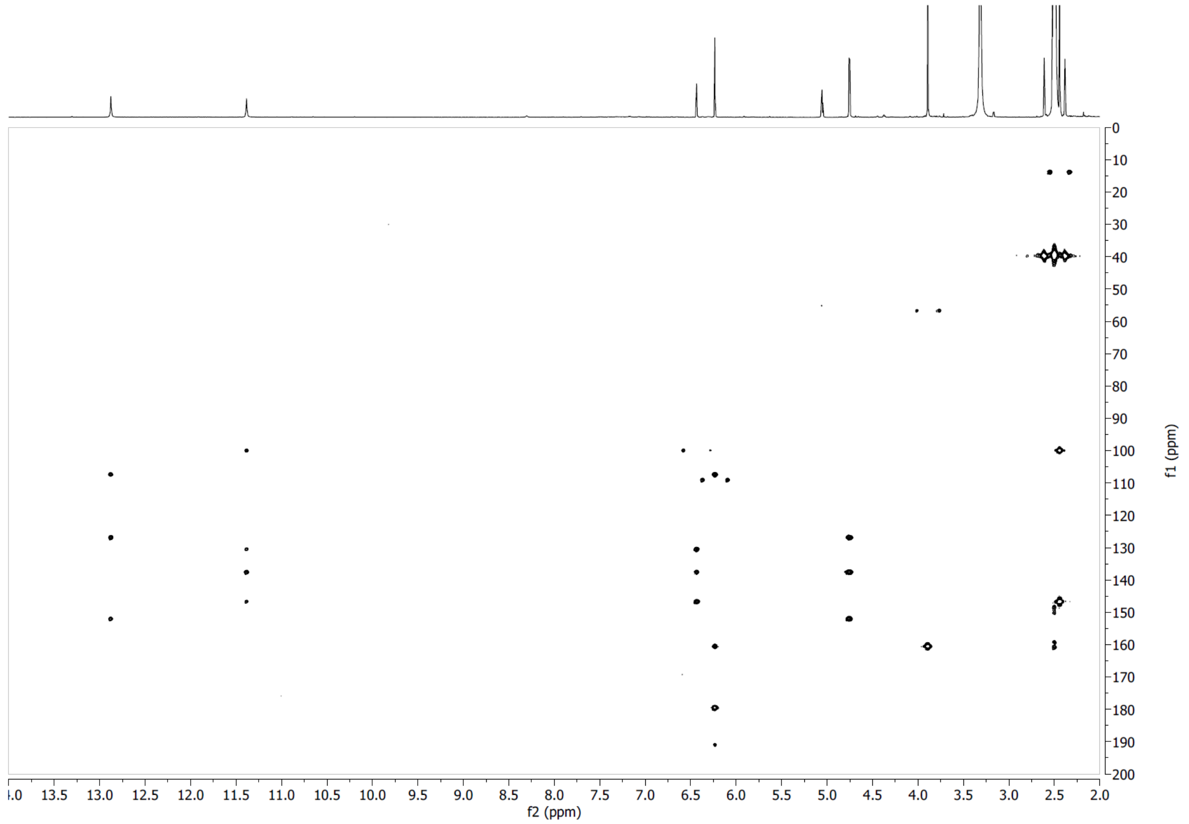


Figure S28: HMBC NMR spectrum of compound **6** in DMSO-$d_6$

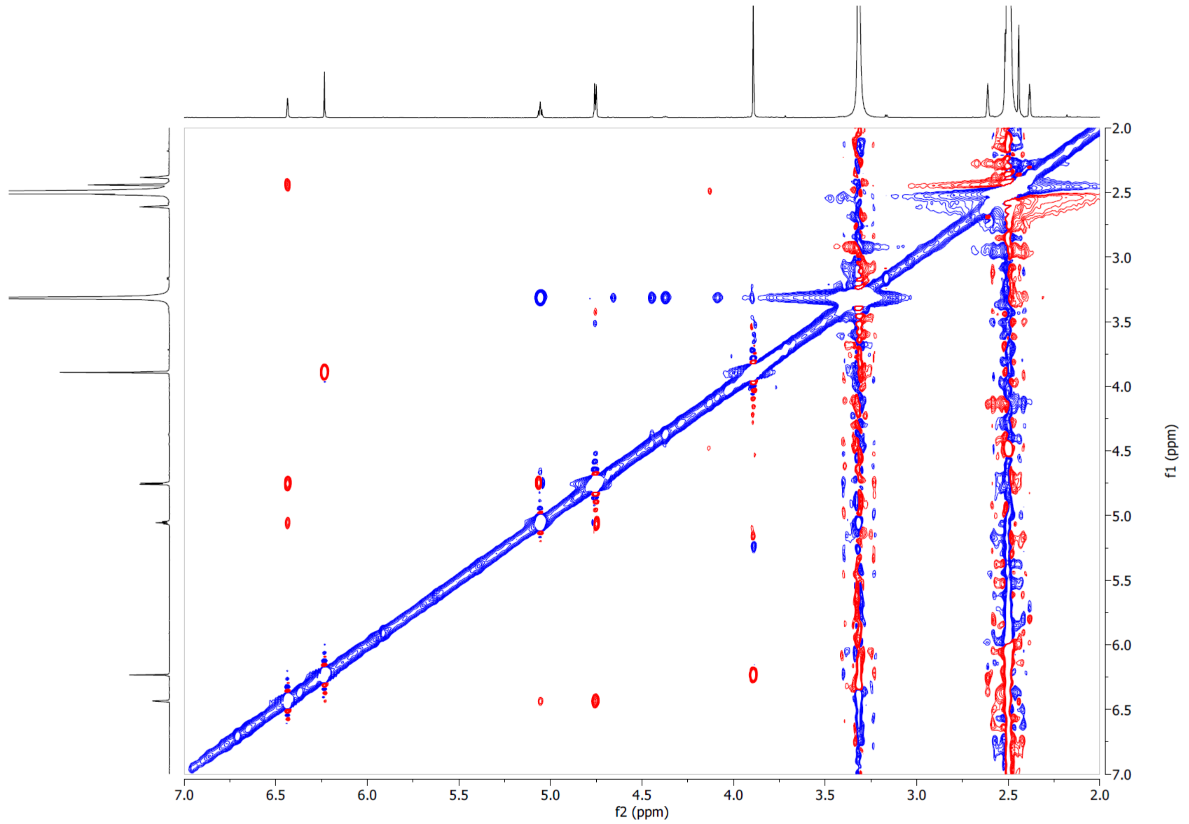

Figure S29: ROESY NMR spectrum of compound **6** in DMSO-$d_6$

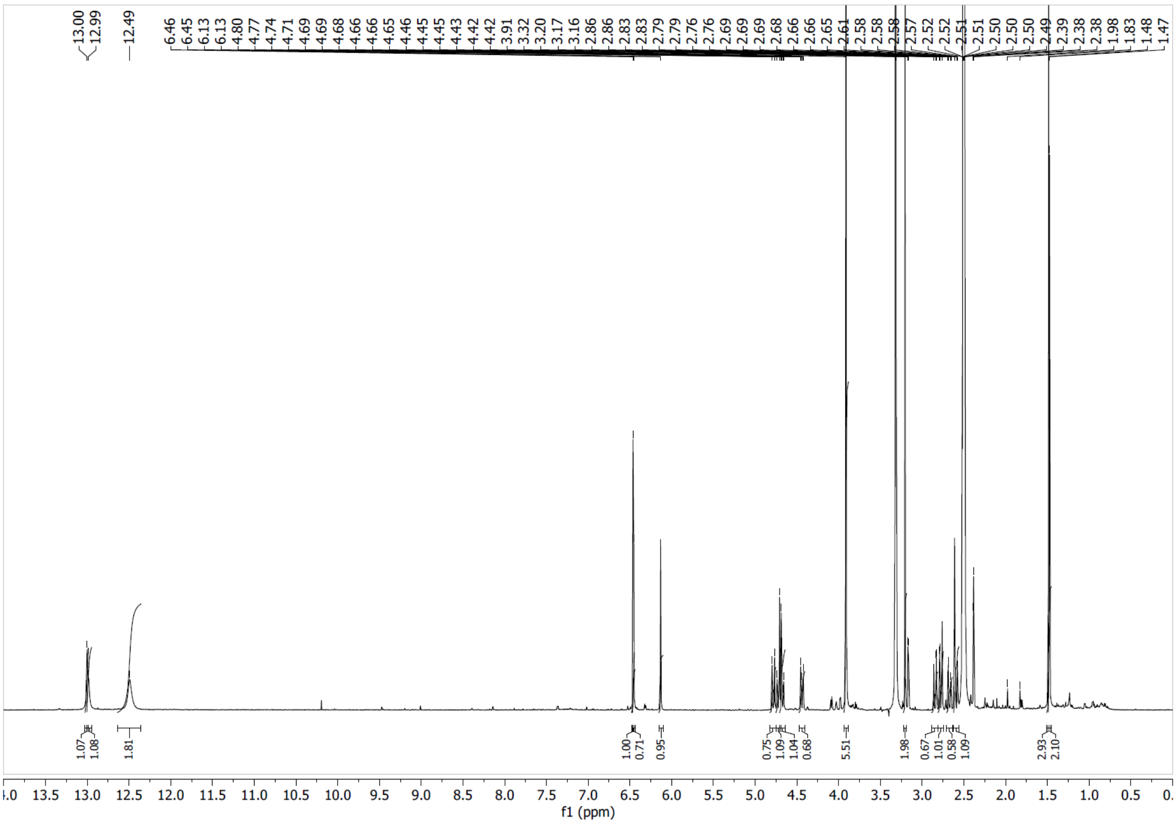


Figure S30: $^{1}$H NMR spectrum of compound **7** et **8** in DMSO-$d_6$ at 600 MHz

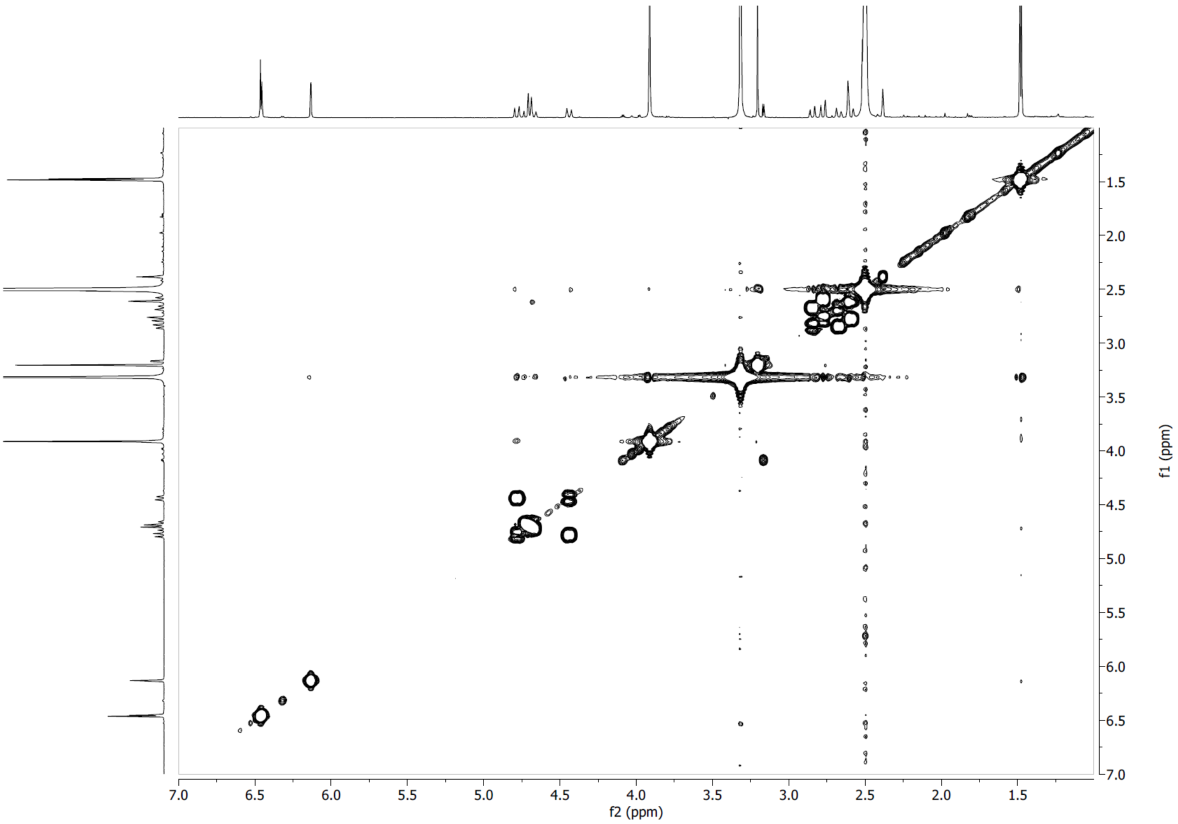

Figure S31: COSY NMR spectrum of compound **7** et **8** in DMSO-$d_6$

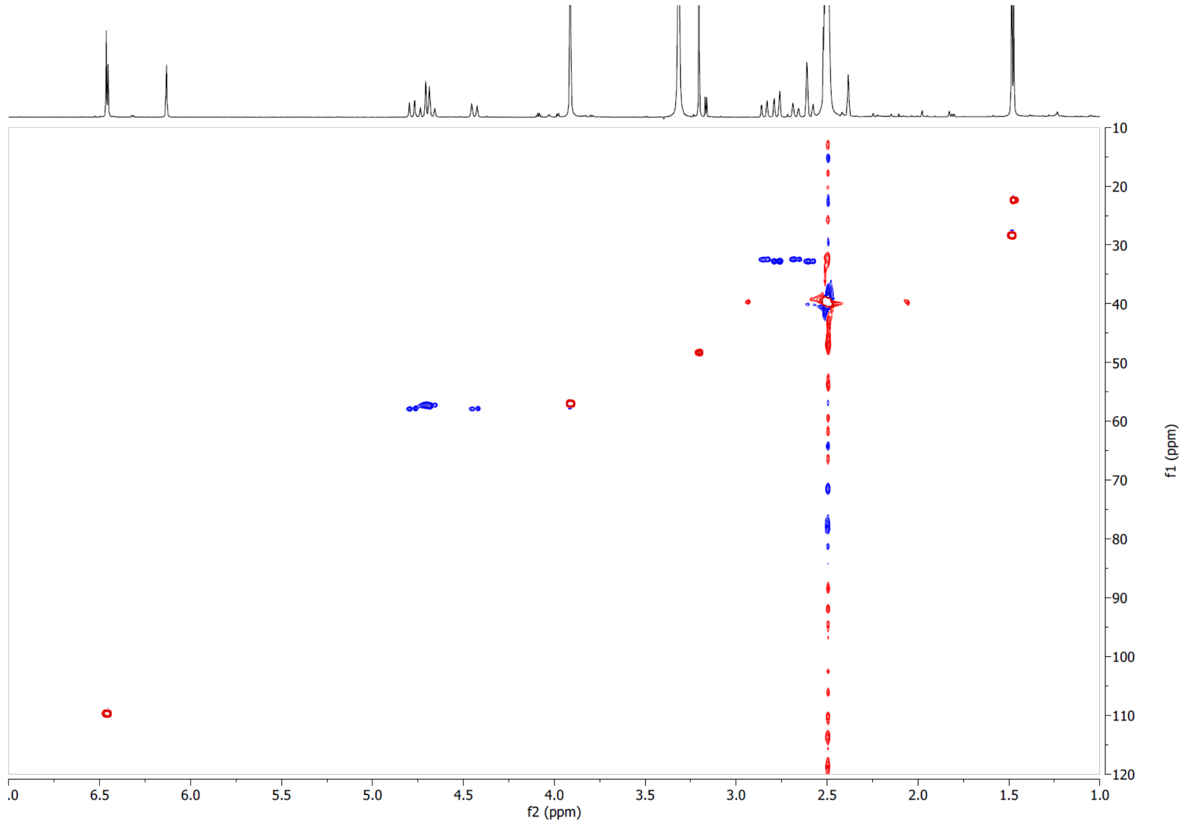


Figure S32: Edited-HSQC NMR spectrum of compound **7** et **8** in DMSO-$d_6$

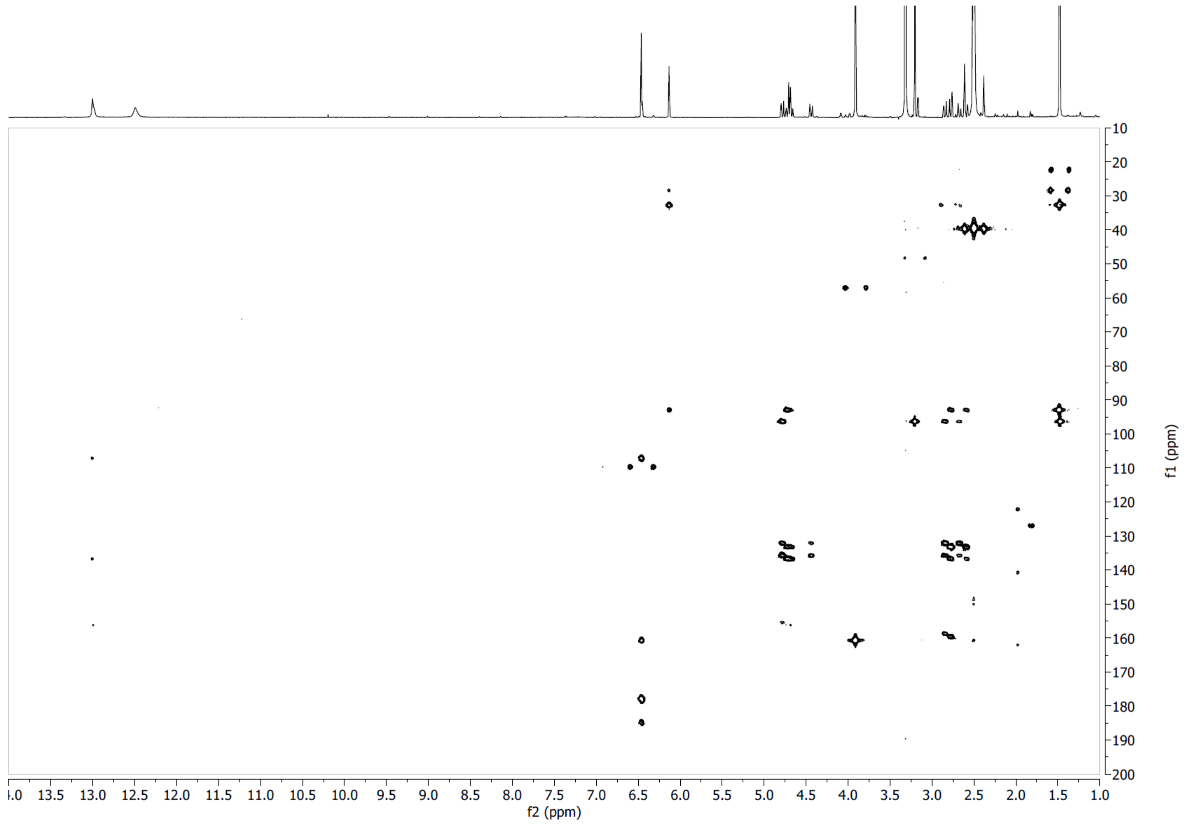

Figure S33: HMBC NMR spectrum of compound **7** et **8** in DMSO-$d_6$

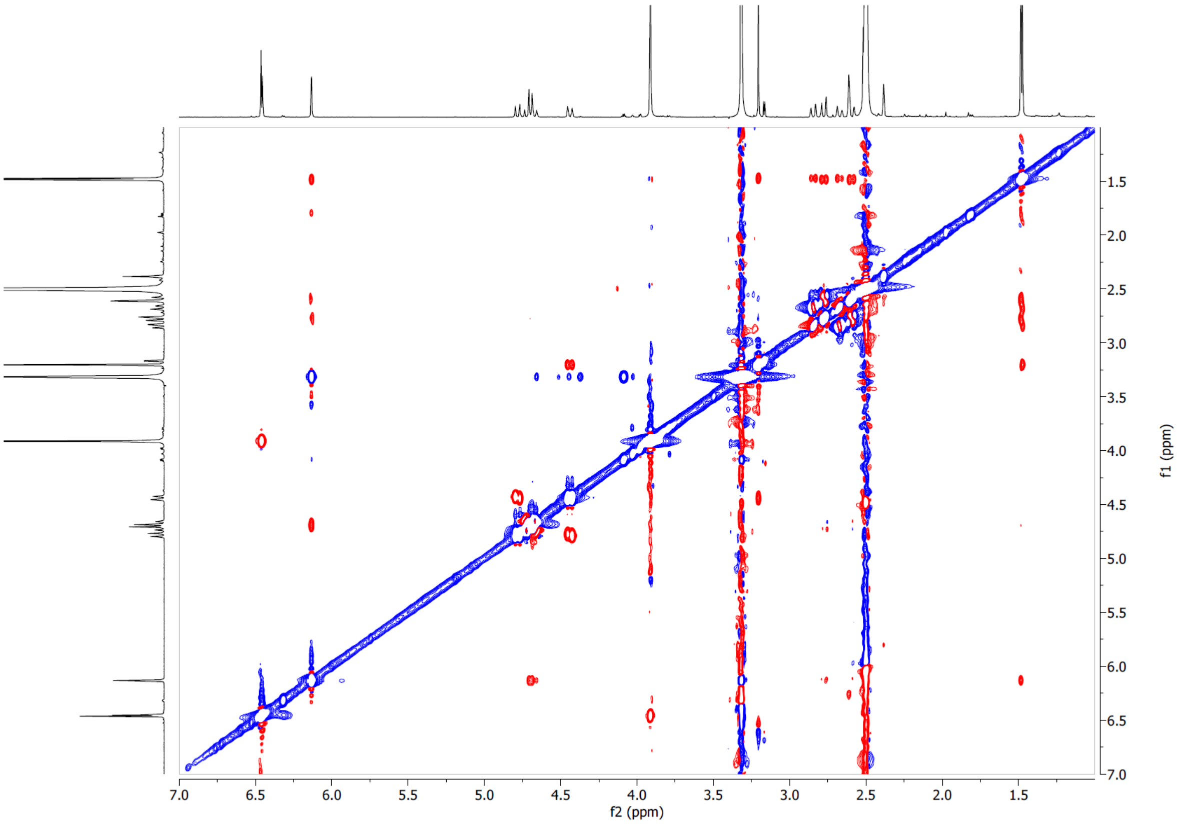


Figure S34: ROESY NMR spectrum of compound **7** et **8** in DMSO-$d_6$

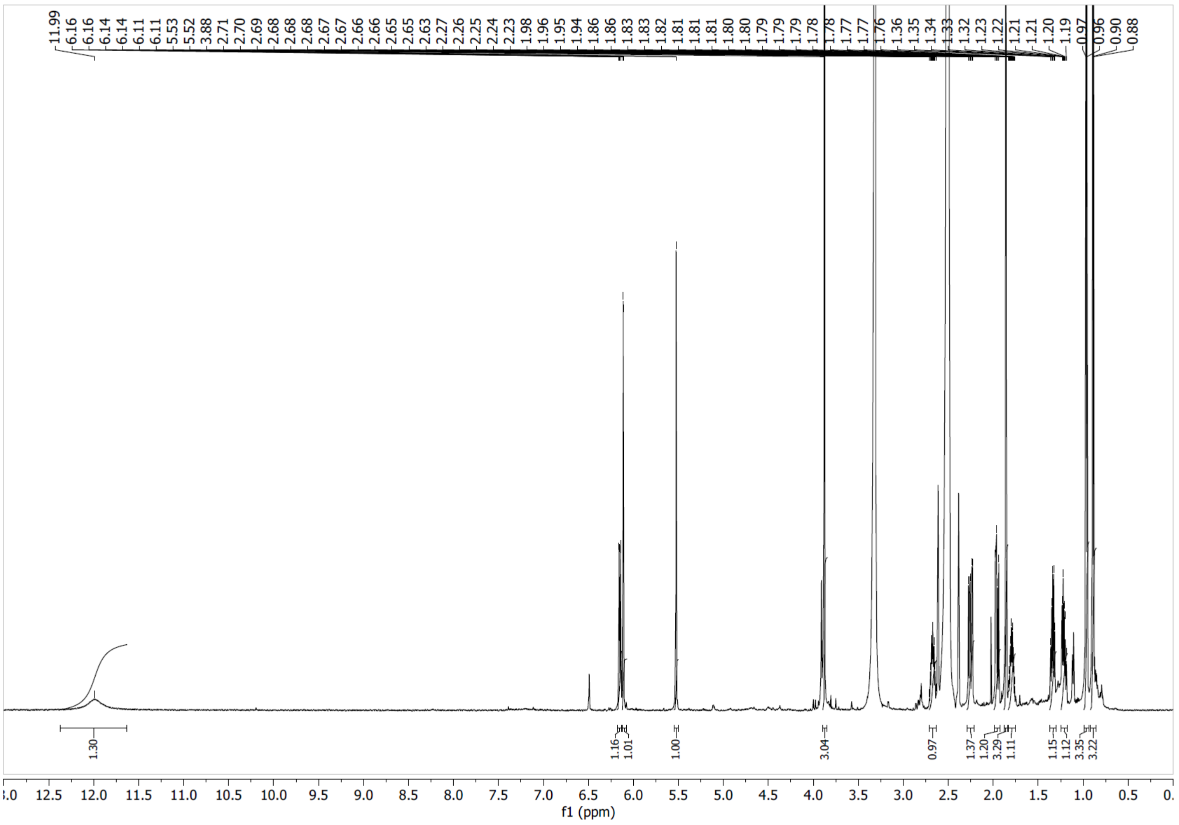

Figure S35: $^{1}$H NMR spectrum of compound **9** in DMSO-$d_6$ at 600 MHz

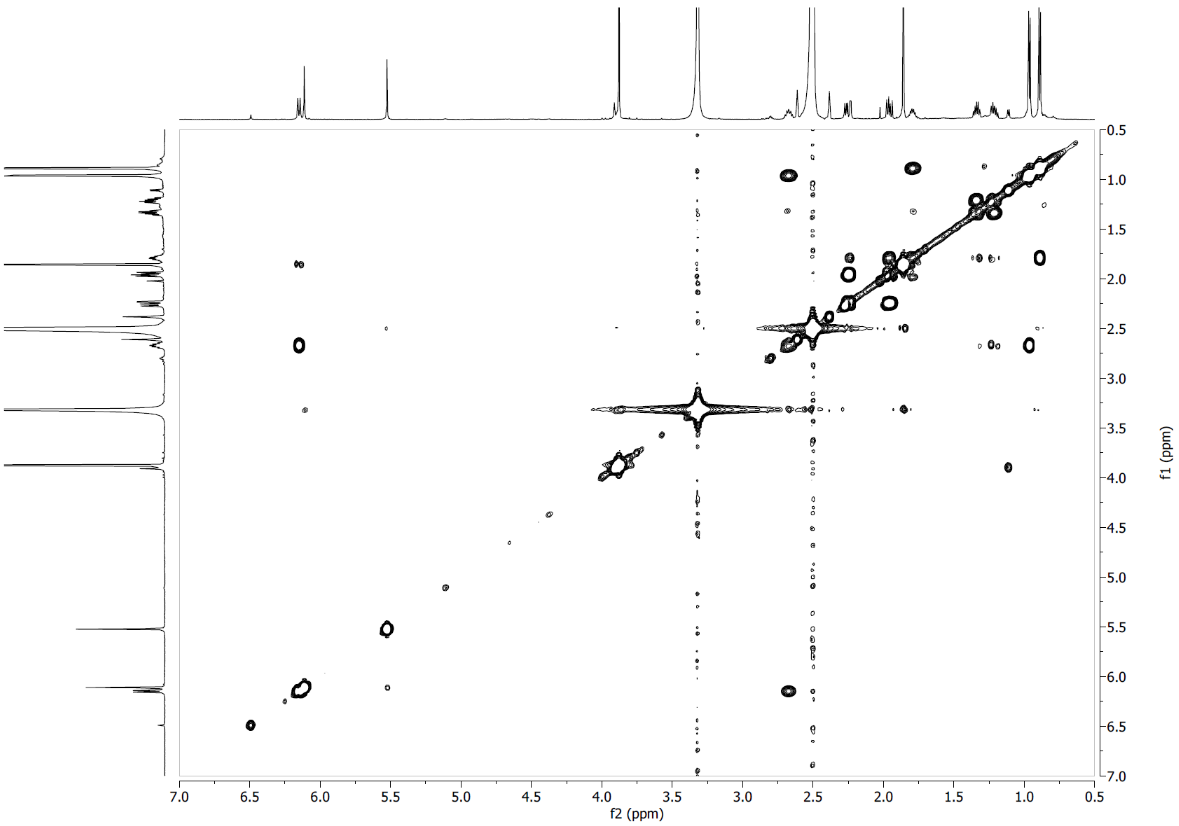


Figure S36: COSY NMR spectrum of compound **9** in DMSO-$d_6$

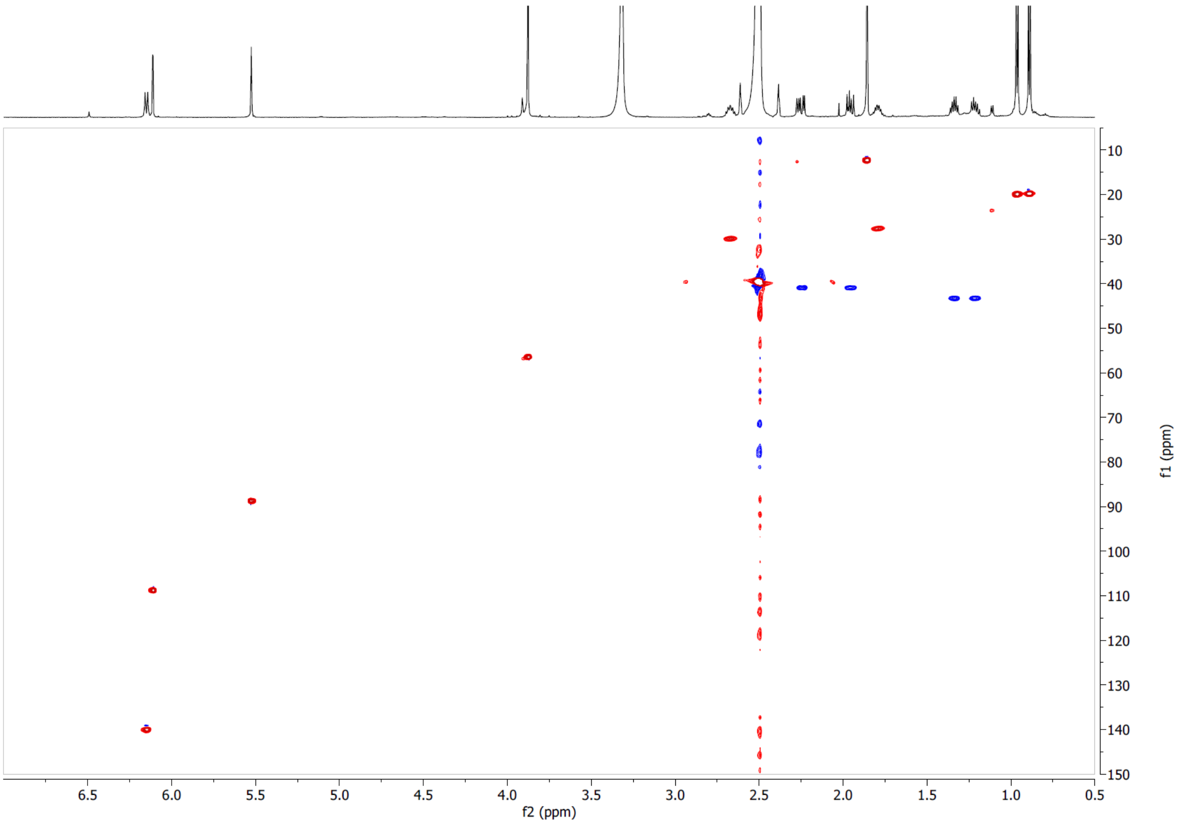

Figure S37: Edited-HSQC NMR spectrum of compound **9** in DMSO-$d_6$

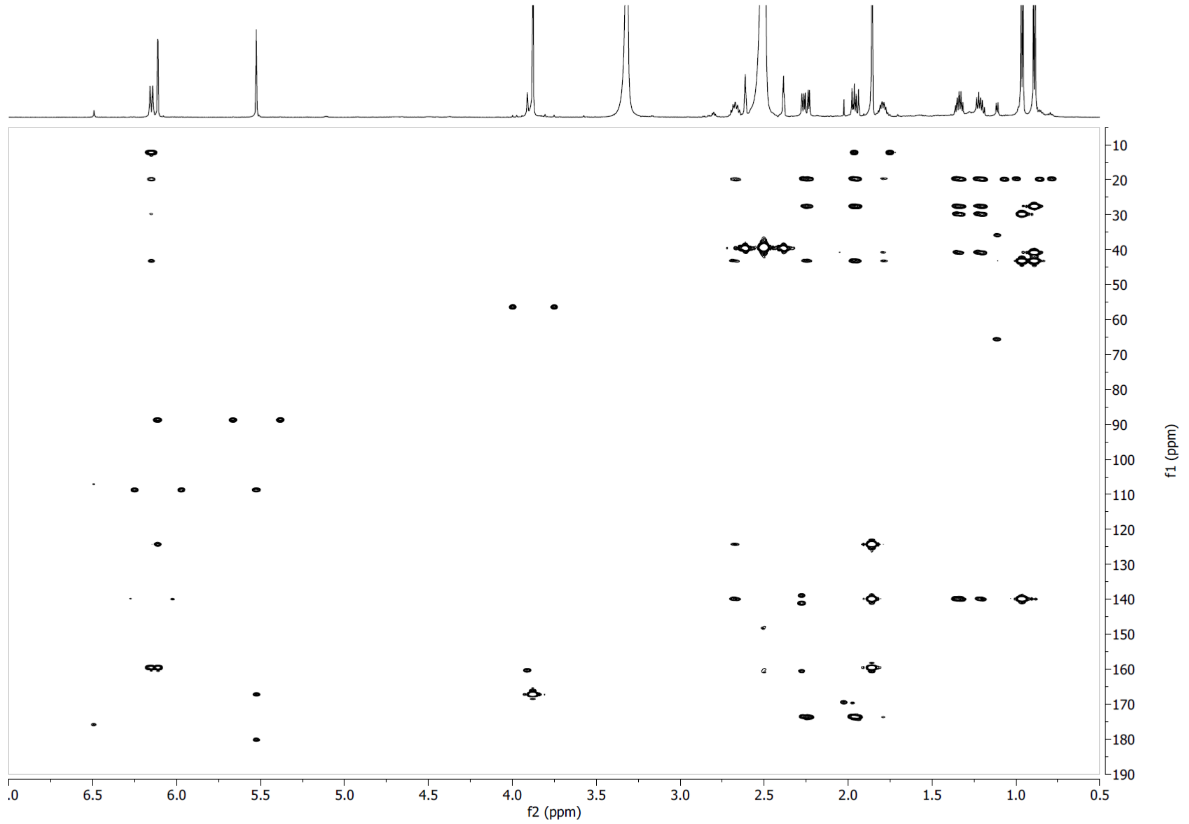


Figure S38: HMBC NMR spectrum of compound **9** in DMSO-$d_6$

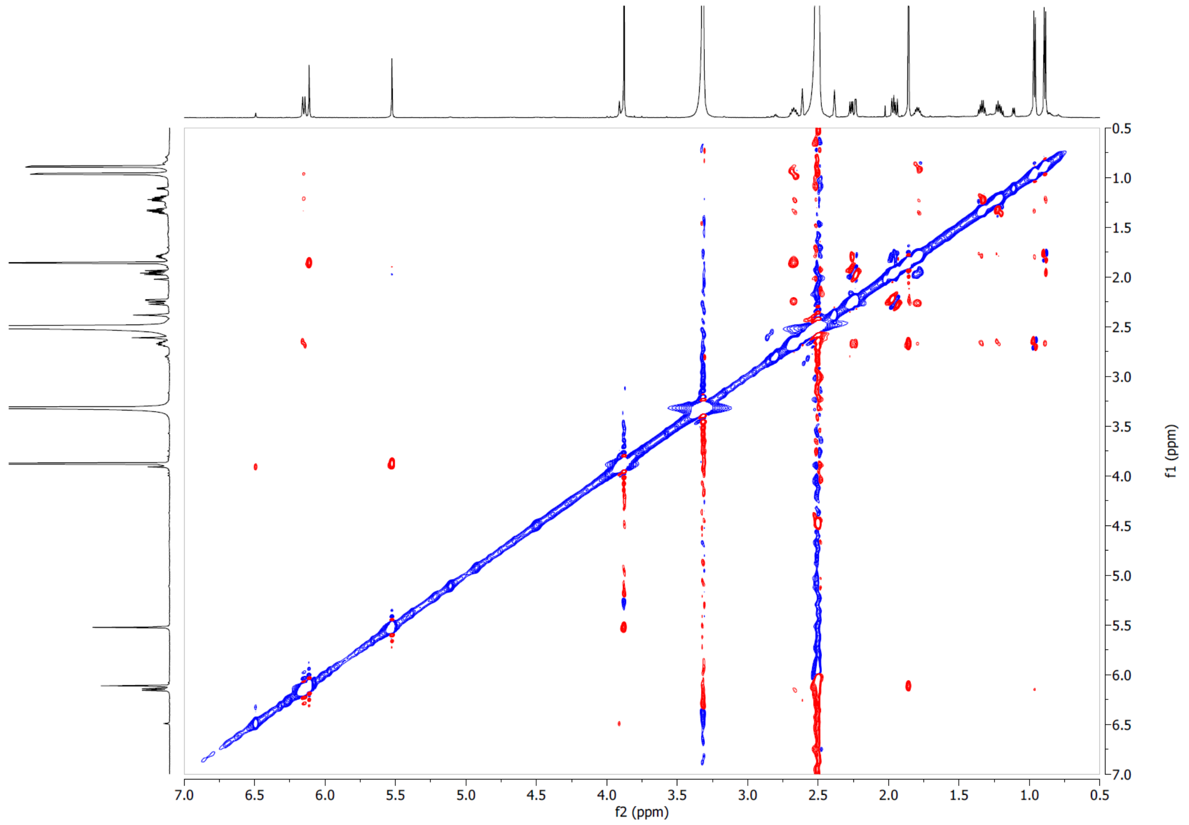

Figure S39: ROESY NMR spectrum of compound **9** in DMSO-$d_6$

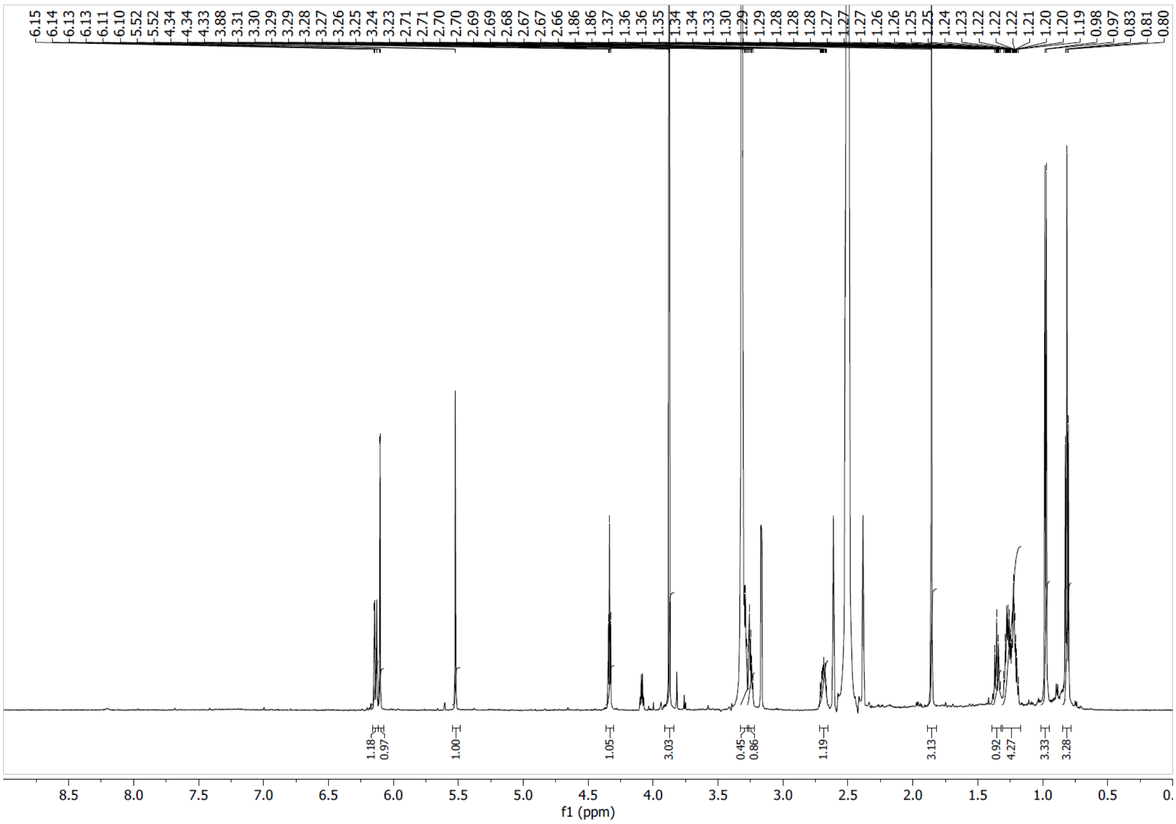


Figure S40: $^{1}$H NMR spectrum of compound **10** in DMSO-$d_6$ at 600 MHz

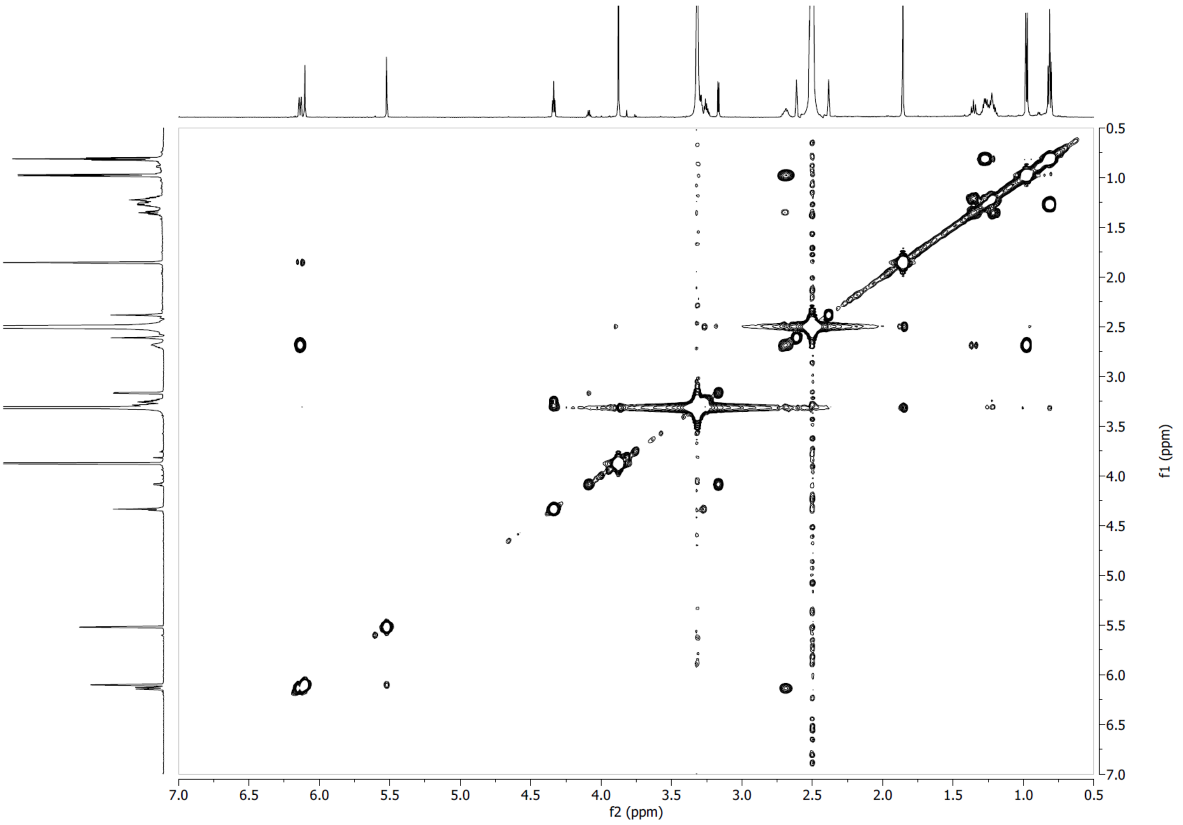

Figure S41: COSY NMR spectrum of compound **10** in DMSO-$d_6$

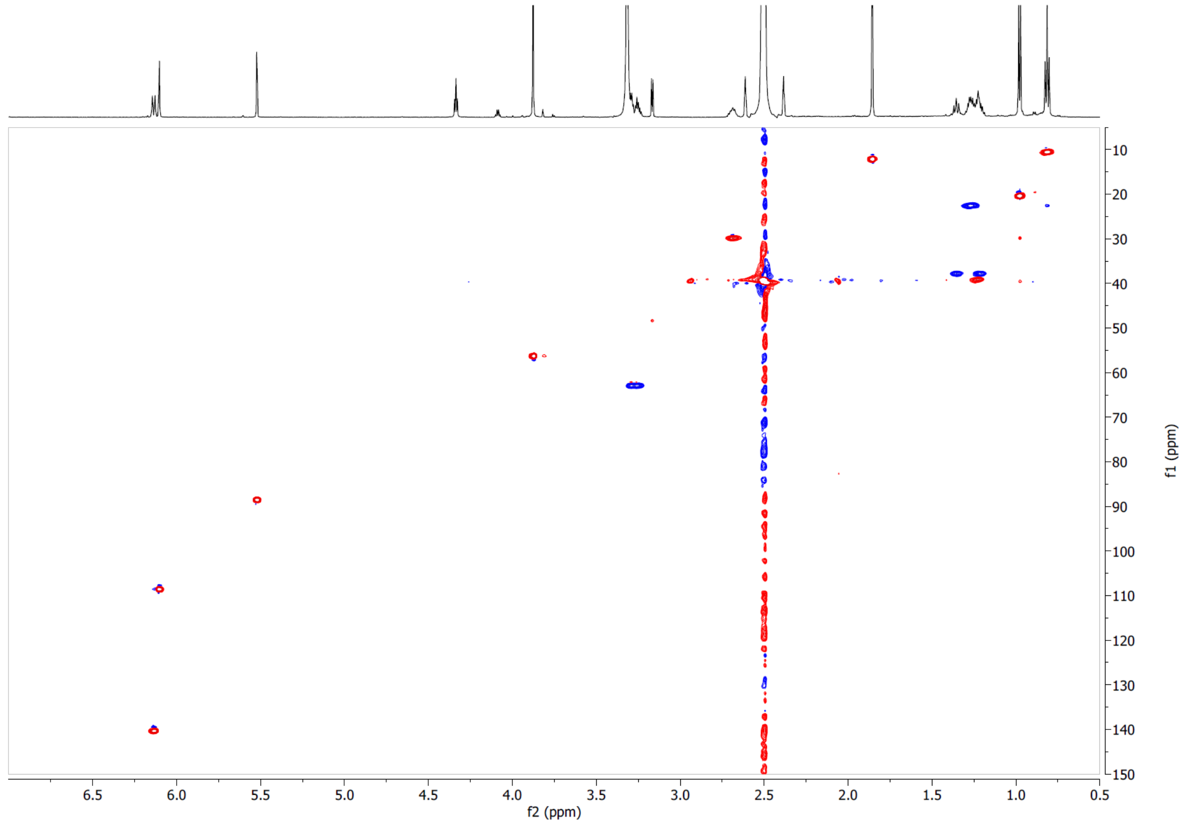


Figure S42: Edited-HSQC NMR spectrum of compound **10** in DMSO-$d_6$

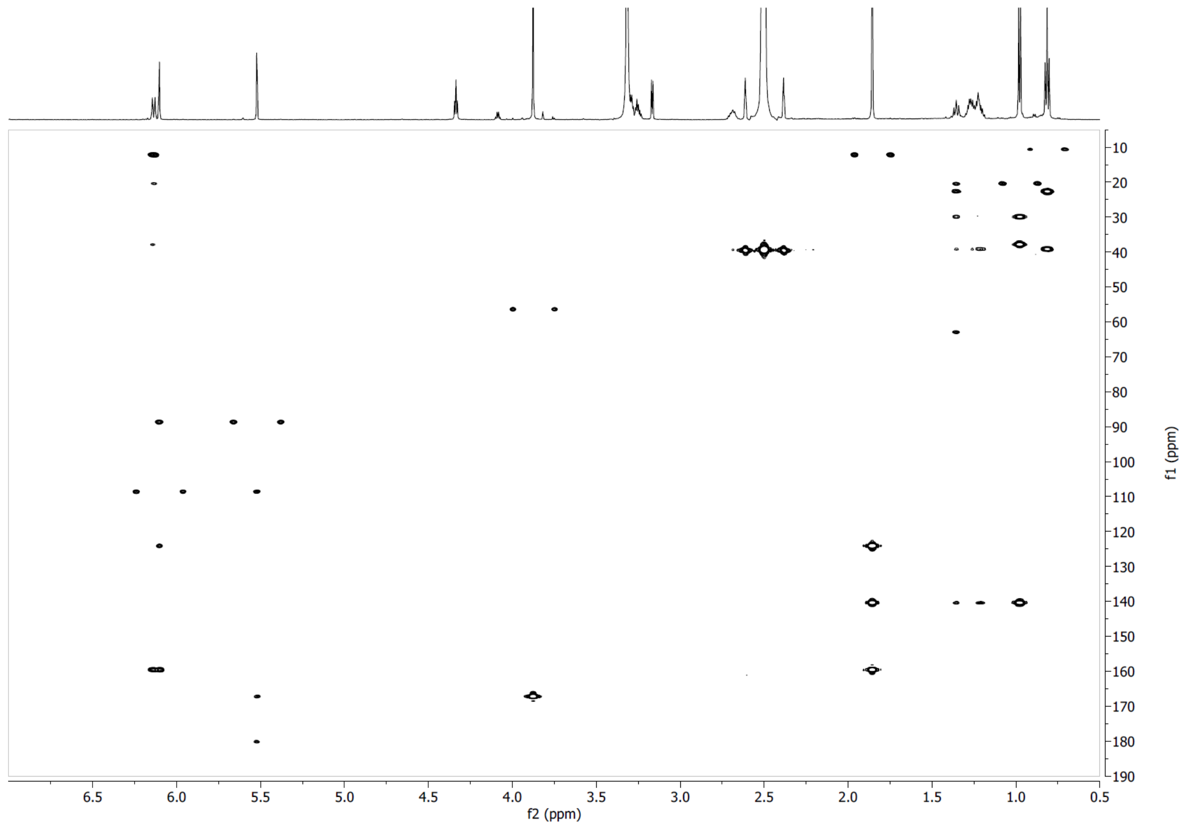

Figure S43: HMBC NMR spectrum of compound **10** in DMSO-$d_6$

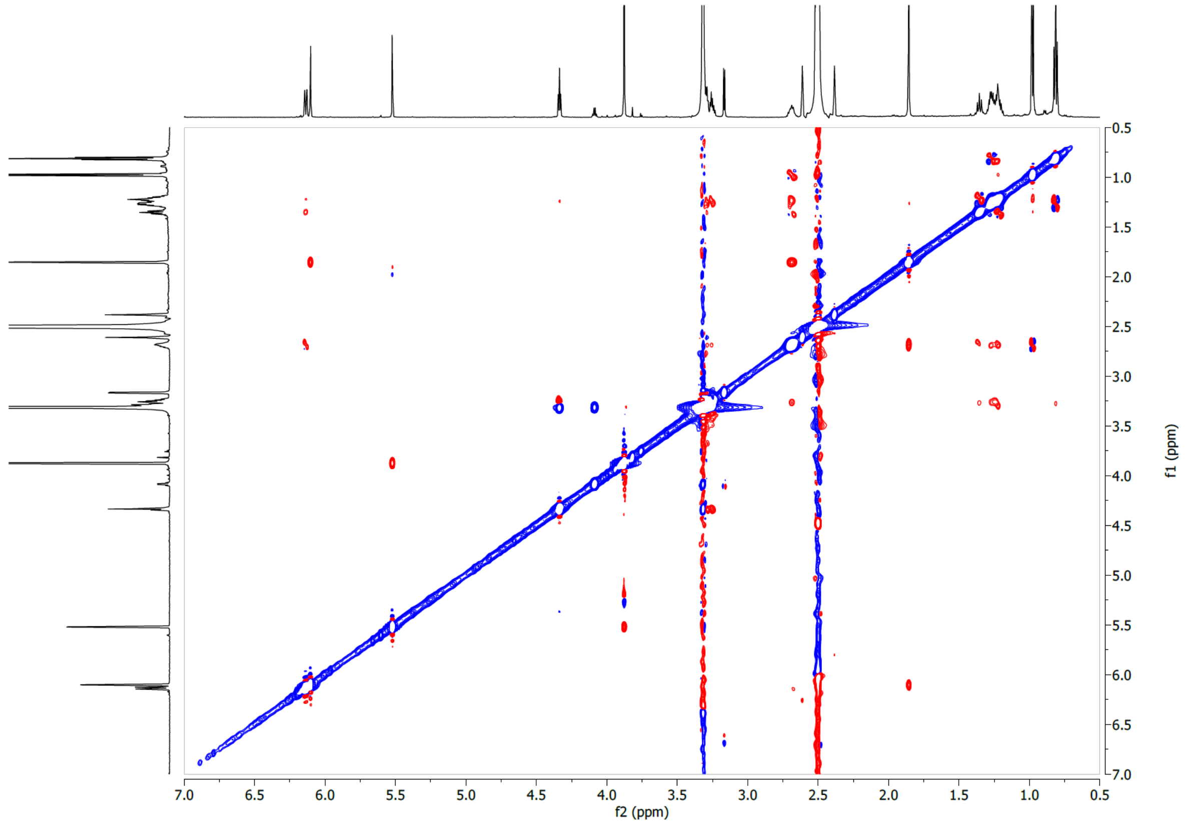


Figure S44: ROESY NMR spectrum of compound **10** in DMSO-$d_6$

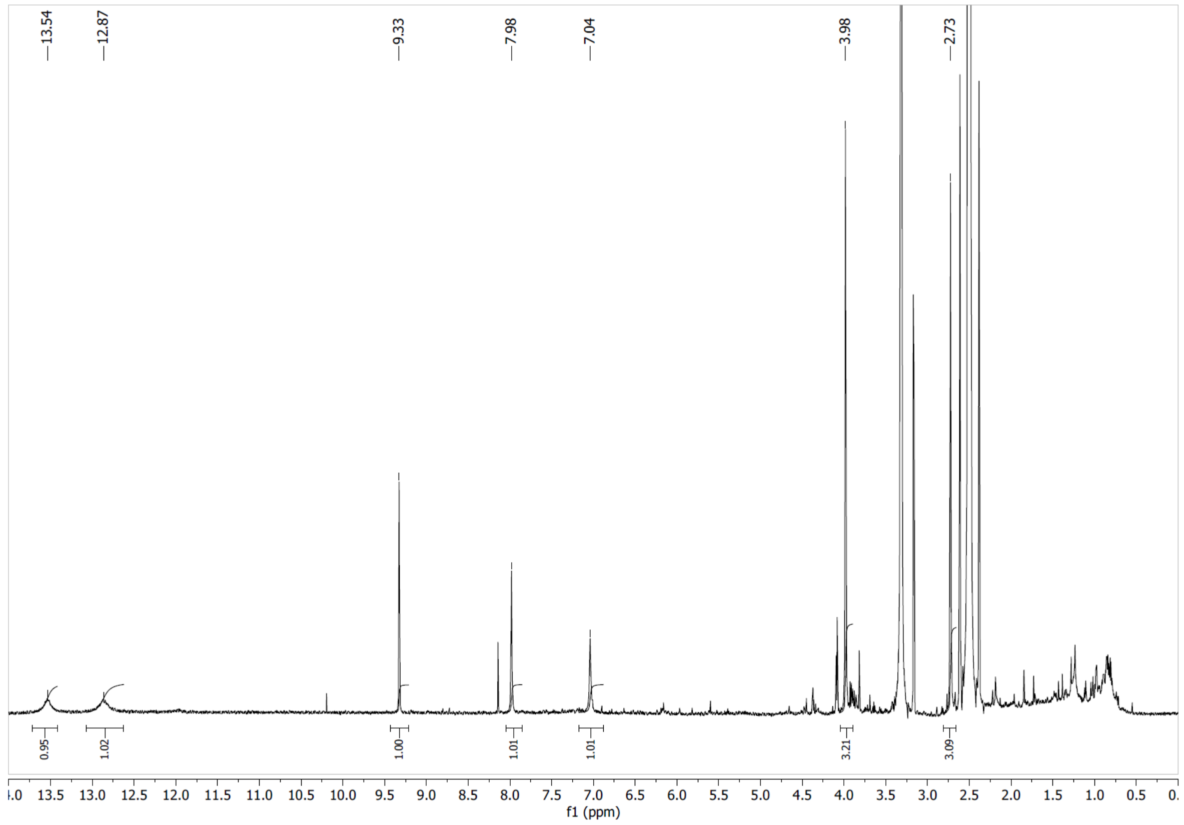

Figure S45: $^{1}$H NMR spectrum of compound **11** in DMSO-$d_6$ at 600 MHz

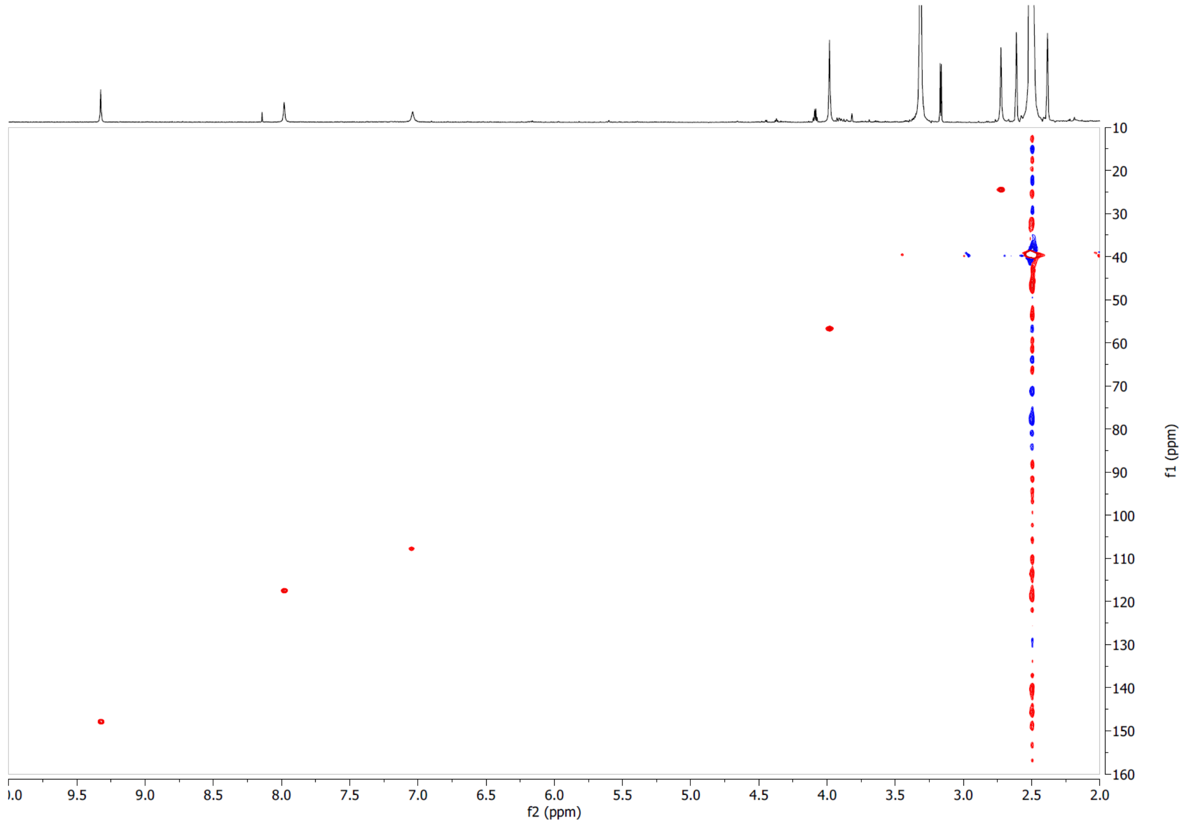


Figure S46: Edited-HSQC NMR spectrum of compound **11** in DMSO-$d_6$

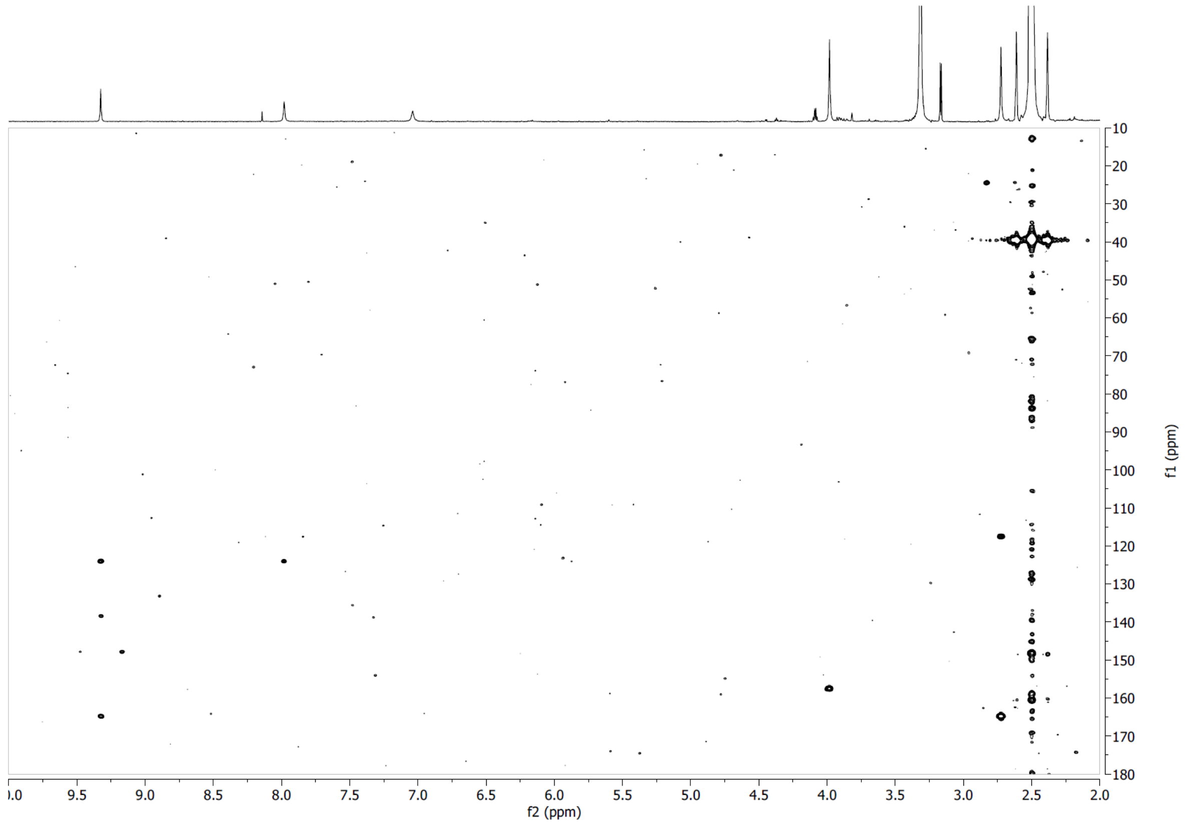

Figure S47: HMBC NMR spectrum of compound **11** in DMSO-$d_6$

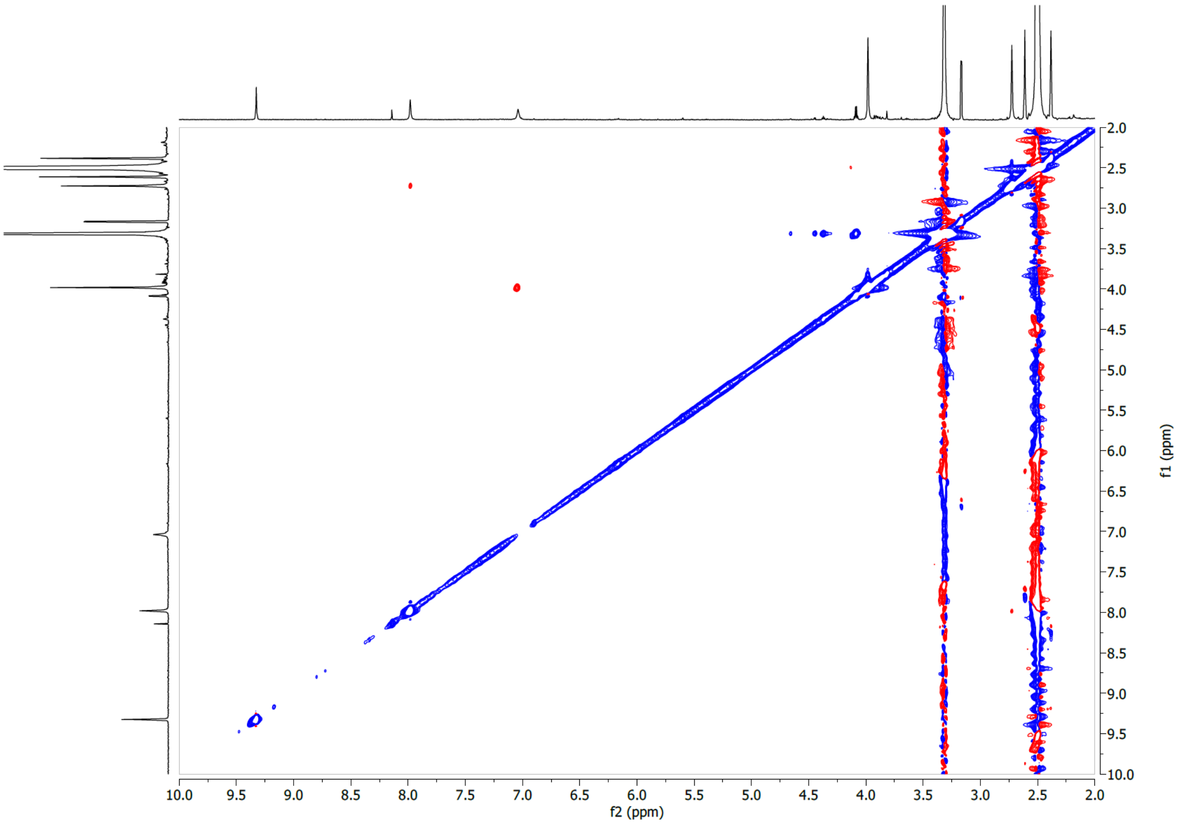


Figure S48: ROESY NMR spectrum of compound **11** in DMSO-$d_6$

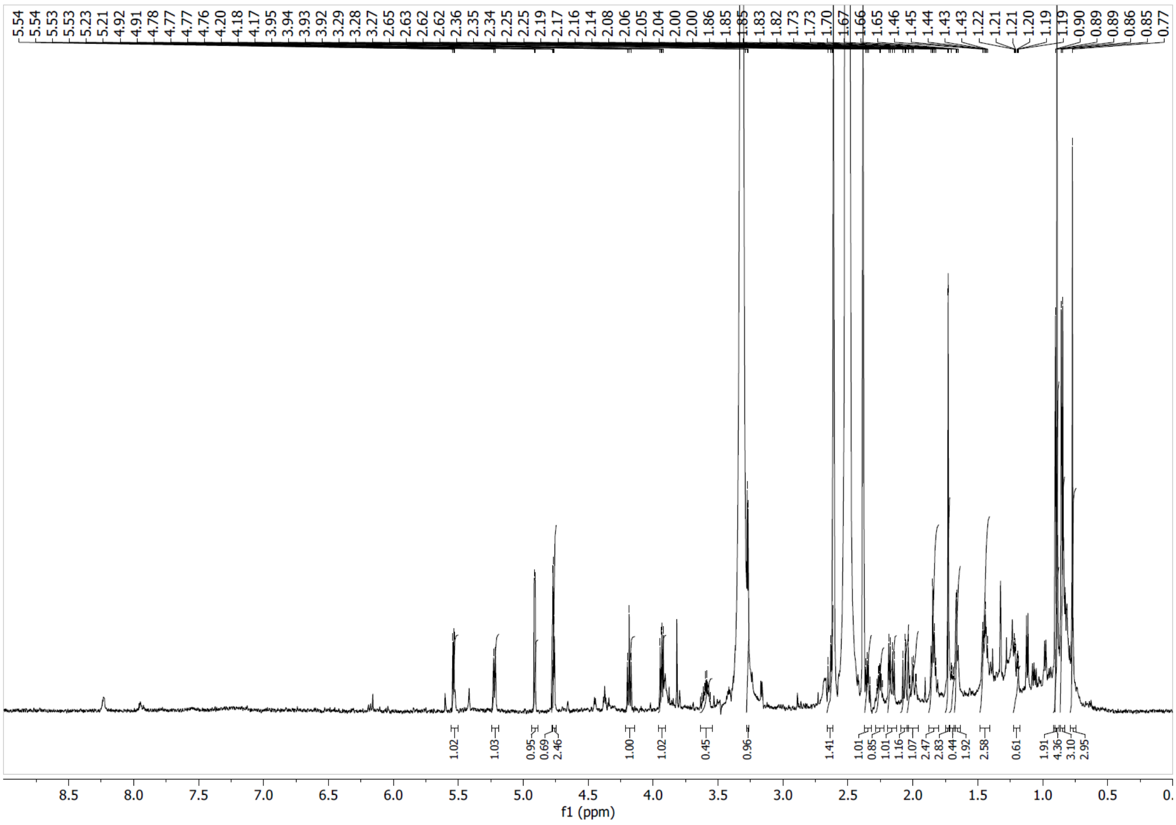

Figure S49: $^{1}$H NMR spectrum of compound **12** in DMSO-$d_6$ at 600 MHz

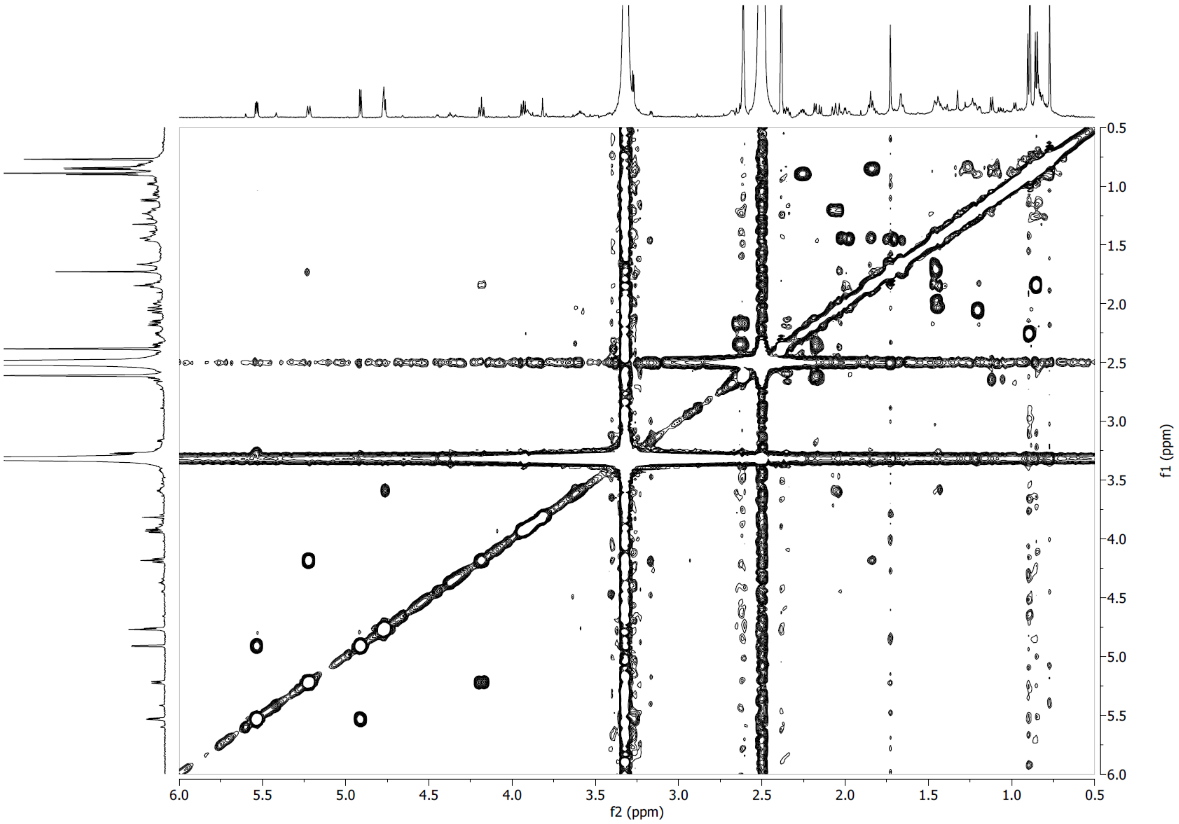


Figure S50: COSY NMR spectrum of compound **12** in DMSO-$d_6$

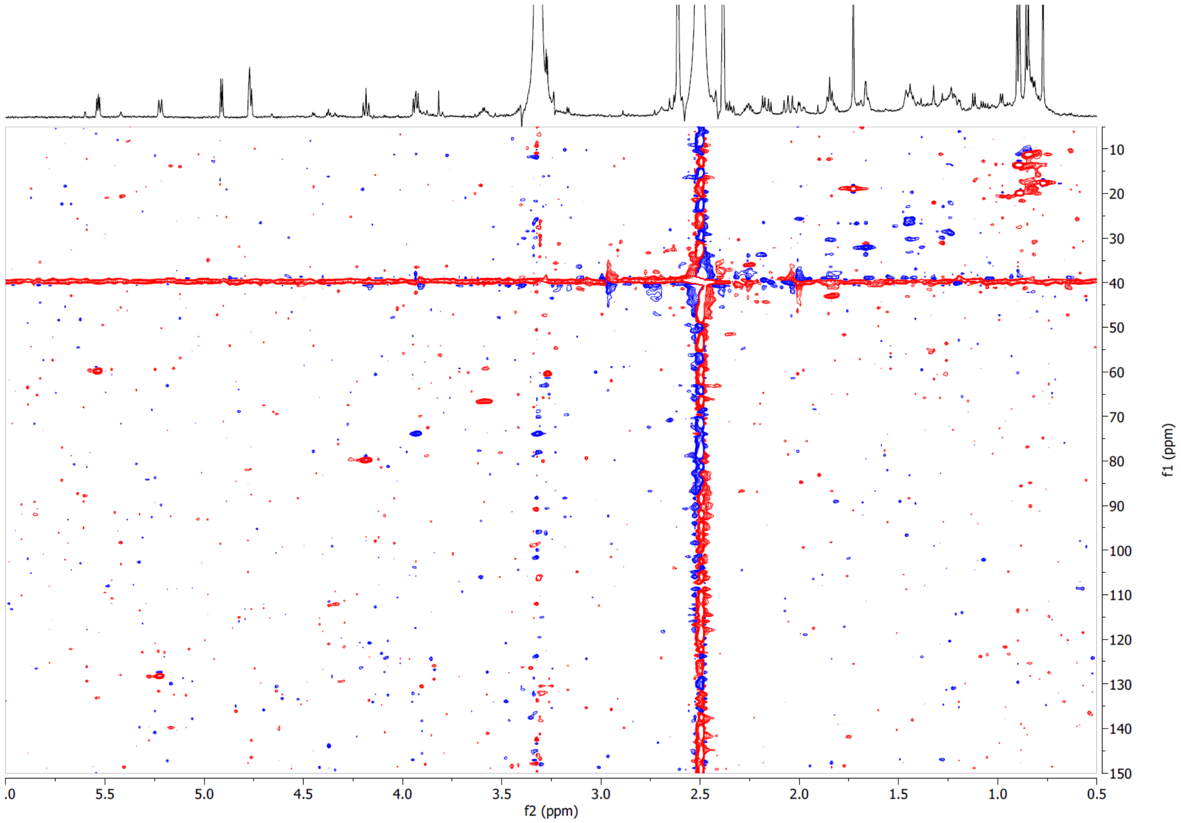

Figure S51: Edited-HSQC NMR spectrum of compound **12** in DMSO-$d_6$

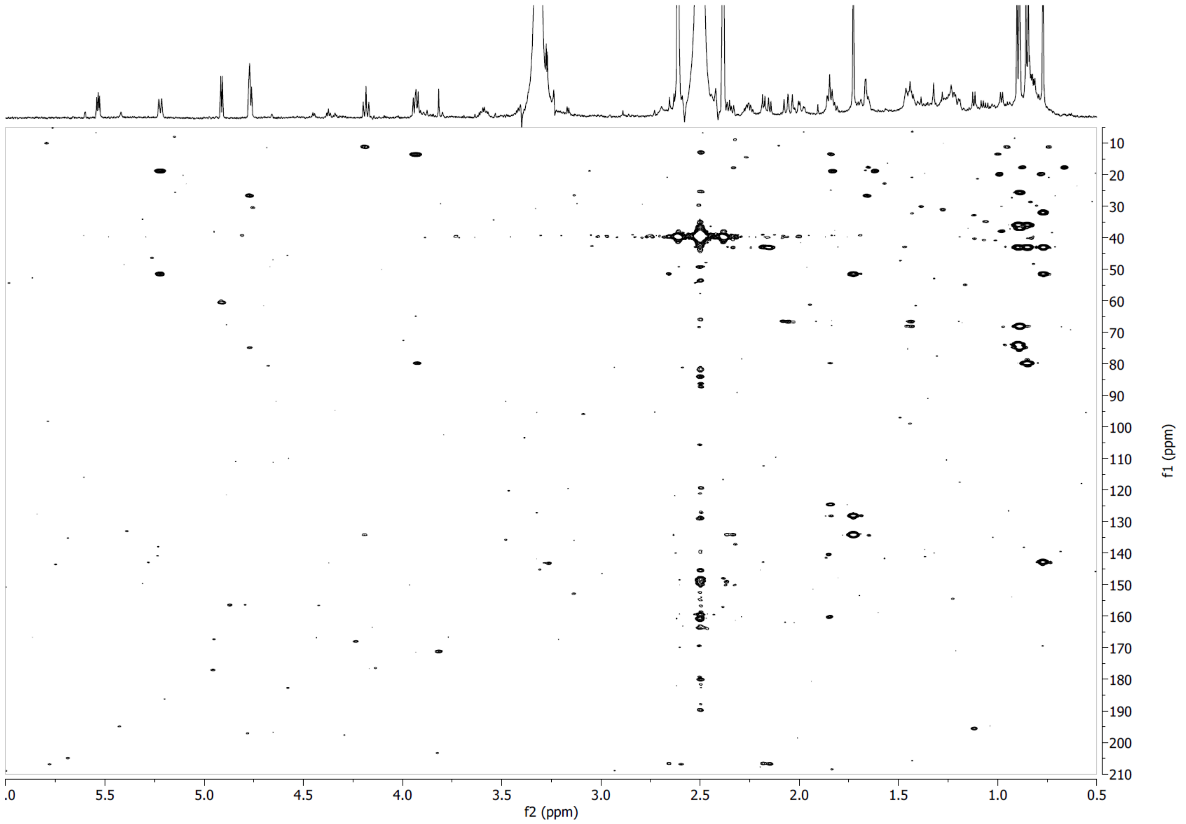


Figure S52: HMBC NMR spectrum of compound **12** in DMSO-$d_6$

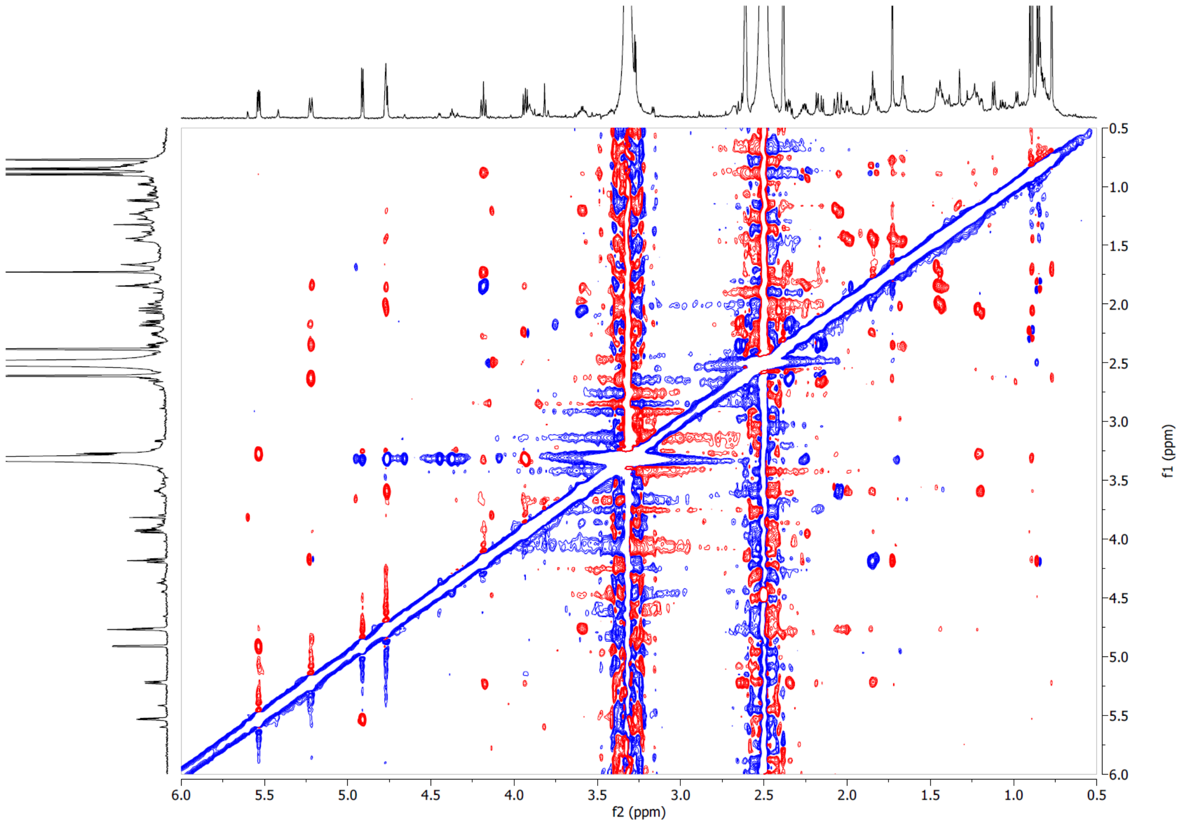

Figure S53: ROESY NMR spectrum of compound **12** in DMSO-$d_6$

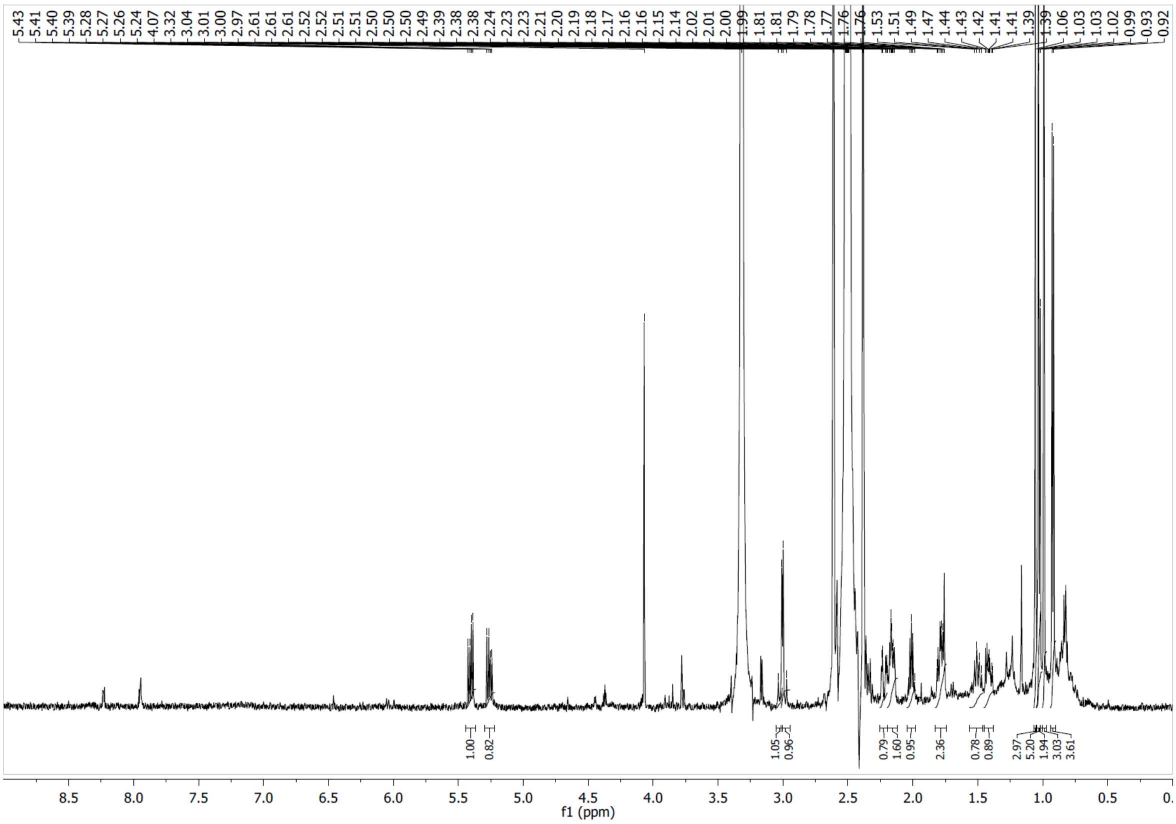


Figure S54: $^{1}H$ NMR spectrum of compound **13** in DMSO-$d_6$ at 600 MHz

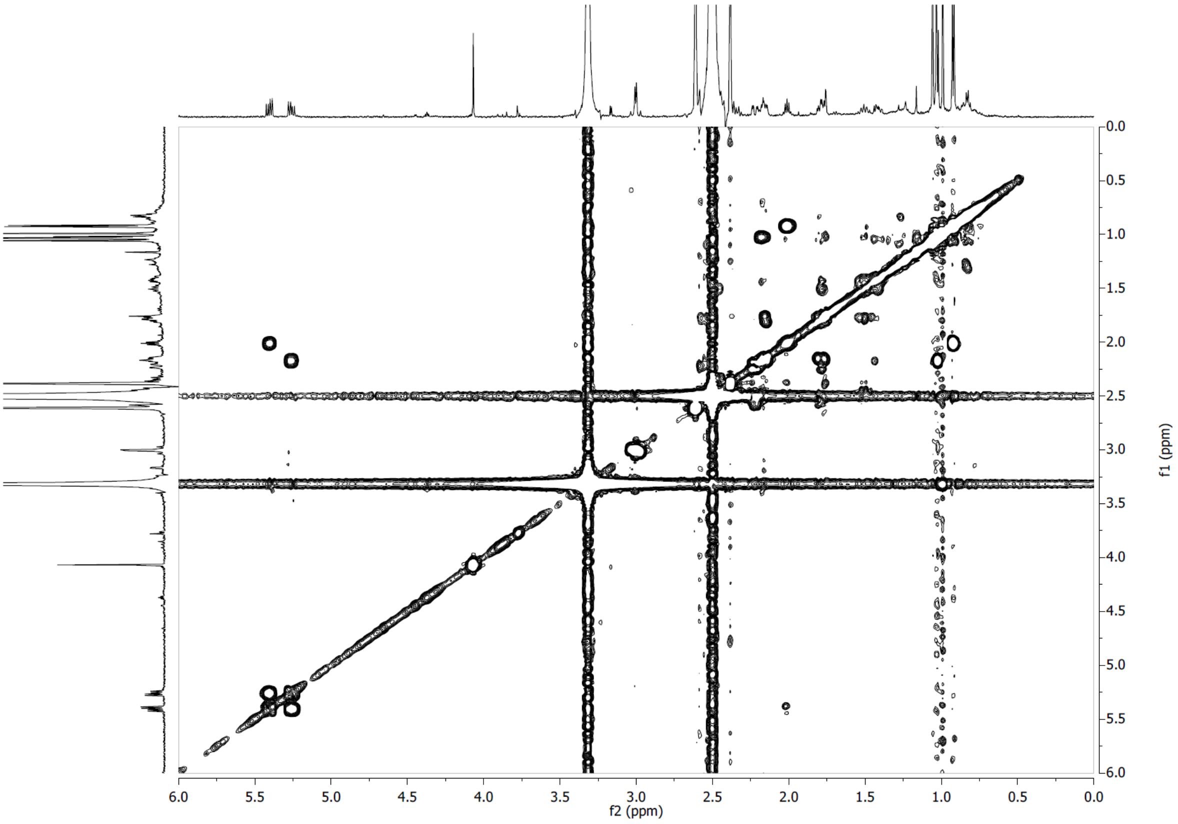

Figure S55: COSY NMR spectrum of compound **13** in DMSO-$d_6$

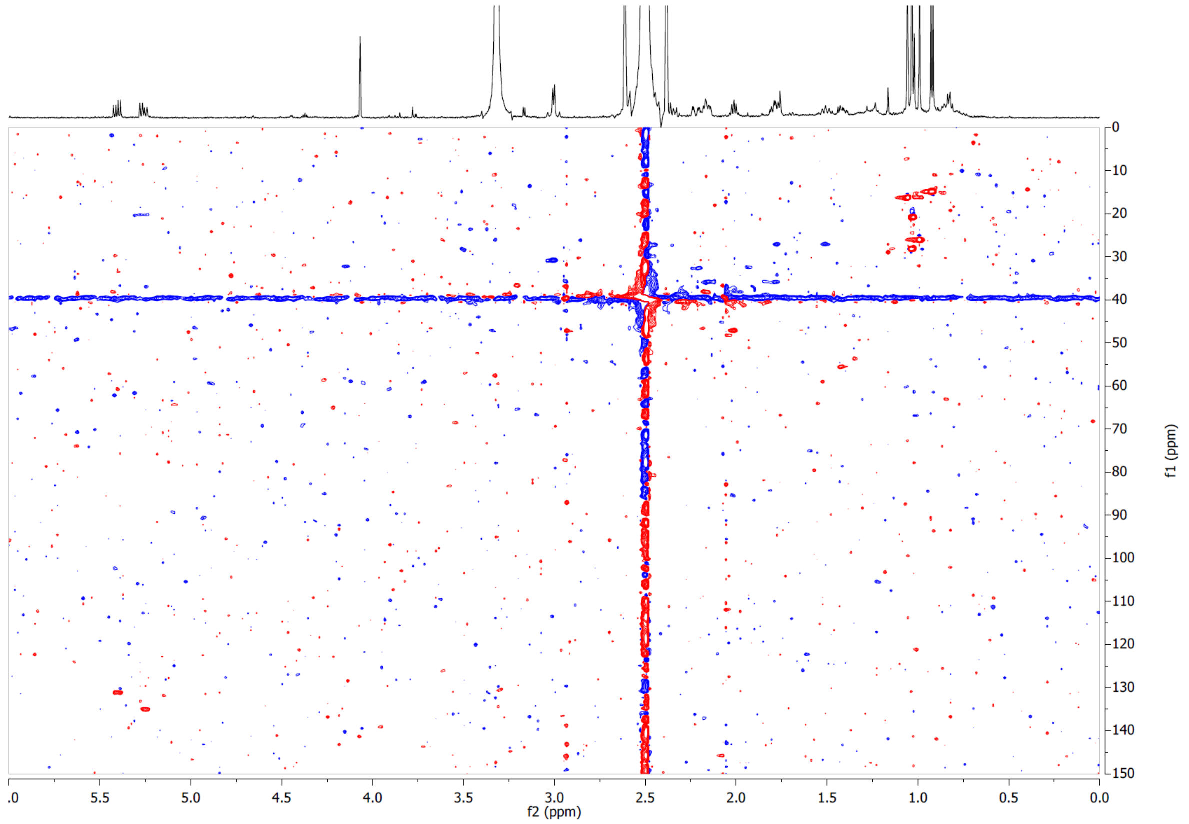


Figure S56: Edited-HSQC NMR spectrum of compound **13** in DMSO-$d_6$

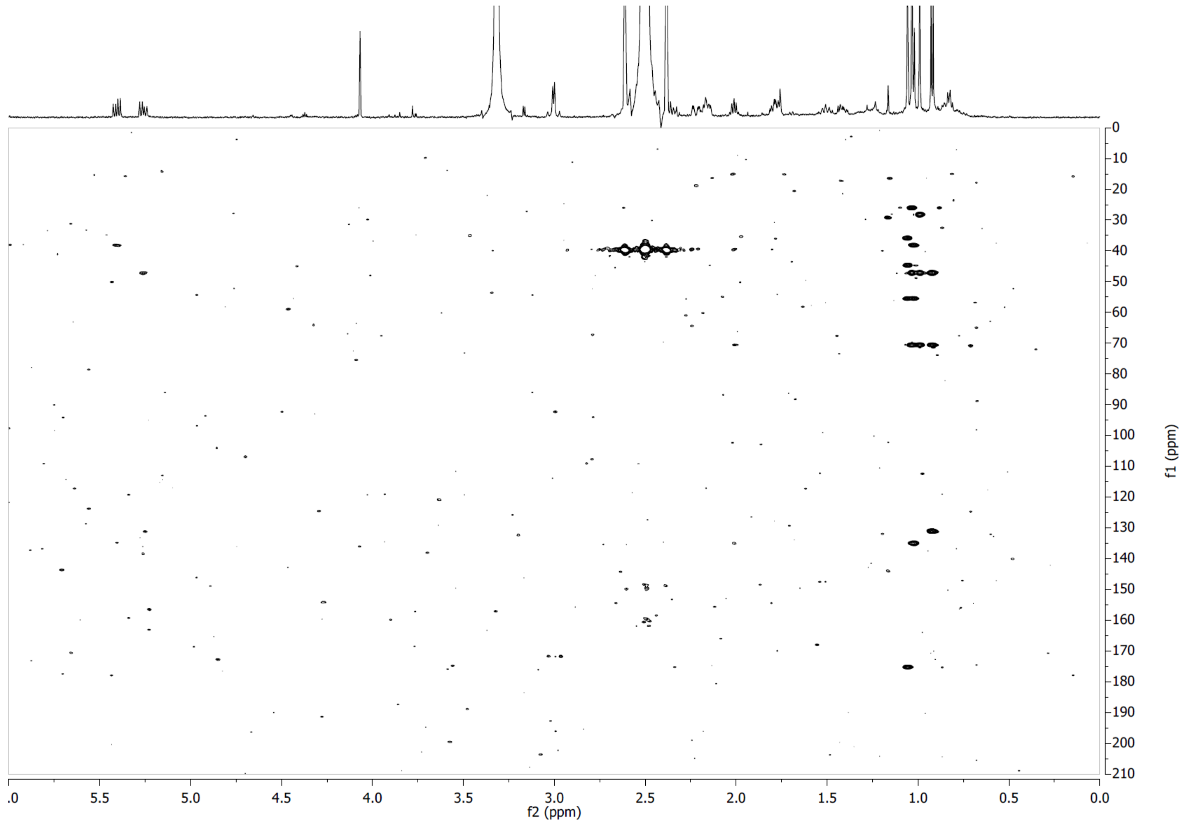

Figure S57: HMBC NMR spectrum of compound **13** in DMSO-$d_6$

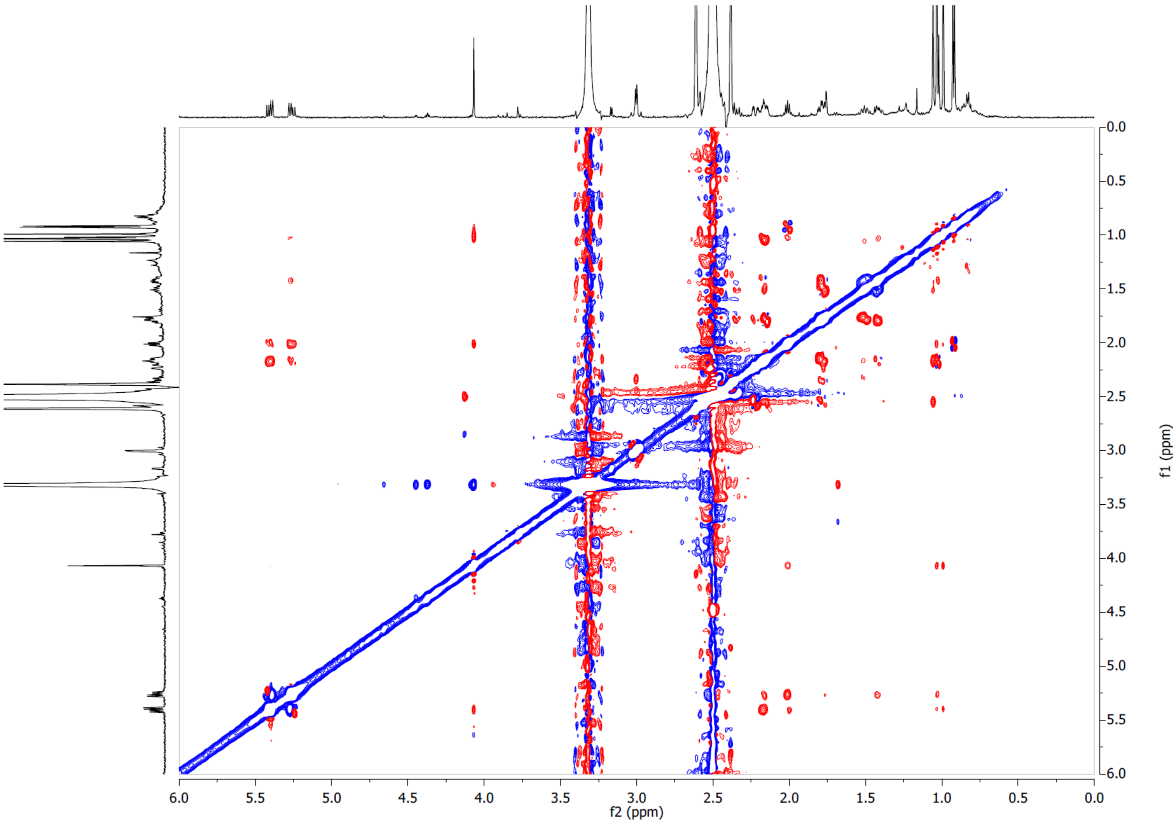


Figure S58: ROESY NMR spectrum of compound **13** in DMSO-$d_6$

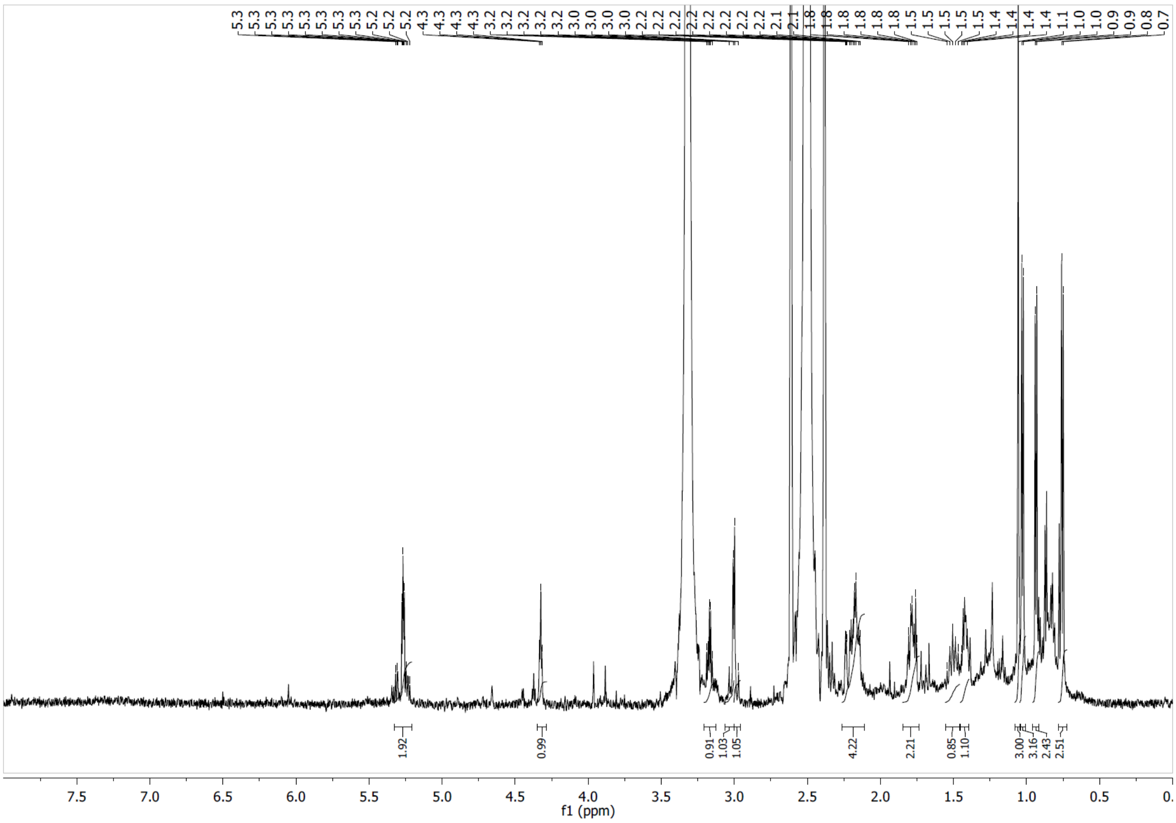

Figure S59: $^{1}$H NMR spectrum of compound **14** in DMSO-$d_6$ at 600 MHz

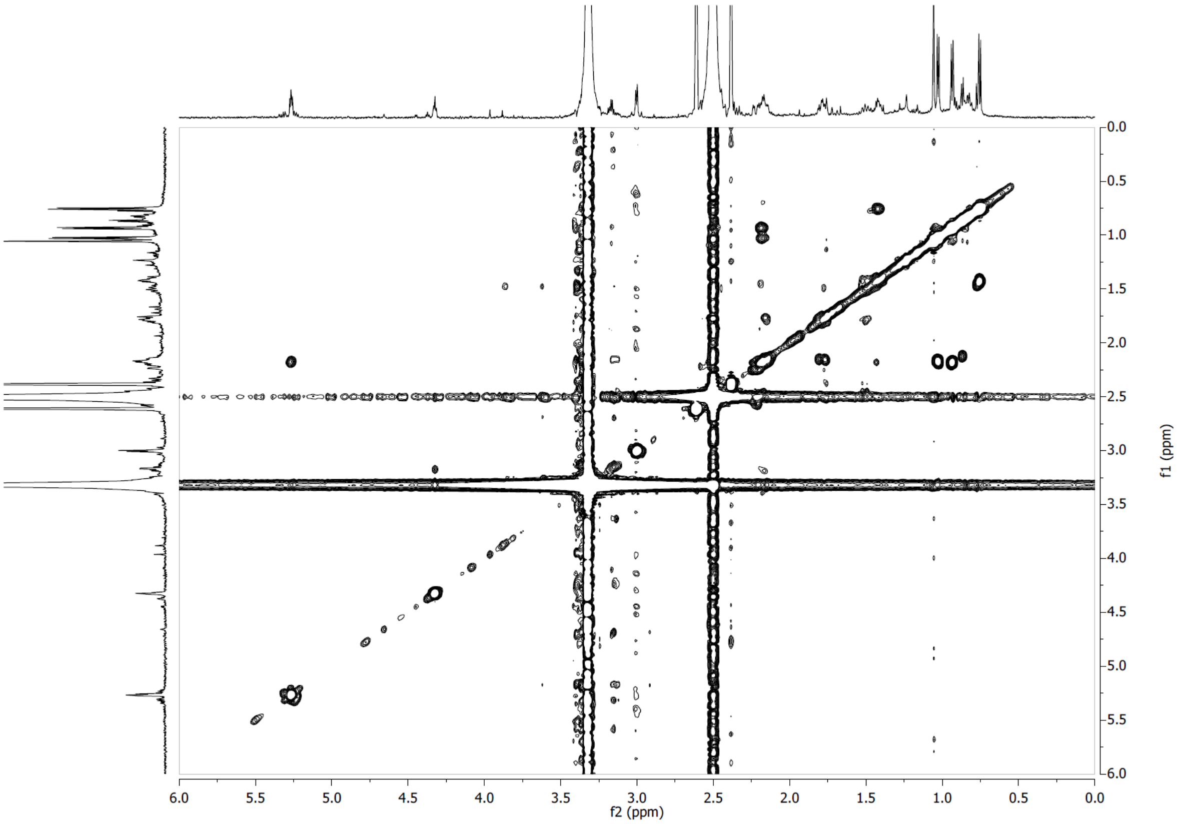


Figure S60: COSY NMR spectrum of compound **14** in DMSO-$d_6$

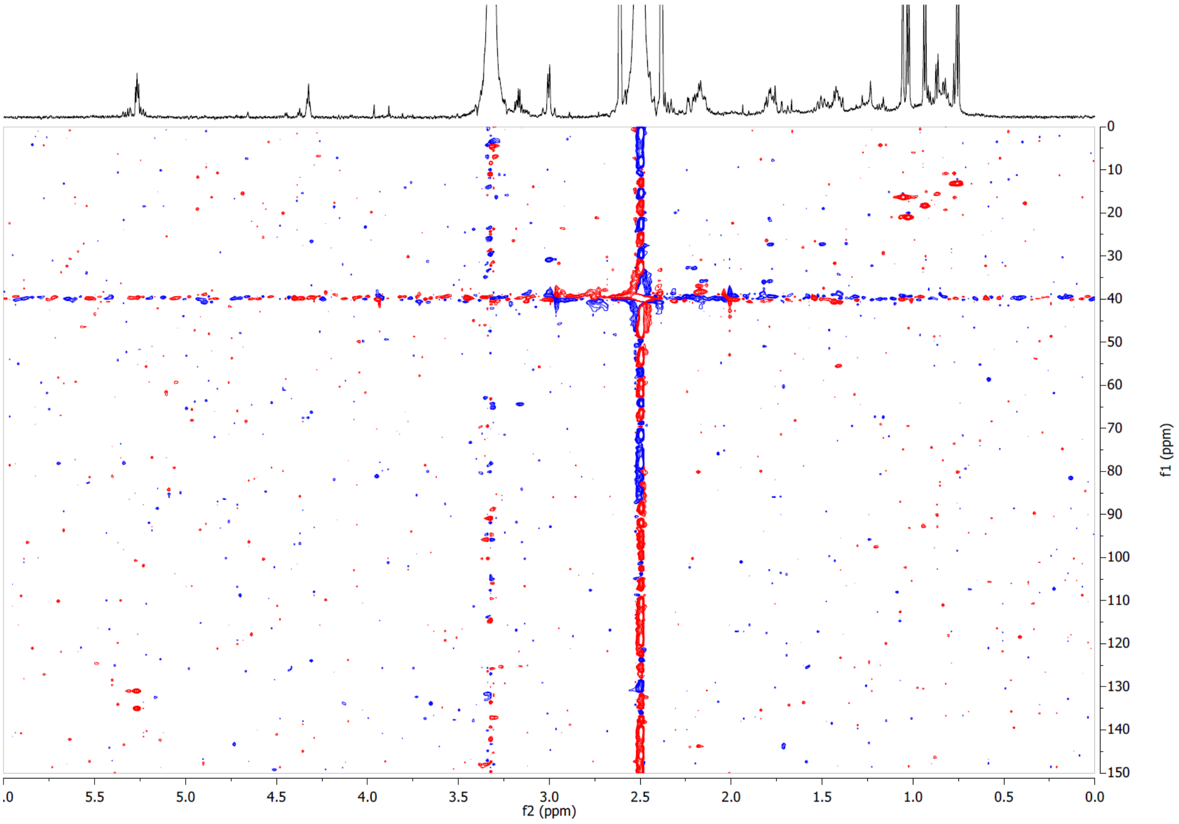

Figure S61: Edited-HSQC NMR spectrum of compound **14** in DMSO-$d_6$

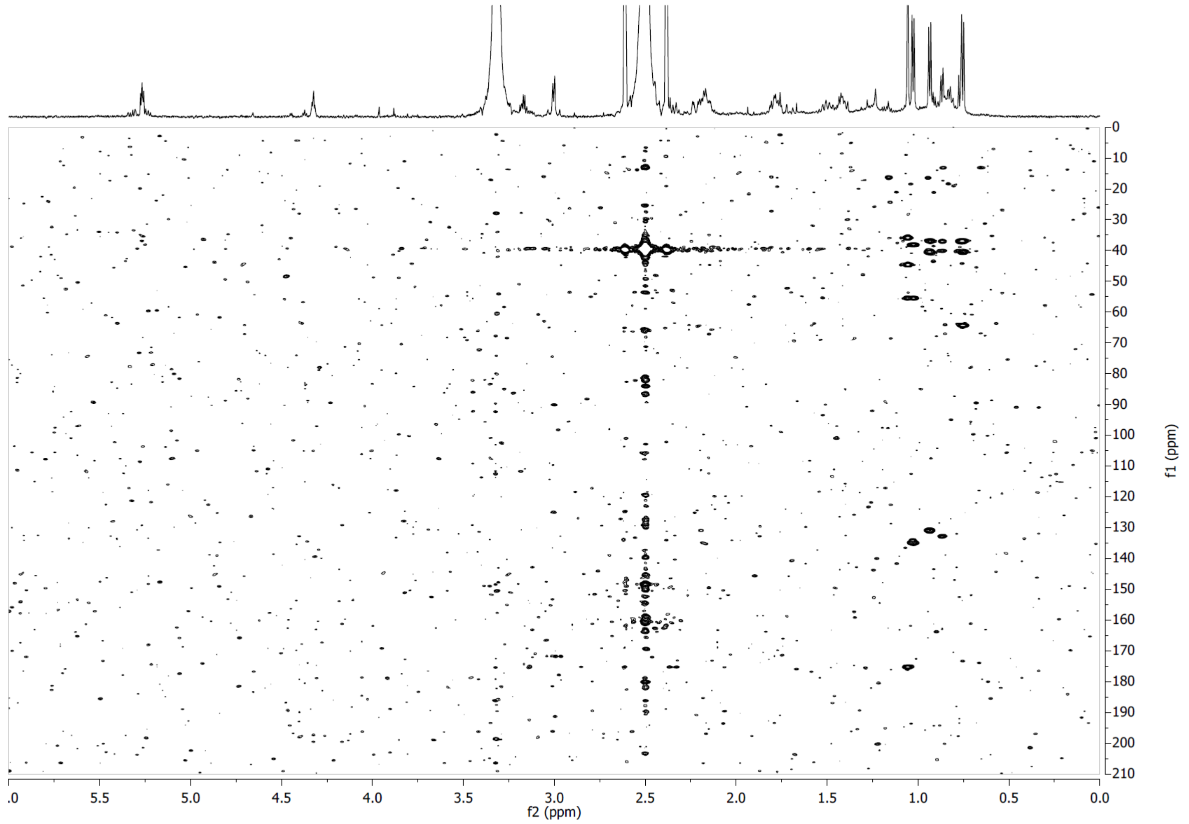


Figure S62: HMBC NMR spectrum of compound **14** in DMSO-$d_6$

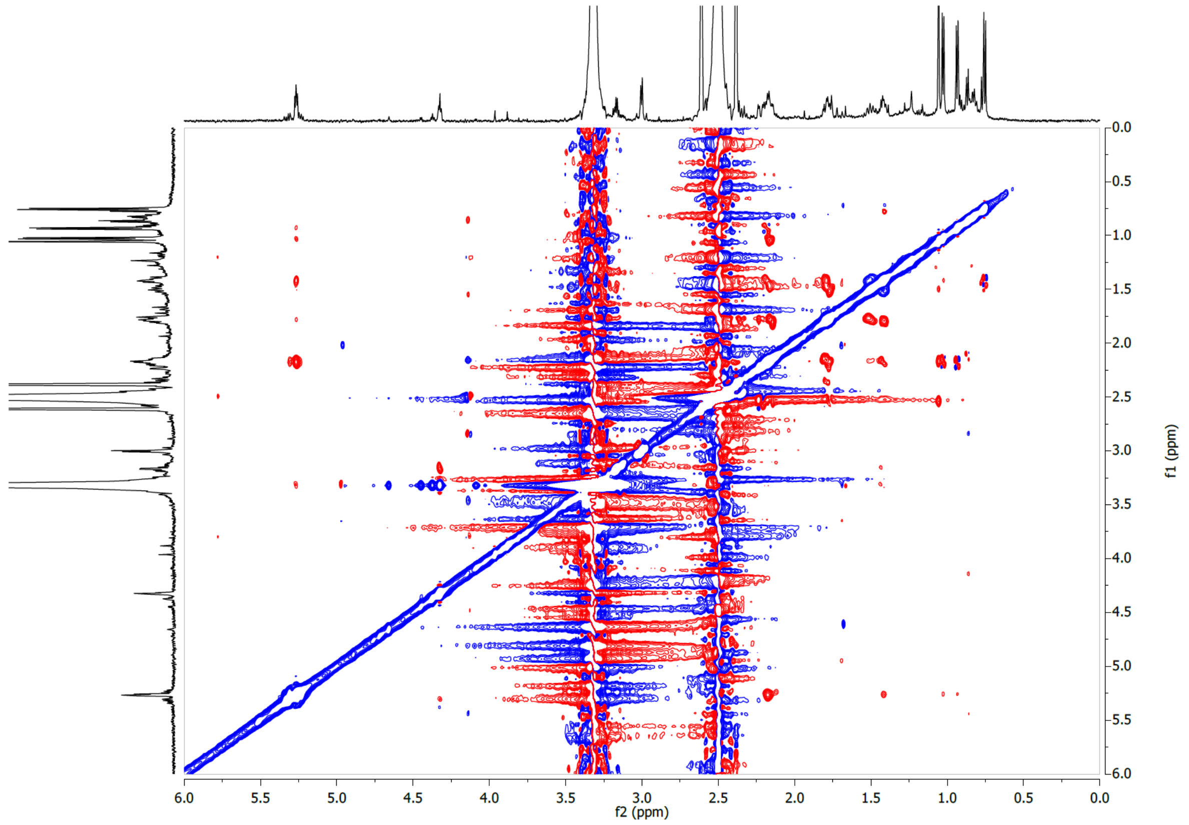

Figure S63: ROESY NMR spectrum of compound **14** in DMSO-$d_6$

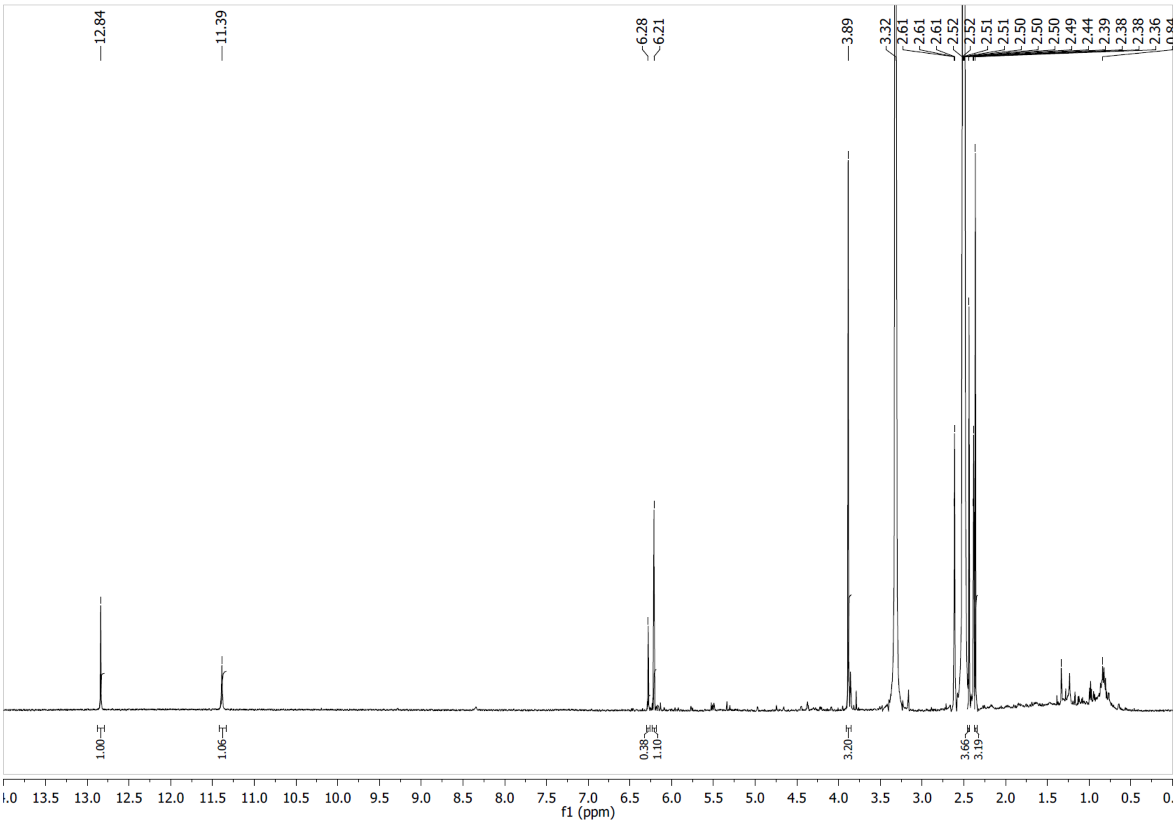


Figure S64: $^{1}$H NMR spectrum of compound **15** in DMSO-$d_6$ at 600 MHz

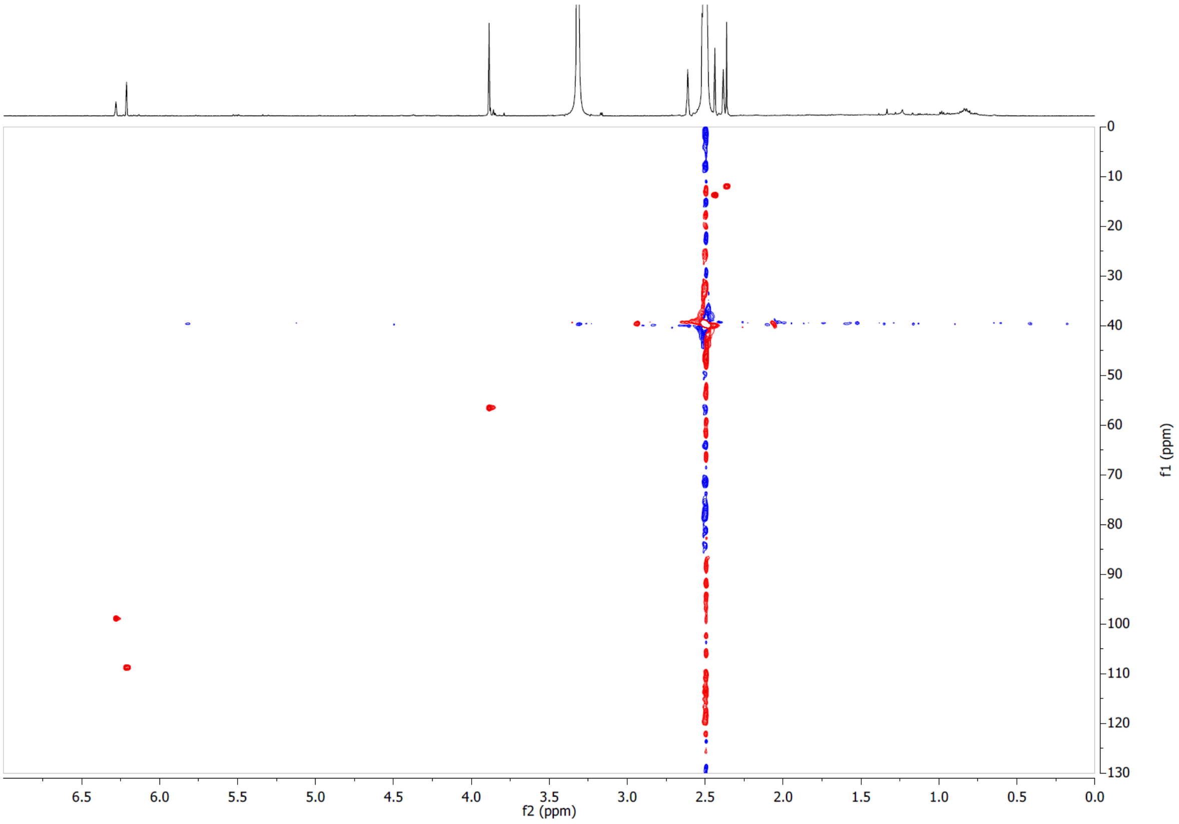

Figure S65: Edited-HSQC NMR spectrum of compound **15** in DMSO-$d_6$

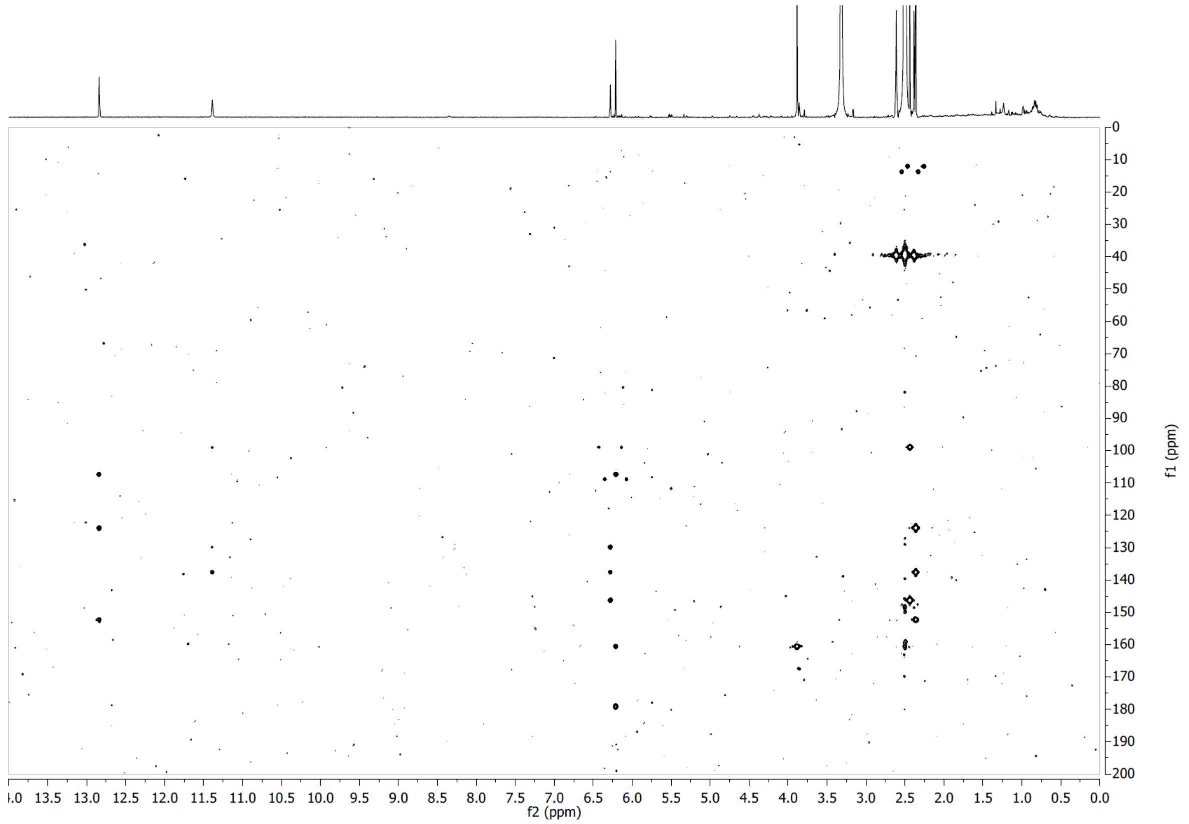


Figure S66: HMBC NMR spectrum of compound **15** in DMSO-$d_6$

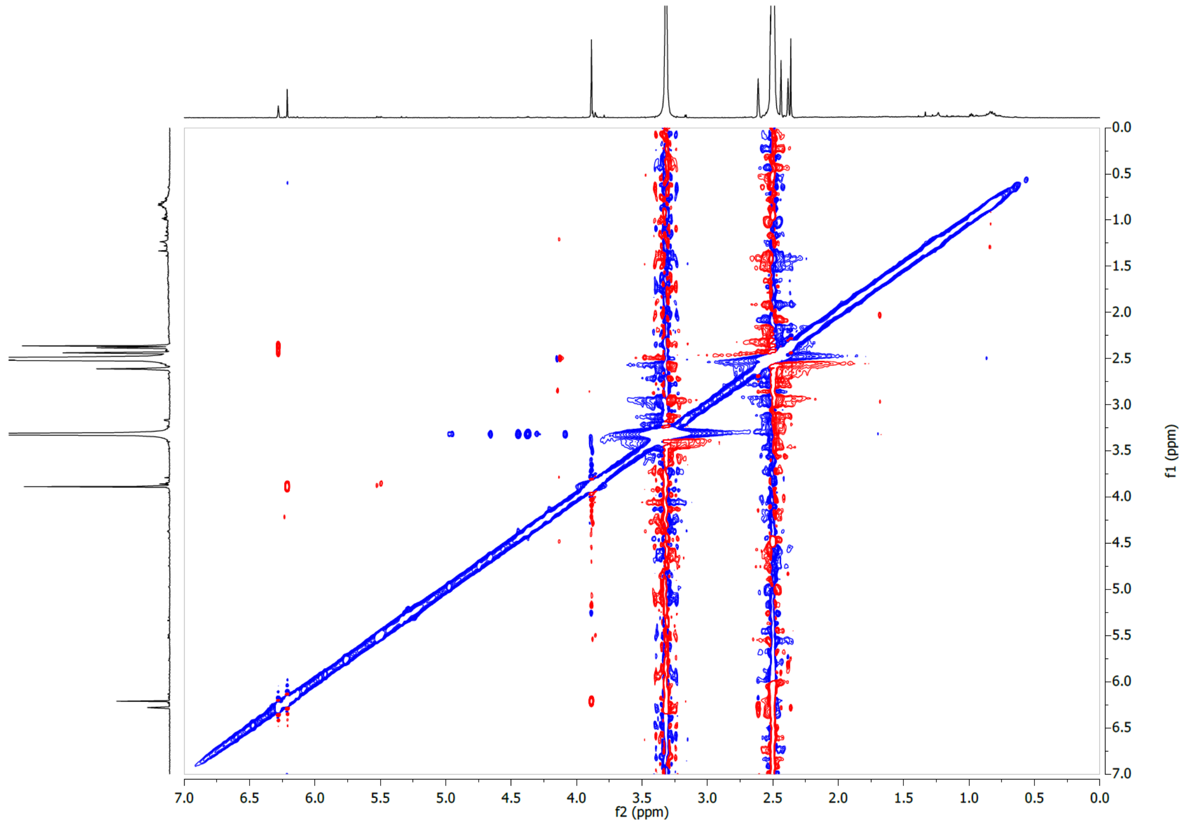

Figure S67: ROESY NMR spectrum of compound **15** in DMSO-$d_6$

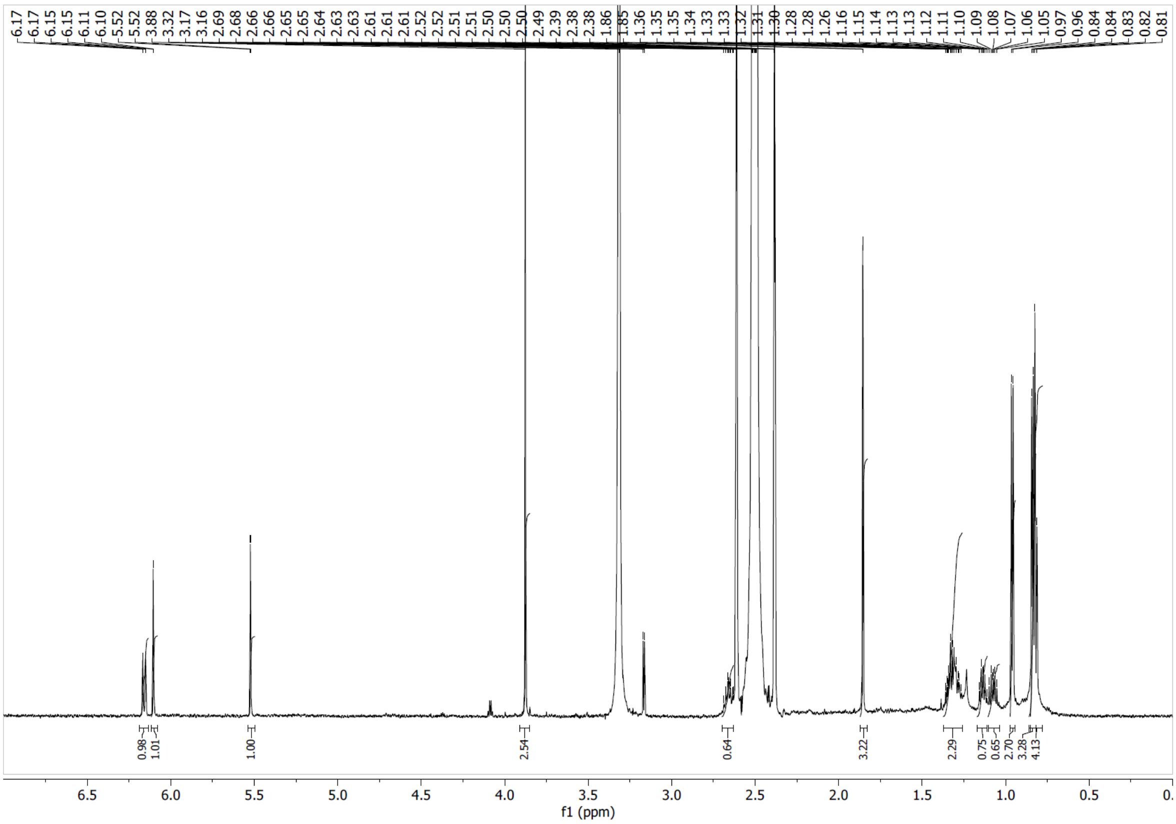


Figure S68: $^{1}$H NMR spectrum of compound **16** in DMSO-$d_6$ at 600 MHz

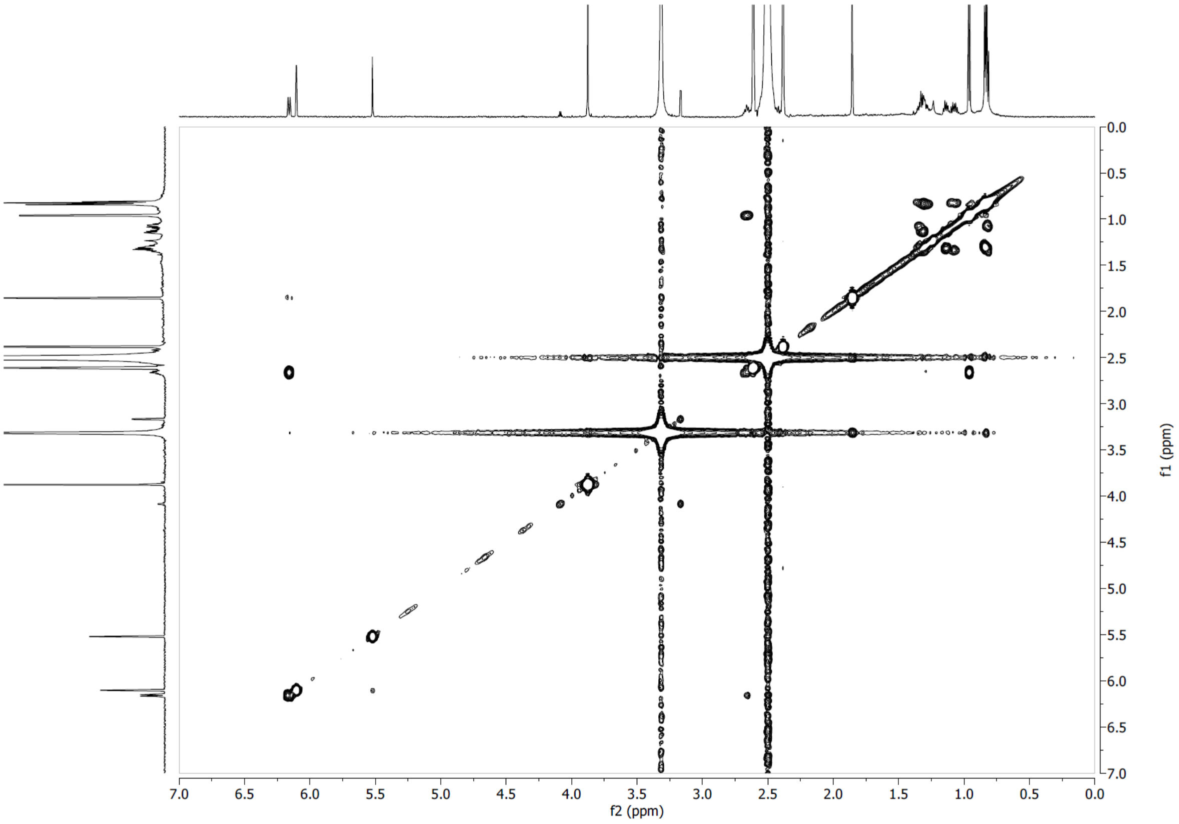

Figure S69: COSY NMR spectrum of compound **16** in DMSO-$d_6$

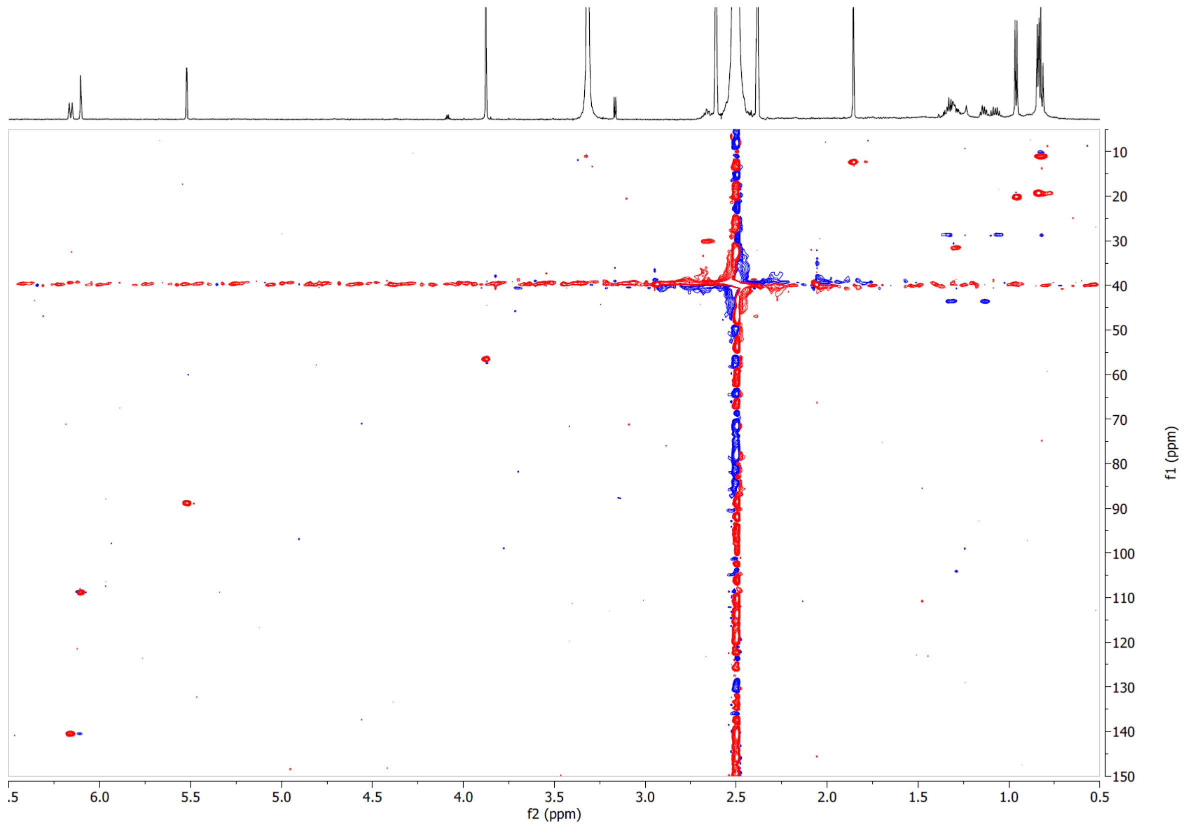


Figure S70: Edited-HSQC NMR spectrum of compound **16** in DMSO-$d_6$

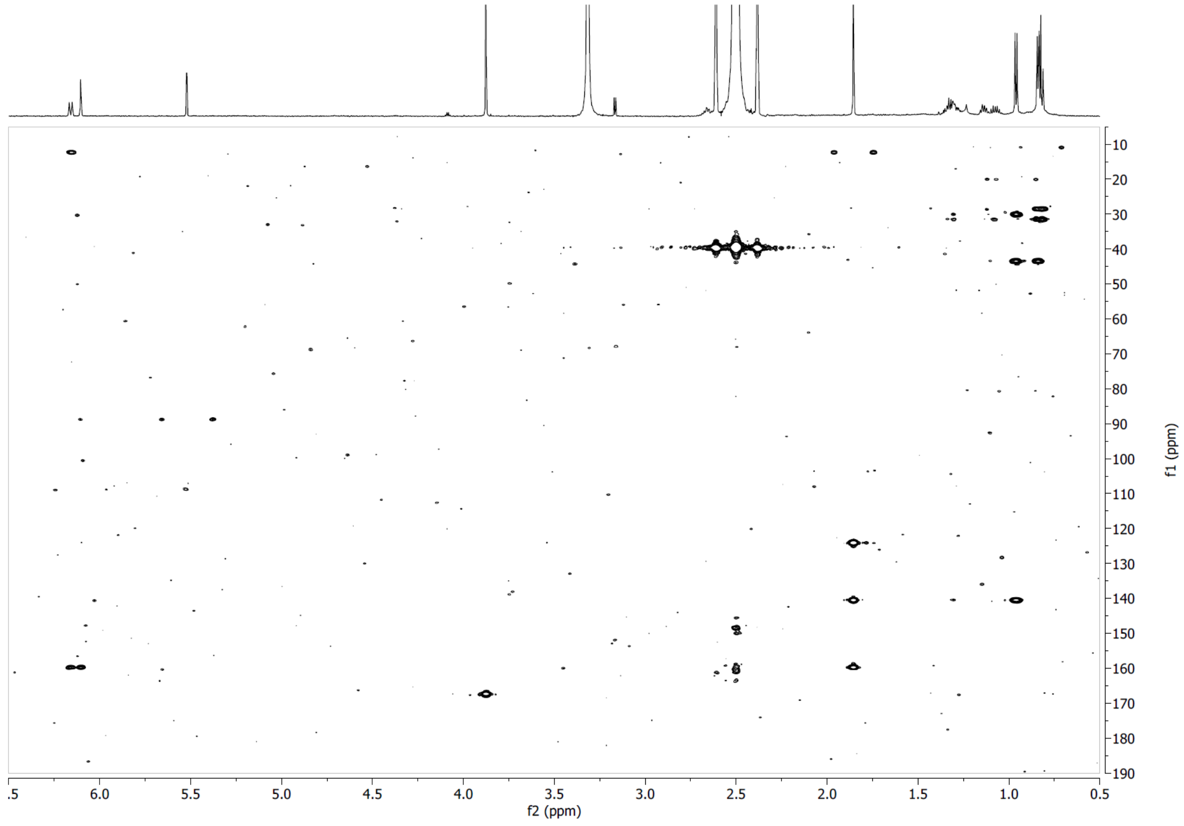

Figure S71: HMBC NMR spectrum of compound **16** in DMSO-$d_6$

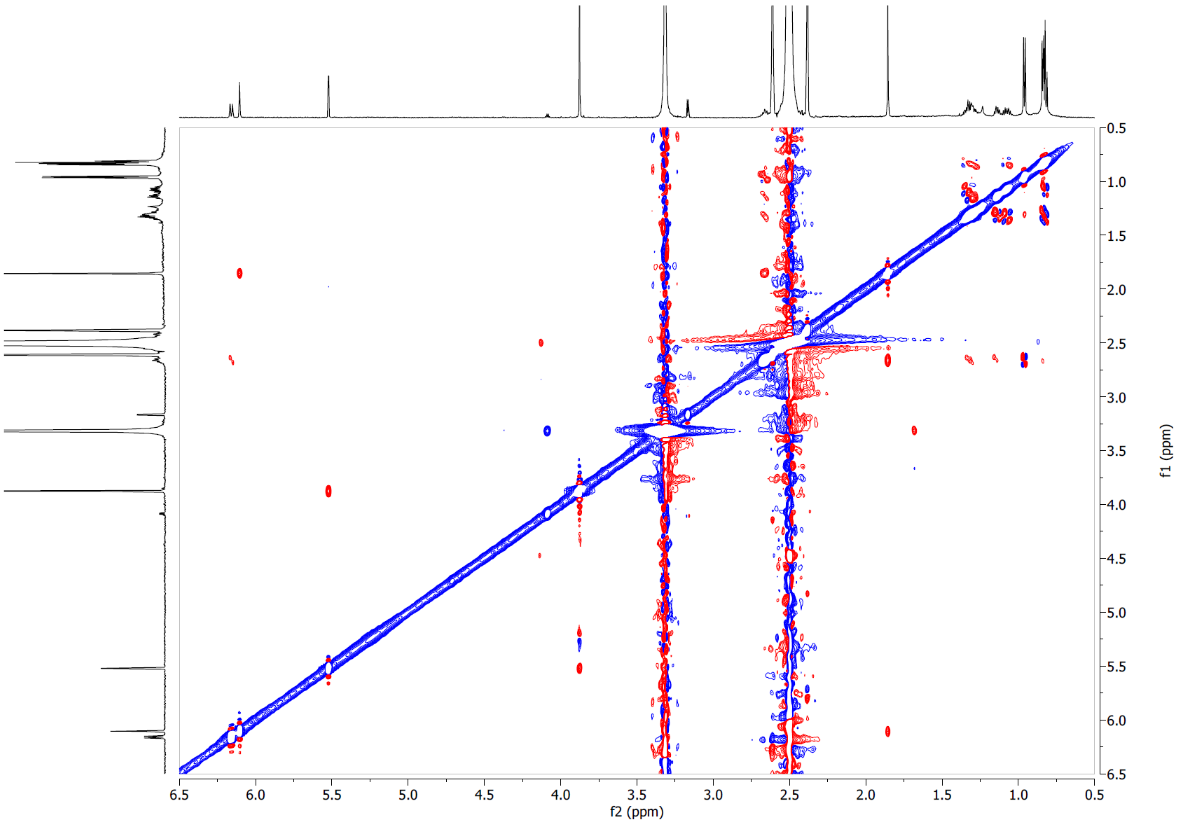


Figure S72: ROESY NMR spectrum of compound **16** in DMSO-$d_6$

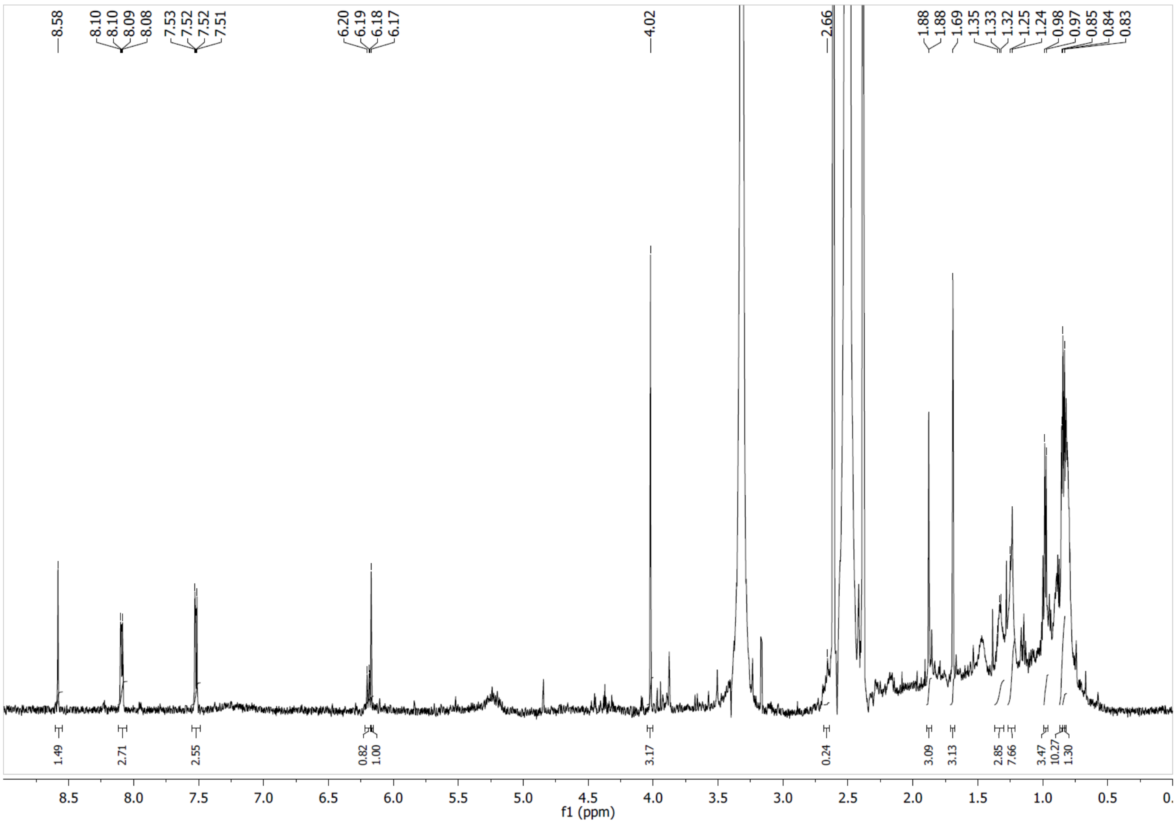

Figure S73: $^{1}$H NMR spectrum of compound **17** in DMSO-$d_6$ at 600 MHz

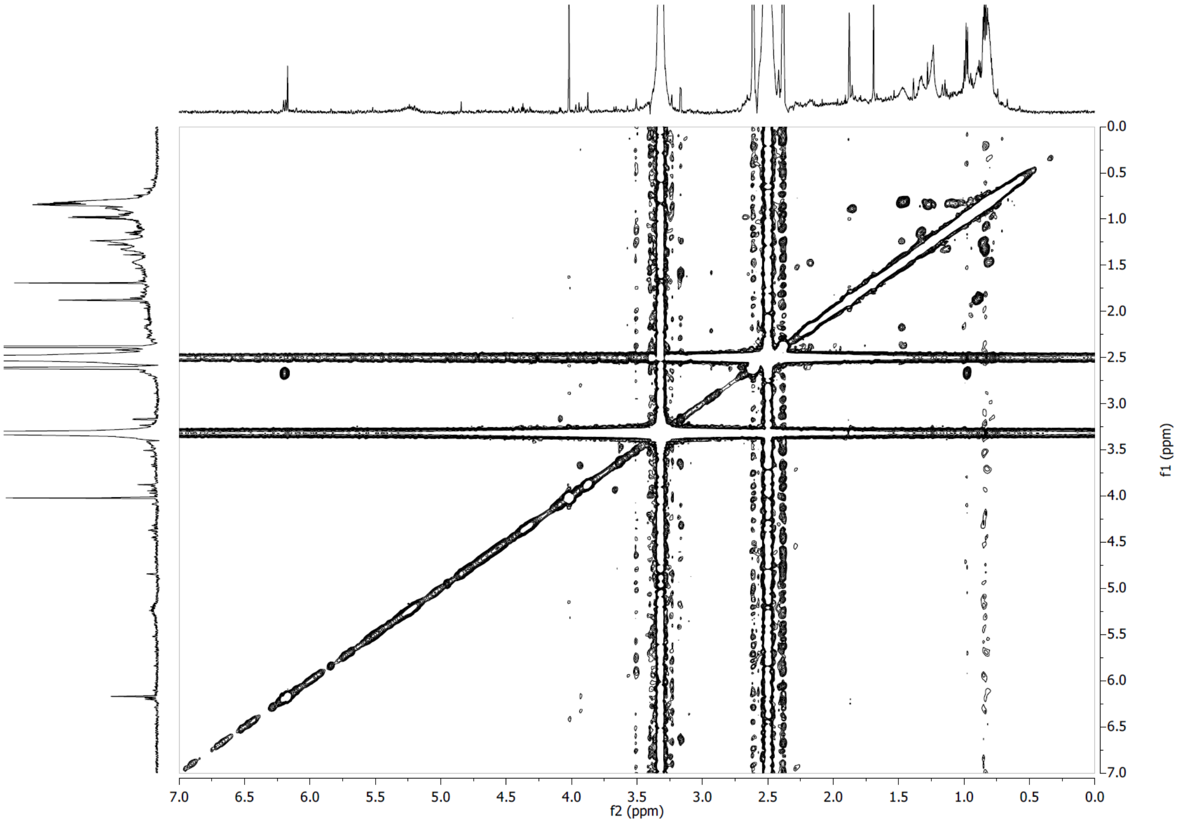


Figure S74: COSY NMR spectrum of compound **17** in DMSO-$d_6$

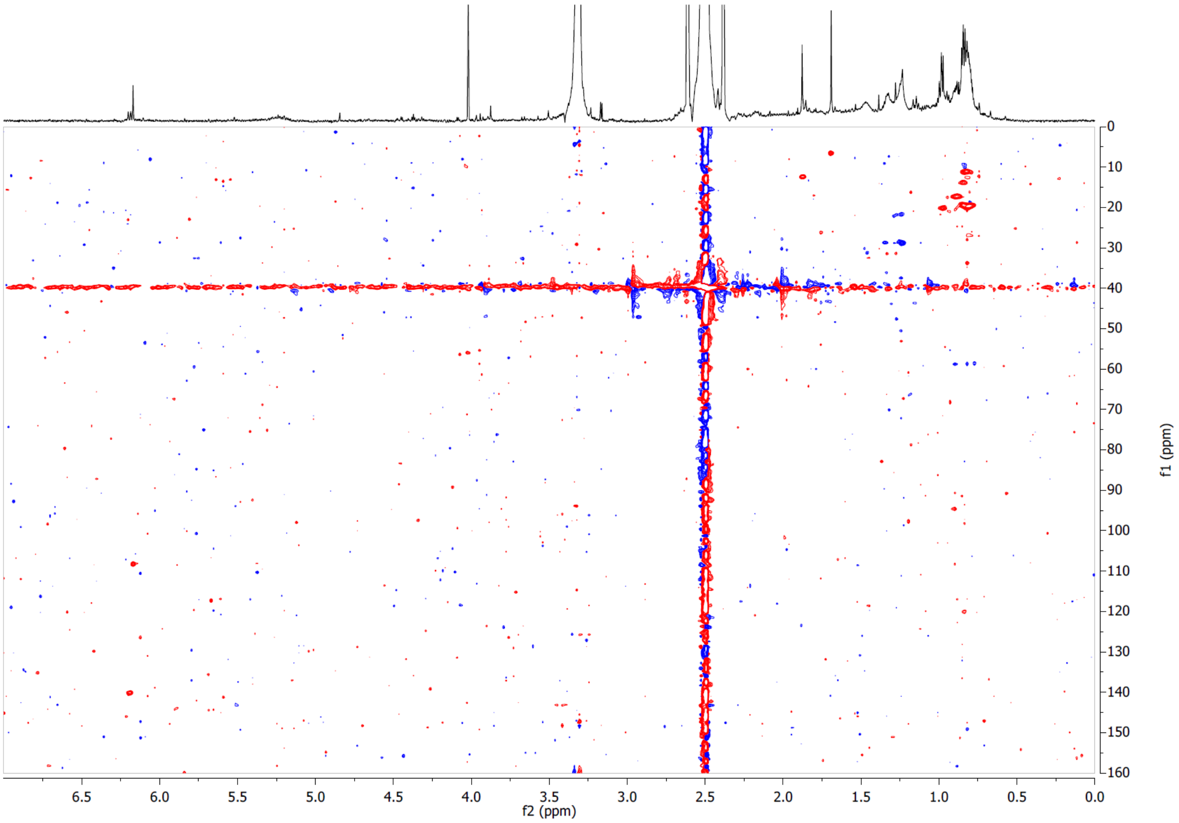

Figure S75: Edited-HSQC NMR spectrum of compound **17** in DMSO-$d_6$

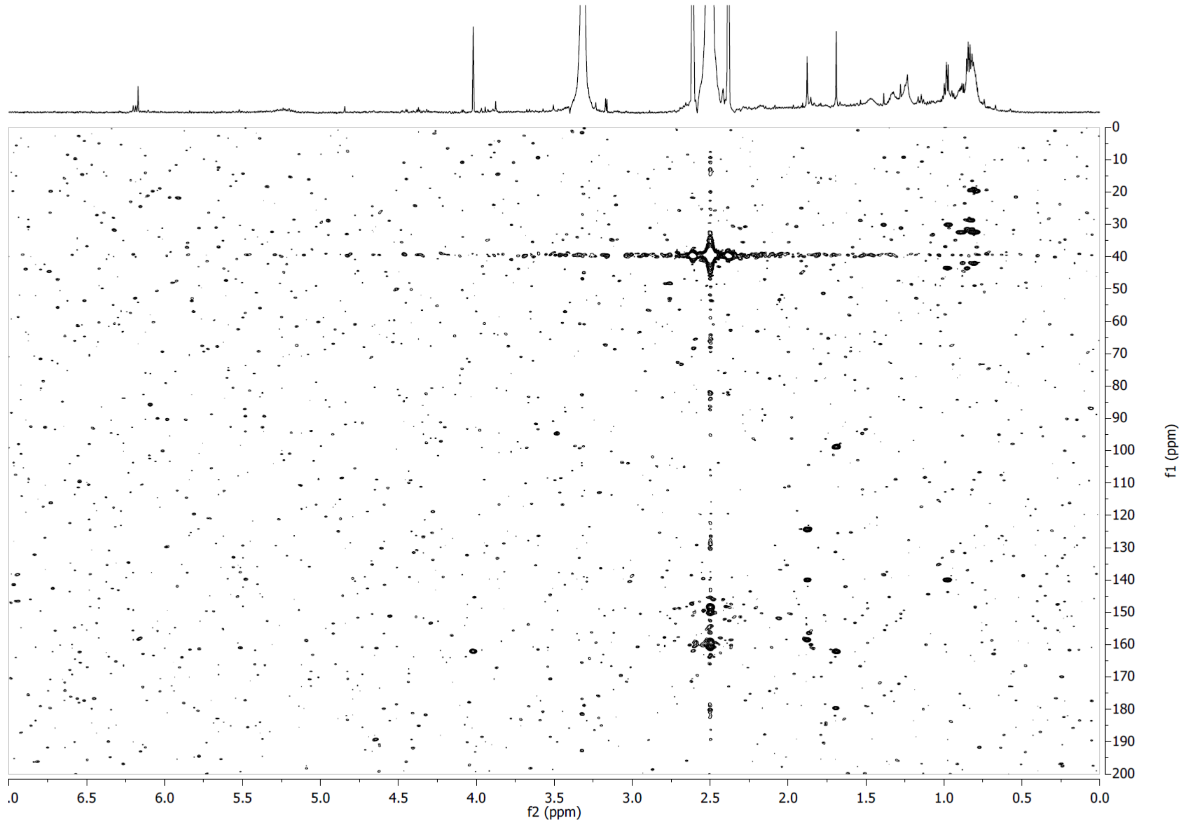


Figure S76: HMBC NMR spectrum of compound **17** in DMSO-$d_6$

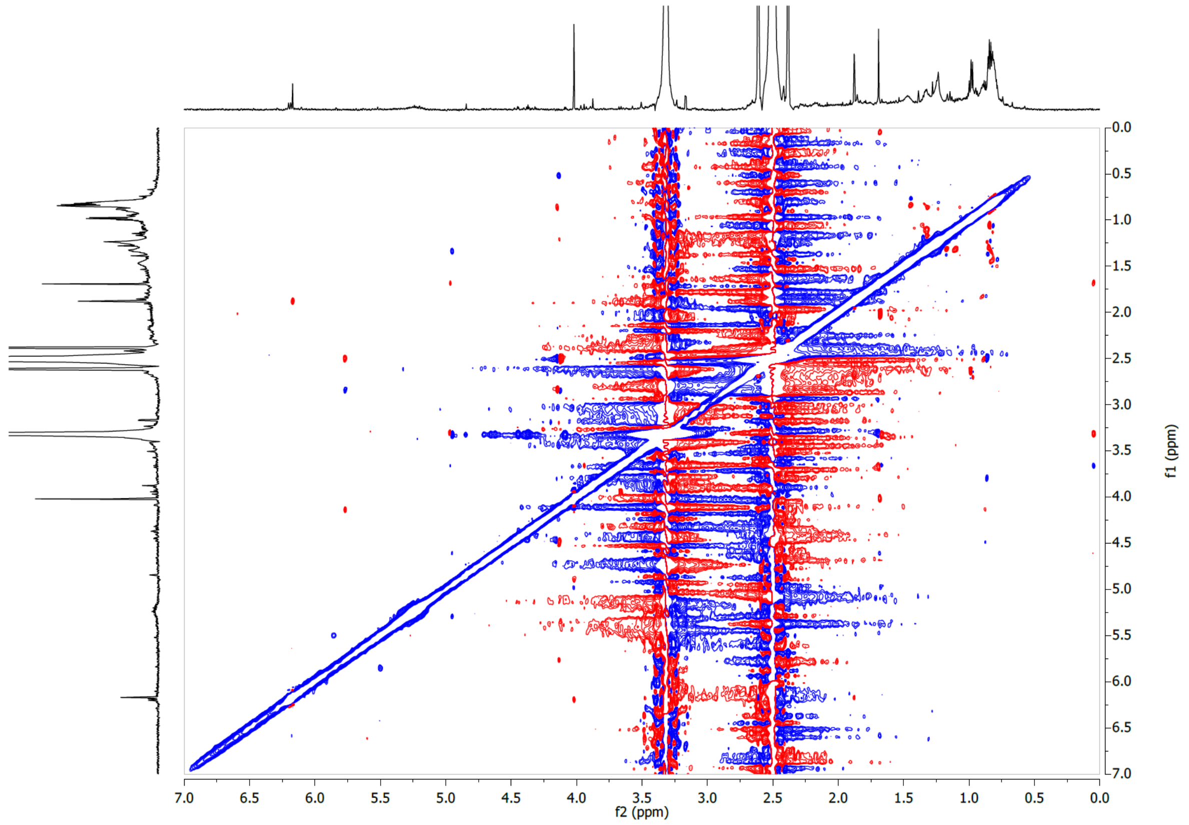

Figure S77: ROESY NMR spectrum of compound **17** in DMSO-$d_6$

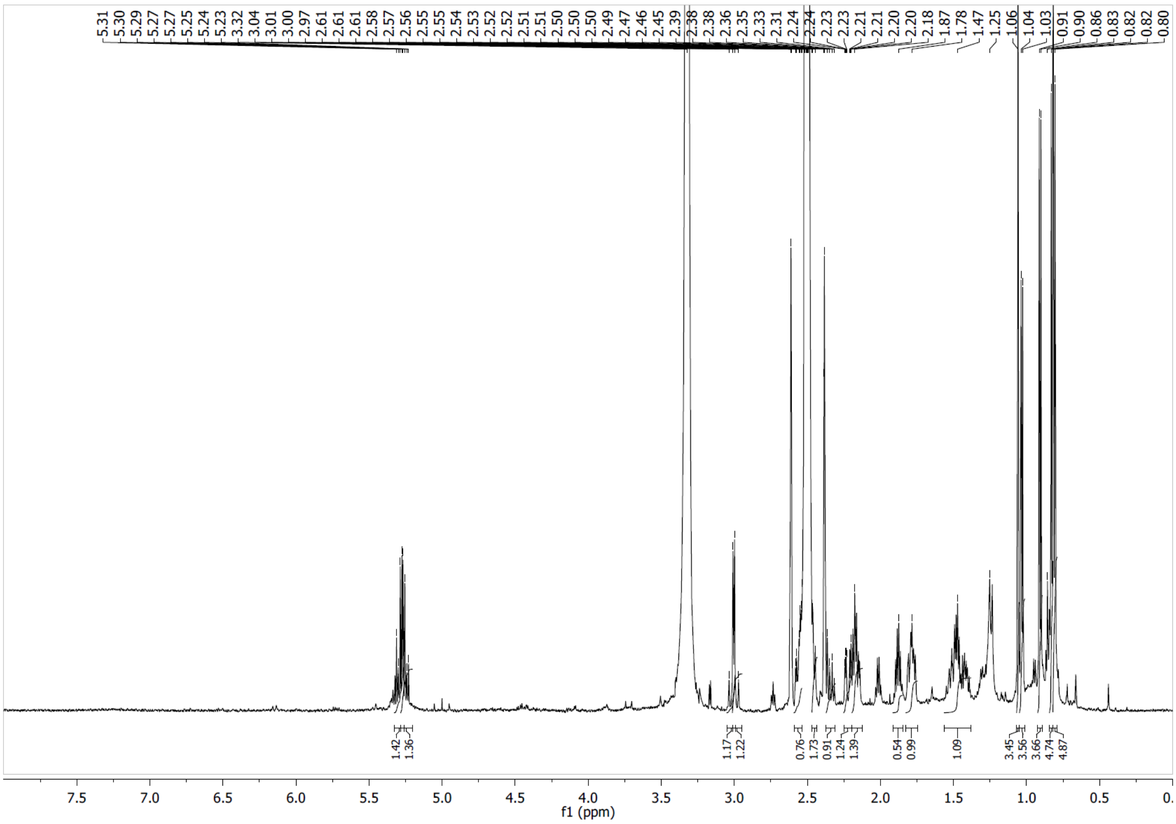


Figure S78: $^{1}$H NMR spectrum of compound **18** in DMSO-$d_6$ at 600 MHz

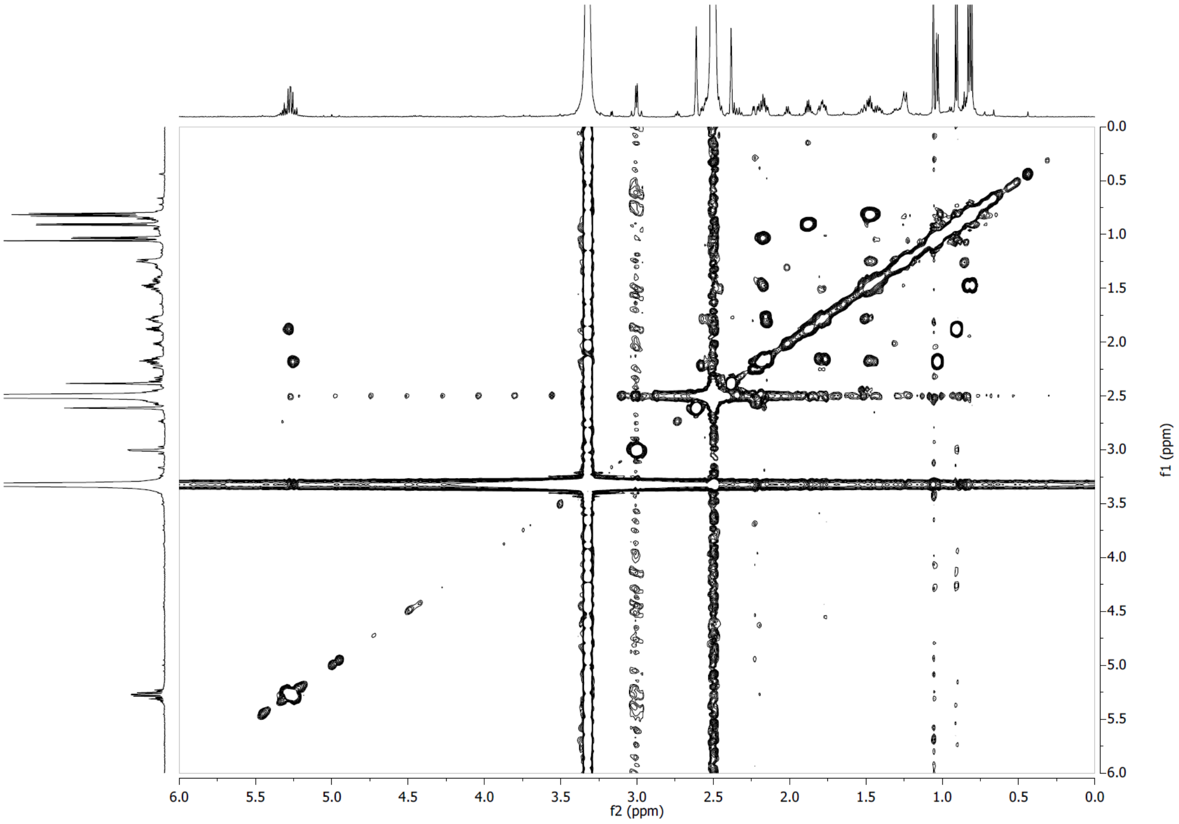

Figure S79: COSY NMR spectrum of compound **18** in DMSO-$d_6$

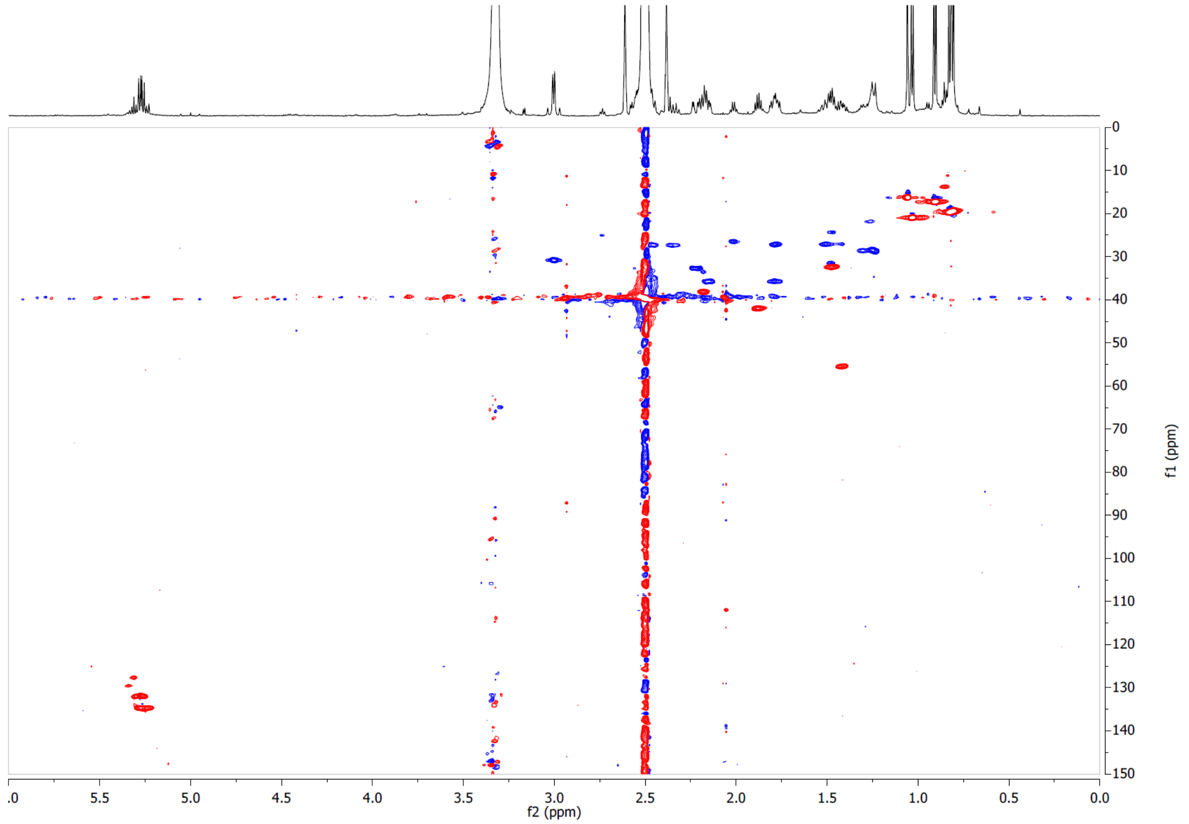


Figure S80: Edited-HSQC NMR spectrum of compound **18** in DMSO-$d_6$

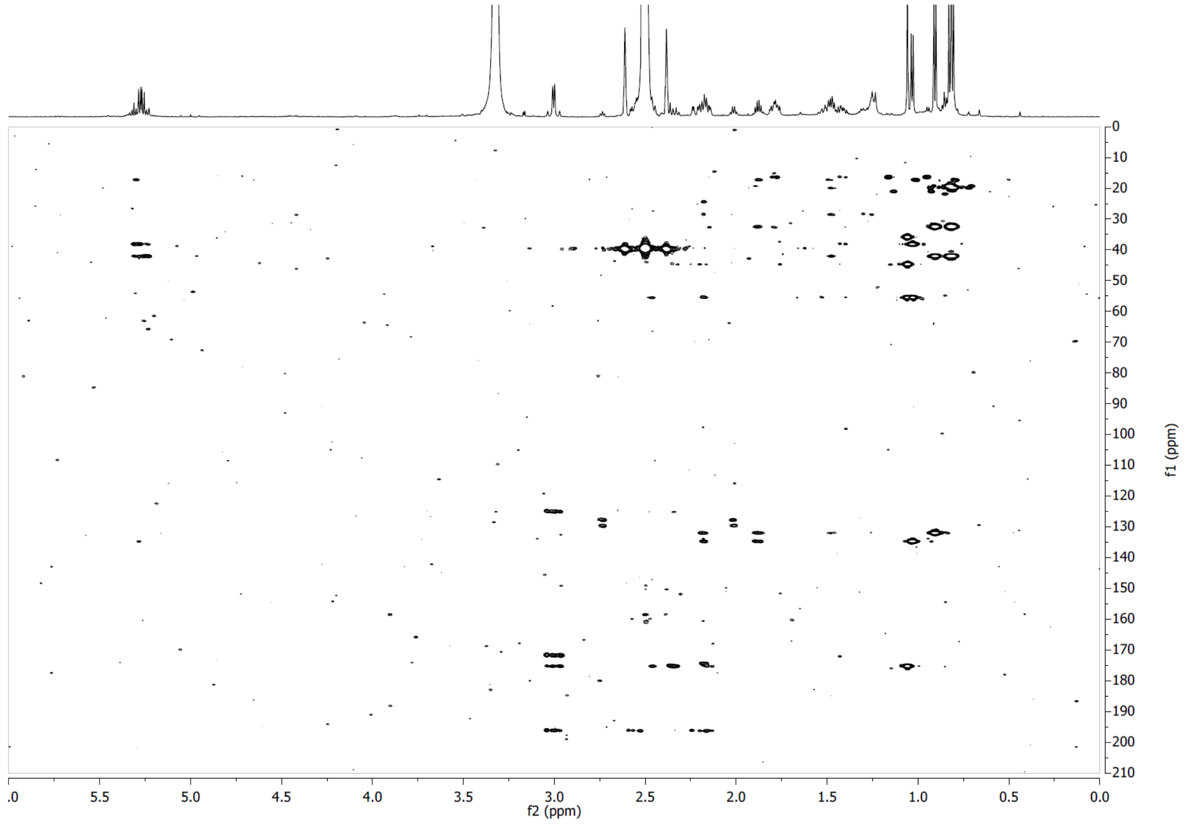

Figure S81: HMBC NMR spectrum of compound **18** in DMSO-$d_6$

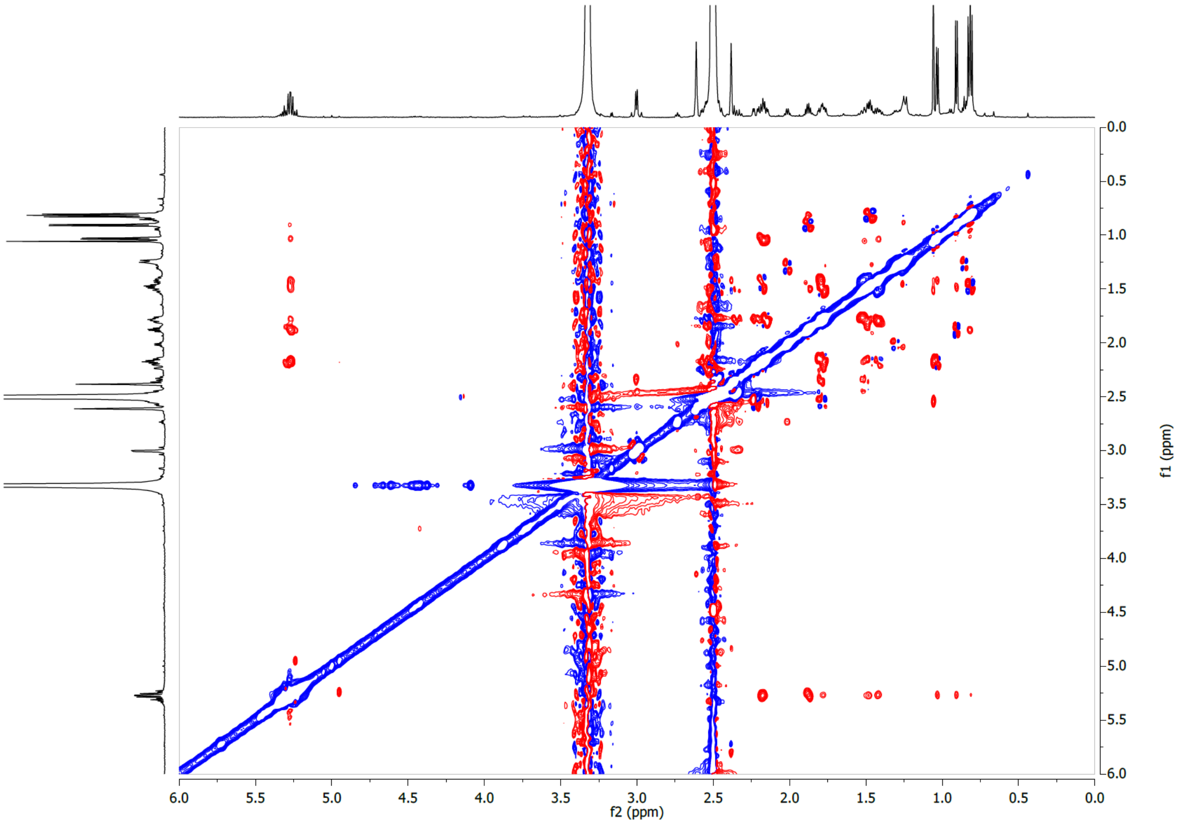


Figure S82: ROESY NMR spectrum of compound **18** in DMSO-$d_6$

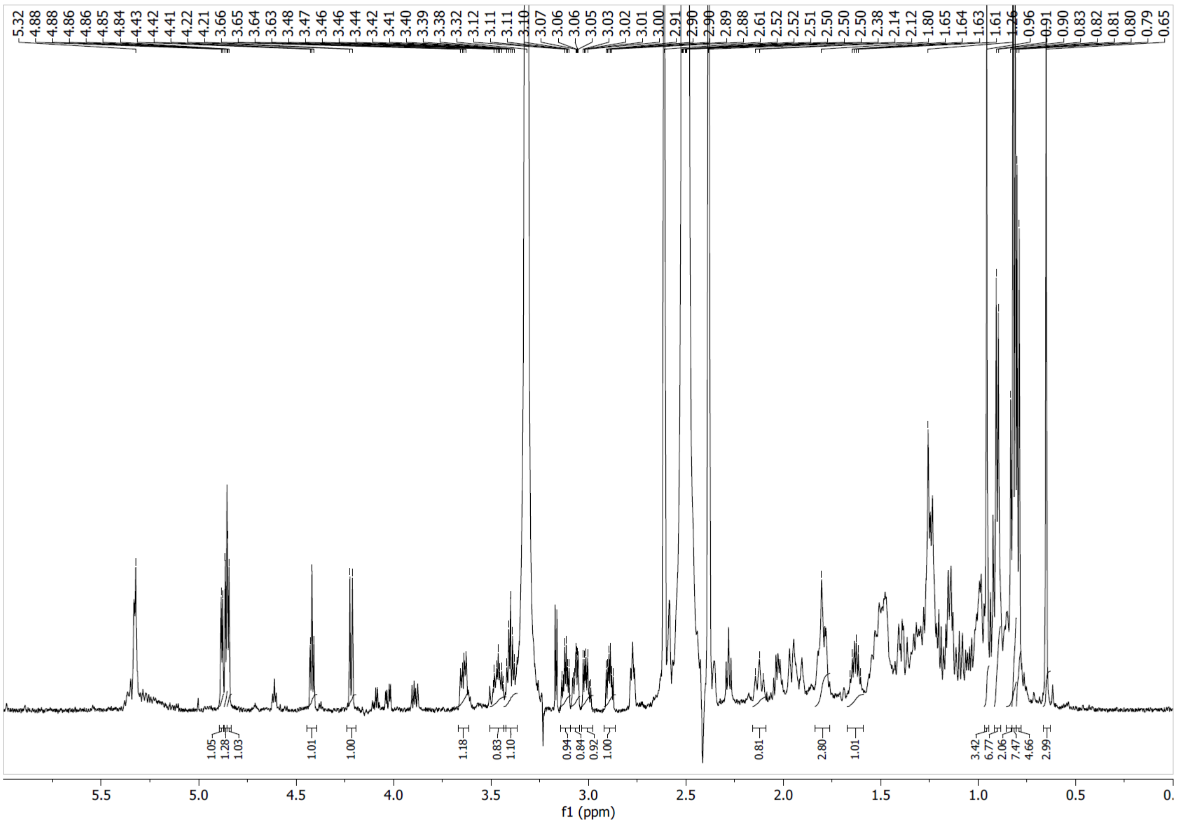

Figure S83: $^{1}$H NMR spectrum of compound **19** in DMSO-$d_6$ at 600 MHz

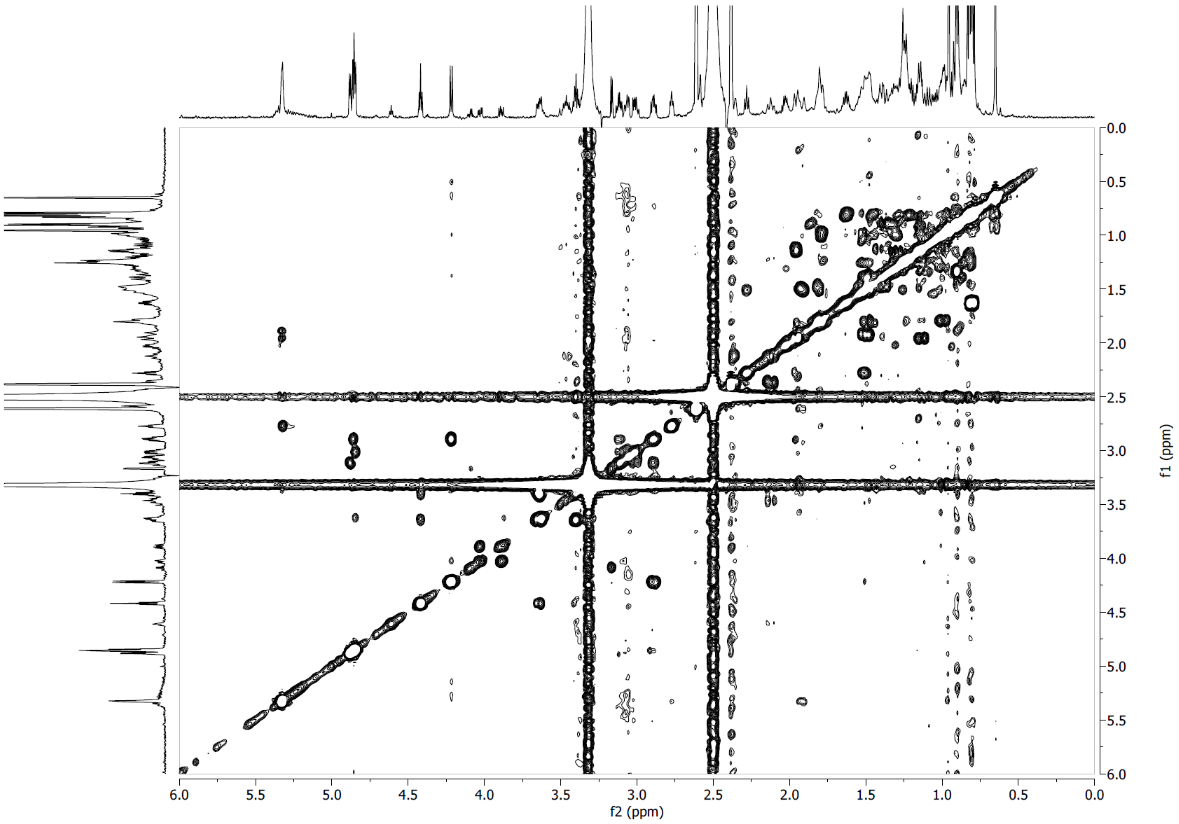


Figure S84: COSY NMR spectrum of compound **18** in DMSO-$d_6$

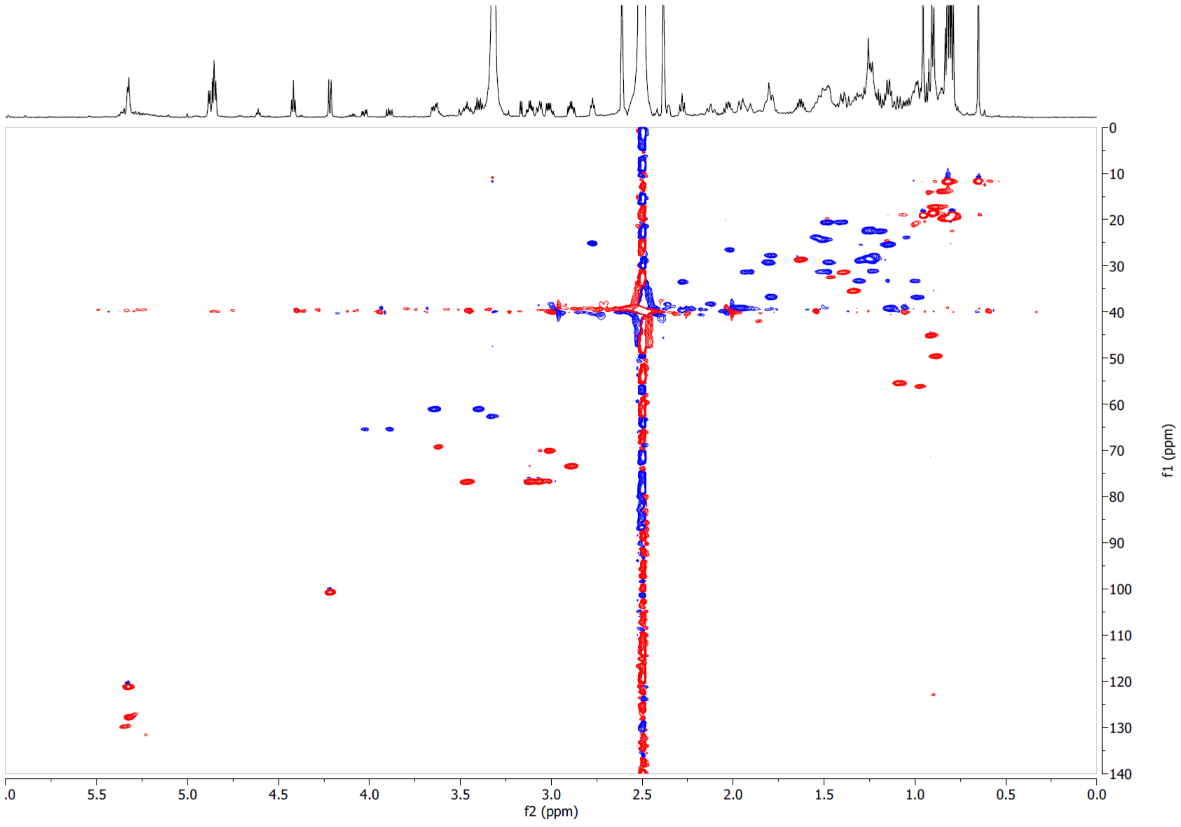

Figure S85: Edited-HSQC NMR spectrum of compound **18** in DMSO-$d_6$

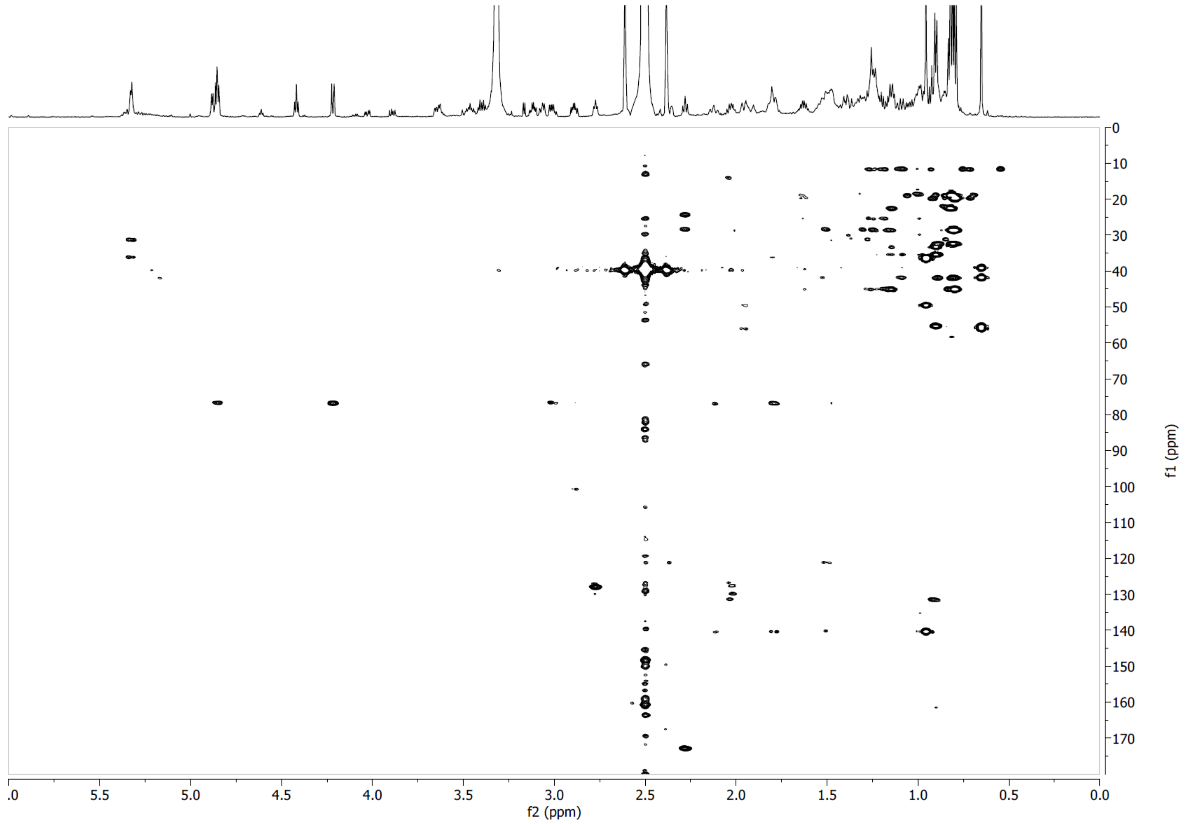


Figure S86: HMBC NMR spectrum of compound **18** in DMSO-$d_6$

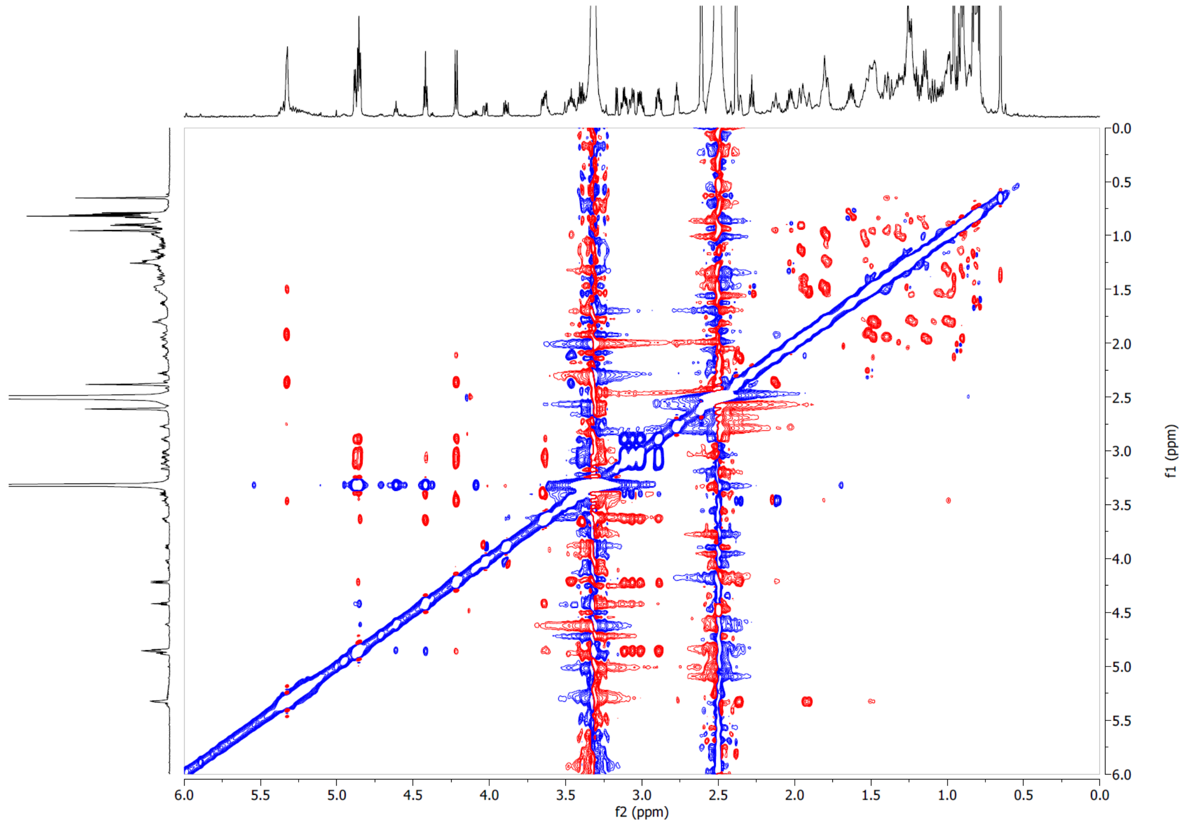

Figure S87: ROESY NMR spectrum of compound **18** in DMSO-$d_6$

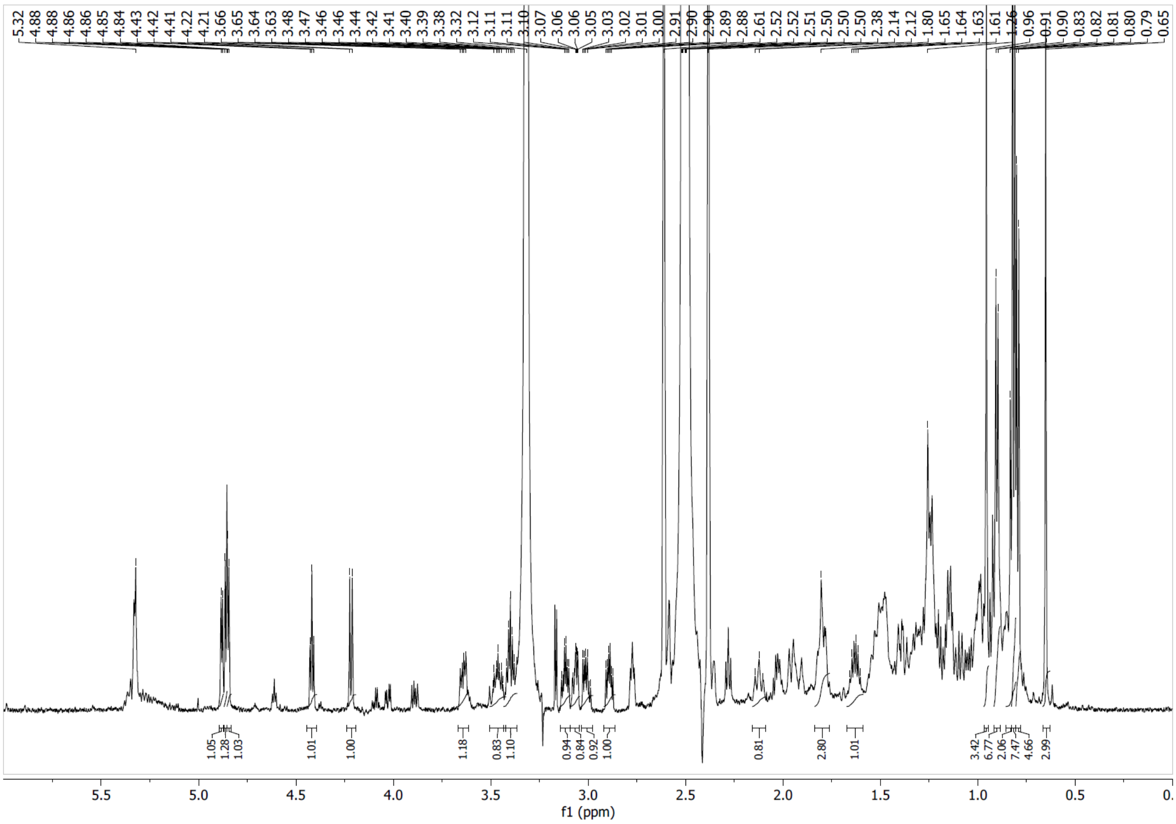


Figure S88: $^{1}$H NMR spectrum of compound **19** in DMSO-$d_6$ at 600 MHz

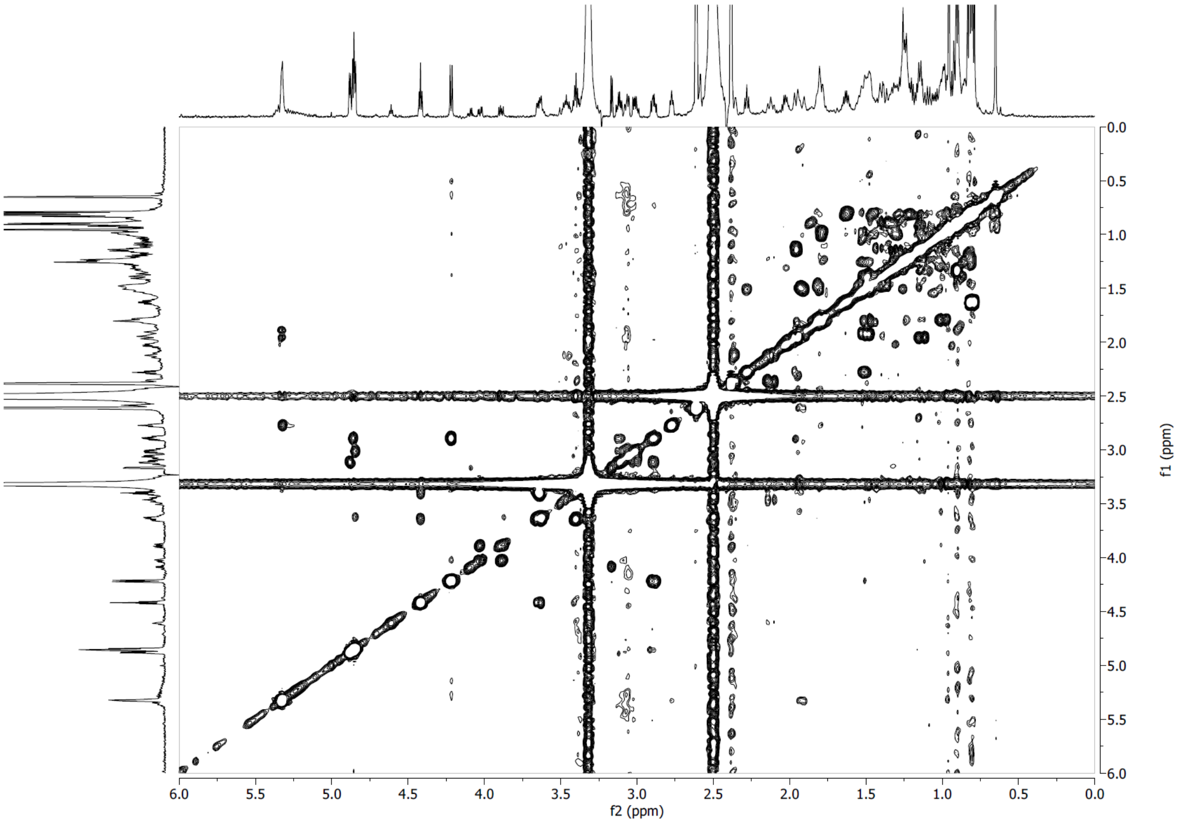

Figure S89: COSY NMR spectrum of compound **19** in DMSO-$d_6$

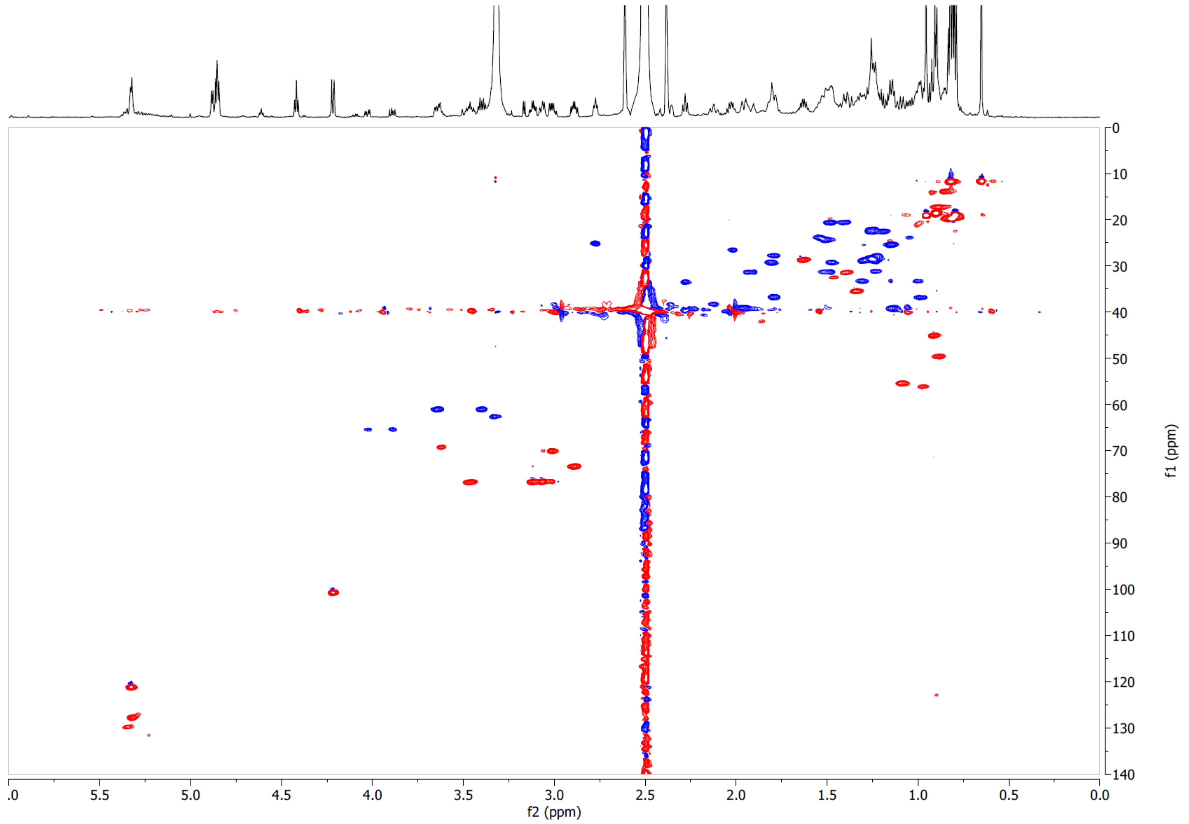


Figure S90: Edited-HSQC NMR spectrum of compound **19** in DMSO-$d_6$

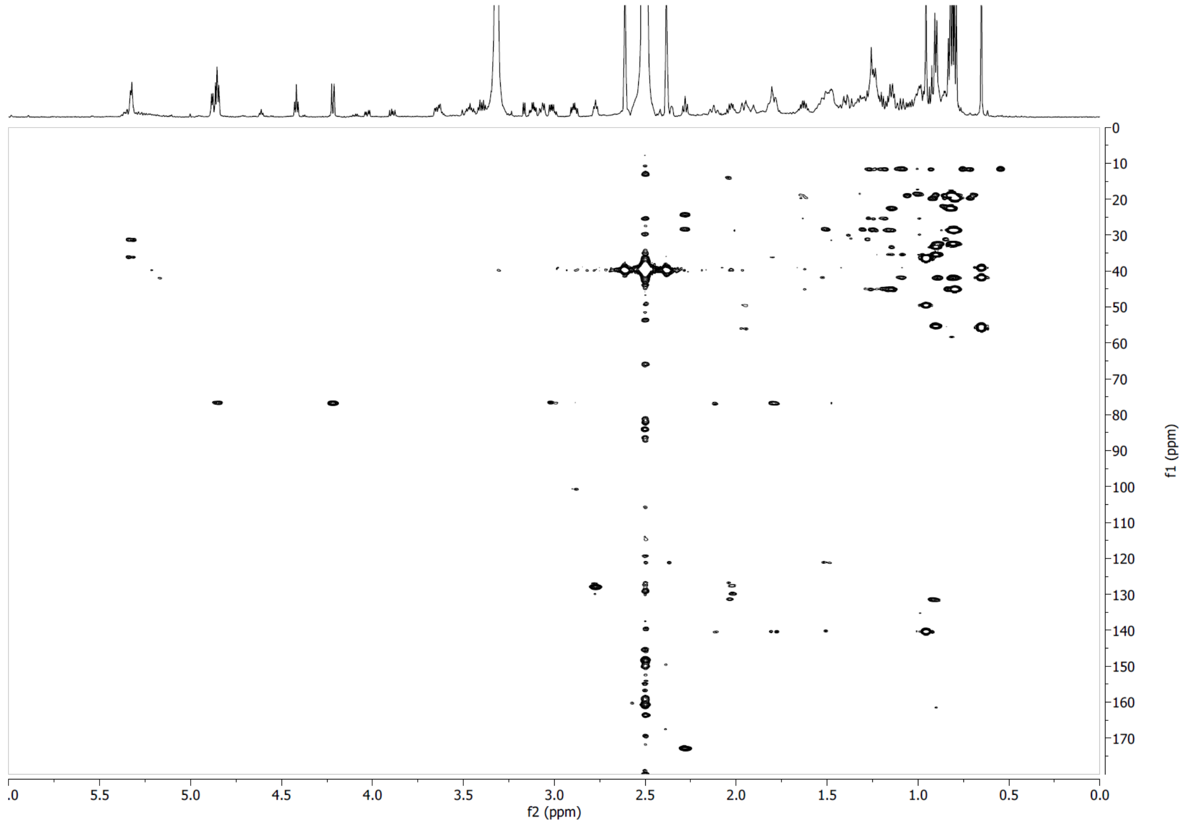

Figure S91: HMBC NMR spectrum of compound **19** in DMSO-$d_6$

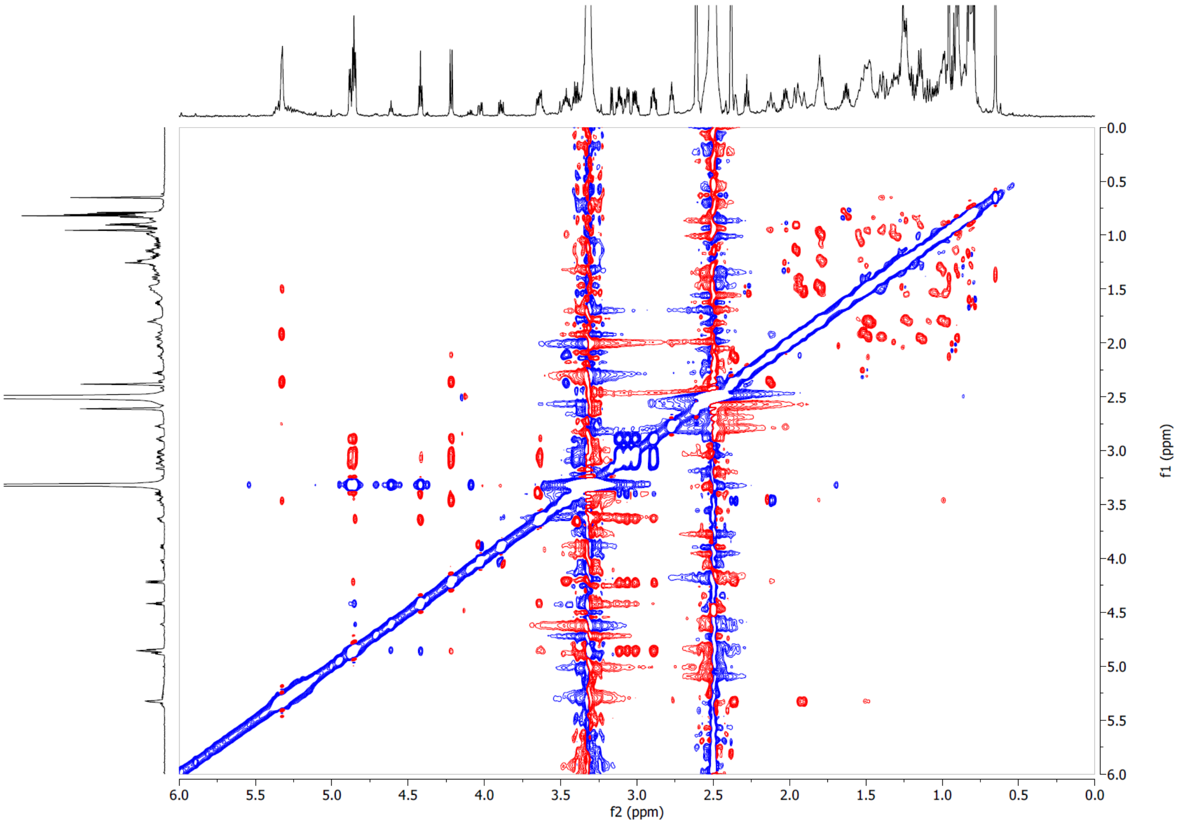

Figure S92: ROESY NMR spectrum of compound **19** in DMSO-$d_6$

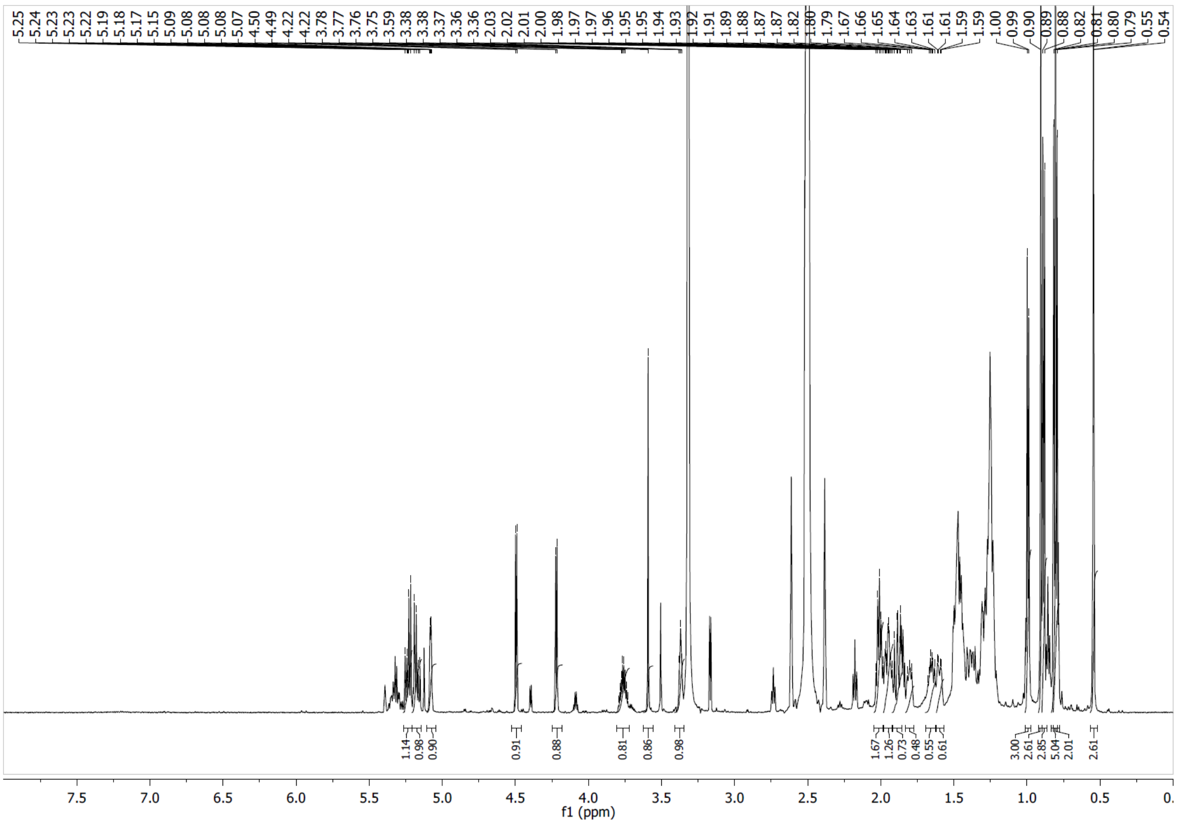

Figure S93: $^{1}$H NMR spectrum of compound **20** in DMSO-$d_6$ at 600 MHz

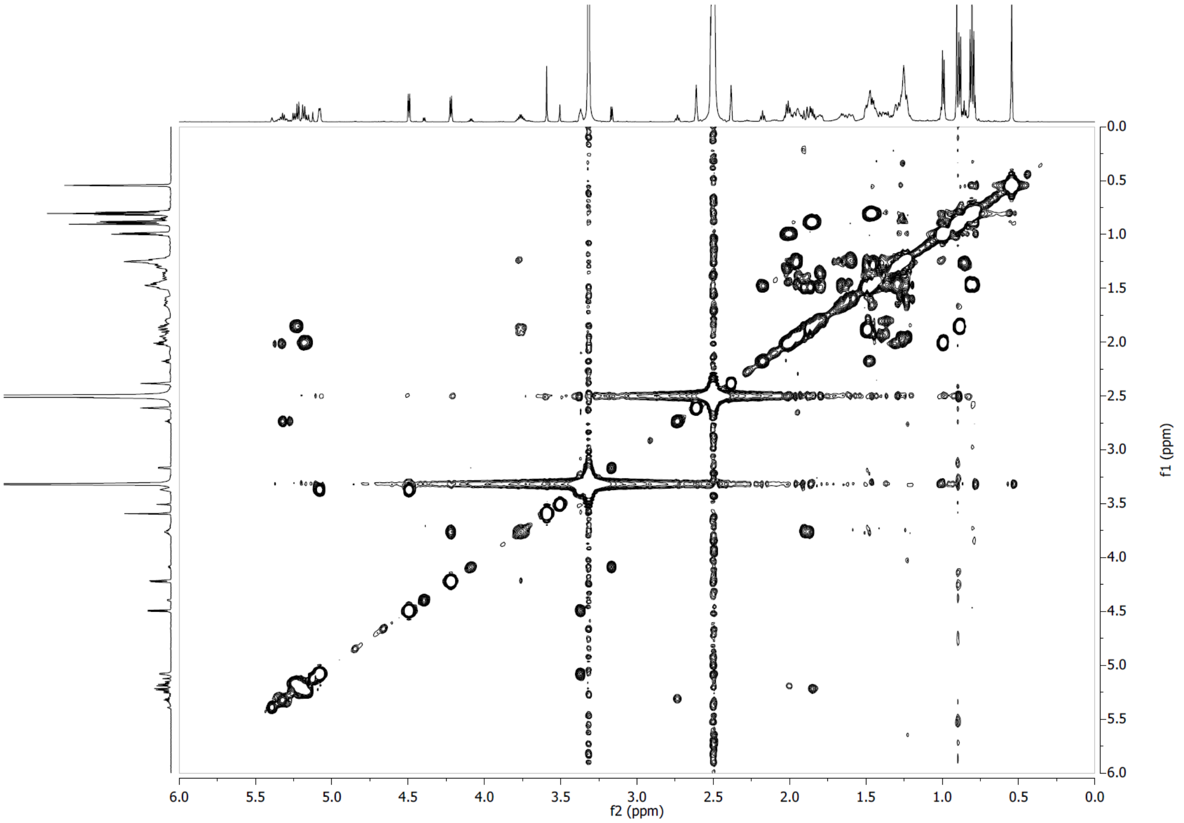


Figure S94: COSY NMR spectrum of compound **20** in DMSO-$d_6$

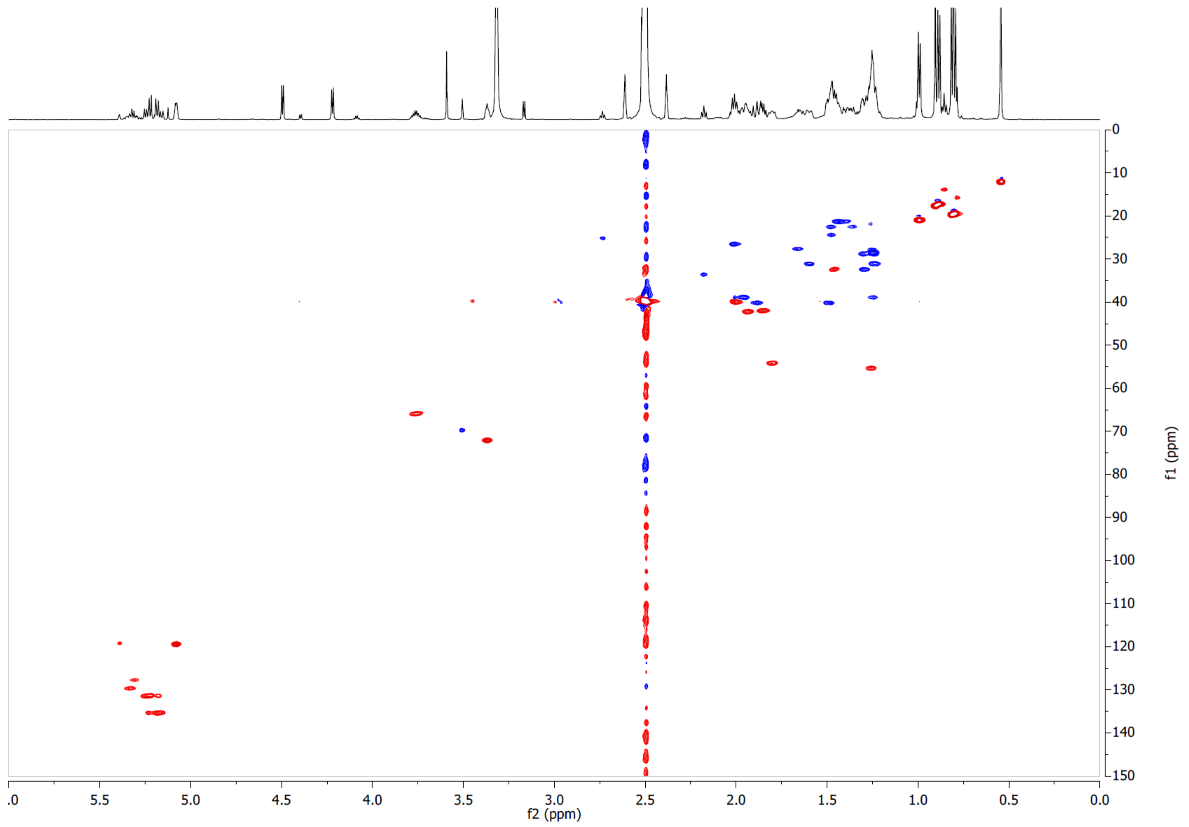

Figure S95: Edited-HSQC NMR spectrum of compound **20** in DMSO-$d_6$

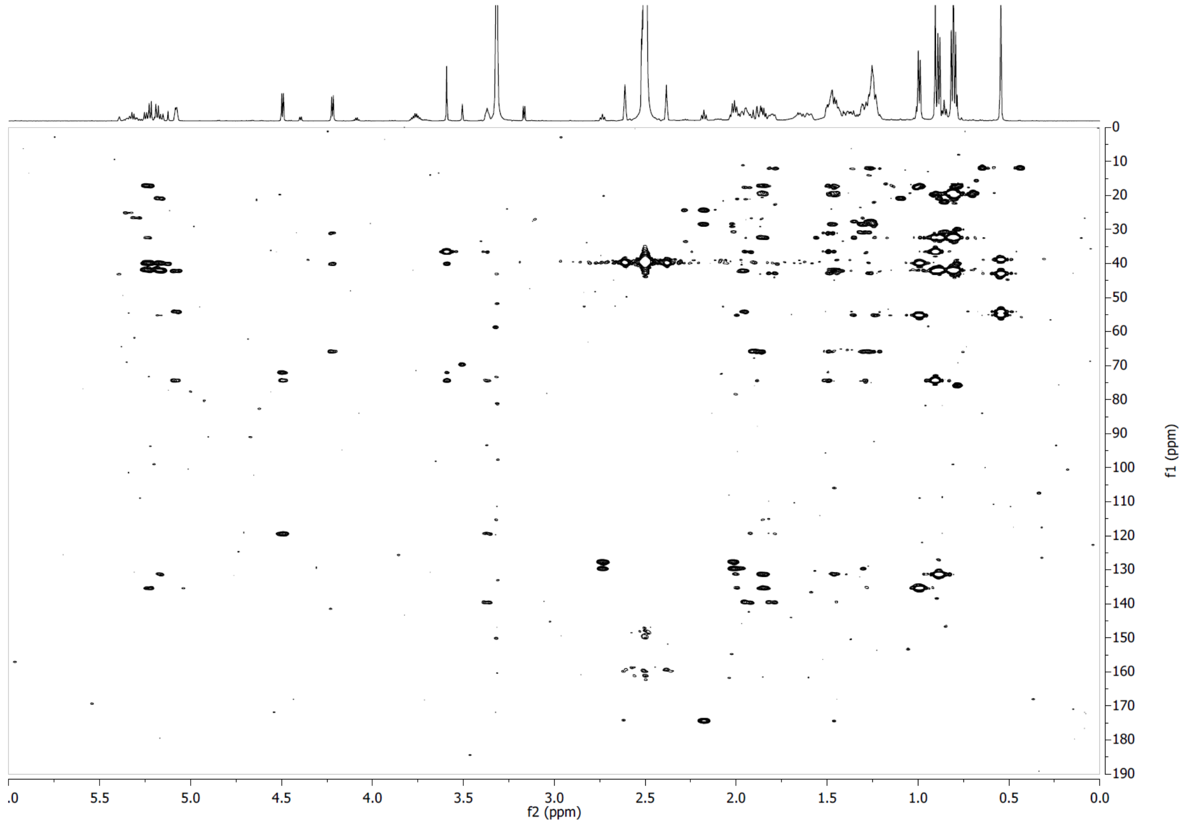


Figure S96: HMBC NMR spectrum of compound **20** in DMSO-$d_6$

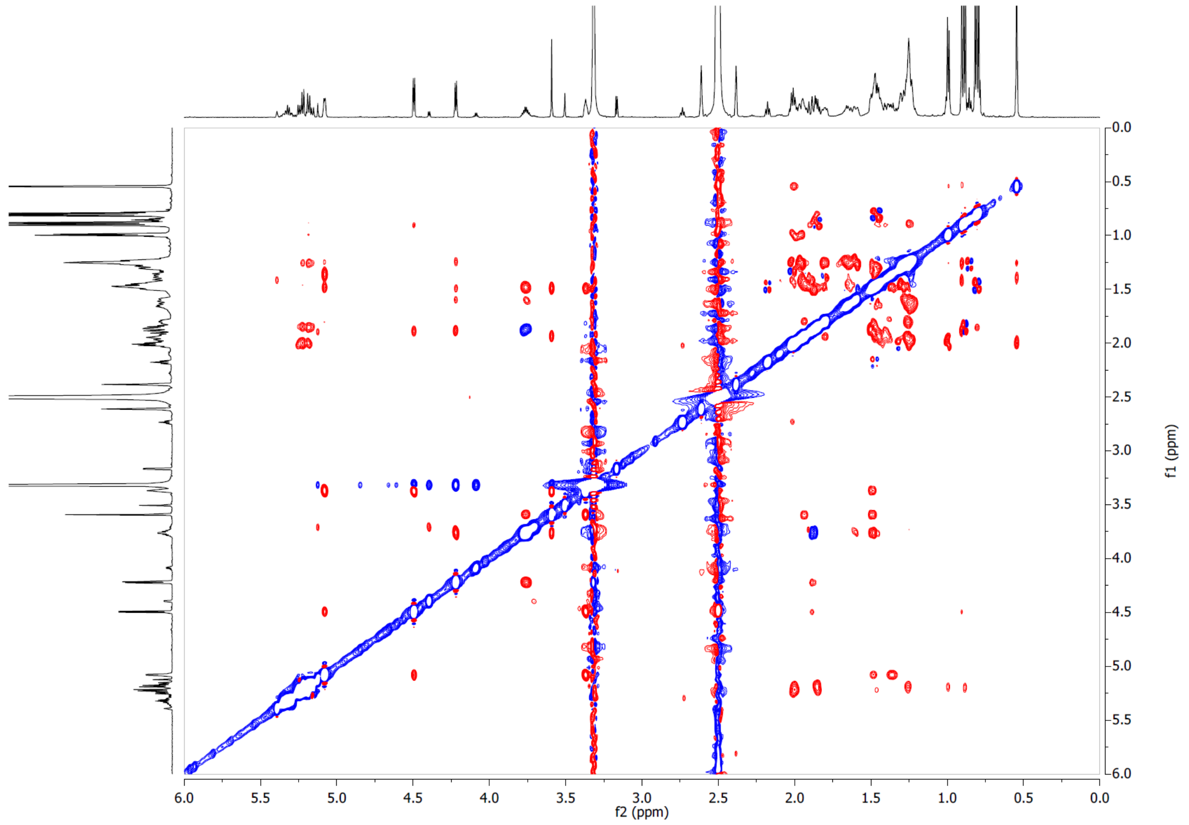

Figure S97: ROESY NMR spectrum of compound **20** in DMSO-$d_6$

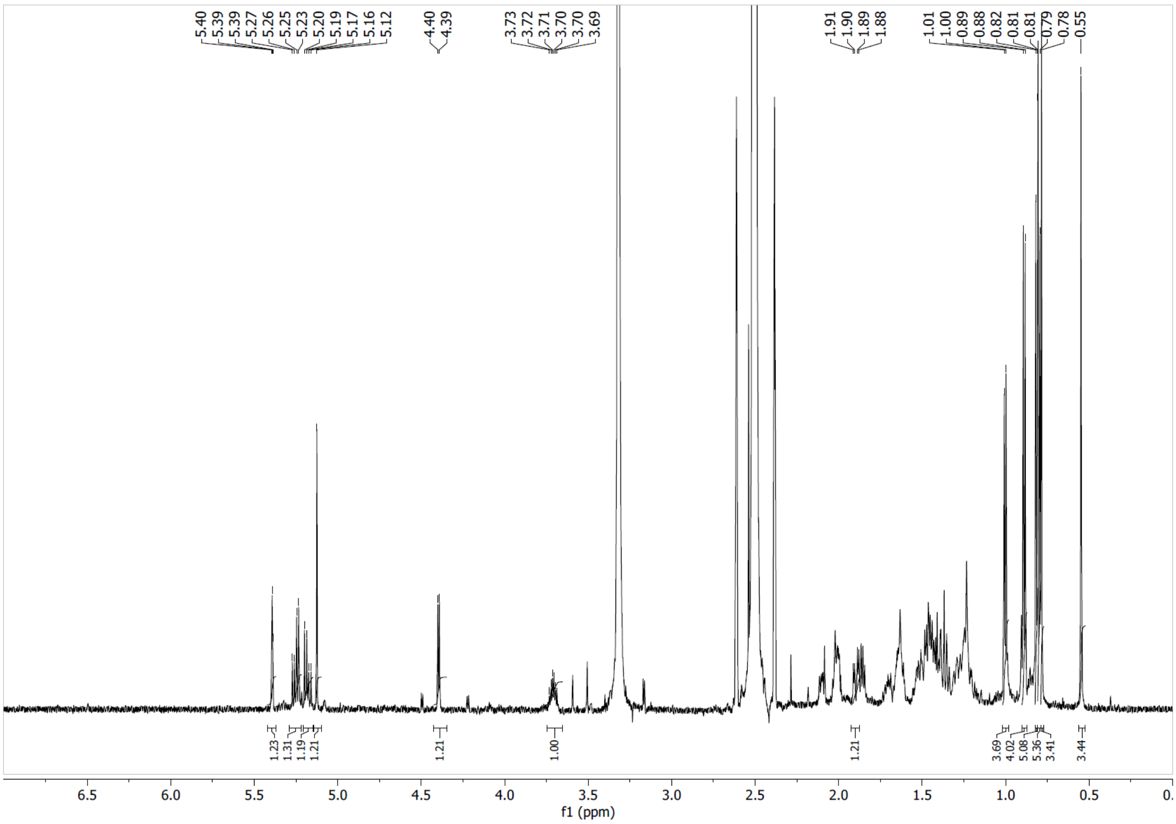


Figure S98: $^{1}$H NMR spectrum of compound **21** in DMSO-$d_6$ at 600 MHz

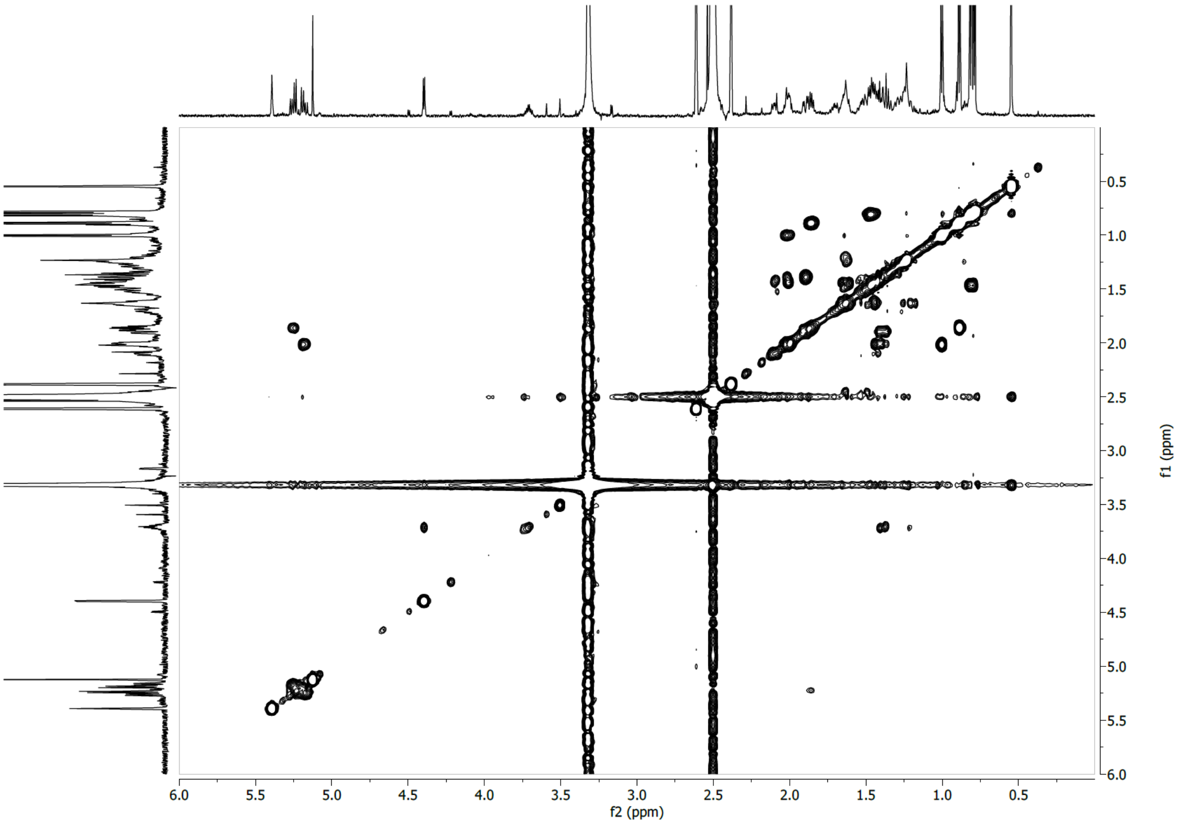

Figure S99: COSY NMR spectrum of compound **21** in DMSO-$d_6$

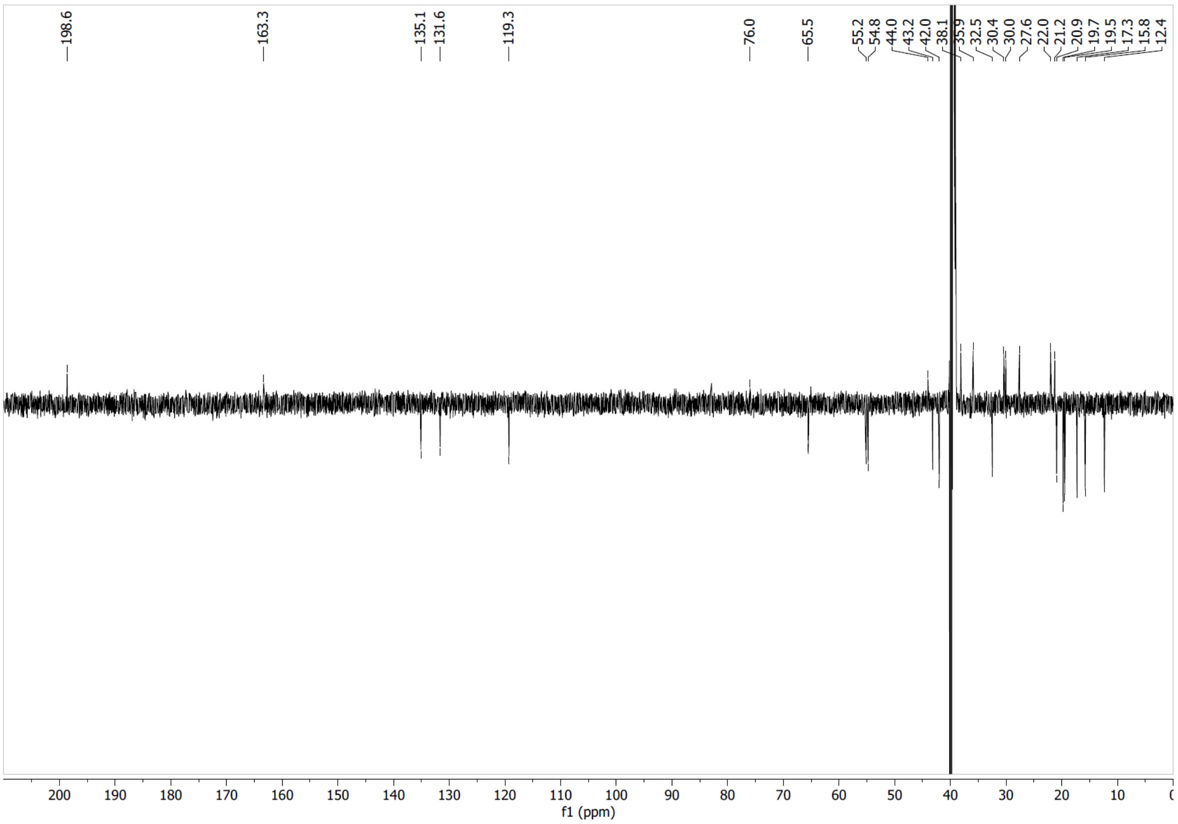


Figure S100: $^{13}$C-DEPTQ NMR spectrum of compound **21** in DMSO-$d_6$ at 151 MHz

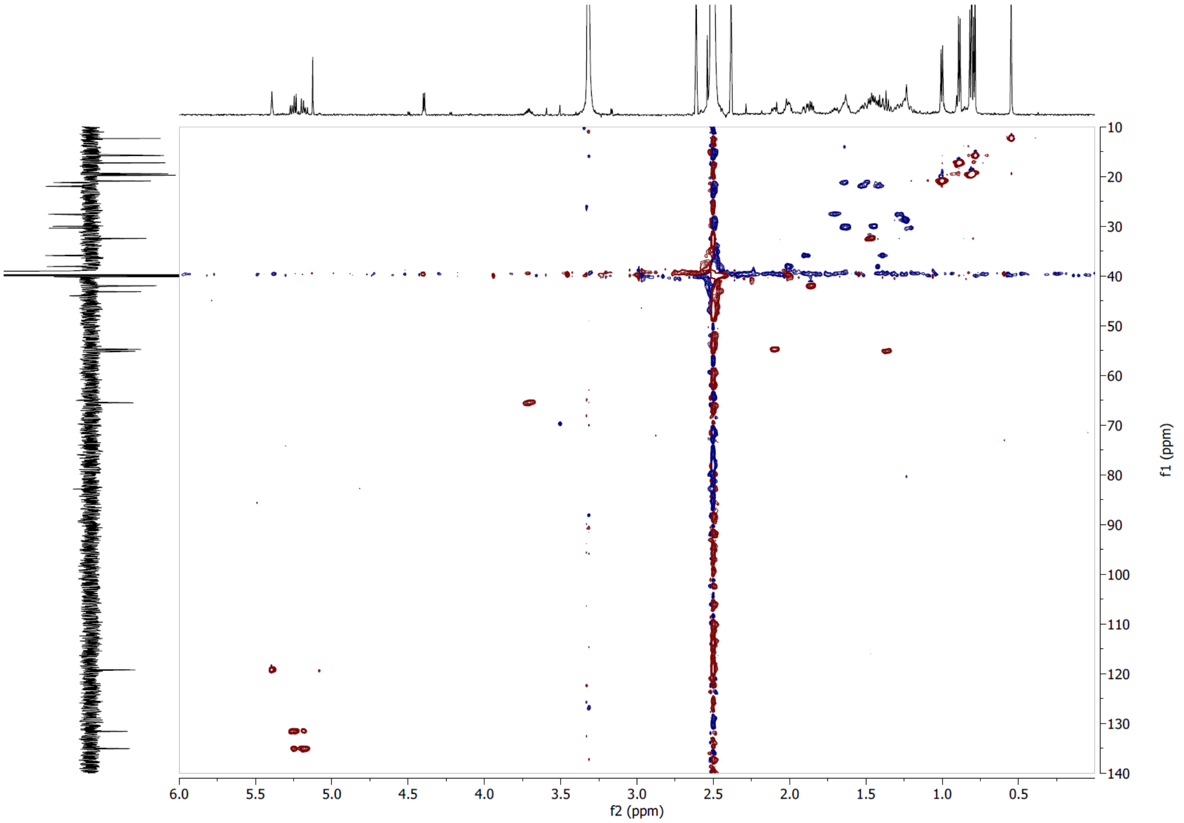

Figure S101: Edited-HSQC NMR spectrum of compound **21** in DMSO-$d_6$

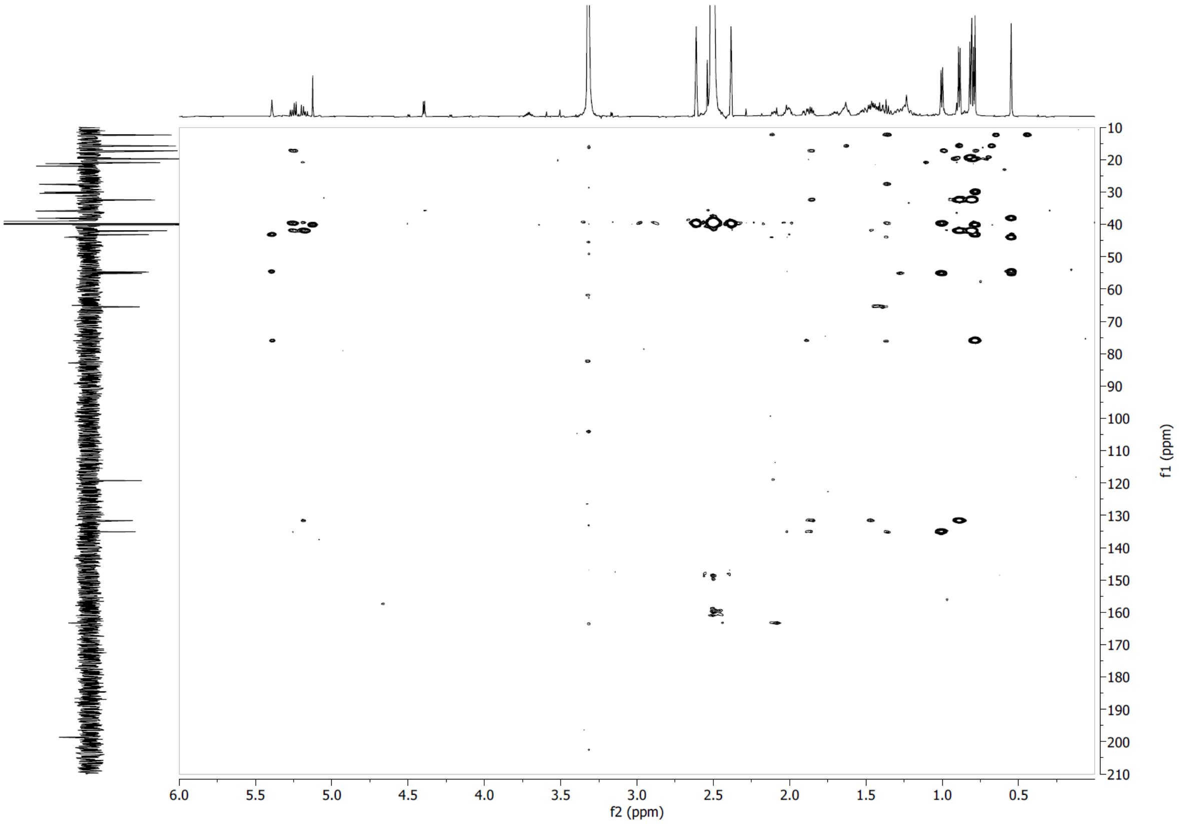


Figure S102: HMBC NMR spectrum of compound **21** in DMSO-$d_6$

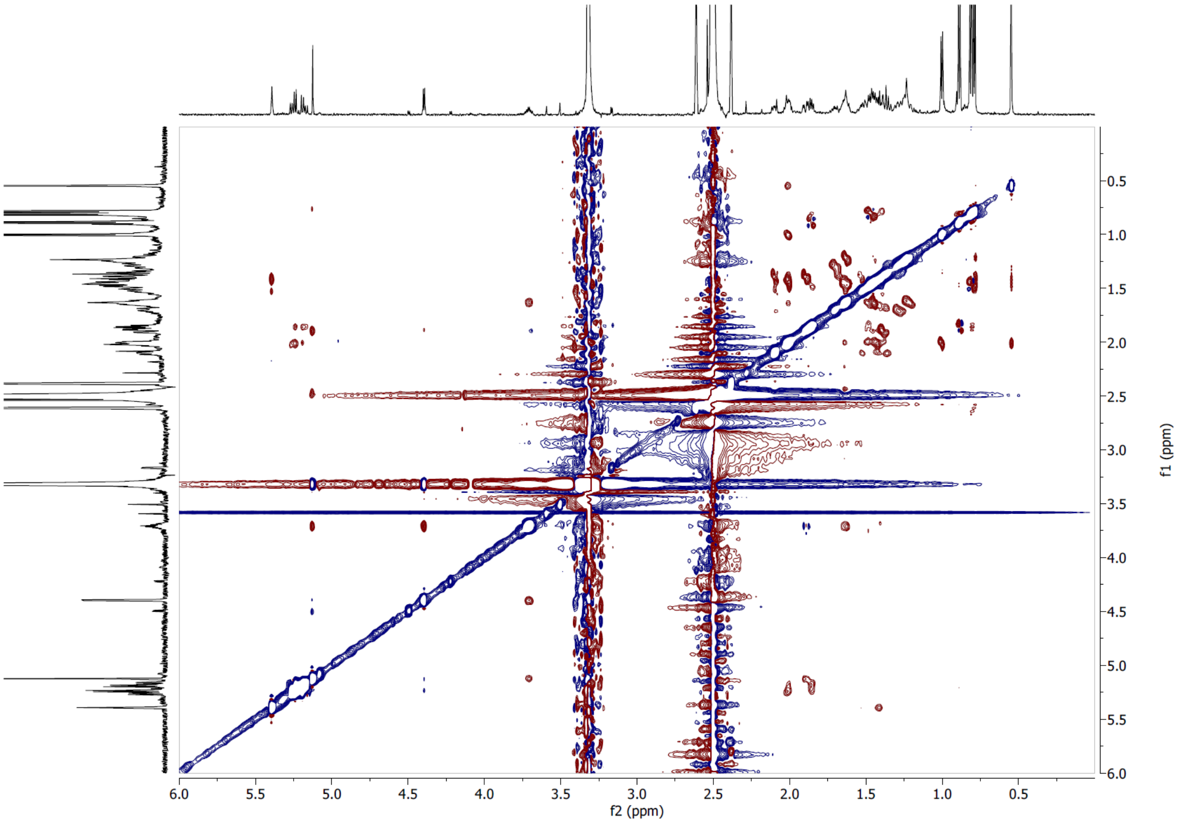

Figure S103: ROESY NMR spectrum of compound **21** in DMSO-$d_6$

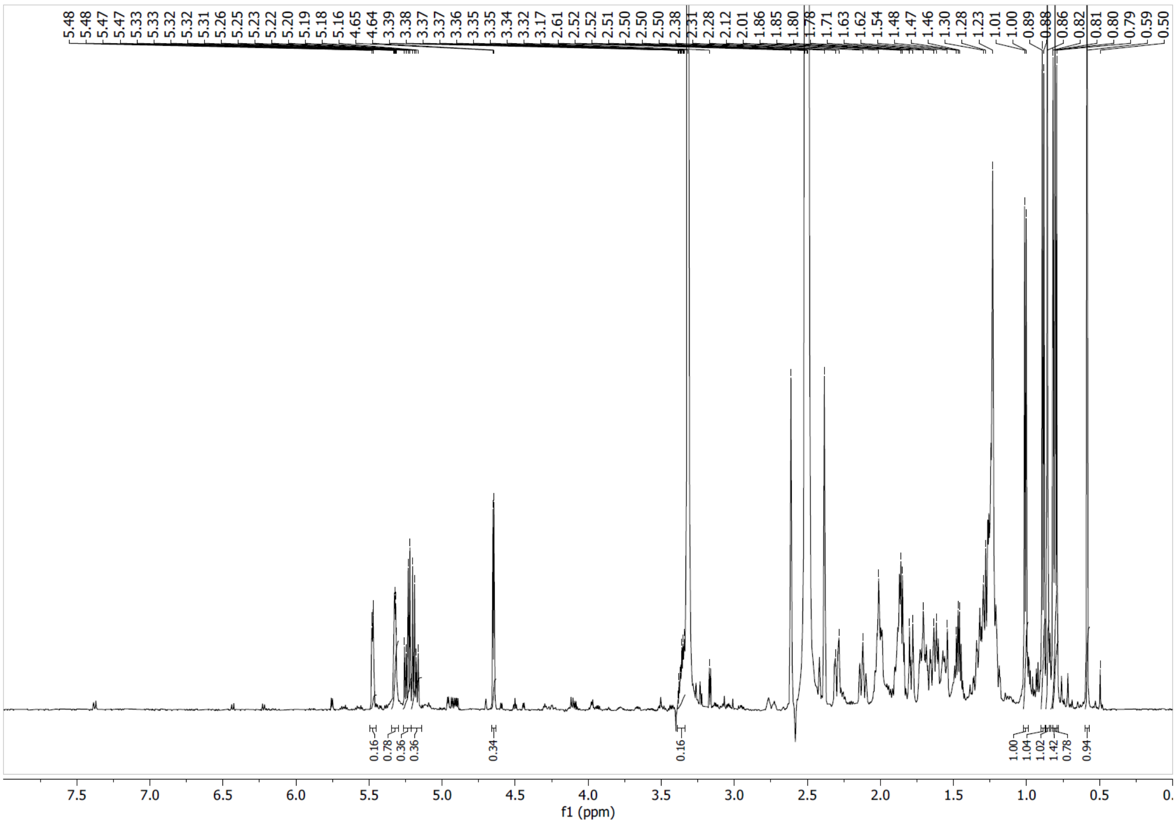


Figure S104: $^{1}$H NMR spectrum of compound **22** in DMSO-$d_6$ at 600 MHz

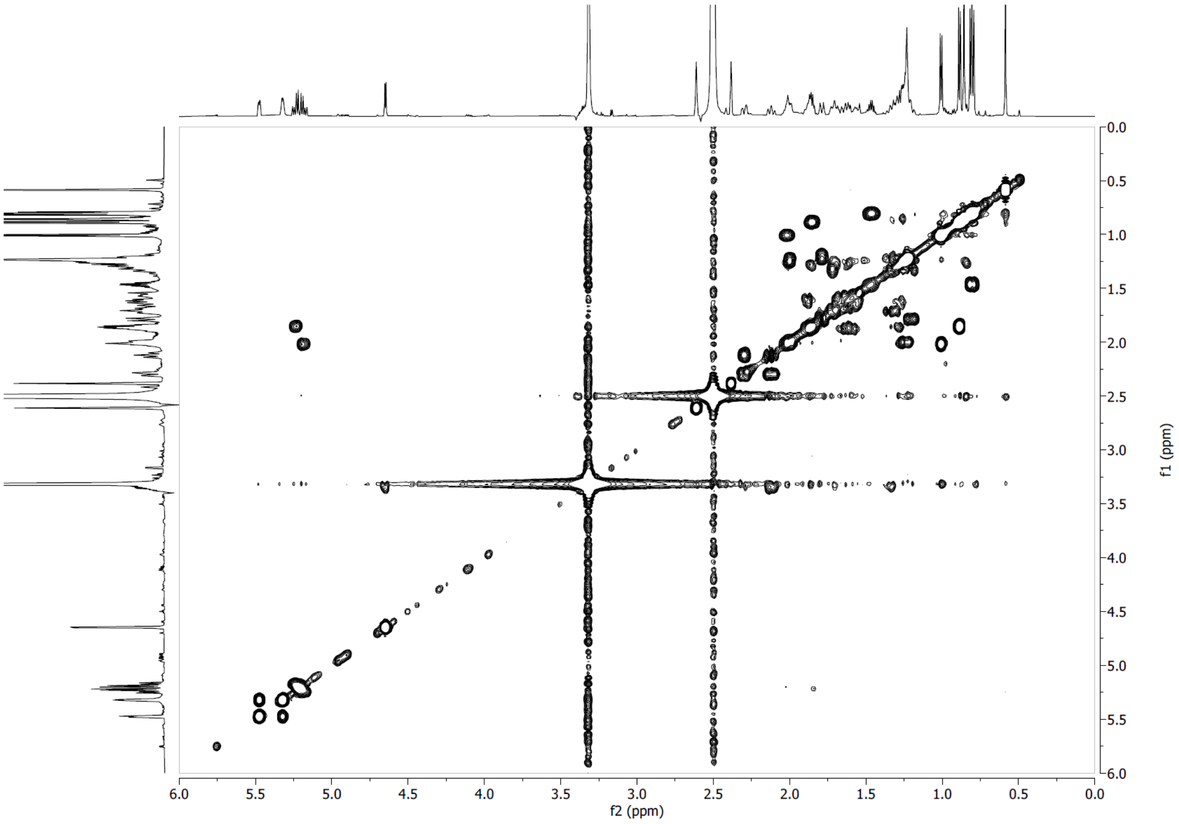

Figure S105: COSY NMR spectrum of compound **22** in DMSO-$d_6$

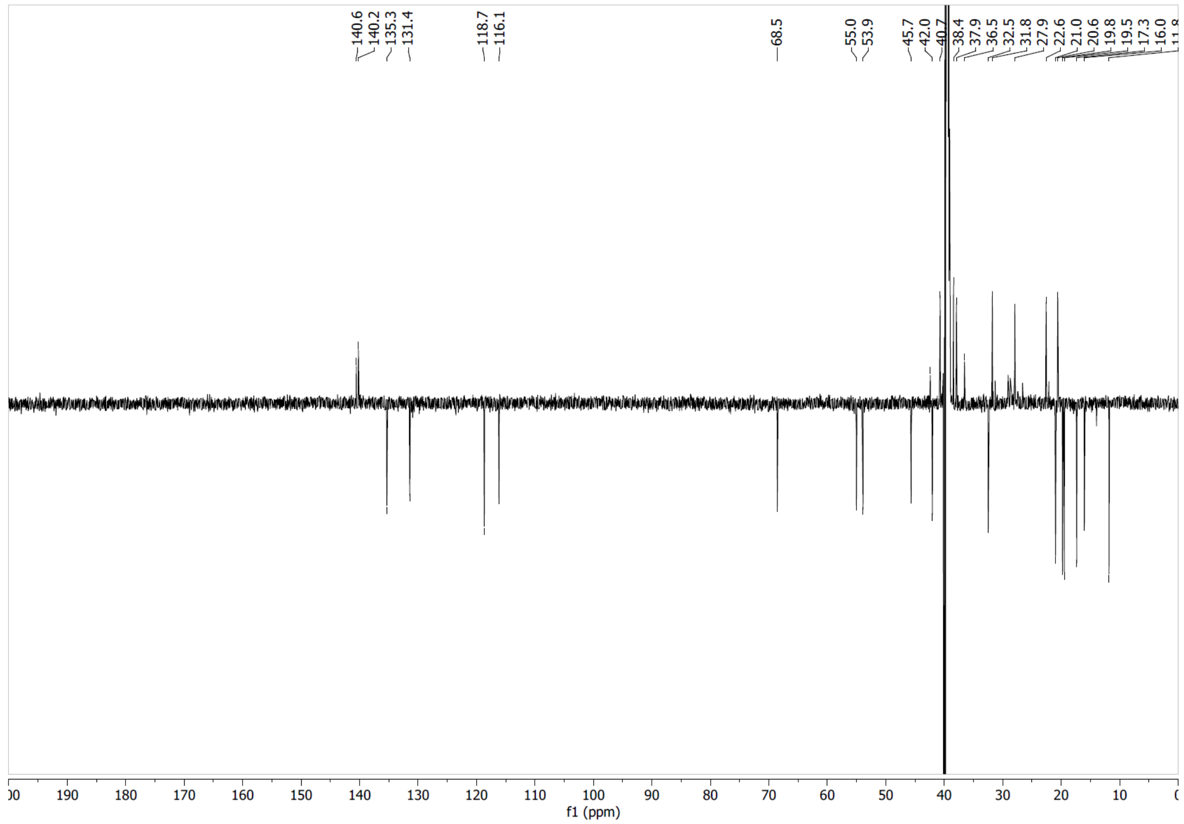


Figure S106: $^{13}$C-DEPTQ NMR spectrum of compound **22** in DMSO-$d_6$ at 151 MHz

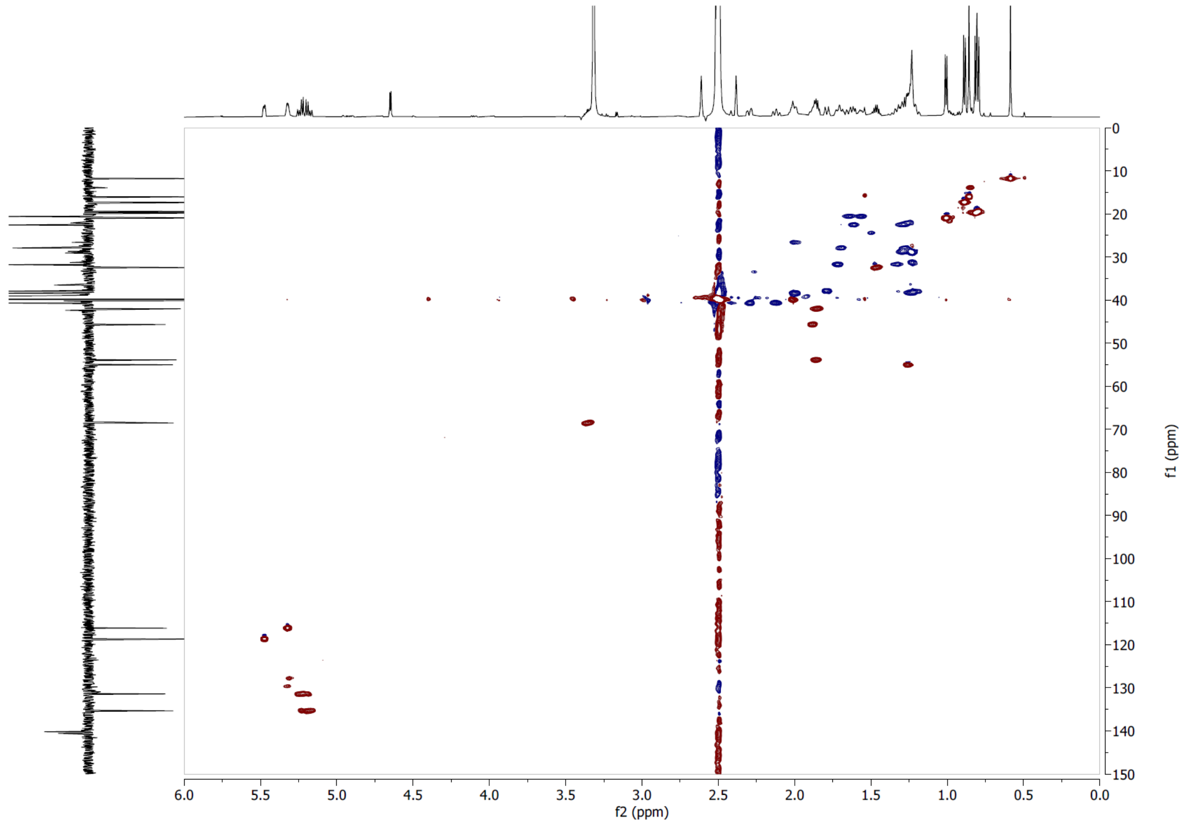

Figure S107: Edited-HSQC NMR spectrum of compound **22** in DMSO-$d_6$

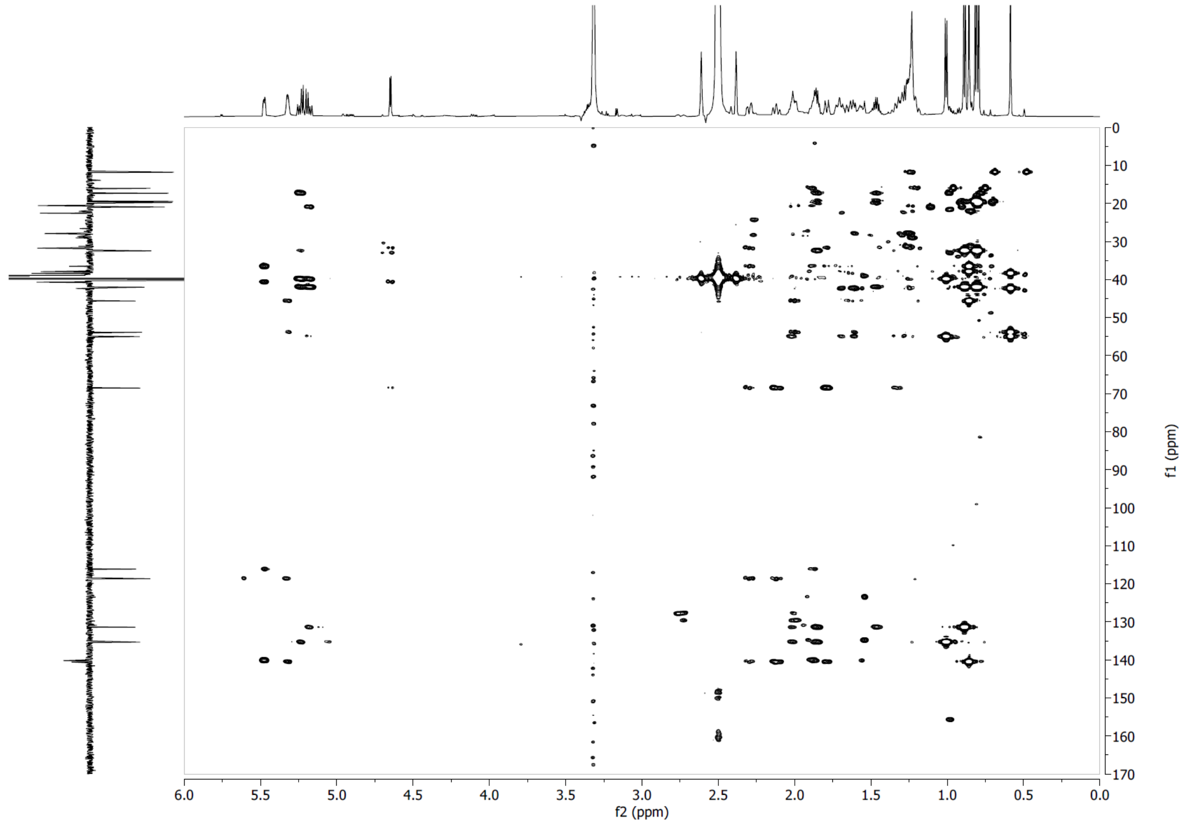


Figure S108: HMBC NMR spectrum of compound **22** in DMSO-$d_6$

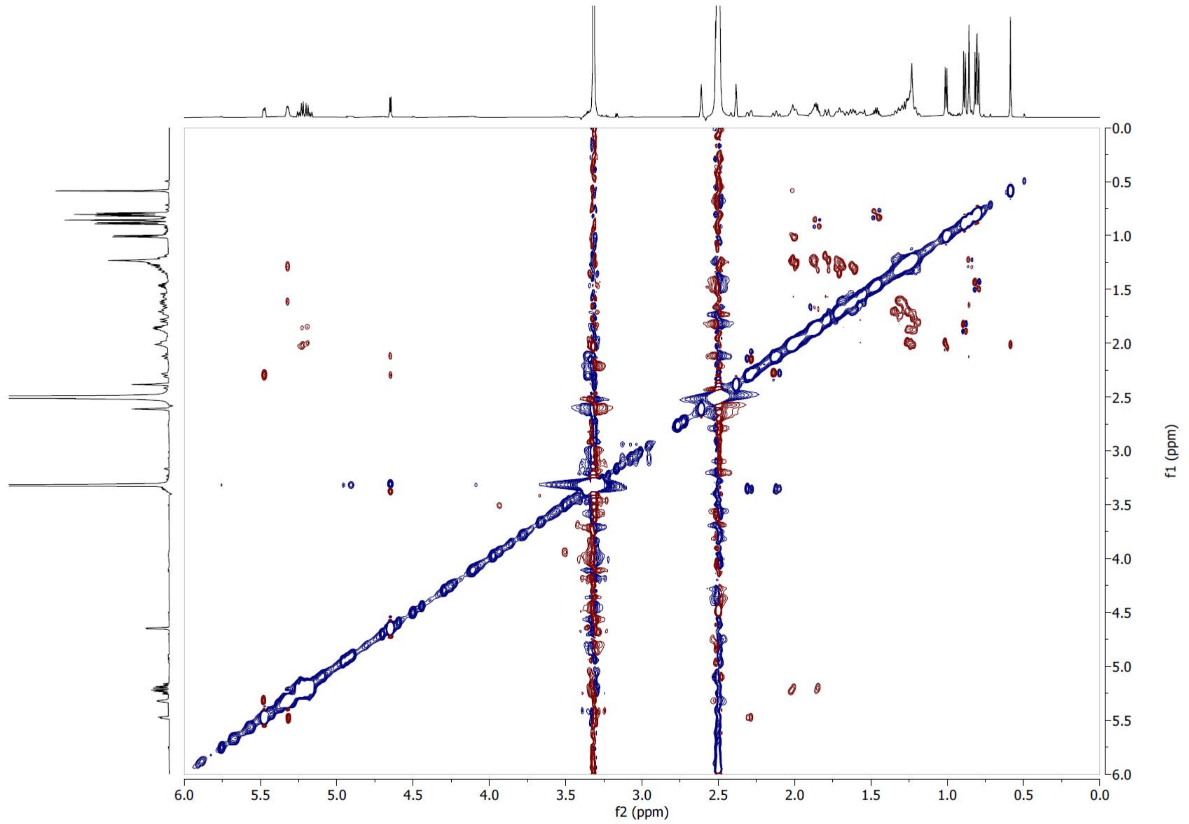

Figure S109: ROESY NMR spectrum of compound **22** in DMSO-$d_6$
