# Supplementary material for: Combination of Pseudo-LC-NMR and HRMS/MS-Based Molecular Networking for the Rapid Identification of Antimicrobial Metabolites From Fusarium petroliphilum
Source: Front Mol Biosci. 2021 Oct 22;8:725691. doi: 10.3389/fmolb.2021.725691 (PMC8569130; doi:10.3389/fmolb.2021.725691)
Supplement: Supplementary file 2 [file DataSheet3.pdf]

Supplementary Table S1. Voucher table for the *Fusarium* (*F.*) 5 locus-64 taxa phylogeny and GenBank (GB) accession numbers for the loci newly sequenced (in blue) or sampled in GB.

Collection(s) number(s)	Taxon	ITS	*RPB*2	*TEF*-1	ß-tubulin	calmodulin
PUF035	*F. acuminatum*	KT323128	JF741183	JF740857	GAU85567	HQ412345
NRRL13308	*F. acutatum*	NR_111142	JX237779	JF740745	U34431	AF158329
NRRL54939	*F. avenaceum*	KP170730	JX171663	KP170732	KP170733	NO
CBS 110306	*F. biseptatum*	EU926255	NO	EU926322	EU926388	NO
NRRL34033	*F. brachygibbosum*	GQ505450	GQ505482	GQ505418	KJ544190	GQ505388
NRRL13371	*F. buharicum*	FBU34581	JX171563	NO	NO	NO
NRRL52744	*F. commune*	KR082312	JF741146	JF740820	AY329043	NO
NRRL13459	*F. concolor*	GQ505763	GQ505852	JF740869	FCU61549	GQ505585
NRRL25475	*F. culmorum*	AF006342	JX171628	KT008433	AB820711	NO
CBS 122570	*F. decemcellulare*	HQ897815	HQ897760	KM231937	KM232070	KM231382
CBS 120718	*F. delphinoides*	NR_130680	KR674023	KR673915	KM232056	KM231363
CBS 108944	*F. dimerum*	JQ434586	KM232363	EU926334	JQ434533	KM231365
SIN 25	*F. cf. dimerum*	KU720853	KU720913	KU720950	KU720757	KU720798
CBS 116517	*F. domesticum*	JQ434584	HQ897694	EU926286	JQ434531	KM231366
NRRL13566	*F. fujikuroi*	NR_111889	JX171570	KR071741	AB725606	AF158332
PUF033	*F. graminearum*	GZU34578	JF741196	JF740871	U34436	HQ412343
298378	*F. incarnatum*	KF255449	KF255548	KF255493	KP453982	JN614903
NRRL43433	*F. keratoplasticum*	DQ790517	DQ790561	DQ790473	FN554619	NO
NRRL13622	*F. lateritium*	KT004553	JX171571	JF740854	KJ001545	NO
NRRL34123	*F. lichenicola*	KJ768839	EU329635	KP903345	NO	NO
CBS 632.76	*F. lunatum*	JQ434583	JX171648	EU926291	KM232057	KM231367
CBS 176.31	*F. nectrioides*	EU926245	JX171591	EU926312	KM232055	KM231362
CBS 562.70	*F. neocosmosporiellum*	AY381138	JX171610	JX997934	KM232067	KM231377
NRRL13448	*F. nygamai*	KR071693	JF741116	JF740790	KF466441	AF158326
PS2AUST186	*F. oxysporum*	NO	KJ397228	KJ397048	NO	KJ397012
PS2AUST68	*F. oxysporum*	NO	KJ397252	KJ397072	NO	KJ397036
PS1AUST589	*F. oxysporum*	NO	KJ397243	KJ397063	NO	KJ397027
SIN5	*F. cf. oxysporum*	KU720841	KU720901	KU720953	KU720745	KU720801
SIN8	*F. cf. oxysporum*	KU720843	KU720903	KU720954	KU720747	KU720802
SIN68	*F. cf. oxysporum*	KU720875	KU720922	KU720964	KU720777	KU720813
SIN101	*F. cf. oxysporum*	KU720890	KU720944	KU720975	KU720792	KU720826
CBS 317.34	*F. penzigii*	KM231795	KM232362	EU926324	EU926390	KM231364
SIN30	*F. petroliphilum*	KU720856	KU720916	NO	KU720760	NO
SIN90	*F. petroliphilum*	KU720888	KU720942	KU720992	KU720790	KU720827
FEP-16	*F. petroliphilum*	MZ396650	MZ421336	MZ421337	MZ421334	MZ421335
SIN30	*F. aff. petroliphilum*	KU720856	KU720916	NO	KU720760	NO
SIN84	*F. aff. petroliphilum*	KU720883	KU720939	KU720995	KU720785	NO

NRRL22944	*F. proliferatum*	FPU34558	JX171617	AF160280	KM232082	AF158333
SIN26	*F. aff. proliferatum*	KU720854	KU720914	KU720977	KU720758	NO
SIN27	*F. aff. proliferatum*	KU720855	KU720915	NO	KU720759	KU720838
SIN78	*F. aff. proliferatum*	KU720880	KU720936	KU720978	KU720782	NO
SIN59	*F. cf. proliferatum*	KU720868	KU720927	KU720980	KU720772	NO
FRC T-0962	*F. poae*	KJ755667	GQ915495	GQ915511	GQ915445	NO
NRRL22901	*F. redolens*	FSU34565	JX171616	JF740748	U34423	NO
NRRL13999	*F. sacchari*	KR071659	JX171580	AF160278	KC571397	AF158331
SIN 107	*F. cf. sacchari*	KU720892	KU720946	NO	KU720794	NO
Myc133	*F. sp.*	KU720893	KU720948	KU720949	KU720795	KU720797
CBS 146.95	*F. sambucinum*	KM231813	JX171606	KM231941	KM232078	KM231391
SIN 52	*F. aff. solani*	KU720861	NO	KU720984	KU720765	NO
SIN 54	*F. aff. solani*	KU720863	NO	KU720985	KU720767	KU720835
SIN 74	*F. aff. solani*	KU720878	KU720934	KU720990	KU720780	KU720831
SIN 76	*F. aff. solani*	KU720879	KU720935	KU720991	KU720781	KU720832
MPIV	*F. cf. solani*	AF178394	EU329496	AF178328	NO	NO
MPVII	*F. cf. solani*	DQ094312	EU329516	AF178353	NO	NO
5514	*F. sporotrichioides*	JX162394	HQ154452	HM744663	HQ141639	KF467348
CBS 101573	*F. striatum*	KM231798	KM232365	KM231927	KM232060	KM231370
NRRL22748	*F. torulosum*	JX534330	JX171615	JF740840	NO	NO
NRRL22172	*F. verticillioides*	FVU34555	JX171598	JF740741	AB587059	AF158315
NRRL31041	*F. virguliforme*	AY220239	JX171643	AY220193	HM453328	EF408397
CBS 837.85	*Fusicolla aquaeductuum*	KM231823	JX171590	KM231955	KM232094	KM231406
CBS 634.76	*Fusicolla violacea*	KM231824	HQ897696	KM231956	KM232095	KM231407
BBA 62172	*Mycrocera ciliata*	HQ897818	HQ897764	NO	EU860030	NO
CBS 310.34	*Microcera coccophila*	HQ897794	JX171576	JF740692	KC291937	KM231410
CBS 264.65	*Ilyonectria radicicola*	AF220968	KM232334	HM364341	HM352856	KM231317

Supplementary Table S2. List of strains of endophytic fungi isolated from *Posidonia oceanica*.

<table>
<tr><th>Nº</th><th>Plant part</th><th>Collection number</th><th>Closest relative in NCBI*</th><th>MIC MRSA (µg/ml)</th><th>MIC PA (µg/ml)</th></tr>
<tr><td>1</td><td>Leaf</td><td>FEP 10</td><td>Acremonium sp.</td><td rowspan="2">> 256</td><td rowspan="25">> 256</td></tr>
<tr><td>2</td><td>Leaf</td><td>FEP 4</td><td>Cladosporium sp.</td></tr>
<tr><td>3</td><td>Leaf</td><td>FEP 11</td><td>Penicillium crustosum</td><td>64</td></tr>
<tr><td>4</td><td>Leaf</td><td>FEP 13</td><td>Penicillium crustosum</td><td rowspan="12">➢ 256</td></tr>
<tr><td>5</td><td>Leaf</td><td>FEP 12</td><td>Penicillium sp.</td></tr>
<tr><td>6</td><td>Leaf</td><td>FEP 15</td><td>Clonostachys cf. rosea</td></tr>
<tr><td>7</td><td>Leaf</td><td>PO 14</td><td>Lulworthiales sp.</td></tr>
<tr><td>8</td><td>Leaf</td><td>PO 15</td><td>Lulworthiales sp.</td></tr>
<tr><td>9</td><td>Leaf</td><td>PO 16</td><td>Lulworthiales sp.</td></tr>
<tr><td>10</td><td>Rhizome</td><td>FEP 5</td><td>Lulworthia sp.</td></tr>
<tr><td>11</td><td>Rhizome</td><td>FEP 8</td><td>Lulworthia sp.</td></tr>
<tr><td>12</td><td>Rhizome</td><td>FEP 21</td><td>Paralulworthia cf. gigaspora</td></tr>
<tr><td>13</td><td>Rhizome</td><td>FEP 18</td><td>Posidoniomyces cf. atricolor</td></tr>
<tr><td>14</td><td>Rhizome</td><td>FEP 19</td><td>Sordariomycetes sp.</td></tr>
<tr><td>15</td><td>Rhizome</td><td>FEP 20</td><td>Sordariomycetes sp.</td></tr>
<tr><td>16</td><td>Rhizome</td><td>FEP 16*</td><td>Fusarium petroliphilum*</td><td>32</td></tr>
<tr><td>17</td><td>Rhizome</td><td>PO 1</td><td>Paralulworthia gigaspora</td><td rowspan="9">> 256</td></tr>
<tr><td>18</td><td>Rhizome</td><td>PO 2</td><td>Paralulworthia gigaspora</td></tr>
<tr><td>19</td><td>Rhizome</td><td>PO 3</td><td>Paralulworthia gigaspora</td></tr>
<tr><td>20</td><td>Rhizome</td><td>PO 4</td><td>Paralulworthia gigaspora</td></tr>
<tr><td>21</td><td>Rhizome</td><td>PO 5</td><td>Paralulworthia gigaspora</td></tr>
<tr><td>22</td><td>Rhizome</td><td>PO 6</td><td>Paralulworthia gigaspora</td></tr>
<tr><td>23</td><td>Rhizome</td><td>PO 7</td><td>Paralulworthia gigaspora</td></tr>
<tr><td>24</td><td>Rhizome</td><td>PO 8</td><td>Paralulworthia gigaspora</td></tr>
<tr><td>25</td><td>Rhizome</td><td>PO 10</td><td>Epicoccum nigrum</td></tr>
</table>

26	Rhizome	PO 11	*Fusarium* sp.		
27	Rhizome	PO 12	*Motierella indohii*		
28	Rhizome	PO 13	Hypocreales sp.		
29	Root	FEP 14	*Fusarium petroliphilum*		
30	Root	FEP 17	*Fusarium petroliphilum*		
31	Root	FEP 22	*Paralulworthia* cf. *gigaspora*		
32	Root	FEP 2	*Penicillium* sp.		
33	Root	FEP 9	*Penicillium* sp.		

* This strain was studied in this work.

Supplementary Table S3. Ingredients of artificial sea water used to prepare the growth media for fungi culture.

Element	Concentration
KBr	1 mM
NaCl	400 mM
$MgCl_2$	110 mM
$CaCl_2$	10 mM
KCl	9 mM
$SrSO_4$	0.2 mM
Na_2SO_4	25 mM
$NaHCO_3$	2 mM
H_3BO_3	0.5 mM

Supplementary Table S4. primers used for the qPCR tests

	forward	reverse
HU	ATCAATGCAGTTGCAGAGCA	CCAGTTTGAGGGTTACGACCT
hld	TAATTAAGGAAGGAGTGATTTCAATG	TTTTTAGTGAATTTGTTCACTGTGTC
spa	CGGCACTACTGCTGACAAAA	AACGCTGCACCTAAGGCTAA
oprF	GGTTACTTCCTGACCGACGA	TCGCTGTTGATGTTGGTGAT
lasB	AAGCCATCACCGAAGTCAAG	GTAGACCAGTTGGGCGATGT
rhlA	CGAGGTCAATCACCTGGTCT	GACGGTCTCGTTGAGCAGAT
pqsA	CAATACACCTCGGGTTCCAC	TGAACCAGGGAAAGAACAGG
