# Supplementary material for: Combination of Pseudo-LC-NMR and HRMS/MS-Based Molecular Networking for the Rapid Identification of Antimicrobial Metabolites From Fusarium petroliphilum
Source: Front Mol Biosci. 2021 Oct 22;8:725691. doi: 10.3389/fmolb.2021.725691 (PMC8569130; doi:10.3389/fmolb.2021.725691)
Supplement: Supplementary file 3 [file DataSheet1.PDF]

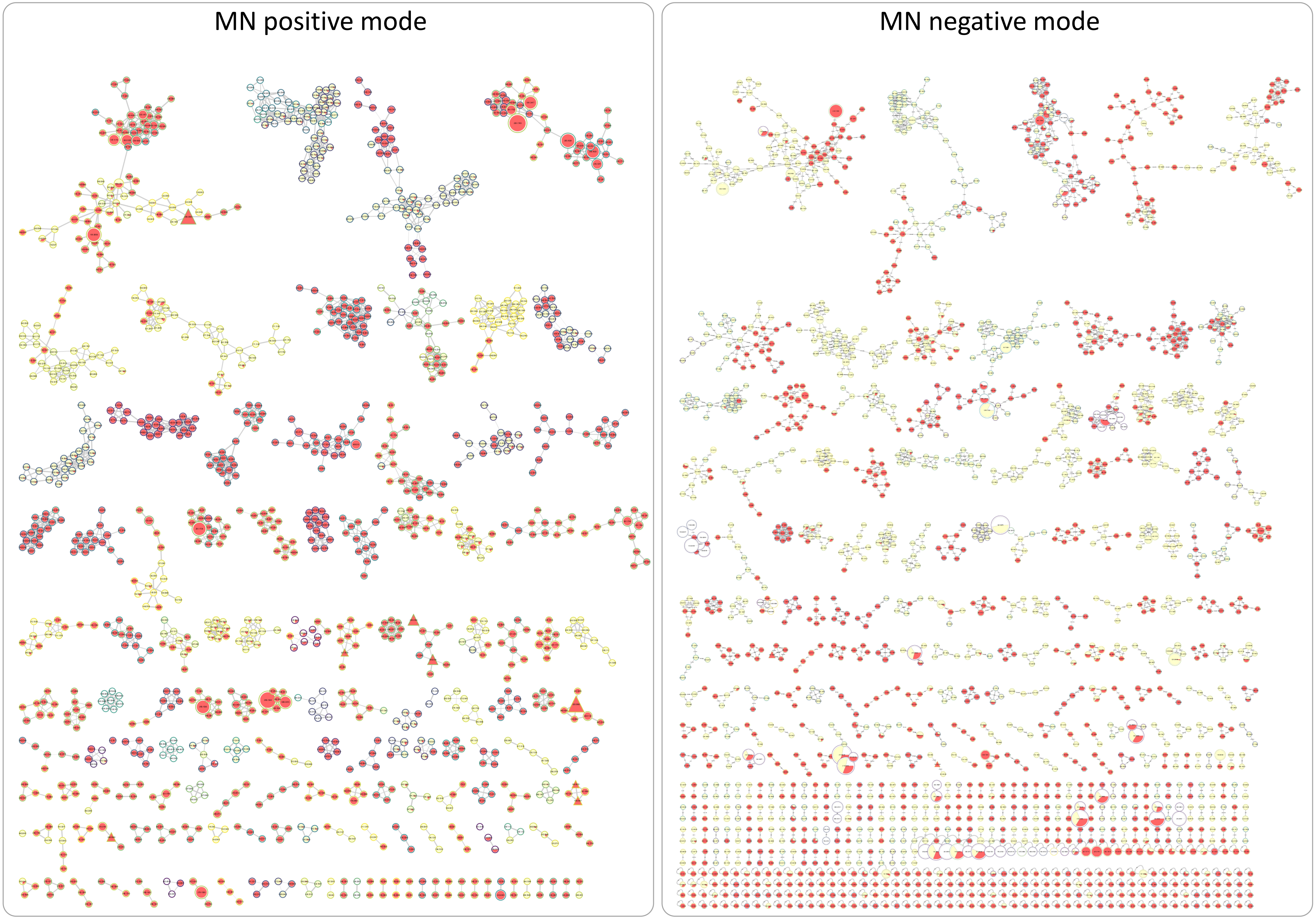

Supplementary Figure S1. Full view of molecular networks which created based on UHPLC-HRMS/MS analysis of the crude extract of *F. petroliphilum* in NI and PI modes. Nodes in red represent a precursor mass that found in the extract, yellow nodes represent the culture media and white nodes for the blank solvent.

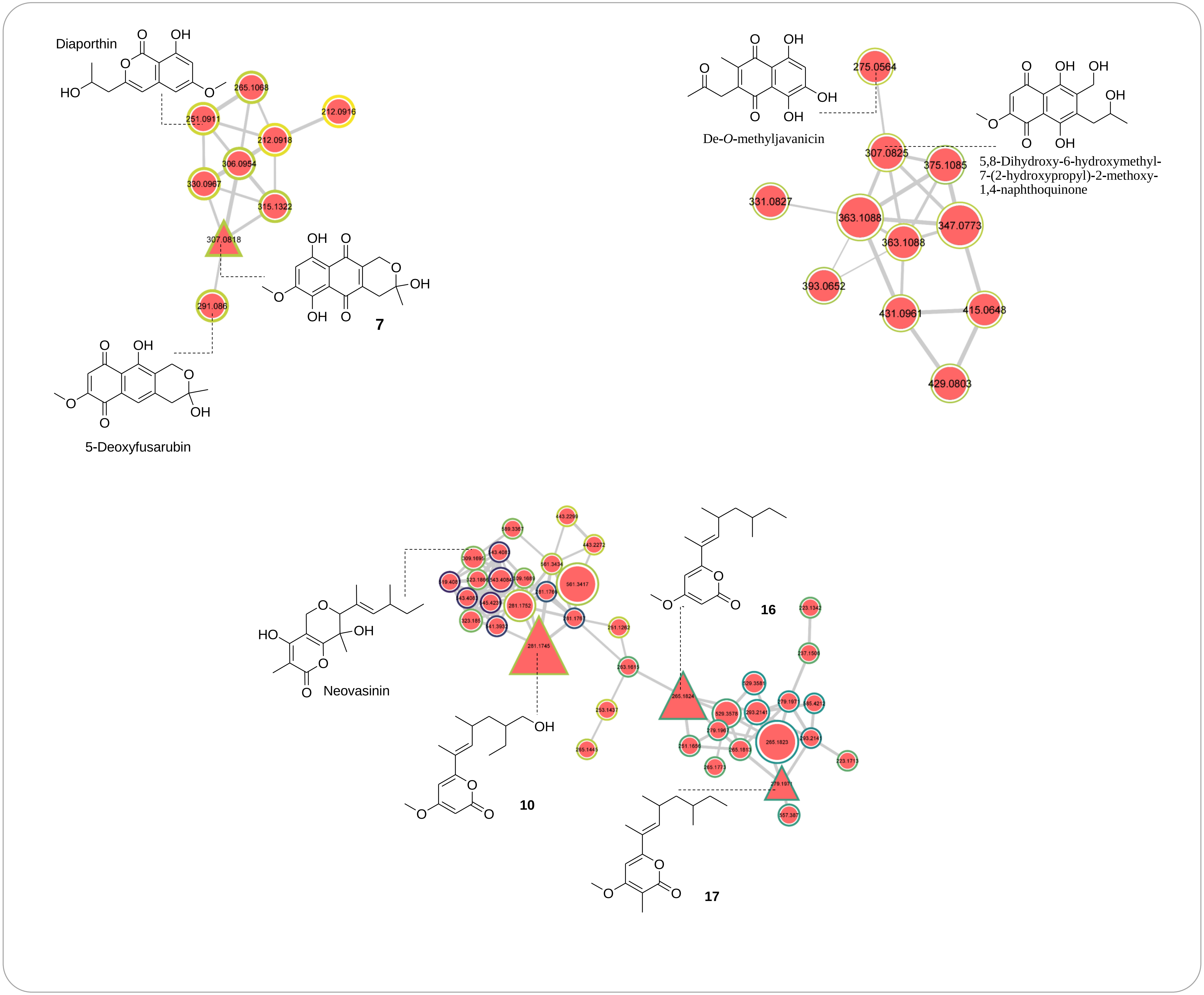

Supplementary Figure S2. Example of the annotation propagation assisted by MN and ISDB-DNP in PI mode. Triangle nodes represent isolated compounds.

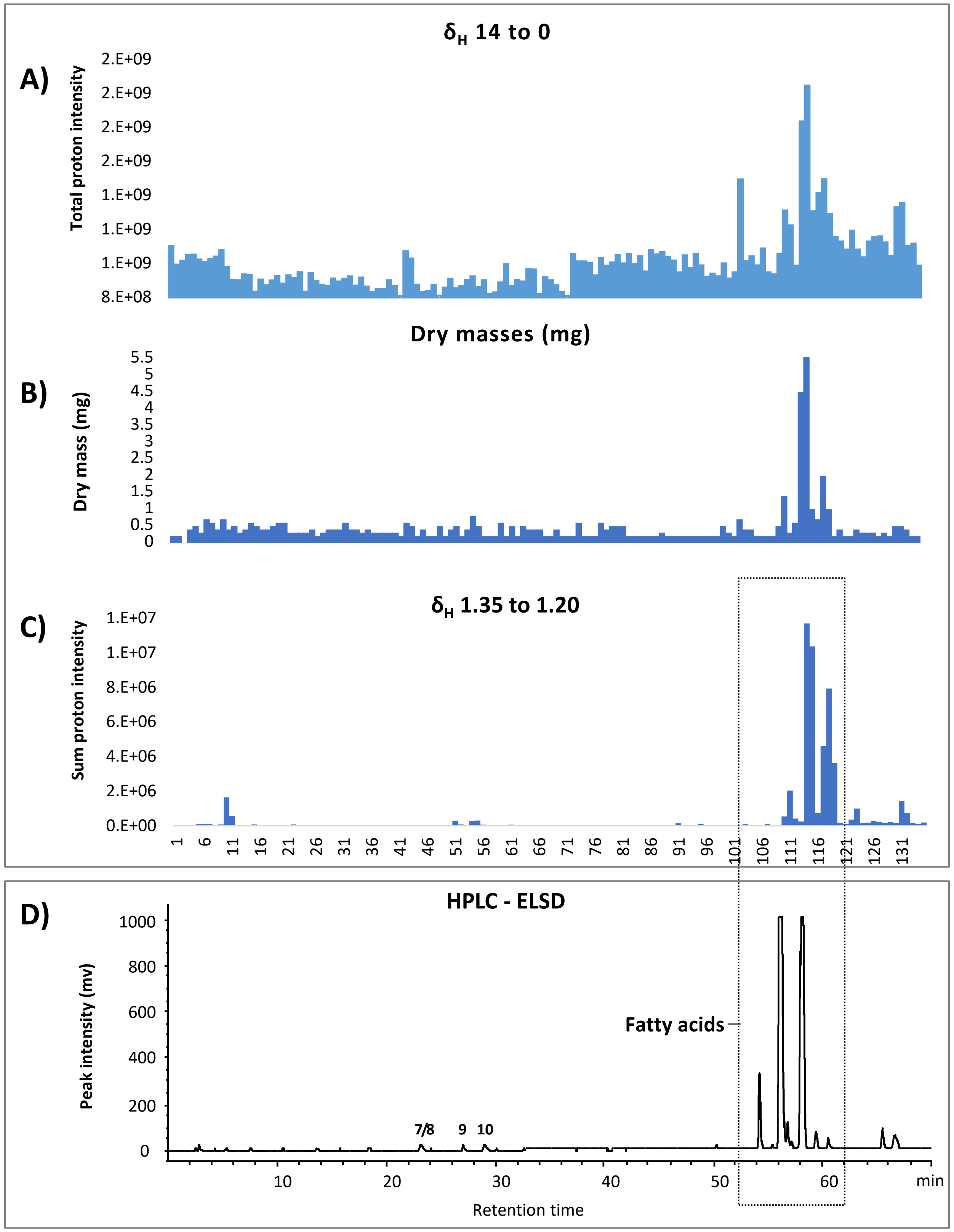

Supplementary Figure S3. (A) Graphical representation of the sum of intensities of all ^{1}H-NMR signals (δ_H 14 to 0). **(B)** Quantities in (mg) of the dried fractions obtained by semi-prep HPLC fractionation. **(C)** Sum intensities of ^{1}H-NMR signals characteristic for long chain fatty acids (δ_H 1.35 to 1.20). **(D)** HPLC-ELSD trace of the crude ethyl acetate extract of *F. petroliphilum*. The square highlights the 3 main peaks which correspond to fatty acids and demonstrate a good fit between the ELSD and NMR profiles .

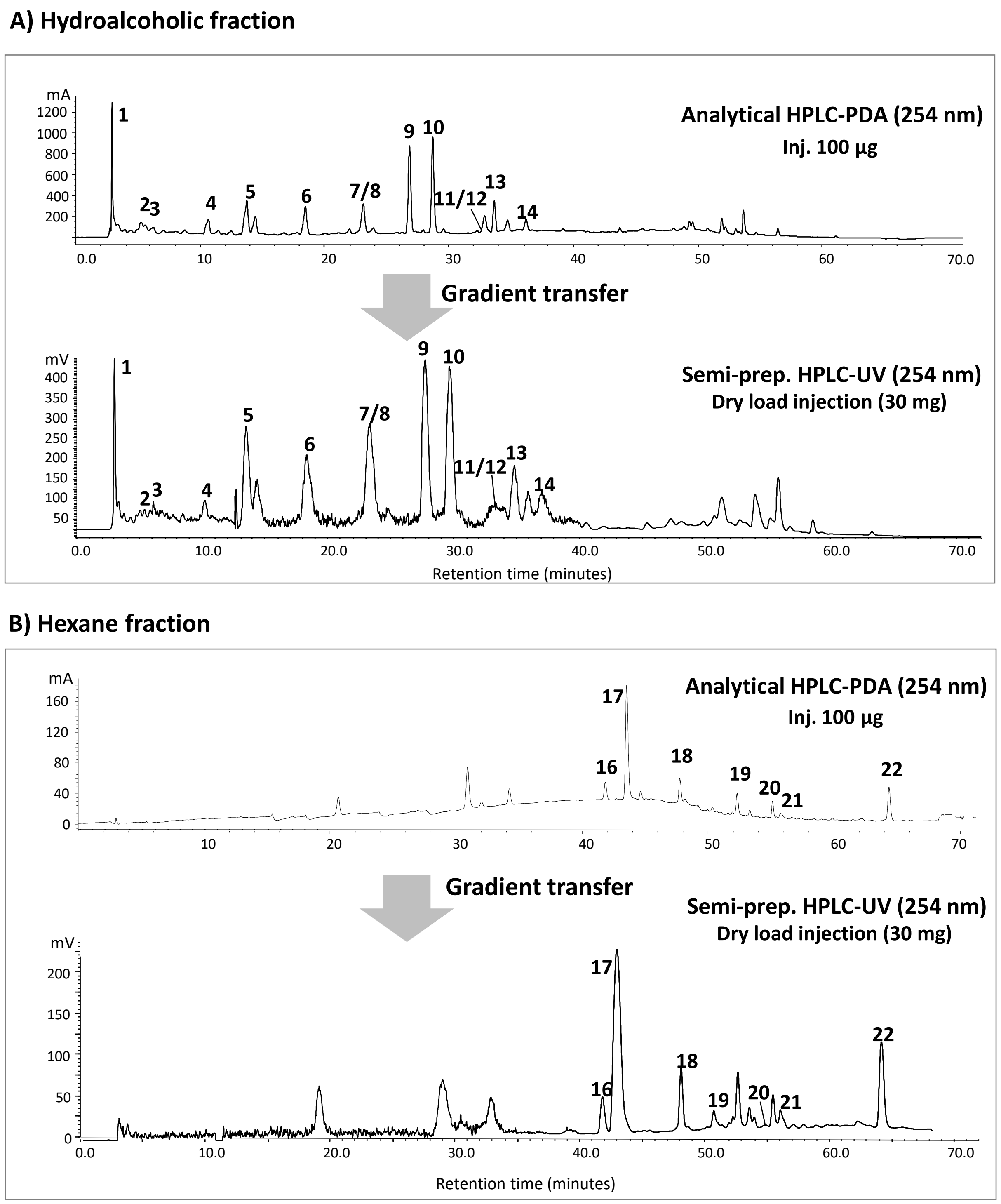

Supplementary Figure S4. Chromatograms of HPLC-PDA for the two main fractions obtained by liquid-liquid separation. Geometric gradient transfer was applied to ensure the same peak selectivity. **(A)** The hydroalcoholic fraction, **(B)** The hexane fraction.
